# Palladium/GF-Phos-catalyzed asymmetric carbenylative amination to access chiral pyrrolidines and piperidines†

**DOI:** 10.1039/d2sc03999k

**Published:** 2022-09-01

**Authors:** Yue Sun, Chun Ma, Zhiming Li, Junliang Zhang

**Affiliations:** Department of Chemistry, Fudan University 2005 Songhu Road Shanghai 200438 China zmli@fudan.edu.cn junliangzhang@fudan.edu.cn; Zhuhai Fudan Innovation Institute Hengqing District Zhuhai 519000 China

## Abstract

The cross-coupling of *N*-tosylhydrazones has emerged as a powerful method for the construction of structurally diverse molecules, but the development of catalytic enantioselective versions still poses considerable challenges and only very limited examples have been reported. We herein report an asymmetric palladium/GF-Phos-catalyzed carbenylative amination reaction of *N*-tosylhydrazones and (*E*)-vinyl iodides pendent with amine, which allows facile access to a range of chiral pyrrolidines and piperidines in good yields (45–93%) with up to 96.5 : 3.5 *er*. Moreover, mild conditions, general substrate scope, scaled-up preparation, as well as the efficient synthesis of natural product (−)-norruspoline are practical features of this method.

## Abstract 2

An efficient asymmetric palladium/GF-Phos-catalyzed carbenylative amination reaction to access structurally diverse chiral pyrrolidines and piperidines in good yields with high chemo-, regio- and enantioselectivities has been developed.

## Graphical abstract

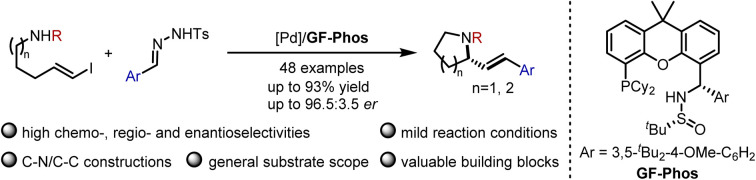

*N*-tosylhydrazones, readily prepared from aldehydes or ketones, served as a safe source of carbene precursors and have attracted much attention of chemists.^1^*N*-tosylhydrazone-mediated applications have been continuously developed, such as cyclopropanation or cyclopropenation, X–H insertion, ylide formation, cycloaddition, aza-Wacker-type cyclization, asymmetric allylic substitution, *etc.*^2^ Among them, transition-metal-catalyzed cross-coupling is one of the powerful protocols for C–X or C=C bond formation in organic synthesis involving versatile intermediates, of which *in situ* generation of diazo compounds and carbene migratory insertion are considered key steps.^3–5^ Over the past decades, considerable progress has been made in the asymmetric cross-coupling reactions of *N*-tosylhydrazones with various coupling partners, including cyclobutanols, terminal alkynes, silacyclobutanes and so on.^4^ Relatively, only a few examples focus on the cross-coupling reactions of aryl halides with *N*-tosylhydrazones involving benzyl metal intermediates [Scheme 1A, eqn. (a)].^6^ For example, Gu,^6*a*^ Wu,^6*b*^ Lassaletta^6*c*^ and coworkers have developed a palladium-catalyzed asymmetric synthesis of axial chiral compounds from aryl bromides and *N*-tosylhydrazones, ending with β-H elimination. Very recently, we realized palladium/GF-Phos catalyzed asymmetric three component cross-coupling reactions of aryl halides, *N*-tosylhydrazones, with terminal alkynes.^6*f*^ In contrast, much less progress has been made in *N*-tosylhydrazone-based carbenylative insertions from vinyl halides, which would generate a π-allylic metal intermediate followed by nucleophile attack, providing a unique approach for building C–X bonds, especially for *N*-heterocyclic compounds [Scheme 1A, eqn. (b)].^7^*N*-heterocycles are important structural motifs for the development of various types of valuable chemicals and materials.^8^ Importantly, optically active 2-substituted pyrrolidine and piperidine derivatives are privileged scaffolds in many natural products and pharmaceuticals with a wide range of biological activities,^9^ as well as the backbone of organocatalysts in asymmetric catalysis (Fig. 1).^10^

**Scheme 1:**
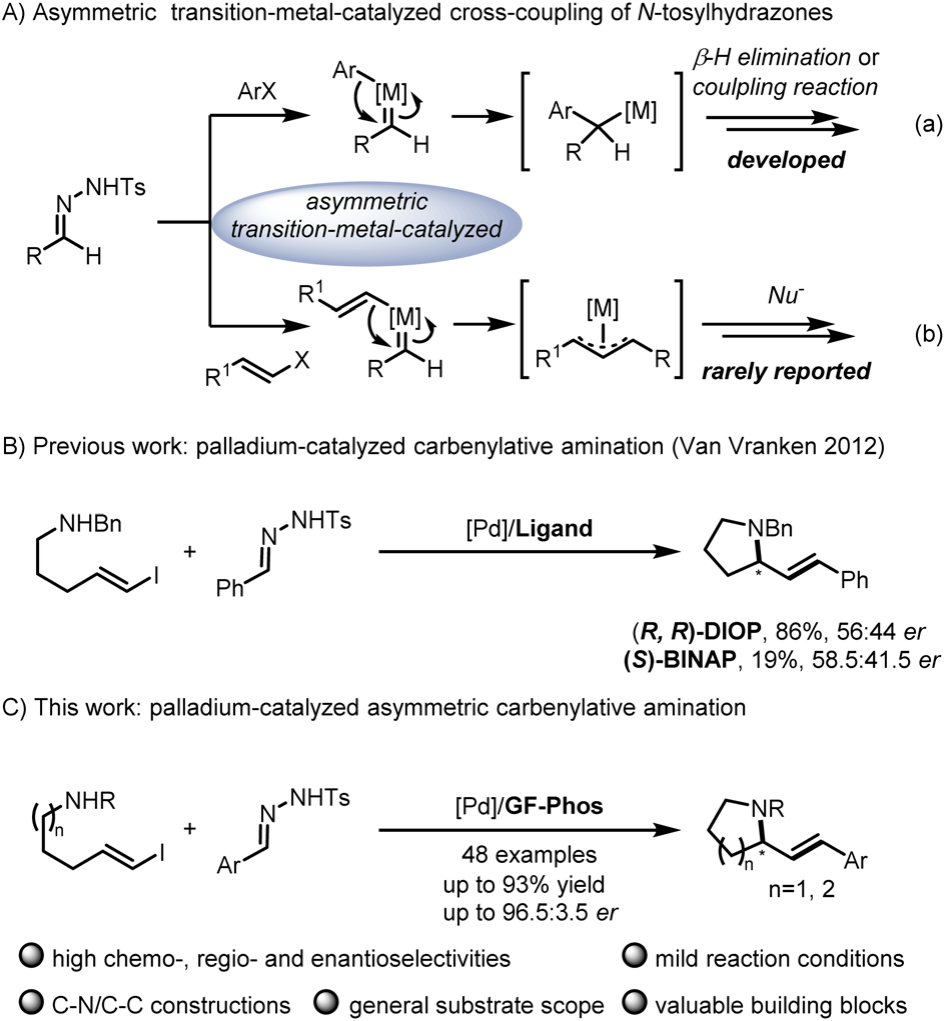
Asymmetric transition-metal-catalyzed carbenylative cross-coupling reactions.

**Fig. 1:**
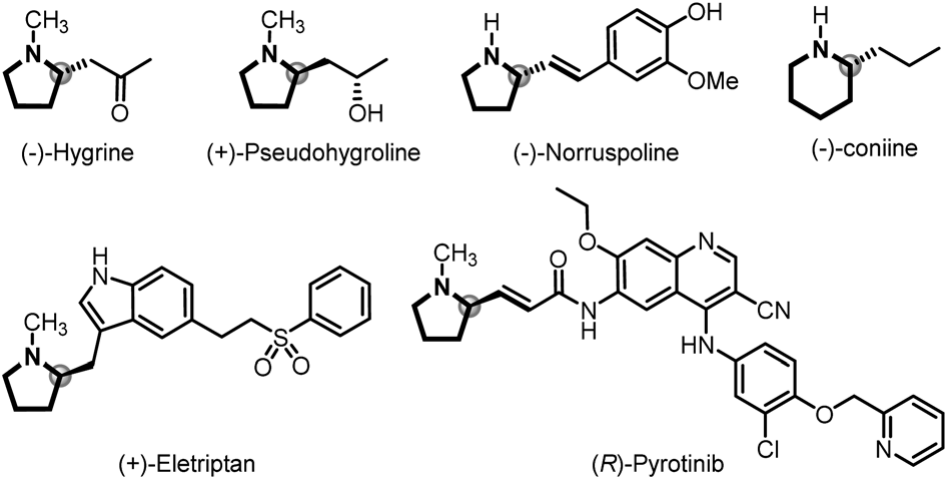
Selected natural products and pharmaceuticals containing chiral 2-substituted pyrrolidine and piperidine units.

Notably, Van Vranken and coworkers reported an elegant palladium-catalyzed carbenylative amination reaction of *N*-tosylhydrazones and (*E*)-vinyl iodides pendent with amine, providing facile access to pyrrolidine and piperidine ring systems that are common to alkaloid natural products (Scheme 1B).^11^ Unfortunately, only up to 58.5 : 41.5 *er* was obtained after they made a lot of efforts to screen a series of chiral phosphine ligands, indicating that this asymmetric reaction indeed poses considerable challenges in addition to competitive side reactions such as the dimerization of vinyl iodides,^12^ the formation of diene *via* the palladatropic rearrangement/β-H elimination or allene *via* β-H elimination from C_sp_^2^,^13^ and the π-allylpalladium intermediate trapped by the byproduct sulfinic acid salt.^14^ Given the significance of chiral pyrrolidines and piperidines as core structures in alkaloid natural products, the development of an asymmetric version of this elegant carbenylative amination reaction is highly desirable. In recent years, our group has developed a series of chiral sulfinamide phosphine ligands (so-called Sadphos), which showed unique potential in asymmetric transition-metal catalysis,^6*f*,15^ so we wondered whether Sadphos could address this challenging asymmetric carbenylative amination reaction (Scheme 1C).

Initially, our study began with (*E*)-vinyl iodide 1a and *N*-tosylhydrazone 2a in the presence of Pd_2_(dba)_3_, *t*-BuOLi, Et_3_N, and triethylbenzylammonium chloride (TEBAC) in THF at 30 °C. A series of commercially available chiral ligands were first screened (Fig. 2). Only (*R*, *R*)-DIOP (L1), (*R*)-DTBM-SegPhos (L3) and (*R*)-MOP (L4) provided the desired product 3aa with poor enantioselectivity and other ligands such as (*R*, *R*)-Ph-BPE (L2), (*R*, *S*)-Josiphos (L5) and (*S*, *S*)-^*i*^Pr-FOXAP (L6) showed low reactivity. We next turned to systematically investigate Sadphos, such as Wei-Phos,^16^ Xiao-Phos,^15*d*,17^ Ming-Phos,^15*a*,18^ Xu-Phos,^15*b*,19^ Xiang-Phos^20^ and PC-Phos^15*c*,21^ (Fig. 2). To our delight, PC1 delivered 3aa in 32% yield and 85.5 : 14.5 *er*. Inspired by this result, we further screened PC2–PC5 which vary in the substituent of phenyl, but unfortunately none of them showed better results. Surprisingly, the reactivity of this reaction could be greatly improved with our recently developed GF-Phos GF1, delivering 71% yield. When steric hindered *tert*-butyl groups were introduced on the phenyl group (GF2), the product 3aa was obtained in 77% yield with 91.5 : 8.5 *er*. After screening different palladium catalysts and solvents (Table 1, entries 1–10), the *er* value has been slightly increased. Additionally, lowering reaction temperature led to an increase in enantioselectivity but a decrease in yield (Table 1, entry 11).

**Fig. 2:**
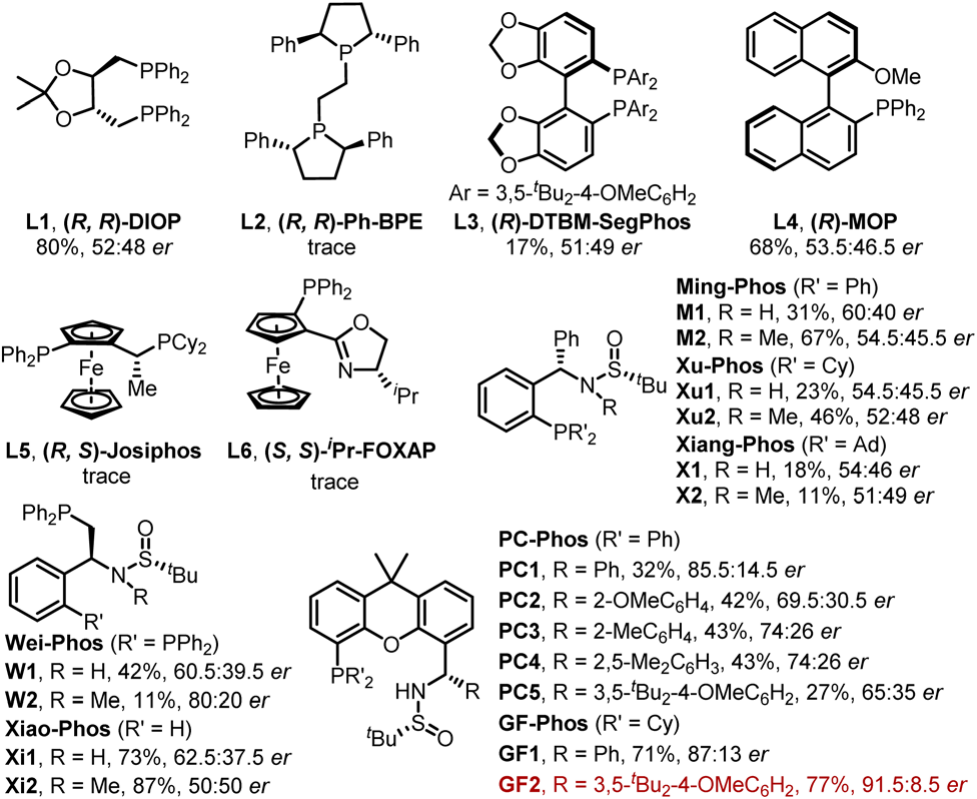
Screened chiral ligands.

**Table 1:** Optimization of reaction conditions^a^

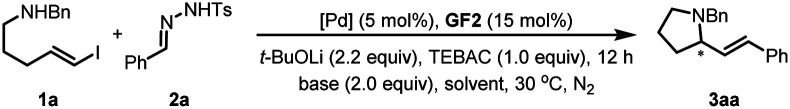					
Entry	[Pd]	Base	Solvent	Yield^b^ (%)	*er* c
1	Pd_2_(dba)_3_	Et_3_N	THF	77	91.5 : 8.5
2	Pd(acac)_2_	Et_3_N	THF	89	86.5 : 13.5
3	Pd(OAc)_2_	Et_3_N	THF	82	88 : 15
4	PdBr_2_	Et_3_N	THF	78	88 : 12
5	Pd_2_(dba)_3_·CHCl_3_	Et_3_N	THF	75	92 : 8
6	Pd_2_(dba)_3_·CHCl_3_	Et_3_N	Toluene	23	92.5 : 7.5
7	Pd_2_(dba)_3_·CHCl_3_	Et_3_N	DMF	90	80 : 20
8	Pd_2_(dba)_3_·CHCl_3_	Et_3_N	MTBE	28	93 : 7
9	Pd_2_(dba)_3_·CHCl_3_	Et_3_N	1,4-Dioxane	38	88.5 : 11.5
10	Pd_2_(dba)_3_·CHCl_3_	Et_3_N	2-Me-THF	89	93 : 7
11^d^	Pd_2_(dba)_3_·CHCl_3_	Et_3_N	2-Me-THF	26	94.5 : 5.5
12	Pd_2_(dba)_3_·CHCl_3_	DABCO	2-Me-THF	76	94 : 6
13	Pd_2_(dba)_3_·CHCl_3_	Cs_2_CO_3_	2-Me-THF	93	92.5 : 7.5
14	Pd_2_(dba)_3_·CHCl_3_	KOH	2-Me-THF	89	93 : 7
15	Pd_2_(dba)_3_·CHCl_3_	None	2-Me-THF	83	93 : 7
16^e^	Pd_2_(dba)_3_·CHCl_3_	None	2-Me-THF	69	88 : 12
17^f^	Pd_2_(dba)_3_·CHCl_3_	None	2-Me-THF	81	94.5 : 5.5

a Reaction conditions: 1a (0.1 mmol), 2a (0.16 mmol), [Pd] (5 mol%), GF2 (15 mol%), t-BuOLi (2.2 equiv.), TEBAC (1.0 equiv.), base (2.0 equiv.) in 0.1 M solvent at 30 °C for 12 h.

b Determined by GC analysis with *n*-tetradecane as an internal standard.

c The *er* value was determined by chiral HPLC.

d 15 °C for 12 h.

e Without TEBAC.

f 15 mol% Ag_2_CO_3_. THF = tetrahydrofuran. MTBE = *tert*-butyl methyl ether. DMF = *N*,*N*-dimethylformamide. DCE = 1,2-dichloroethane. DMSO = dimethyl sulfoxide.

We also found that, besides *t*-BuOLi, there was little effect on the yield or enantioselectivity by changing another base. The study was therefore continued without it (Table 1, entries 12–15). Moreover, in the absence of TEBAC, 3aa was produced in only 69% yield and 88 : 12 *er*. TEBAC probably helps to increase the solubility of the anion of *N*-tosylhydrazones (Table 1, entry 16). Interestingly, we investigated a series of additives, and the results indicated that the addition of Ag_2_CO_3_ could further provide slightly higher enantioselectivity (94.5 : 5.5 *er*) (Table 1, entry 17, see the ESI for more details†).

The scope of the carbenylative amination reaction was then studied using the optimized reaction conditions (Table 2). A wide range of *N*-tosylhydrazones 2 bearing electron-withdrawing or donating groups at the *ortho*-, *meta*- or *para*-position of the phenyl ring were tested, giving the corresponding products 3aa–3aj in moderate to good yields with 92.5 : 7.5–96 : 4 *er*. The absolute configuration of 3ac was confirmed as *S* by single crystal X-ray diffraction analysis.^22^ Multisubstituted phenyl and naphthyl groups were also well-tolerated (3am, 3an, 3ap–3as). It is note-worthy that the 2,4,6-trimethylphenyl-substituted substrate delivered 3ao in 57% yield with 7/1 *E*/*Z* selectivity, probably due in part to the steric hindrance. Moreover, *N*-tosylhydrazones containing heterocycles reacted smoothly to furnish the expected products 3at–3aw. Besides diverse substituted *N*-tosylhydrazones 2, various kinds of vinyl iodide derivatives 1 with functional groups such as halides, methyl, *tert*-butyl, methoxy and 1-naphthyl at different positions on the phenyl ring also worked well and afforded 3ba–3ja in good yields. Surprisingly, when the protective group on the nitrogen atom was replaced by a *p*-toluenesulfonyl or *p*-nitrophenylsulfonyl group, the corresponding cyclic products 3ka, 3lx, and 3ly were successfully produced in high yields and enantioselectivities.

**Table 2:** Scope for enantioselective formation of pyrrolidines^a^

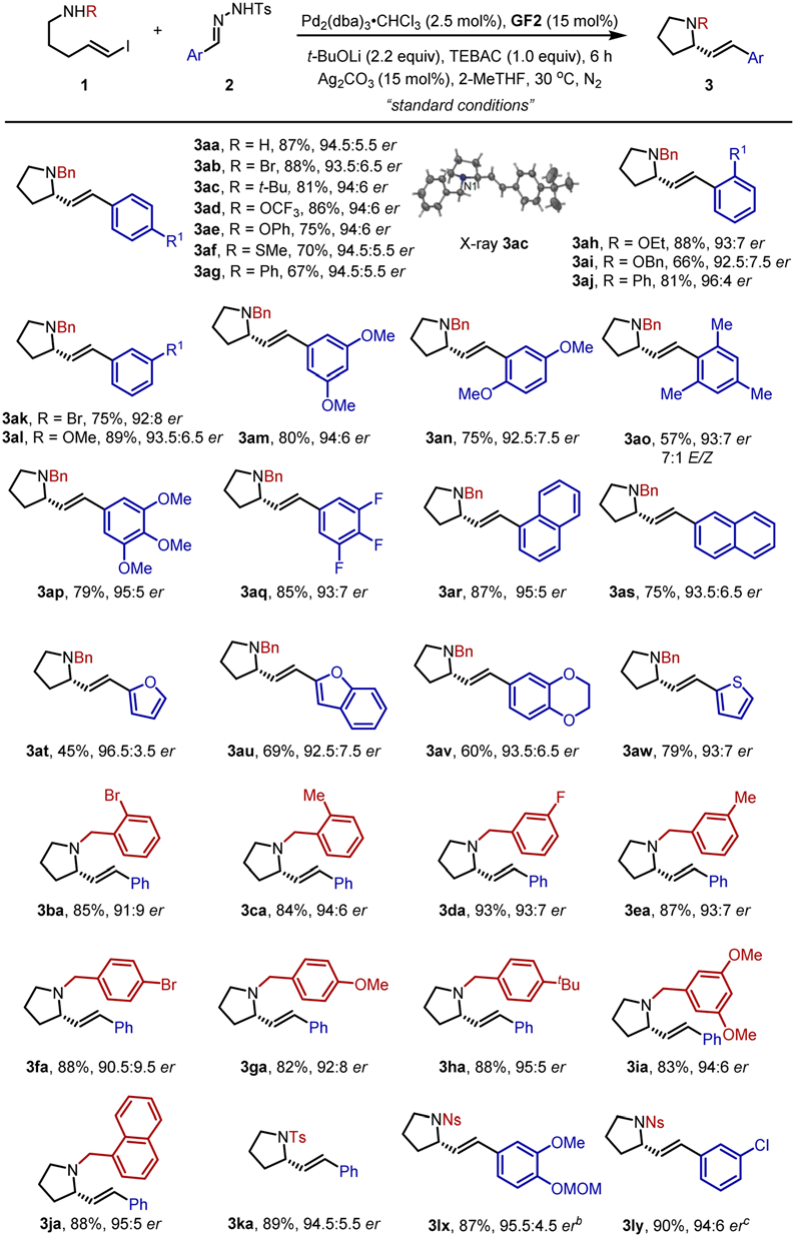

a Reaction conditions: 1 (0.3 mmol), 2 (0.48 mmol), Pd_2_(dba)_3_·CHCl_3_ (2.5 mol%). GF2 (15 mol%), *t*-BuOLi (2.2 equiv.), TEBAC (1.0 equiv.), Ag_2_CO_3_ (15 mol%) in 0.1 M 2-MeTHF at 30 °C for 6 h.

b 1.8 mmol scale, 24 h.

c 2.0 mmol scale, 20 h.

Subsequently, we further turned our efforts to the synthesis of piperidine derivatives. As shown in Table 3, the desired six-membered heterocycles 5aa–5dz could be obtained efficiently in 77–85% yields with 93.5 : 6.5–95 : 5 *er* under standard conditions. Similarly, the *p*-nitrophenylsulfonyl group was also a compatible partner to give 5ea in 81% yield with 93.5 : 6.5 *er*. In parallel, a variety of *N*-tosylhydrazones 2 mentioned above were studied, affording structurally diverse piperidines 5ab–5ar smoothly. In addition, 2-furan- and thienyl-substituted *N*-tosylhydrazones were transformed into 5at and 5aw in good yields with high *er* values.

**Table 3:** Scope for enantioselective formation of piperidines^a^

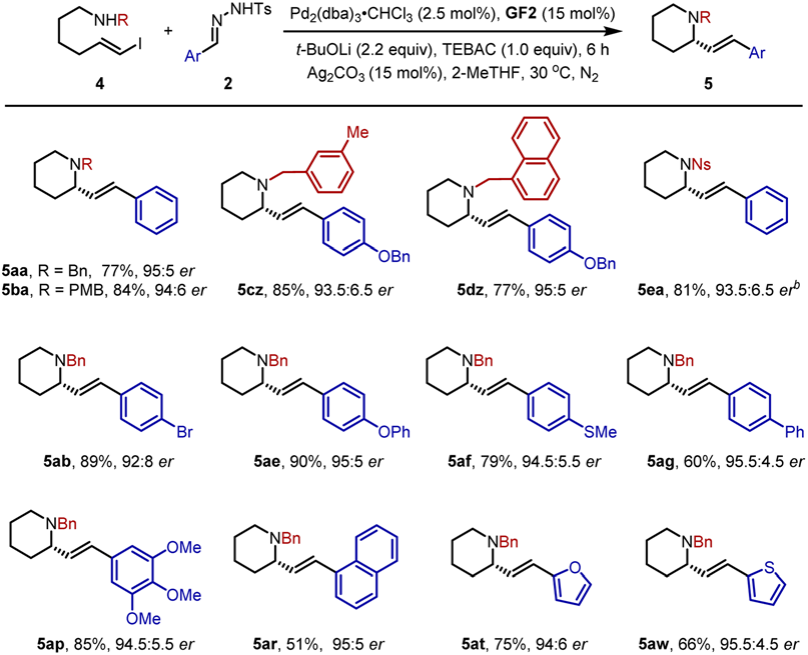

a Reaction conditions: 1 (0.3 mmol), 2 (0.48 mmol), Pd_2_(dba)_3_·CHCl_3_ (2.5 mol%), GF2 (15 mol%). *t*-BuOLi (2.2 equiv.), TEBAC (1.0 equiv.), Ag_2_CO_3_ (15 mol%) in 0.1 M 2-MeTHF at 30 °C for 6 h.

b 12 h.

To evaluate the synthetic utility of this asymmetric carbenylative amination reaction, we carried out a gram–scale reaction under standard conditions, providing the product 3aj in 85% yield with 95.5 : 4.5 *er* (Scheme 2a). Of note, a 2-step deprotection of 3lx with *p*-toluenethiol/K_2_CO_3_ and HCl (1 M) enabled the synthesis of natural product (−)-norruspoline in 51% overall yield. Additionally, replacing the protecting group of 3ly with the Boc group afforded 6 in 67% yield without the loss of enantioselectivity and it has been previously shown that 6 is a synthetic intermediate for the preparation of natural product (−)-indolizidine 201 (Scheme 2b).^23^ A linear relationship was demonstrated by a nonlinear effect study on the *ee* value of GF2 and product 3aa, which implied that the catalytically active structure contains only a single chiral ligand. (please find more details in the ESI†).

**Scheme 2:**
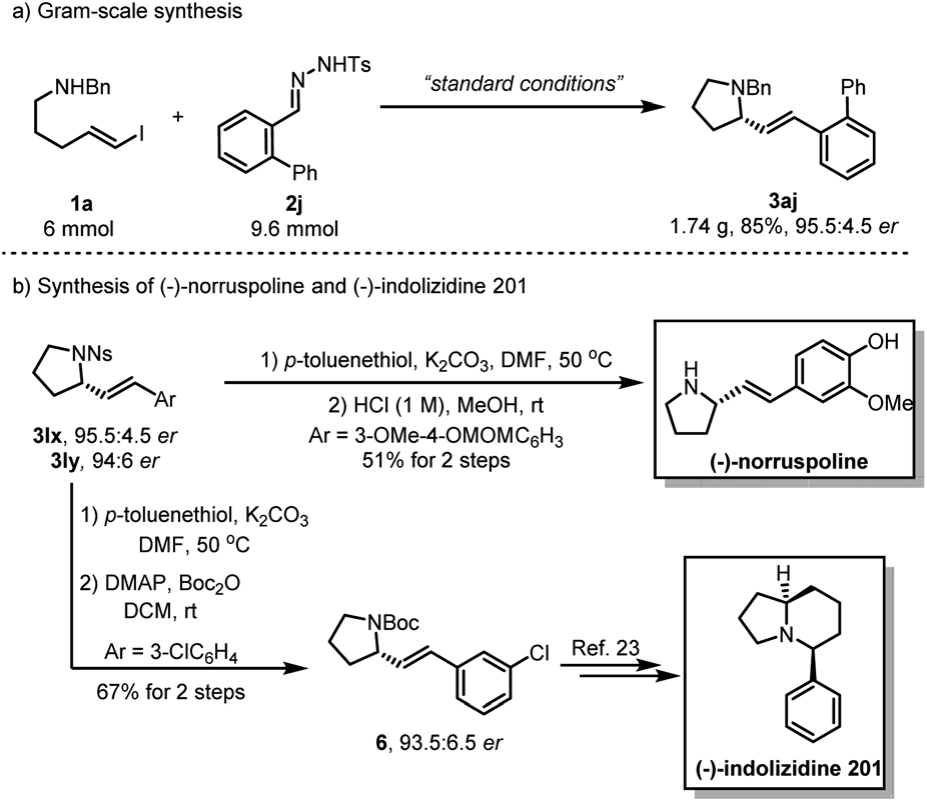
Gram-scale synthesis and synthetic applications.

Based on our study and previous work,^24^ a catalytic cycle pathway to rationalize the synthesis of chiral pyrrolidines is illustrated in Scheme 3. First, the oxidative addition of vinyl iodide 1a to a Pd^0^/GF-Phos complex would generate vinyl Pd^II^ species A. In the presence of a base, *N*-tosylhydrazone 2a*in situ* generated a diazo intermediate and formed palladium carbene B with vinyl Pd^II^ species A, followed by migratory insertion to generate the π-allylpalladium intermediate C, as displayed in path a. Alternatively, the reaction proceeds in a palladium carbene/oxidative addition sequence as in path b. Next, the nucleophilic attack of the nitrogen atom on π-allylpalladium delivered product 3aa and regenerated the Pd^0^ complex, thus completing the entire catalytic cycle. In light of the structure of the chiral ligand GF2 and the absolute configuration of product (*S*)-3, a chirality induction model for stereochemical induction was proposed (Fig. 3).

**Scheme 3:**
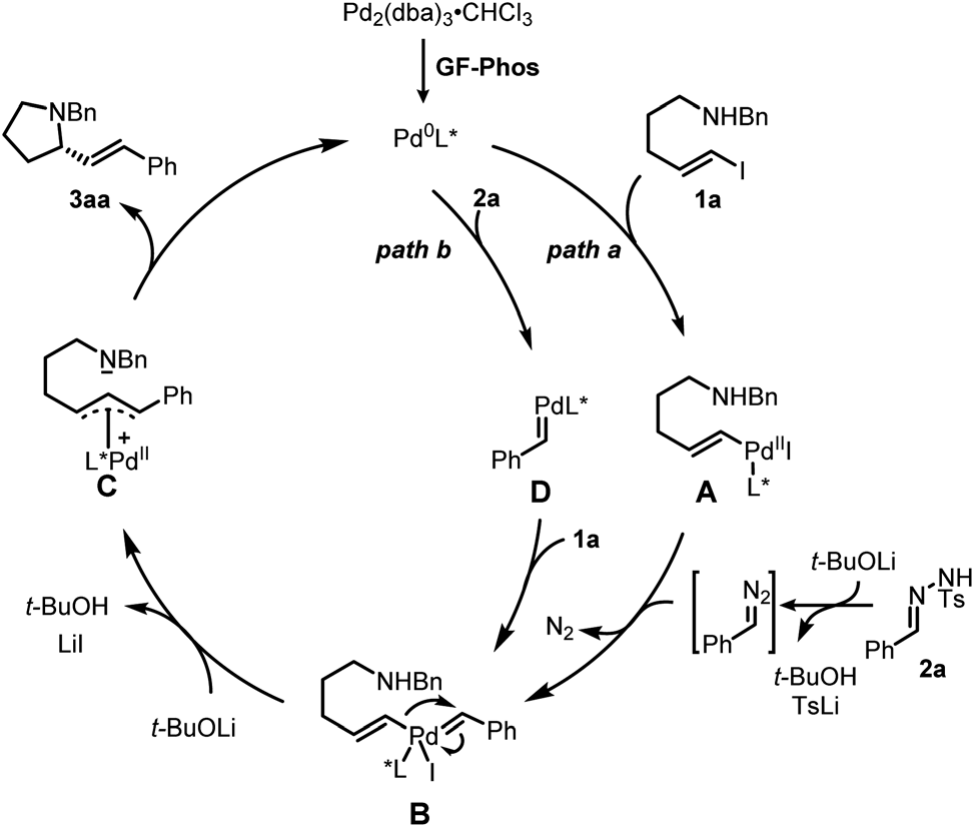
Proposed catalytic cycle.

**Fig. 3:**
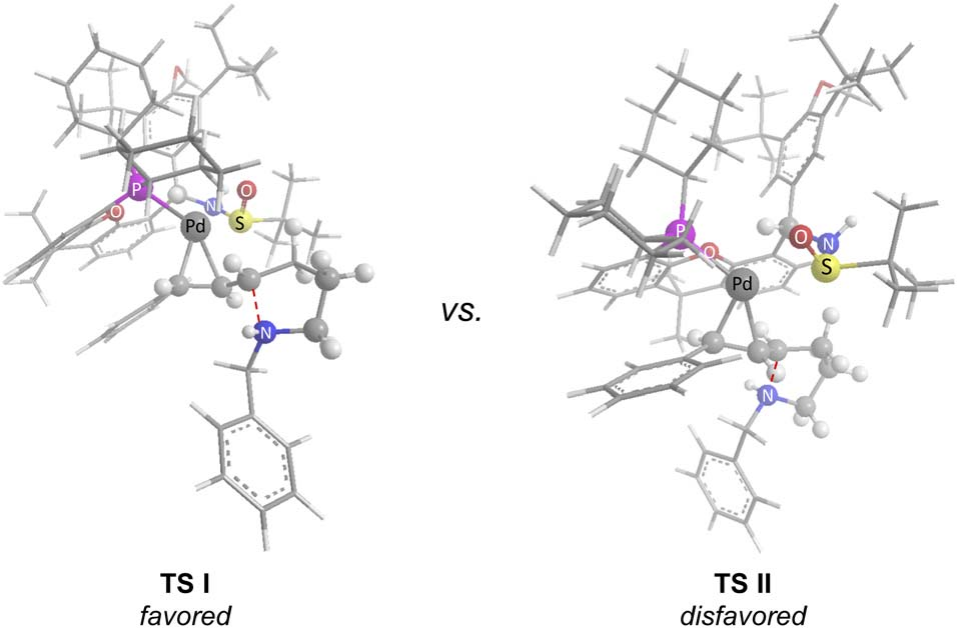
Proposed chirality induction model.

In conclusion, we have developed a palladium/GF-Phos catalyzed asymmetric carbenylative amination of (*E*)-vinyl iodides with *N*-tosylhydrazones *via* a carbene migratory insertion/Tsuji-Trost sequence to build C–N/C–C more efficiently. This catalytic system exhibits general functional group tolerance and enables rapid access to a variety of chiral 2-substituted pyrrolidines and piperidines in moderate to good yields with high chemo-, regio-, enantioselectivities under mild conditions. Our approach can be applied to the direct synthesis of significant natural product (−)-norruspoline and provides an alternative route for the formal synthesis of (−)-indolizidine 201.

## Data availability

All experimental data and detailed experimental procedures are available in the ESI.†

## Author contributions

Y. S. conducted the experiments and analysed the data. C. M. conducted the preparation of the starting materials. Z. L. and J. Z. directed the project. Y. S., Z. L. and J. Z. prepared the manuscript.

## Conflicts of interest

There are no conflicts to declare.

## Supplementary Material

SC-013-D2SC03999K-s001

SC-013-D2SC03999K-s002
