# Supplementary material for: Palladium/GF-Phos-catalyzed asymmetric carbenylative amination to access chiral pyrrolidines and piperidines
Source: Chem Sci. 2022 Sep 1;13(37):11150–5. doi: 10.1039/d2sc03999k (PMC9517724; doi:10.1039/d2sc03999k)
Supplement: SC-013-D2SC03999K-s001 [file SC-013-D2SC03999K-s001.pdf]

Electronic Supplementary Material (ESI) for Chemical Science.
This journal is © The Royal Society of Chemistry 2022

Supporting Information

Palladium/GF-Phos-Catalyzed Asymmetric Carbenylative Amination to Access Chiral Pyrrolidines and Piperidines

Yue Sun, Chun Ma, Zhiming Li* and Junliang Zhang*

Contents

1. General Information S2
2. Optimization of Reaction Conditions S3
3. General Procedure for the Synthesis of Substrates 1, 4 .... S6
4. General Procedure for the Synthesis of Products 3, 5 .... S12
5. Gram-scale Reaction and Synthetic Applications S45
6. Nonlinear Effect Study S48
7. References S49
8. X-ray Single Crystal Data for Compound 3ac S50
9. ^{1}H, ^{13}C, ^{19}F NMR Spectra S51

1. General Information

^{1}H NMR spectra, ^{13}C NMR spectra and ^{19}F NMR spectra were recorded on a Bruker 400 MHz spectrometer in $CDCl_3$. All signals are reported in parts per million (ppm), and were referenced to $CDCl_3$ (δ 7.26 ppm for ^{1}H NMR and 77.0 ppm for ^{13}C NMR) as the internal standard. Data for ^{1}H NMR spectra are reported as follows: chemical shift (δ ppm), multiplicity (s = singlet, d = doublet, t = triplet, q = quartet, dd = doublet of doublets, dt = doublet of triplets, td = triplet of doublets, m = multiplet), coupling constant (Hz) and integration. HRMS analysis was performed on a Q-TOF mass analyzer using the ESI ionization method. Optical rotation values were measured with instruments operating at λ = 589 nm, corresponding to the sodium D line at the temperatures indicated. The *er* value was determined by HPLC with a chiral stationary phase using a Chiralpak AD-H, OD-H, OJ-H. All samples tested by chiral stationary phase HPLC were dissolved in *n*-hexane/isopropanol.

Unless otherwise noted, all reactions were carried out under a nitrogen atmosphere in sealed tube with magnetic stirring. All commercial materials were used without further purification. 2-Methyltetrahydrofuran (anhydrous) was purchased from Energy Chemical. Visualization of the developed chromatogram was performed by UV light, staining with iodine (dispersed in silica gel), or by $KMnO_4$ stain. Flash column chromatography was performed over silica gel (300-400 mesh, purchased from Yantai, China). Substrates (*E*)-vinyl iodides **1**, **4**[1-3], *N*-tosylhydrazones **2**[4] were synthesized according to the literature method.

2. Optimization of Reaction Conditions

2.1 Table S1. Optimization of palladium catalyst.[a]

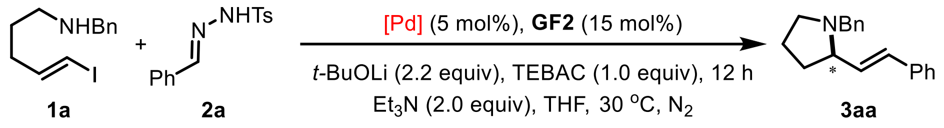

entry	[Pd]	yield (%)[b]	*er*[c]
1	$Pd_2(dba)_3$	77	91.5:8.5
2	$Pd_2(dba)_3$•$CHCl_3$	75	92:8
3	$Pd(dba)_2$	86	89.5:10.5
4	$[Pd(C_3H_5)Cl]_2$	80	91.5:8.5
5	$Pd(OAc)_2$	82	85:15
6	$PdBr_2$	78	88:12
7	PdI_2	74	88.5:11.5
8	$Pd(TFA)_2$	81	89:11
9	$Pd(acac)_2$	89	86.5:13.5

[a]Reaction conditions: **1a** (0.1 mmol), **2a** (0.16 mmol), [Pd] (5 mol%), **GF2** (15 mol%), *t*-BuOLi (2.2 equiv), TEBAC (1.0 equiv), Et_3N (2.0 equiv) in 0.1 M THF at 30 °C for 12 h. [b]Determined by GC analysis with *n*-tetradecane as an internal standard. [c]The *er* value was determined by HPLC.

2.2 Table S2. Optimization of solvent.[a]

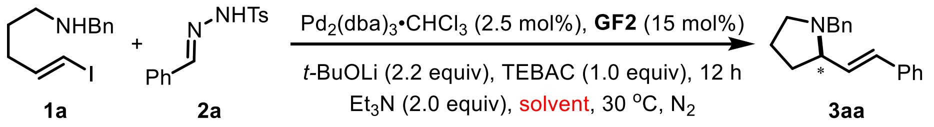

entry	solvent	yield (%)[b]	*er*[c]
1	THF	75	92:8
2	2-MeTHF	89	93:7
3	1,4-Dioxane	38	88.5:11.5
4	MTBE	28	93:7
5	DMF	90	80:20
6	MeOH	23	86.5:13.5
7	DCE	38	79.5:20.5
8	Toluene	23	92.5:7.5
9	DMSO	71	66.5:33.5
10	CH_3CN	53	80:20
11	EtOAc	67	93:7

[a]Reaction conditions: **1a** (0.1 mmol), **2a** (0.16 mmol), $Pd_2(dba)_3$•$CHCl_3$ (2.5 mol%), **GF2** (15 mol%), *t*-BuOLi (2.2 equiv), TEBAC (1.0 equiv), Et_3N (2.0 equiv) in 0.1 M solvent at 30 °C for 12 h. [b]Determined by GC analysis with *n*-tetradecane as an internal standard. [c]The *er* value was determined by HPLC. THF = Tetrahydrofuran. MTBE = methyl *tert*-butyl ether. DMF = *N,N*-Dimethylformamide. DCE = 1,2-Dichloroethane. DMSO = Dimethyl sulfoxide.

2.3 Table S3 Optimization of temperature.[a]

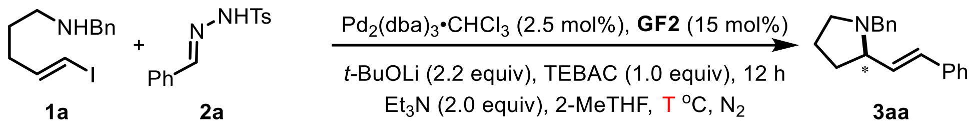

entry	T (°C)	yield (%)[b]	*er*[c]
1	15	26	94.5:5.5
2	30	89	93:7
3	45	71	90:10
4	60	63	88:12

[a]Reaction conditions: **1a** (0.1 mmol), **2a** (0.16 mmol), $Pd_2(dba)_3$•$CHCl_3$ (2.5 mol%), **GF2** (15 mol%), *t*-BuOLi (2.2 equiv), TEBAC (1.0 equiv), Et_3N (2.0 equiv) in 0.1 M 2-MeTHF for 12 h. [b]Determined by GC analysis with *n*-tetradecane as an internal standard. [c]The *er* value was determined by HPLC.

2.4 Table S4. Optimization of base.[a]

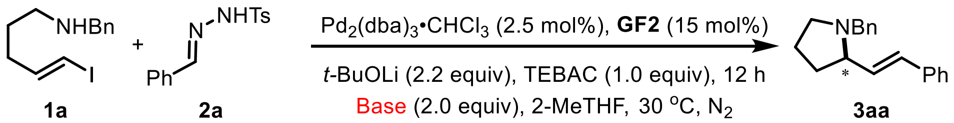

entry	Base	yield (%)[b]	*er*[c]
1	None	83	93:7
2	Et_3N	89	93:7
3	DABCO	76	94:6
4	DIPEA	86	93:7
5	PhNHMe	91	92.5:7.5
6	$PhNMe_2$	83	92.5:7.5
7	KOH	89	93:7
8	$KHCO_3$	70	93.5:6.5
9	$NaHCO_3$	83	93:7
10	Na_2CO_3	83	92:8
11	Cs_2CO_3	93	92.5:7.5
12	CsOAc	69	88:12
13[d]	None	84	91:9

[a]Reaction conditions: **1a** (0.1 mmol), **2a** (0.16 mmol), $Pd_2(dba)_3$•$CHCl_3$ (2.5 mol%), **GF2** (15 mol%), *t*-BuOLi (2.2 equiv), TEBAC (1.0 equiv), Base (2.0 equiv) in 0.1 M 2-MeTHF at 30 °C for 12 h. [b]Determined by GC analysis with *n*-tetradecane as an internal standard. [c]The *er* value was determined by HPLC. [d]Without TEBAC. DABCO = Triethylenediamine. DIPEA = *N,N*-Diisopropylethylamine.

2.5 Table S5. Optimization of additive.[a]

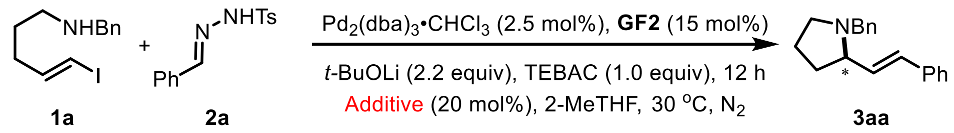

entry	Additive	yield (%)[b]	*er*[c]
1	None	83	93:7
2	AcOH	71	93:7
3	TsOH	64	93:7
4	$Sc(OTf)_3$	57	92.5:7.5
5	$Zn(OTf)_2$	56	92.5:7.5
6	AgOTf	87	93:7
7	$AgClO_4$	76	92.5:7.5
8	$AgPF_6$	78	93:7
9	$AgBF_4$	75	93:7
10	AgF	77	94:6
11	Ag_2SO_4	79	94:6
12	Ag_2CO_3	81	94.5:5.5
13[d]	Ag_2CO_3	77	94.5:5.5
14[e]	Ag_2CO_3	67	92.5:7.5

[a]Reaction conditions: **1a** (0.1 mmol), **2a** (0.16 mmol), $Pd_2(dba)_3$•$CHCl_3$ (2.5 mol%), **GF2** (15 mol%), *t*-BuOLi (2.2 equiv), TEBAC (1.0 equiv), additive (20 mol%) in 0.1 M 2-MeTHF at 30 °C for 12 h. [b]Determined by GC analysis with *n*-tetradecane as an internal standard. [c]The *er* value was determined by HPLC. [d]15 mol% Ag_2CO_3. [e]30 mol% Ag_2CO_3.

3. General Procedure for the Synthesis of Substrates 1, 4

The materials **1** and **4** were prepared according to the known synthetic route in literature. [1-3]

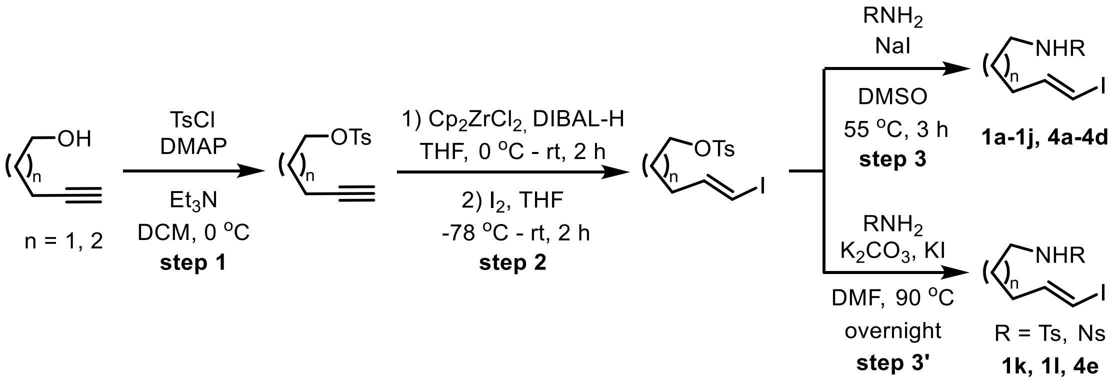

Step 1: To a solution of alkynol (1.0 equiv), Et_3N (1.5 equiv), and 4-(dimethylamino) pyridine (5 mol%) in DCM (0.5 M) at 0 °C was added *p*-toluenesulfonyl chloride (1.3 equiv) in three portions. The reaction mixture was stirred at room temperature until the starting material was no longer detectable by thin layer chromatography (2 h). The reaction mixture was added aqueous NaOH solution (1 M) and vigorously stirred for 30 min at room temperature, and then was extracted with DCM. The combined organic phases were dried over Na_2SO_4 and concentrated *in vacuo*. The residue was purified by silica gel chromatography using PE/EA = 20/1 as the eluent to afford the desired product.

Step 2: A flame-dried flask equipped with a stir bar under N_2 was charged with Cp_2ZrCl_2 (1.2 equiv) in dry THF to give a 2.5 M suspension. The reaction mixture was kept in ice bath, DIBAL-H (1.22 equiv) was added dropwise and stirred for 1 h at room temperature. Then the reaction mixture was cooled to 0 °C again and the alkyne obtained above in THF was added dropwise and stirred for 1 h at room temperature. After cooling to -78 °C, iodine (1.4 equiv) in THF (1 M) was added dropwise and the reaction mixture was stirred for 3 h at room temperature. Upon completion, the reaction was quenched with water at 0 °C and then added aqueous NaOH solution (3 M). The mixture was extracted with EtOAc and washed with $Na_2S_2O_3$. The organic layer was dried over Na_2SO_4 and concentrated *in vacuo*. The residue was purified by silica gel

chromatography using PE/EA = 20/1 as the eluent to afford the desired vinyl iodide.

Step 3: To a solution of the above obtained vinyl iodide (1.0 equiv), NaI (0.055 equiv) in DMSO (0.3 M) was added corresponding primary amine (1.5 equiv). The mixture was heated to 55 ºC until the starting material was no longer detectable by thin layer chromatography (3-6 h). The reaction mixture was cooled to room temperature and quenched with a saturated solution of NH_4Cl. Then the mixture was extracted with EtOAc and washed with water. The organic layer was dried over Na_2SO_4 and concentrated *in vacuo*. The residue was purified by silica gel chromatography using PE/EA/Et_3N as the eluent to afford the desired product **1a-1j**, **4a-4d**.

Step 3': To a solution of the above obtained vinyl iodide (1.0 equiv), K_2CO_3 (2 equiv), KI (0.1 equiv) in DMF (0.5 M) was added corresponding primary amine (1.5 equiv). The mixture was heated to 90 ºC and stirred overnight. The reaction mixture was cooled to room temperature and quenched with a saturated solution of NH_4Cl. Then the mixture was extracted with EtOAc and washed with water. The organic layer was dried over Na_2SO_4 and concentrated *in vacuo*. The residue was purified by silica gel chromatography using PE/EA/DCM as the eluent to afford the desired product **1k**, **1l**, **4e**.

Characterization of structurally novel substrates 1b-1l, 4c-4e

(*E*)-*N*-(2-bromobenzyl)-5-iodopent-4-en-1-amine (1b):

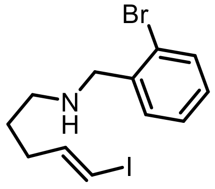

Orange oil.

^{1}H NMR (400 MHz, $CDCl_3$) δ 7.53 (d, *J* = 7.9 Hz, 1H), 7.36 (dd, *J* = 7.6, 1.5 Hz, 1H), 7.31 – 7.23 (m, 1H), 7.11 (td, *J* = 7.7, 1.6 Hz, 1H), 6.49 (dt, *J* = 14.3, 7.2 Hz, 1H), 5.99 (d, *J* = 14.4 Hz, 1H), 3.83 (s, 2H), 2.61 (t, *J* = 7.1 Hz, 2H), 2.15 – 2.07 (m, 2H), 1.64 – 1.59 (m, 2H), 1.58 (s, 1H); ^{13}C NMR (100 MHz, $CDCl_3$) δ 145.9, 139.1, 132.7, 130.2, 128.5, 127.3, 123.8, 74.9, 53.6, 48.0, 33.6, 28.5. HRMS (ESI) calculated for $C_{12}H_{16}BrIN$ [(M+H)$^+$]: 379.9505, found: 379.9505.

(*E*)-5-iodo-*N*-(2-methylbenzyl)pent-4-en-1-amine (1c):

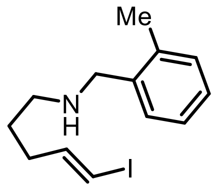

Dark orange oil.

^{1}H NMR (400 MHz, $CDCl_3$) δ 7.33 – 7.26 (m, 1H), 7.23 – 7.15 (m, 3H), 6.54 (dt, *J* = 14.3, 7.1 Hz, 1H), 6.01 (d, *J* = 14.4 Hz, 1H), 3.77 (s, 2H), 2.69 (t, *J* = 7.1 Hz, 2H), 2.37 (s, 3H), 2.20 – 2.09 (m, 2H), 1.68 – 1.58 (m, 2H), 1.38 (s, 1H); ^{13}C NMR (100 MHz, $CDCl_3$) δ 146.0, 138.2, 136.1, 130.2, 128.2, 126.9, 125.8, 74.7, 51.5, 48.7, 33.7, 28.6, 18.9. HRMS (ESI) calculated for $C_{13}H_{19}IN$ [(M+H)$^+$]: 316.0557, found: 316.0551.

(*E*)-*N*-(3-fluorobenzyl)-5-iodopent-4-en-1-amine (1d):

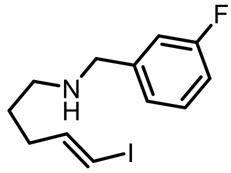

Brown oil.

^{1}H NMR (400 MHz, $CDCl_3$) δ 7.30 – 7.23 (m, 1H), 7.07 (d, *J* = 7.7 Hz, 1H), 7.05 – 7.00 (m, 1H), 6.93 (td, *J* = 8.4, 2.2 Hz, 1H), 6.50 (dt, *J* = 14.3, 7.1 Hz, 1H), 6.00 (d, *J* = 14.4 Hz, 1H), 3.76 (s, 2H), 2.61 (t, *J* = 7.1 Hz, 2H), 2.14 – 2.08 (m, 2H), 1.64 – 1.54 (m, 2H), 1.46 (s, 1H); ^{13}C NMR (100 MHz, $CDCl_3$) δ 162.9 (d, *J* = 244.2 Hz), 145.9, 143.1 (d, *J* = 7.0 Hz), 129.7 (d, *J* = 8.1 Hz), 123.5 (d, *J* = 2.8 Hz), 114.7 (d, *J* = 21.0 Hz), 113.7 (d, *J* = 21.1 Hz), 74.8, 53.3, 53.2, 48.3, 33.7, 28.6. ^{19}F NMR (377 MHz, $CDCl_3$) δ -113.5. HRMS (ESI) calculated for $C_{12}H_{16}FIN$ [(M+H)$^+$]: 320.0306, found: 320.0300.

(*E*)-5-iodo-*N*-(3-methylbenzyl)pent-4-en-1-amine (1e):

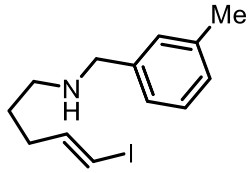

Orange oil.

^{1}H NMR (400 MHz, $CDCl_3$) δ 7.24 – 7.20 (m, 1H), 7.16 – 7.07 (m, 2H), 7.07 (d, *J* = 7.5 Hz, 1H), 6.52 (dt, *J* = 14.3, 7.2 Hz, 1H), 6.00 (dt, *J* = 14.3, 1.4 Hz, 1H), 3.74 (s, 2H), 2.63 (t, *J* = 7.1 Hz, 2H), 2.35 (s, 3H), 2.15 – 2.09 (m, 2H), 1.64 – 1.58 (m, 2H), 1.34 (s, 1H); ^{13}C NMR (100 MHz, $CDCl_3$) δ 146.0, 140.2, 138.0, 128.8, 128.2, 127.6, 125.1, 74.8, 53.9, 48.4, 33.7, 28.6, 21.4. HRMS (ESI) calculated for $C_{13}H_{19}IN$ [(M+H)$^+$]: 316.0557, found: 316.0549.

(*E*)-*N*-(4-bromobenzyl)-5-iodopent-4-en-1-amine (1f):

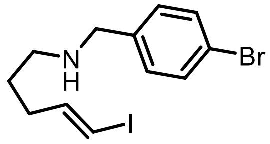

Orange oil.

^{1}H NMR (400 MHz, $CDCl_3$) δ 7.43 (d, *J* = 8.3 Hz, 2H), 7.18 (d, *J* = 8.2 Hz, 2H), 6.49 (dt, *J* = 14.3, 7.1 Hz, 1H), 5.99 (dt, *J* =

14.4, 1.4 Hz, 1H), 3.72 (s, 2H), 2.60 (t, *J* = 7.1 Hz, 2H), 2.14 – 2.07 (m, 2H), 1.64 – 1.54 (m, 2H), 1.51 (s, 1H); ^{13}C NMR (100 MHz, $CDCl_3$) δ 145.9, 139.3, 131.4, 129.7, 120.6, 74.8, 53.2, 48.3, 33.7, 28.6. HRMS (ESI) calculated for $C_{12}H_{16}BrIN$ [(M+H)$^+$]: 379.9505, found: 379.9503.

(*E*)-5-iodo-*N*-(4-methoxybenzyl)pent-4-en-1-amine (1g):

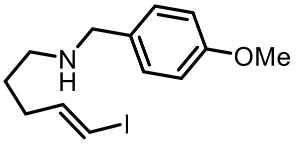

Yellow oil.

^{1}H NMR (400 MHz, $CDCl_3$) δ 7.22 (d, *J* = 7.3 Hz, 2H), 6.86 (d, *J* = 7.3 Hz, 2H), 6.50 (dt, *J* = 14.3, 7.1 Hz, 1H), 5.99 (d, *J* = 14.4 Hz, 1H), 3.80 (s, 3H), 3.71 (s, 2H), 2.61 (t, *J* = 7.1 Hz, 2H), 2.15 – 2.07 (m, 2H), 1.63 – 1.55 (m, 2H), 1.43 (s, 1H); ^{13}C NMR (100 MHz, $CDCl_3$) δ 158.5, 146.0, 132.4, 129.1, 113.7, 74.7, 55.2, 53.2, 48.2, 33.7, 28.6. HRMS (ESI) calculated for $C_{13}H_{19}INO$ [(M+H)$^+$]: 332.0506, found: 332.0506.

(*E*)-*N*-(4-(*tert*-butyl)benzyl)-5-iodopent-4-en-1-amine (1h):

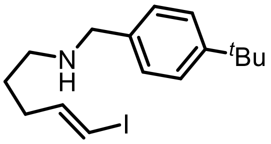

Brown oil.

^{1}H NMR (400 MHz, $CDCl_3$) δ 7.41 – 7.36 (m, 2H), 7.30 – 7.26 (m, 2H), 6.54 (dt, *J* = 14.3, 7.2 Hz, 1H), 6.02 (dt, *J* = 14.3, 1.4 Hz, 1H), 3.77 (s, 2H), 2.67 (t, *J* = 7.1 Hz, 2H), 2.17 – 2.11 (m, 2H), 1.67 – 1.61 (m, 2H), 1.58 (s, 1H), 1.35 (s, 9H). ^{13}C NMR (100 MHz, $CDCl_3$) δ 149.8, 146.0, 137.1, 127.8, 125.3, 74.8, 53.5, 48.4, 34.4, 33.7, 31.3, 28.6. HRMS (ESI) calculated for $C_{16}H_{25}IN$ [(M+H)$^+$]: 358.1026, found: 358.1021.

(*E*)-*N*-(3,5-dimethoxybenzyl)-5-iodopent-4-en-1-amine (1i):

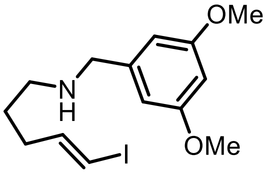

Yellow oil.

^{1}H NMR (400 MHz, $CDCl_3$) δ 6.54 – 6.47 (m, 1H), 6.46 (s, 2H), 6.34 (s, 1H), 5.98 (d, *J* = 14.3 Hz, 1H), 3.77 (s, 6H), 3.69 (s, 2H), 2.60 (t, *J* = 7.1 Hz, 2H), 2.12 – 2.05 (m, 2H), 1.71 (s, 1H), 1.61 – 1.54 (m, 2H); ^{13}C NMR (100 MHz, $CDCl_3$) δ 160.7, 145.9, 142.7, 105.8, 98.8, 74.7, 55.2, 53.9, 48.2, 33.6, 28.5. HRMS (ESI) calculated for $C_{14}H_{21}INO_2$ [(M+H)$^+$]: 362.0612, found: 362.0613.

(*E*)-5-iodo-*N*-(naphthalen-1-ylmethyl)pent-4-en-1-amine (1j):

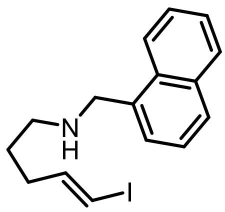

Yellow oil.

^{1}H NMR (400 MHz, $CDCl_3$) δ 8.13 (d, *J* = 8.3 Hz, 1H), 7.88 (d, *J* = 7.9 Hz, 1H), 7.78 (d, *J* = 7.7 Hz, 1H), 7.56 – 7.42 (m, 4H), 6.52 (dddt, *J* = 14.3, 8.7, 7.1, 1.5 Hz, 1H), 5.98 (dq, *J* = 14.4, 1.7 Hz, 1H), 4.23 (s, 2H), 2.78 – 2.72 (m, 2H), 2.17 – 2.10 (m, 2H), 1.68 – 1.61 (m, 2H), 1.42 (s, 1H); ^{13}C NMR (100 MHz, $CDCl_3$) δ 145.9, 135.8, 133.7, 131.6, 128.6, 127.6, 125.9, 125.8, 125.5, 125.3, 123.5, 74.8, 51.5, 48.8, 33.6, 28.5. HRMS (ESI) calculated for $C_{16}H_{19}IN$ [$(M+H)^+$]: 352.0557, found: 352.0557.

(*E*)-*N*-(5-iodopent-4-en-1-yl)-4-methylbenzenesulfonamide (1k):

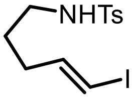

Gray solid (m.p. 62-63 °C).

^{1}H NMR (400 MHz, $CDCl_3$) δ 7.74 (d, *J* = 8.2 Hz, 2H), 7.32 (d, *J* = 8.0 Hz, 2H), 6.37 (dt, *J* = 14.4, 7.2 Hz, 1H), 5.94 (d, *J* = 14.4 Hz, 1H), 4.76 (t, *J* = 6.1 Hz, 1H), 2.97 – 2.88 (m, 2H), 2.43 (s, 3H), 2.08 – 2.01 (m, 2H), 1.59 – 1.51 (m, 2H); ^{13}C NMR (100 MHz, $CDCl_3$) δ 144.7, 143.5, 136.7, 129.7, 127.0, 75.7, 42.1, 32.7, 28.1, 21.5. HRMS (ESI) calculated for $C_{12}H_{16}INNaO_2S$ [$(M+Na)^+$]: 387.9839, found: 387.9839.

(*E*)-*N*-(5-iodopent-4-en-1-yl)-4-nitrobenzenesulfonamide (1l):

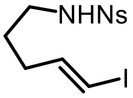

Yellow solid (m.p. 105-106 °C).

^{1}H NMR (400 MHz, $CDCl_3$) δ 8.41 – 8.33 (m, 2H), 8.09 – 8.01 (m, 2H), 6.39 (dt, *J* = 14.4, 7.2 Hz, 1H), 6.01 (dt, *J* = 14.4, 1.4 Hz, 1H), 5.04 (t, *J* = 6.1 Hz, 1H), 3.04 – 2.95 (m, 2H), 2.12 – 2.02 (m, 2H), 1.67 – 1.55 (m, 2H); ^{13}C NMR (100 MHz, $CDCl_3$) δ 150.1, 145.7, 144.3, 128.2, 124.5, 76.1, 42.3, 32.6, 28.2. HRMS (ESI) calculated for $C_{11}H_{13}IN_2NaO_4S$ [$(M+Na)^+$]: 418.9533, found: 418.9526.

(*E*)-*N*-(6-iodohex-5-en-1-yl)-3-methylaniline (4c):

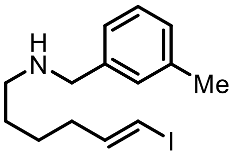

Yellow oil.

^{1}H NMR (400 MHz, $CDCl_3$) δ 7.24 – 7.19 (m, 1H), 7.14 (s, 1H), 7.10 (d, *J* = 7.5 Hz, 1H), 7.07 (d, *J* = 7.6 Hz, 1H), 6.50 (dt, *J* = 14.3, 7.1 Hz, 1H), 5.98 (dt, *J* = 14.4, 1.4 Hz, 1H), 3.74 (s, 2H), 2.62 (t, *J* = 7.0 Hz, 2H), 2.35 (s, 3H), 2.11 – 2.02 (m, 2H), 1.56 – 1.49 (m, 2H), 1.49 – 1.41 (m, 3H); ^{13}C NMR (100 MHz, $CDCl_3$) δ 146.3, 140.3, 138.0, 128.8, 128.2, 127.6, 125.1, 74.6, 54.0, 49.1, 35.8,

29.3, 26.0, 21.4. HRMS (ESI) calculated for $C_{14}H_{21}IN$ [(M+H)$^+$]: 330.0713, found: 330.0716.

(*E*)-*N*-(6-iodohex-5-en-1-yl)naphthalen-1-amine (4d):

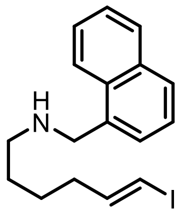

Yellow oil.

^{1}H NMR (400 MHz, $CDCl_3$) δ 8.14 (d, *J* = 8.5 Hz, 1H), 7.92 – 7.85 (m, 1H), 7.79 (d, *J* = 7.9 Hz, 1H), 7.58 – 7.50 (m, 2H), 7.50 – 7.47 (m, 1H), 7.47 – 7.42 (m, 1H), 6.51 (dt, *J* = 14.3, 7.2 Hz, 1H), 5.99 (dt, *J* = 14.3, 1.4 Hz, 1H), 4.24 (s, 2H), 2.74 (t, *J* = 7.0 Hz, 2H), 2.11 – 2.02 (m, 2H), 1.61 – 1.51 (m, 2H), 1.51 – 1.43 (m, 2H), 1.41 (s, 1H); ^{13}C NMR (100 MHz, $CDCl_3$) δ 146.3, 135.9, 133.8, 131.7, 128.6, 127.6, 126.0, 125.9, 125.5, 125.3, 123.5, 74.6, 51.6, 49.5, 35.8, 29.3, 26.0. HRMS (ESI) calculated for $C_{17}H_{21}IN$ [(M+H)$^+$]: 366.0713, found: 366.0709.

(*E*)-*N*-(6-iodohex-5-en-1-yl)-4-nitrobenzenesulfonamide (4e):

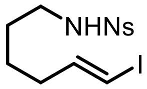

Yellow solid (m.p. 73-74 ^{o}C).

^{1}H NMR (400 MHz, $CDCl_3$) δ 8.35 – 8.30 (m, 2H), 8.07 – 8.01 (m, 2H), 6.35 (dt, *J* = 14.3, 7.1 Hz, 1H), 5.91 (dt, *J* = 14.4, 1.4 Hz, 1H), 5.39 (s, 1H), 2.96 (t, *J* = 6.9 Hz, 2H), 2.03 – 1.94 (m, 2H), 1.50 – 1.41 (m, 2H), 1.40 – 1.31 (m, 2H); ^{13}C NMR (100 MHz, $CDCl_3$) δ 149.7, 145.6, 145.4, 128.1, 124.3, 75.1, 42.8, 35.0, 28.5, 24.8. HRMS (ESI) calculated for $C_{12}H_{15}IN_2NaO_4S$ [(M+Na)$^+$]: 432.9689, found: 432.9694.

4. General Procedure for the Synthesis of Products 3, 5

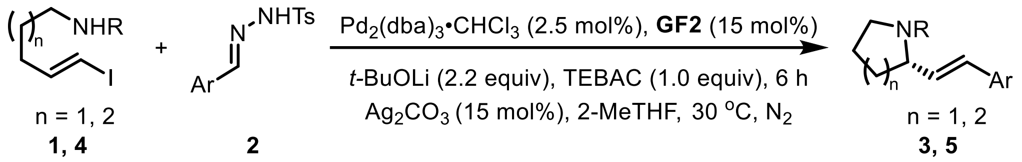

To a 10 mL sealed tube was added *N*-tosylhydrazones **2** (0.48 mmol, 1.6 equiv), $Pd_2(dba)_3·CHCl_3$ (0.0075 mmol, 2.5 mol%), **GF2** (0.045 mmol, 15 mol%), *t*-BuOLi (0.66 mmol, 2.2 equiv), Ag_2CO_3 (0.045 mmol, 15 mol%), and triethylbenzylammonium chloride (TEBAC, 0.3 mmol, 1.0 equiv) was added in the glove box. The reaction tube was evacuated and back-filled with N_2 three times and a solution of the vinyl iodide **1**, **4** (0.3 mmol, 1.0 equiv, 0.1 M in 2- MeTHF) was added under nitrogen atmosphere. Then the tube was stirred at 30 °C for 6 h. Upon completion, the reaction mixture was filtered through celite, evaporated *in vacuo* and purified by silica gel column chromatography (DCM to PE/Et_3N = 50/1) to give the desired product **3**, **5**.

***(S,E)*-1-benzyl-2-styrylpyrrolidine (3aa):**

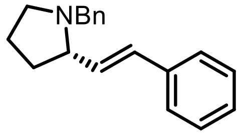

White solid (69 mg, 87% yield), 94.5:5.5 *er*. m.p. 61-62 °C; $[\alpha]_D^{20}$ = -38.2 (*c* 0.56, $CHCl_3$); ^1H NMR (400 MHz, $CDCl_3$) δ 7.42 (d, *J* = 7.2 Hz, 2H), 7.38 – 7.27 (m, 6H), 7.27 – 7.17 (m, 2H), 6.58 (d, *J* = 15.8 Hz, 1H), 6.21 (dd, *J* = 15.8, 8.3 Hz, 1H), 4.09 (d, *J* = 12.9 Hz, 1H), 3.16 (d, *J* = 12.9 Hz, 1H), 3.01 (t, *J* = 8.2 Hz, 2H), 2.25 – 2.13 (m, 1H), 2.10 – 1.97 (m, 1H), 1.92 – 1.71 (m, 3H); ^{13}C NMR (100 MHz, $CDCl_3$) δ 139.5, 137.2, 132.7, 131.6, 129.0, 128.5, 128.1, 127.3, 126.7, 126.3, 67.9, 58.3, 53.4, 31.8, 22.2. HRMS (ESI) calculated for $C_{19}H_{22}N$ $[(M+H)^+]$: 264.1747, found: 264.1749. HPLC (Daicel Chiralpak OD-H column, *n*-hexane/isopropanol = 90/10, flow rate 1.0 mL/min, 254 nm): t_R (minor) = 3.7 min, t_R (major) = 3.9 min.

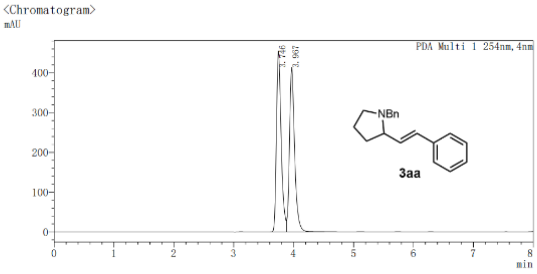

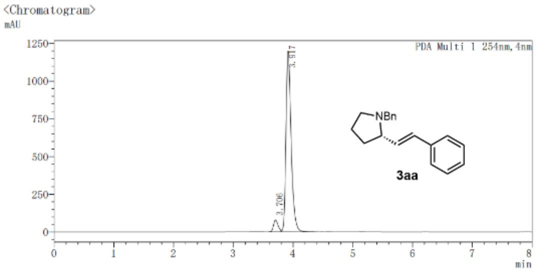

<Peak Table>
PDA Ch1 254nm

No.	Ret. Time (min)	Height (uAU)	Height%	Area (uAU*min)	Area%
1	3.746	455680	52.424	2512284	50.585
2	3.967	413532	47.576	2454196	49.415
Total		869212	100.000	4966479	100.000

<Peak Table>
PDA Ch1 254nm

No.	Ret. Time (min)	Height (uAU)	Height%	Area (uAU*min)	Area%
1	3.706	78858	6.167	421061	5.514
2	3.917	1199937	93.833	7215378	94.486
Total		1278795	100.000	7636439	100.000

***(S,E)*-1-benzyl-2-(4-bromostyryl)pyrrolidine (3ab):**

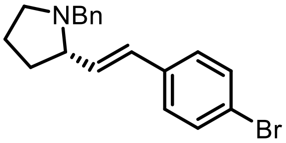

Yellow solid (90.2 mg, 88% yield), 93.5:6.5 *er*. m.p. 51-52 °C; $[\alpha]_D^{20}$ = +8.2 (*c* 0.6, $CHCl_3$); ^{1}H NMR (400 MHz, $CDCl_3$) δ 7.47 – 7.40 (m, 2H), 7.35 – 7.28 (m, 4H), 7.27 – 7.26 (m, 1H), 7.26 – 7.20 (m, 2H), 6.50 (d, *J* = 15.9 Hz, 1H), 6.19 (dd, *J* = 15.8, 8.3 Hz, 1H), 4.04 (d, *J* = 13.0 Hz, 1H), 3.18 (d, *J* = 13.0 Hz, 1H), 3.06 – 2.93 (m, 2H), 2.25 – 2.14 (m, 1H), 2.06 – 2.00 (m, 1H), 1.89 – 1.70 (m, 3H); ^{13}C NMR (100 MHz, $CDCl_3$) δ 139.3, 136.1, 133.6, 131.6, 130.3, 128.9, 128.1, 127.8, 126.8, 121.0, 67.7, 58.4, 53.4, 31.8, 22.2. HRMS (ESI) calculated for $C_{19}H_{21}BrN$ $[(M+H)^+]$: 342.0852, found: 342.0851. HPLC (Daicel Chiralpak AD-H column, *n*-hexane/isopropanol = 90/10, flow rate 1.0 mL/min, 254 nm): t_R (major) = 4.0 min, t_R (minor) = 4.6 min.

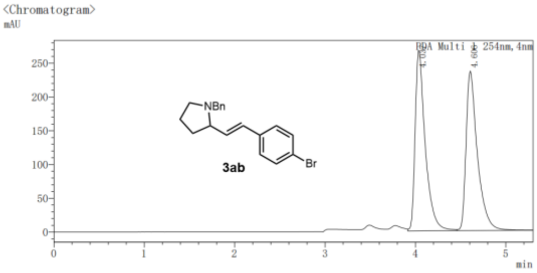

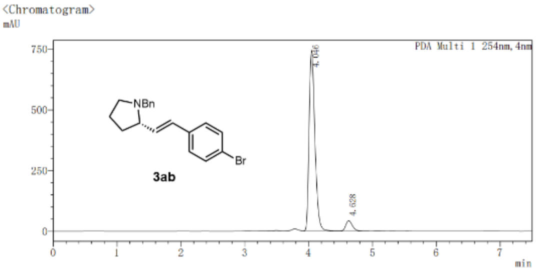

<Peak Table>
PDA Ch1 254nm

No.	Ret. Time (min)	Height (uAU)	Height%	Area (uAU*min)	Area%
1	4.037	267013	53.087	2081272	50.252
2	4.606	235961	46.913	2060357	49.748
Total		502974	100.000	4141629	100.000

<Peak Table>
PDA Ch1 254nm

No.	Ret. Time (min)	Height (uAU)	Height%	Area (uAU*min)	Area%
1	4.046	745548	94.477	4738808	93.412
2	4.628	43583	5.523	334185	6.588
Total		789131	100.000	5072993	100.000

***(S,E)*-1-benzyl-2-(4-(*tert*-butyl)styryl)pyrrolidine (3ac):**

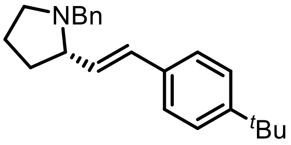

White solid (77.1 mg, 81% yield), 94:6 *er*. m.p. 77-78 °C; $[\alpha]_D^{20}$ = -17.9 (*c* 0.62, $CHCl_3$); ^{1}H NMR (400 MHz, $CDCl_3$) δ 7.44 – 7.37 (m, 4H), 7.36 – 7.28 (m, 4H), 7.28 – 7.22 (m, 1H), 6.59 (d, *J* = 15.8 Hz, 1H), 6.21 (dd, *J* = 15.8, 8.4 Hz, 1H), 4.11 (d, *J* = 12.9 Hz, 1H),

3.16 (d, J = 12.9 Hz, 1H), 3.07 – 2.94 (m, 2H), 2.26 – 2.15 (m, 1H), 2.09 – 2.02 (m, 1H), 1.92 – 1.69 (m, 3H), 1.36 (s, 9H); ^{13}C NMR (100 MHz, $CDCl_3$) δ 150.4, 139.4, 134.3, 131.8, 131.5, 129.0, 128.1, 126.7, 126.0, 125.4, 67.9, 58.2, 53.3, 34.5, 31.8, 31.3, 22.1. HRMS (ESI) calculated for $C_{23}H_{30}N$ [(M+H)$^+$]: 320.2373, found: 320.2366. HPLC (Daicel Chiralpak AD-H column, *n*-hexane/isopropanol = 97/3, flow rate 0.5 mL/min, 254 nm): t_R (major) = 7.2 min, t_R (minor) = 7.9 min.

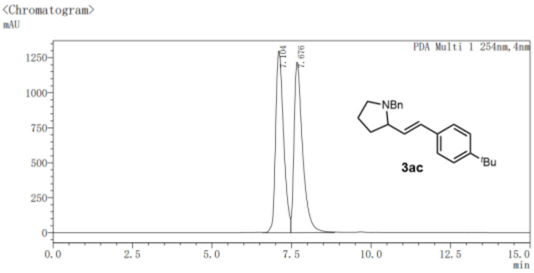

<Peak Table>
PDA Ch1 254nm

No.	Ret.Time(min)	Height(uAU)	Height%	Area(uAU*min)	Area%
1	7.104	1298677	51.581	22671086	49.808
2	7.676	1219050	48.419	22845847	50.192
Total		2517727	100.000	45516933	100.000

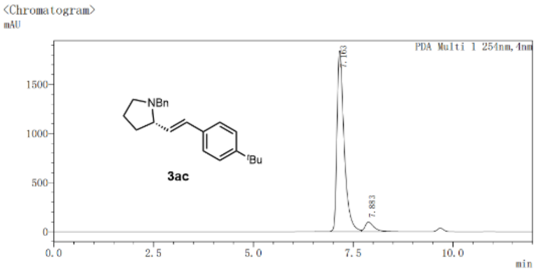

<Peak Table>
PDA Ch1 254nm

No.	Ret.Time(min)	Height(uAU)	Height%	Area(uAU*min)	Area%
1	7.163	1840283	94.997	22968920	93.957
2	7.883	96913	5.003	1477199	6.043
Total		1937196	100.000	24446119	100.000

***(S,E)*-1-benzyl-2-(4-(trifluoromethoxy)styryl)pyrrolidine (3ad):**

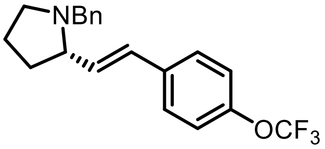

Yellow solid (89.5 mg, 86% yield), 94:6 *er*. m.p. 31-32 °C; $[\alpha]_D^{20}$ = -7.7 (c 0.73, $CHCl_3$); ^{1}H NMR (400 MHz, $CDCl_3$) δ 7.44 – 7.38 (m, 2H), 7.37 – 7.28 (m, 4H), 7.26 – 7.21 (m, 1H), 7.18 (d, J = 8.2 Hz, 2H), 6.55 (d, J = 15.9 Hz, 1H), 6.19 (dd, J = 15.8, 8.3 Hz, 1H), 4.06 (d, J = 13.0 Hz, 1H), 3.19 (d, J = 13.0 Hz, 1H), 3.07 – 2.94 (m, 2H), 2.26 – 2.16 (m, 1H), 2.08 – 2.01 (m, 1H), 1.92 – 1.71 (m, 3H); ^{13}C NMR (100 MHz, $CDCl_3$) δ 148.3, 139.4, 136.0, 133.9, 130.1, 129.0, 128.2, 127.5, 126.8, 121.1, 120.5 (d, J = 255.2 Hz), 67.7, 58.4, 53.5, 31.9, 22.3; ^{19}F NMR (377 MHz, $CDCl_3$) δ -57.8. HRMS (ESI) calculated for $C_{20}H_{21}F_3NO$ [(M+H)$^+$]: 348.1570, found: 348.1568. HPLC (Daicel Chiralpak AD-H column, *n*-hexane/isopropanol = 90/10, flow rate 1.0 mL/min, 254 nm): t_R (major) = 3.5 min, t_R (minor) = 3.8 min.

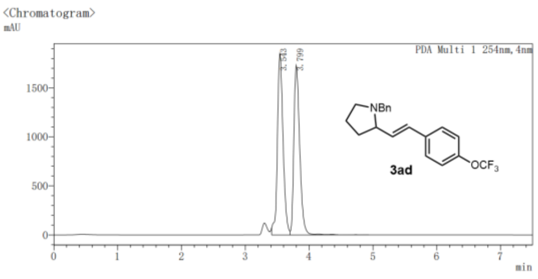

<Peak Table>
PDA Ch1 254nm

No.	Ret. Time (min)	Height (uAU)	Height%	Area (uAU*min)	Area%
1	3.543	1846943	51.635	11401707	50.684
2	3.799	1729988	48.365	11094102	49.316
Total		3576931	100.000	22495809	100.000

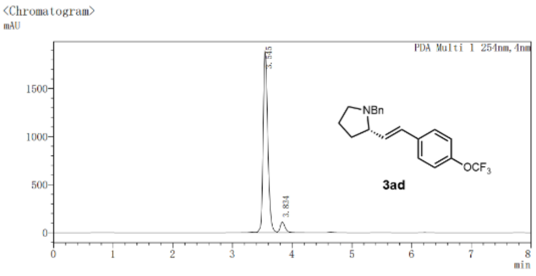

<Peak Table>
PDA Ch1 254nm

No.	Ret. Time (min)	Height (uAU)	Height%	Area (uAU*min)	Area%
1	3.545	1881584	94.508	10224428	94.185
2	3.834	109339	5.492	631312	5.815
Total		1990923	100.000	10855740	100.000

(*S,E*)-1-benzyl-2-(4-phenoxystyryl)pyrrolidine (3ae):

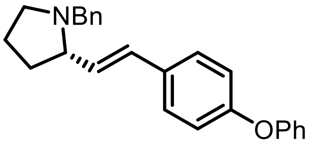

Yellow solid (79.7 mg, 75% yield), 94:6 *er*. m.p. 64-65 °C; $[\alpha]_D^{20}$ = +12.5 (*c* 0.59, $CHCl_3$); ^{1}H NMR (400 MHz, $CDCl_3$) δ 7.40 (d, *J* = 8.6 Hz, 2H), 7.38 – 7.35 (m, 2H), 7.35 – 7.30 (m, 4H), 7.27 – 7.23 (m, 1H), 7.16 – 7.10 (m, 1H), 7.04 (d, *J* = 7.8 Hz, 2H), 7.00 (d, *J* = 8.6 Hz, 2H), 6.57 (d, *J* = 15.8 Hz, 1H), 6.14 (dd, *J* = 15.8, 8.3 Hz, 1H), 4.11 (d, *J* = 13.0 Hz, 1H), 3.17 (d, *J* = 13.0 Hz, 1H), 3.07 – 2.95 (m, 2H), 2.26 – 2.15 (m, 1H), 2.08 – 2.01 (m, 1H), 1.90 – 1.72 (m, 3H); ^{13}C NMR (100 MHz, $CDCl_3$) δ 157.2, 156.5, 139.4, 132.4, 131.7, 130.8, 129.7, 129.0, 128.1, 127.6, 126.7, 123.2, 119.0, 118.8, 67.9, 58.3, 53.3, 31.8, 22.1. HRMS (ESI) calculated for $C_{25}H_{26}NO$ [$(M+H)^+$]: 356.2009, found: 356.2007. HPLC (Daicel Chiralpak AD-H column, *n*-hexane/isopropanol = 90/10, flow rate 1.0 mL/min, 254 nm): t_R (major) = 4.5 min, t_R (minor) = 5.7 min.

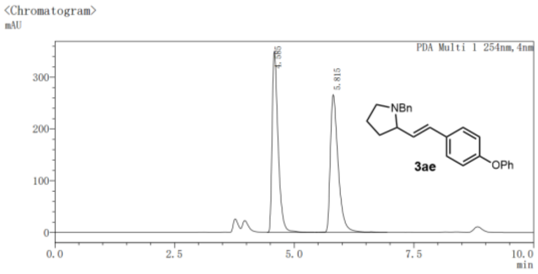

<Peak Table>
PDA Ch1 254nm

No.	Ret. Time (min)	Height (uAU)	Height%	Area (uAU*min)	Area%
1	4.585	349557	56.780	3042456	49.938
2	5.815	266081	43.220	3049992	50.062
Total		615638	100.000	6092448	100.000

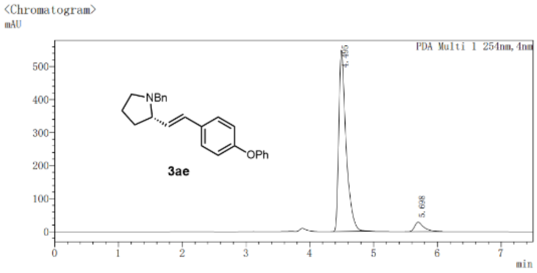

<Peak Table>
PDA Ch1 254nm

No.	Ret. Time (min)	Height (uAU)	Height%	Area (uAU*min)	Area%
1	4.495	547904	95.079	4382958	93.862
2	5.698	28356	4.921	286602	6.138
Total		576260	100.000	4669560	100.000

(*S,E*)-1-benzyl-2-(4-(methylthio)styryl)pyrrolidine (3af):

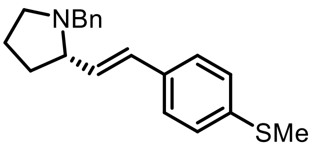

Yellow solid (65.1 mg, 70% yield), 94.5:5.5 *er*. m.p. 49-50 °C; $[\alpha]_D^{20}$ = -26.0 (*c* 0.58, $CHCl_3$); ^{1}H NMR (400 MHz, $CDCl_3$) δ 7.37 – 7.28 (m, 6H), 7.26 – 7.20 (m, 3H), 6.53 (d,

J = 15.8 Hz, 1H), 6.17 (dd, *J* = 15.8, 8.3 Hz, 1H), 4.08 (d, *J* = 13.0 Hz, 1H), 3.16 (d, *J* = 13.0 Hz, 1H), 3.05 – 2.95 (m, 2H), 2.50 (s, 3H), 2.24 – 2.14 (m, 1H), 2.07 – 2.01 (m, 1H), 1.89 – 1.71 (m, 3H); ^{13}C NMR (100 MHz, $CDCl_3$) δ 139.4, 137.3, 134.2, 132.1, 130.9, 128.9, 128.1, 126.8, 126.7, 126.6, 67.8, 58.3, 53.3, 31.8, 22.1, 15.9. HRMS (ESI) calculated for $C_{20}H_{24}NS$ [$(M+H)^+$]: 310.1624, found: 310.1619. HPLC (Daicel Chiralpak AD-H column, *n*-hexane/isopropanol = 90/10, flow rate 1.0 mL/min, 254 nm): t_R (major) = 4.6 min, t_R (minor) = 6.3 min.

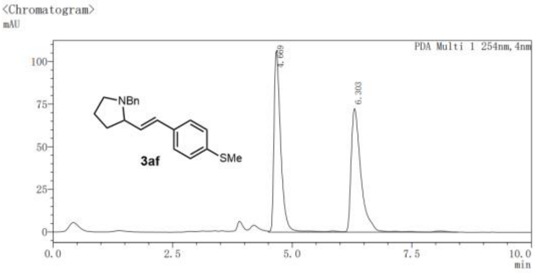

<Peak Table>
PDA Ch1 254nm

No.	Ret.Time(min)	Height(uAU)	Height%	Area(uAU*min)	Area%
1	4.669	106498	59.518	1052673	50.238
2	6.303	72437	40.482	1042696	49.762
Total		178934	100.000	2095369	100.000

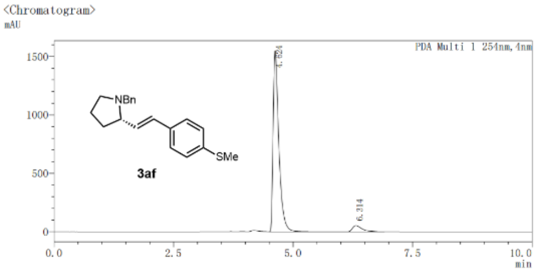

<Peak Table>
PDA Ch1 254nm

No.	Ret.Time(min)	Height(uAU)	Height%	Area(uAU*min)	Area%
1	4.624	1547743	96.699	12917693	94.609
2	6.314	52836	3.301	736108	5.391
Total		1600579	100.000	13653801	100.000

***(S,E)*-2-(2-([1,1'-biphenyl]-4-yl)vinyl)-1-benzylpyrrolidine (3ag):**

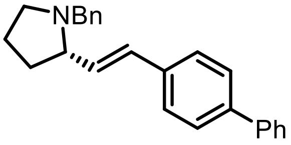

Yellow solid (68.5 mg, 67% yield), 94.5:5.5 *er*. m.p. 99-100 °C; $[\alpha]_D^{20}$ = -13.0 (*c* 0.71, $CHCl_3$); ^{1}H NMR (400 MHz, $CDCl_3$) δ 7.67 – 7.61 (m, 2H), 7.59 (d, *J* = 8.3 Hz, 2H), 7.50 (d, *J* = 8.3 Hz, 2H), 7.48 – 7.43 (m, 2H), 7.39 – 7.30 (m, 5H), 7.28 – 7.22 (m, 1H), 6.63 (d, *J* = 15.8 Hz, 1H), 6.27 (dd, *J* = 15.8, 8.3 Hz, 1H), 4.11 (d, *J* = 13.0 Hz, 1H), 3.19 (d, *J* = 13.0 Hz, 1H), 3.11 – 2.97 (m, 2H), 2.28 – 2.17 (m, 1H), 2.09 – 2.04 (m, 1H), 1.90 – 1.73 (m, 3H); ^{13}C NMR (100 MHz, $CDCl_3$) δ 140.7, 140.1, 139.4, 136.2, 132.8, 131.2, 129.0, 128.7, 128.1, 127.2, 126.9, 126.8, 126.7, 67.9, 58.3, 53.4, 31.8, 22.2. HRMS (ESI) calculated for $C_{25}H_{26}N$ [$(M+H)^+$]: 340.2060, found: 340.2051. HPLC (Daicel Chiralpak AD-H column, *n*-hexane/isopropanol = 90/10, flow rate 1.0 mL/min, 254 nm): t_R (major) = 4.7 min, t_R (minor) = 6.7 min.

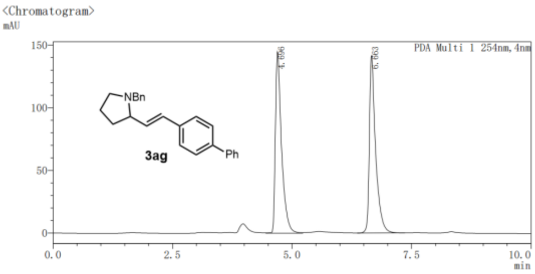

<Peak Table>
PDA Ch1 254nm

No.	Ret. Time (min)	Height (uAU)	Height%	Area (uAU*min)	Area%
1	4.696	144720	50.534	1303977	50.194
2	6.663	141663	49.466	1293888	49.806
Total		286383	100.000	2597866	100.000

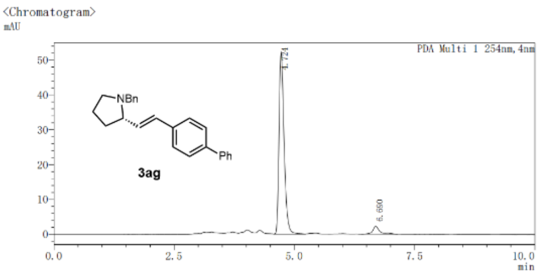

<Peak Table>
PDA Ch1 254nm

No.	Ret. Time (min)	Height (uAU)	Height%	Area (uAU*min)	Area%
1	4.724	52013	95.824	381646	94.440
2	6.690	2267	4.176	22470	5.560
Total		54280	100.000	404115	100.000

(*S,E*)-1-benzyl-2-(2-ethoxystyryl)pyrrolidine (3ah):

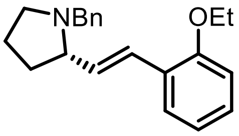

Yellow oil (81.5 mg, 88% yield), 93:7 *er*. $[\alpha]_D^{20}$ = +15.3 (*c* 0.66, $CHCl_3$); ^{1}H NMR (400 MHz, $CDCl_3$) δ 7.53 (d, *J* = 7.6 Hz, 1H), 7.37 (d, *J* = 7.5 Hz, 2H), 7.35 – 7.30 (m, 2H), 7.28 – 7.19 (m, 2H), 6.96 (dd, *J* = 16.3, 9.3 Hz, 2H), 6.88 (d, *J* = 8.2 Hz, 1H), 6.25 (dd, *J* = 16.0, 8.3 Hz, 1H), 4.14 (d, *J* = 13.2 Hz, 1H), 4.09 (dd, *J* = 14.1, 7.1 Hz, 2H), 3.20 (d, *J* = 13.0 Hz, 1H), 3.10 – 2.96 (m, 2H), 2.25 – 2.17 (m, 1H), 2.11 – 2.03 (m, 1H), 1.91 – 1.73 (m, 3H), 1.49 (t, *J* = 7.0 Hz, 3H); ^{13}C NMR (100 MHz, $CDCl_3$) δ 155.9, 139.5, 132.7, 129.1 (overlap), 128.2, 128.0, 126.6, 126.4, 126.2, 120.5, 112.0, 68.1, 63.8, 58.1, 53.2, 31.8, 22.1, 14.9. HRMS (ESI) calculated for $C_{21}H_{26}NO$ $[(M+H)^+]$: 308.2009, found: 308.2006. HPLC (Daicel Chiralpak OD-H column, *n*-hexane/isopropanol = 90/10, flow rate 1.0 mL/min, 254 nm): t_R (major) = 3.9 min, t_R (minor) = 4.1 min.

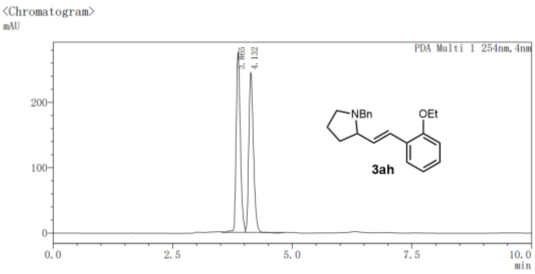

<Peak Table>
PDA Ch1 254nm

No.	Ret. Time (min)	Height (uAU)	Height%	Area (uAU*min)	Area%
1	3.865	275630	52.970	1682792	50.564
2	4.132	244719	47.030	1645249	49.436
Total		520349	100.000	3328041	100.000

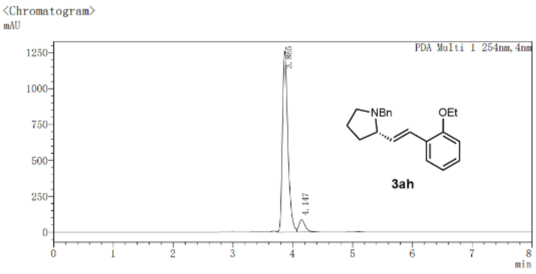

<Peak Table>
PDA Ch1 254nm

No.	Ret. Time (min)	Height (uAU)	Height%	Area (uAU*min)	Area%
1	3.865	1257508	93.592	7973247	92.897
2	4.147	86099	6.408	609628	7.103
Total		1343608	100.000	8582875	100.000

(*S,E*)-1-benzyl-2-(2-(benzyloxy)styryl)pyrrolidine (3ai):

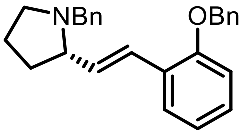

Yellow oil (72.8 mg, 66% yield), 92.5:7.5 *er*. $[\alpha]_D^{20}$ = +66.3 (*c* 0.61, $CHCl_3$); ^{1}H NMR (400 MHz, $CDCl_3$) δ 7.57 (dd, *J* = 7.6, 1.5 Hz, 1H), 7.49 (d, *J* = 7.3 Hz, 2H), 7.44 – 7.39 (m, 2H), 7.39 – 7.33

(m, 3H), 7.33 – 7.28 (m, 2H), 7.27 – 7.20 (m, 2H), 7.03 (d, *J* = 16.1 Hz, 1H), 7.01 – 6.93 (m, 2H), 6.25 (dd, *J* = 16.0, 8.4 Hz, 1H), 5.15 (s, 2H), 4.13 (d, *J* = 13.0 Hz, 1H), 3.20 (d, *J* = 13.0 Hz, 1H), 3.10 – 2.97 (m, 2H), 2.26 – 2.16 (m, 1H), 2.08 – 2.02 (m, 1H), 1.90 – 1.72 (m, 3H); ^{13}C NMR (100 MHz, $CDCl_3$) δ 155.6, 139.4, 137.2, 133.1, 129.1, 128.5, 128.3, 128.0, 127.8 (overlap), 127.2, 126.7, 126.6, 126.3, 121.0, 112.5, 70.3, 68.0, 58.1, 53.2, 31.8, 22.1. HRMS (ESI) calculated for $C_{26}H_{28}NO$ [(M+H)$^+$]: 370.2165, found: 370.2164. HPLC (Daicel Chiralpak OD-H column, *n*-hexane/isopropanol = 90/10, flow rate 1.0 mL/min, 254 nm): t_R (major) = 5.2 min, t_R (minor) = 6.0 min.

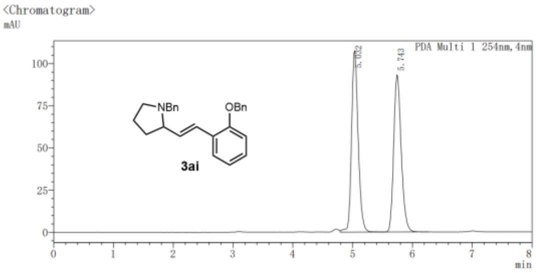

<Peak Table>

PDA Ch1 254nm

No.	Ret.Time(min)	Height(uAU)	Height%	Area(uAU*min)	Area%
1	5.032	107327	53.587	825169	50.335
2	5.743	92960	46.413	814183	49.665
Total		200288	100.000	1639351	100.000

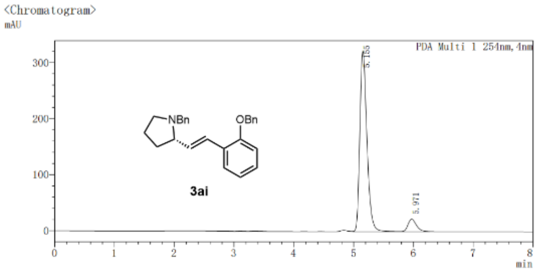

<Peak Table>

PDA Ch1 254nm

No.	Ret.Time(min)	Height(uAU)	Height%	Area(uAU*min)	Area%
1	5.155	322504	93.518	2649418	92.340
2	5.971	22355	6.482	219787	7.660
Total		344859	100.000	2869205	100.000

***(S,E)*-2-(2-([1,1'-biphenyl]-2-yl)vinyl)-1-benzylpyrrolidine (3aj):**

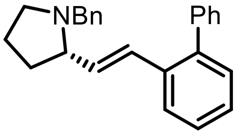

Yellow oil (82.7 mg, 81% yield), 96:4 *er*. $[\alpha]_D^{20}$ = +118.0 (*c* 0.52, $CHCl_3$); ^{1}H NMR (400 MHz, $CDCl_3$) δ 7.67 (d, *J* = 7.3 Hz, 1H), 7.46 – 7.36 (m, 6H), 7.35 – 7.28 (m, 6H), 7.27 – 7.23 (m, 1H), 6.59 (d, *J* = 15.8 Hz, 1H), 6.16 (dd, *J* = 15.8, 8.3 Hz, 1H), 4.05 (d, *J* = 13.0 Hz, 1H), 3.17 (d, *J* = 13.0 Hz, 1H), 2.98 (t, *J* = 7.7 Hz, 1H), 2.94 – 2.84 (m, 1H), 2.19 – 2.12 (m, 1H), 2.05 – 1.93 (m, 1H), 1.86 – 1.69 (m, 3H); ^{13}C NMR (100 MHz, $CDCl_3$) δ 141.0, 140.6, 139.2, 135.3, 133.5, 130.6, 130.1, 129.8, 129.0, 128.1, 128.0, 127.4, 127.2, 126.9, 126.7, 126.2, 67.6, 58.0, 53.1, 31.7, 22.1. HRMS (ESI) calculated for $C_{25}H_{26}N$ [(M+H)$^+$]: 340.2060, found: 340.2064. HPLC (Daicel Chiralpak OD-H+OD-H column, *n*-hexane/isopropanol = 99/1, flow rate 0.5 mL/min, 254 nm): t_R (minor) = 21.3 min, t_R (major) = 23.2 min.

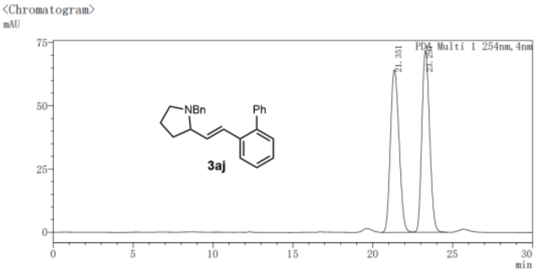

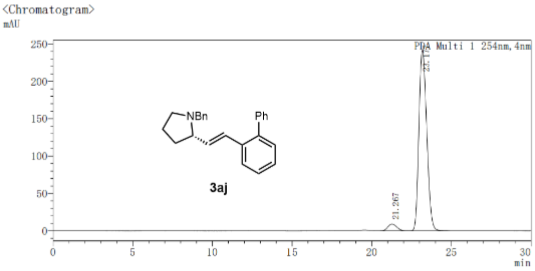

<Peak Table>

PDA Ch1 254nm

No.	Ret. Time (min)	Height (uAU)	Height%	Area (uAU*min)	Area%
1	21.351	64278	47.228	2424061	50.015
2	23.297	71823	52.772	2422605	49.985
Total		136101	100.000	4846666	100.000

<Peak Table>

PDA Ch1 254nm

No.	Ret. Time (min)	Height (uAU)	Height%	Area (uAU*min)	Area%
1	21.267	9041	3.608	349110	4.044
2	23.177	241535	96.392	8283020	95.956
Total		250577	100.000	8632130	100.000

***(S,E)*-1-benzyl-2-(3-bromostyryl)pyrrolidine (3ak):**

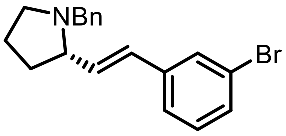

Yellow oil (76.6 mg, 75% yield), 92:8 *er*. $[\alpha]_D^{20}$ = +50.2 (*c* 0.55, $CHCl_3$); ^1H NMR (400 MHz, $CDCl_3$) δ 7.55 – 7.51 (m, 1H), 7.38 – 7.33 (m, 1H), 7.32 – 7.30 (m, 4H), 7.30 – 7.28 (m, 1H), 7.26 – 7.20 (m, 1H), 7.21 – 7.14 (m, 1H), 6.48 (d, *J* = 15.8 Hz, 1H), 6.20 (dd, *J* = 15.8, 8.2 Hz, 1H), 4.03 (d, *J* = 13.0 Hz, 1H), 3.18 (d, *J* = 13.0 Hz, 1H), 3.05 – 2.95 (m, 2H), 2.24 – 2.13 (m, 1H), 2.08 – 1.98 (m, 1H), 1.86 – 1.72 (m, 3H); ^{13}C NMR (100 MHz, $CDCl_3$) δ 139.3, 139.2, 134.4, 130.1, 130.0, 129.9, 129.2, 128.9, 128.1, 126.8, 124.9, 122.7, 67.6, 58.3, 53.4, 31.8, 22.2. HRMS (ESI) calculated for $C_{19}H_{21}BrN$ $[(M+H)^+]$: 342.0852, found: 342.0844. HPLC (Daicel Chiralpak AD-H column, *n*-hexane/isopropanol = 90/10, flow rate 1.0 mL/min, 254 nm): t_R (major) = 3.7 min, t_R (minor) = 4.2 min.

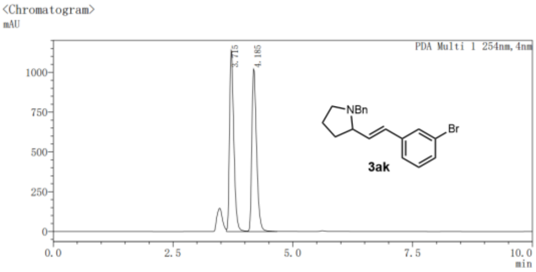

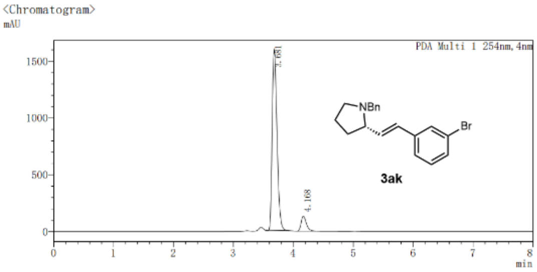

<Peak Table>

PDA Ch1 254nm

No.	Ret. Time (min)	Height (uAU)	Height%	Area (uAU*min)	Area%
1	3.715	1137150	52.720	7067219	50.133
2	4.185	1019802	47.280	7029677	49.867
Total		2156952	100.000	14096896	100.000

<Peak Table>

PDA Ch1 254nm

No.	Ret. Time (min)	Height (uAU)	Height%	Area (uAU*min)	Area%
1	3.681	1583826	92.347	8891809	91.821
2	4.168	131261	7.653	792033	8.179
Total		1715086	100.000	9683842	100.000

***(S,E)*-1-benzyl-2-(3-methoxystyryl)pyrrolidine (3al):**

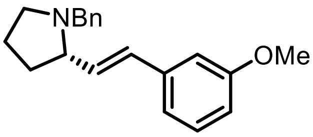

Yellow oil (78.6 mg, 89% yield), 93.5:6.5 *er*. $[\alpha]_D^{20}$ = -21.3 (*c* 0.67, $CHCl_3$); ^1H NMR (400 MHz, $CDCl_3$) δ 7.35 – 7.27 (m, 4H), 7.26 – 7.19 (m, 2H), 7.00 (d, *J* = 7.7 Hz, 1H), 6.97

– 6.94 (m, 1H), 6.80 (dd, J = 8.1, 2.3 Hz, 1H), 6.54 (d, J = 15.8 Hz, 1H), 6.21 (dd, J = 15.8, 8.3 Hz, 1H), 4.08 (d, J = 13.0 Hz, 1H), 3.82 (s, 3H), 3.16 (d, J = 13.0 Hz, 1H), 3.05 – 2.95 (m, 2H), 2.23 – 2.14 (m, 1H), 2.07 – 2.00 (m, 1H), 1.89 – 1.70 (m, 3H); ^{13}C NMR (100 MHz, $CDCl_3$) δ 159.8, 139.3, 138.6, 132.9, 131.5, 129.5, 129.0, 128.1, 126.7, 119.0, 113.1, 111.4, 67.8, 58.3, 55.2, 53.3, 31.8, 22.2. HRMS (ESI) calculated for $C_{20}H_{24}NO$ [(M+H)$^+$]: 294.1852, found: 294.1853. HPLC (Daicel Chiralpak OD-H column, *n*-hexane/isopropanol = 90/10, flow rate 1.0 mL/min, 254 nm): t_R (minor) = 4.2 min, t_R (major) = 5.1 min.

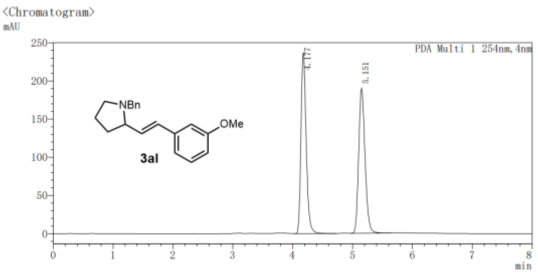

<Peak Table>
PDA Ch1 254nm

No.	Ret.Time(min)	Height(uAU)	Height%	Area(uAU*min)	Area%
1	4.177	236718	55.499	1429479	50.485
2	5.151	189806	44.501	1401993	49.515
Total		426524	100.000	2831473	100.000

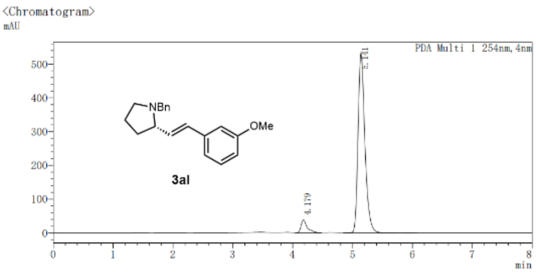

<Peak Table>
PDA Ch1 254nm

No.	Ret.Time(min)	Height(uAU)	Height%	Area(uAU*min)	Area%
1	4.179	38838	6.775	288566	6.358
2	5.141	534430	93.225	4249787	93.642
Total		573269	100.000	4538353	100.000

***(S,E)*-1-benzyl-2-(3,5-dimethoxystyryl)pyrrolidine (3am):**

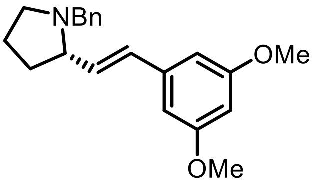

Yellow oil (77.1 mg, 80% yield), 94:6 *er*. $[\alpha]_D^{20}$ = -41.9 (*c* 0.93, $CHCl_3$); ^{1}H NMR (400 MHz, $CDCl_3$) δ 7.38 – 7.28 (m, 4H), 7.26 – 7.21 (m, 1H), 6.59 (s, 2H), 6.51 (d, J = 15.8 Hz, 1H), 6.39 (s, 1H), 6.21 (dd, J = 15.7, 8.3 Hz, 1H), 4.08 (d, J = 13.0 Hz, 1H), 3.82 (s, 6H), 3.18 (d, J = 13.0 Hz, 1H), 3.06 – 2.94 (m, 2H), 2.23 – 2.15 (m, 1H), 2.09 – 2.00 (m, 1H), 1.89 – 1.70 (m, 3H); ^{13}C NMR (100 MHz, $CDCl_3$) δ 160.9, 139.2, 139.1, 133.1, 131.6, 129.0, 128.1, 126.7, 104.3, 100.0, 67.7, 58.3, 55.6, 53.3, 31.8, 22.1. HRMS (ESI) calculated for $C_{21}H_{26}NO_2$ [(M+H)$^+$]: 324.1958, found: 324.1950. HPLC (Daicel Chiralpak AD-H column, *n*-hexane/isopropanol = 90/10, flow rate 1.0 mL/min, 254 nm): t_R (major) = 6.0 min, t_R (minor) = 6.9 min.

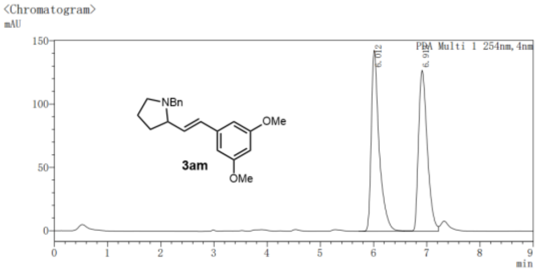

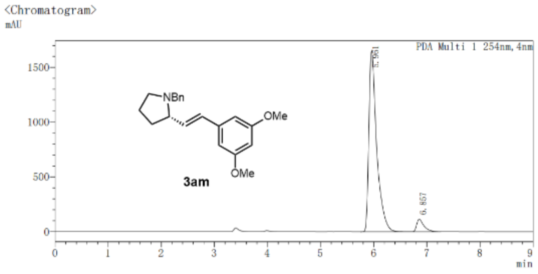

<Peak Table>
PDA Ch1 254nm

No.	Ret. Time (min)	Height (uAU)	Height%	Area (uAU*min)	Area%
1	6.012	142925	52.957	1481421	50.338
2	6.913	126965	47.043	1461552	49.662
Total		269890	100.000	2942973	100.000

<Peak Table>
PDA Ch1 254nm

No.	Ret. Time (min)	Height (uAU)	Height%	Area (uAU*min)	Area%
1	5.961	1650904	93.501	16957988	93.943
2	6.857	114755	6.499	1093401	6.057
Total		1765659	100.000	18051389	100.000

***(S,E)*-1-benzyl-2-(2,5-dimethoxystyryl)pyrrolidine (3an):**

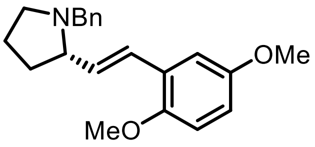

Yellow oil (73.2 mg, 75% yield), 92.5:7.5 *er*. $[\alpha]_D^{20}$ = +20.6 (*c* 0.51, $CHCl_3$); ^1H NMR (400 MHz, $CDCl_3$) δ 7.37 – 7.27 (m, 4H), 7.26 – 7.20 (m, 1H), 7.08 – 7.05 (m, 1H), 6.90 (d, *J* = 16.0 Hz, 1H), 6.84 – 6.75 (m, 2H), 6.20 (dd, *J* = 16.0, 8.4 Hz, 1H), 4.09 (d, *J* = 13.0 Hz, 1H), 3.82 (s, 3H), 3.80 (s, 3H), 3.16 (d, *J* = 13.0 Hz, 1H), 3.07 – 2.95 (m, 2H), 2.24 – 2.15 (m, 1H), 2.09 – 2.00 (m, 1H), 1.87 – 1.71 (m, 3H); ^{13}C NMR (100 MHz, $CDCl_3$) δ 153.7, 151.0, 139.5, 133.3, 129.0, 128.1, 126.9, 126.7, 126.1, 113.6, 112.3, 111.8, 68.2, 58.3, 56.2, 55.7, 53.4, 31.8, 22.1. HRMS (ESI) calculated for $C_{21}H_{26}NO_2$ $[(M+H)^+]$: 324.1958, found: 324.1954. HPLC (Daicel Chiralpak AD-H column, *n*-hexane/isopropanol = 90/10, flow rate 1.0 mL/min, 254 nm): t_R (major) = 5.1 min, t_R (minor) = 6.7 min.

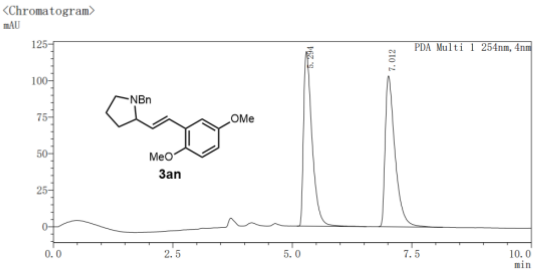

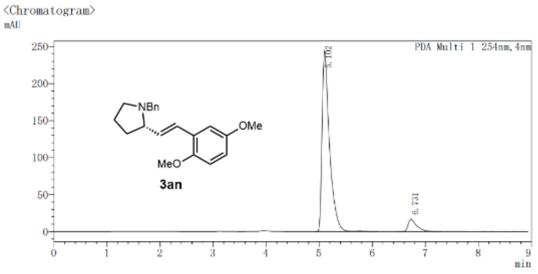

<Peak Table>
PDA Ch1 254nm

No.	Ret. Time (min)	Height (uAU)	Height%	Area (uAU*min)	Area%
1	5.294	119511	53.670	1481442	50.089
2	7.012	103167	46.330	1476153	49.911
Total		222678	100.000	2957595	100.000

<Peak Table>
PDA Ch1 254nm

No.	Ret. Time (min)	Height (uAU)	Height%	Area (uAU*min)	Area%
1	5.102	243960	93.558	2385055	92.613
2	6.731	16797	6.442	190232	7.387
Total		260758	100.000	2575287	100.000

***(S,E)*-1-benzyl-2-(2,4,6-trimethylstyryl)pyrrolidine (3ao):**

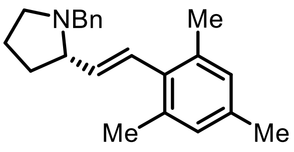

Yellow oil (52.6 mg, 57% yield, 7:1 *E/Z*), 93:7 *er*. $[\alpha]_D^{20}$ = +68.0 (*c* 0.49, $CHCl_3$); ^1H NMR (400 MHz, $CDCl_3$) *E* isomer: δ 7.37 – 7.27 (m, 4H), 7.26 – 7.22 (m, 1H), 6.89 (s, 2H), 6.54

(d, J = 16.2 Hz, 1H), 5.69 (dd, J = 16.2, 8.3 Hz, 1H), 4.21 (d, J = 12.8 Hz, 1H), 3.18 (d, J = 12.8 Hz, 1H), 3.05 – 2.95 (m, 2H), 2.32 (s, 6H), 2.29 (s, 3H), 2.11 – 2.04 (m, 1H), 2.00 – 1.87 (m, 1H), 1.84 – 1.72 (m, 3H); ^{13}C NMR (100 MHz, $CDCl_3$) δ 139.4, 137.3, 136.0, 135.8, 134.0, 129.3, 129.1, 128.5, 128.2, 126.8, 68.4, 58.9, 53.2, 31.8, 22.1, 21.0, 20.9. HRMS (ESI) calculated for $C_{22}H_{28}N$ [(M+H)$^+$]: 306.2216, found: 306.2210. HPLC (Daicel Chiralpak OJ-H column, *n*-hexane/isopropanol = 99/1, flow rate 0.5 mL/min, 254 nm): t_R (minor) = 8.9 min, t_R (major) = 10.1 min.

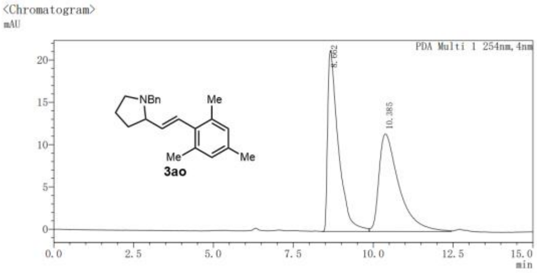

<Peak Table>
PDA Ch1 254nm

No.	Ret.Time(min)	Height(uAU)	Height%	Area(uAU*min)	Area%
1	8.662	21387	64.931	501547	49.762
2	10.385	11551	35.069	506352	50.238
Total		32939	100.000	1007898	100.000

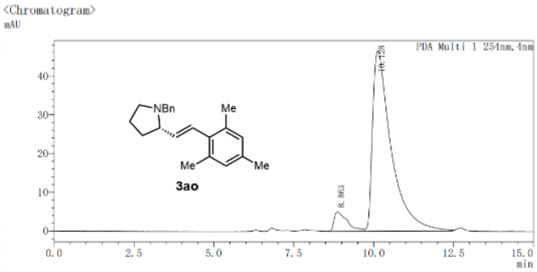

<Peak Table>
PDA Ch1 254nm

No.	Ret.Time(min)	Height(uAU)	Height%	Area(uAU*min)	Area%
1	8.863	4980	9.671	142506	6.989
2	10.128	46512	90.329	1896600	93.011
Total		51492	100.000	2039107	100.000

***(S,E)*-1-benzyl-2-(3,4,5-trimethoxystyryl)pyrrolidine (3ap):**

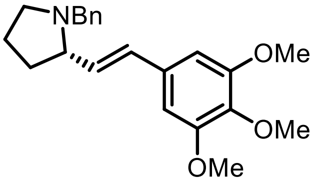

Yellow oil (83.2 mg, 79% yield), 95:5 *er*. $[\alpha]_D^{20}$ = +4.1 (*c* 0.55, $CHCl_3$); ^{1}H NMR (400 MHz, $CDCl_3$) δ 7.36 – 7.27 (m, 4H), 7.25 – 7.18 (m, 1H), 6.63 (s, 2H), 6.49 (d, J = 15.7 Hz, 1H), 6.11 (dd, J = 15.7, 8.3 Hz, 1H), 4.07 (d, J = 13.0 Hz, 1H), 3.88 (s, 6H), 3.85 (s, 3H), 3.17 (d, J = 13.0 Hz, 1H), 3.05 – 2.94 (m, 2H), 2.22 – 2.14 (m, 1H), 2.08 – 2.00 (m, 1H), 1.86 – 1.70 (m, 3H); ^{13}C NMR (100 MHz, $CDCl_3$) δ 153.2, 139.2, 137.6, 132.8, 132.1, 131.4, 128.9, 128.1, 126.7, 103.3, 67.7, 60.8, 58.3, 56.0, 53.4, 31.8, 22.1. HRMS (ESI) calculated for $C_{22}H_{28}NO_3$ [(M+H)$^+$]: 354.2064, found: 354.2061. HPLC (Daicel Chiralpak AD-H column, *n*-hexane/isopropanol = 90/10, flow rate 1.0 mL/min, 254 nm): t_R (major) = 7.2 min, t_R (minor) = 12.9 min.

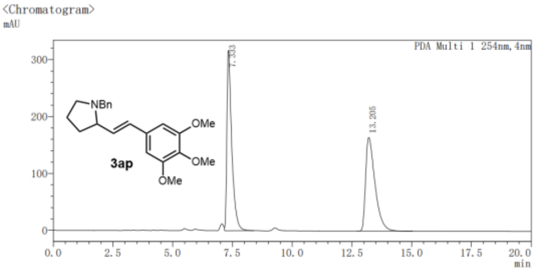

<Peak Table>

PDA Ch1 254nm

No.	Ret. Time (min)	Height (uAU)	Height%	Area (uAU*min)	Area%
1	7.333	316756	65.842	4497583	50.025
2	13.205	164327	34.158	4493165	49.975
Total		481083	100.000	8990748	100.000

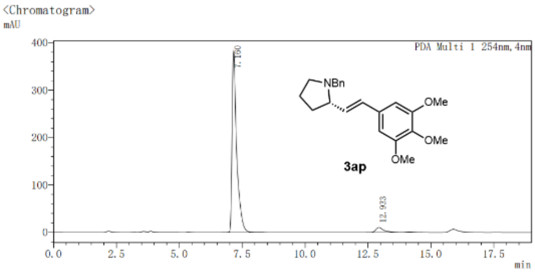

<Peak Table>

PDA Ch1 254nm

No.	Ret. Time (min)	Height (uAU)	Height%	Area (uAU*min)	Area%
1	7.160	382675	97.343	4817002	95.013
2	12.933	10445	2.657	252820	4.987
Total		393121	100.000	5069821	100.000

***(S,E)*-1-benzyl-2-(3,4,5-trifluorostyryl)pyrrolidine (3aq):**

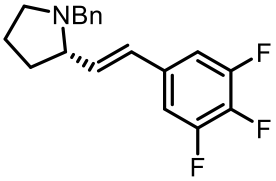

Yellow oil (81.1 mg, 85% yield), 93:7 *er*. $[\alpha]_D^{20}$ = +59.5 (*c* 0.44, $CHCl_3$); ^1H NMR (400 MHz, $CDCl_3$) δ 7.32 (s, 2H), 7.32 – 7.28 (m, 2H), 7.26 – 7.18 (m, 1H), 7.08 – 6.86 (m, 2H), 6.40 (d, *J* = 15.8 Hz, 1H), 6.12 (dd, *J* = 15.8, 8.1 Hz, 1H), 3.99 (d, *J* = 13.0 Hz, 1H), 3.22 (d, *J* = 13.0 Hz, 1H), 3.08 – 2.94 (m, 2H), 2.27 – 2.15 (m, 1H), 2.07 – 2.01 (m, 1H), 1.90 – 1.67 (m, 3H); ^{13}C NMR (100 MHz, $CDCl_3$) δ 151.3 (ddd, *J* = 247.5, 10.0, 4.2 Hz), 139.3, 138.8 (dt, *J* = 249.5, 15.1 Hz), 135.5, 133.5 (td, *J* = 7.5, 4.4 Hz), 128.8, 128.5 (d, *J* = 2.1 Hz), 128.2, 126.8, 109.9 (dd, *J* = 15.8, 5.7 Hz), 67.3, 58.5, 53.5, 31.8, 22.3; ^{19}F NMR (377 MHz, $CDCl_3$) δ -134.9, -162.2. HRMS (ESI) calculated for $C_{19}H_{19}F_3N$ [$(M+H)^+$]: 318.1464, found: 318.1457. HPLC (Daicel Chiralpak OD-H column, *n*-hexane/isopropanol = 95/5, flow rate 1.0 mL/min, 254 nm): t_R (minor) = 3.8 min, t_R (major) = 4.2 min.

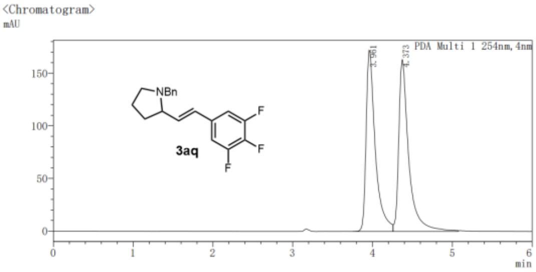

<Peak Table>

PDA Ch1 254nm

No.	Ret. Time (min)	Height (uAU)	Height%	Area (uAU*min)	Area%
1	3.961	172070	51.342	1377957	49.194
2	4.373	163074	48.658	1423131	50.806
Total		335143	100.000	2801089	100.000

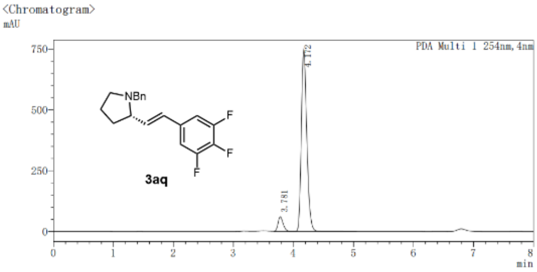

<Peak Table>

PDA Ch1 254nm

No.	Ret. Time (min)	Height (uAU)	Height%	Area (uAU*min)	Area%
1	3.781	60517	7.510	362626	7.091
2	4.172	745305	92.490	4751151	92.909
Total		805822	100.000	5113777	100.000

***(S,E)*-1-benzyl-2-(2-(naphthalen-1-yl)vinyl)pyrrolidine (3ar):**

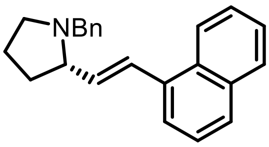

Yellow oil (81.7 mg, 87% yield), 95:5 *er*. $[\alpha]_D^{20}$ = +48.9 (*c* 0.64, $CHCl_3$); ^1H NMR (400 MHz, $CDCl_3$) δ 8.18 (d, *J* = 8.0 Hz, 1H), 7.89 (d, *J* = 7.7 Hz, 1H), 7.81 (d, *J* = 8.2 Hz, 1H), 7.66 (d, *J* = 7.1 Hz, 1H), 7.58 – 7.47 (m, 3H), 7.41 (d, *J* = 7.5 Hz, 2H), 7.39 – 7.33 (m, 3H), 7.29 (d, *J* = 7.0 Hz, 1H), 6.29 (dd, *J* = 15.5, 8.3 Hz, 1H), 4.21 (d, *J* = 13.0 Hz, 1H), 3.29 (d, *J* = 13.0 Hz, 1H), 3.23 – 3.13 (m, 1H), 3.08 (t, *J* = 8.1 Hz, 1H), 2.33 – 2.23 (m, 1H), 2.20 – 2.10 (m, 1H), 1.95 – 1.79 (m, 3H); ^{13}C NMR (100 MHz, $CDCl_3$) δ 139.5, 135.9, 134.8, 133.6, 131.1, 129.0, 128.7, 128.5, 128.1, 127.7, 126.7, 125.9, 125.7, 125.6, 123.9, 123.8, 68.0, 58.4, 53.5, 31.9, 22.2. HRMS (ESI) calculated for $C_{23}H_{24}N$ [$(M+H)^+$]: 314.1903, found: 314.1898. HPLC (Daicel Chiralpak OD-H column, *n*-hexane/isopropanol = 90/10, flow rate 1.0 mL/min, 254 nm): t_R (minor) = 4.6 min, t_R (major) = 5.1 min.

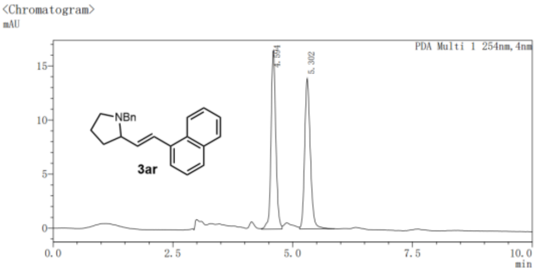

<Peak Table>

PDA Ch1 254nm

No.	Ret.Time(min)	Height(uAU)	Height%	Area(uAU*min)	Area%
1	4.594	16530	54.309	114934	50.089
2	5.302	13907	45.691	114528	49.911
Total		30436	100.000	229462	100.000

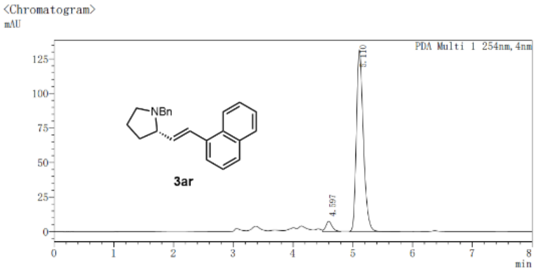

<Peak Table>

PDA Ch1 254nm

No.	Ret.Time(min)	Height(uAU)	Height%	Area(uAU*min)	Area%
1	4.597	7506	5.417	55305	4.937
2	5.110	131051	94.583	1065009	95.063
Total		138557	100.000	1120314	100.000

(*S,E*)-1-benzyl-2-(2-(naphthalen-2-yl)vinyl)pyrrolidine (3as):

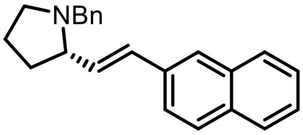

Yellow solid (70.8 mg, 75% yield), 93.5:6.5 *er*. m.p. 109-110 °C; $[\alpha]_D^{20}$ = +28.4 (*c* 0.55, $CHCl_3$); ^1H NMR (400 MHz, $CDCl_3$) δ 7.85 – 7.76 (m, 3H), 7.74 (s, 1H), 7.64 (dd, *J* = 8.6, 1.5 Hz, 1H), 7.50 – 7.41 (m, 2H), 7.38 – 7.28 (m, 4H), 7.26 – 7.20 (m, 1H), 6.74 (d, *J* = 15.8 Hz, 1H), 6.33 (dd, *J* = 15.8, 8.3 Hz, 1H), 4.12 (d, *J* = 13.0 Hz, 1H), 3.20 (d, *J* = 13.0 Hz, 1H), 3.11 – 2.98 (m, 2H), 2.28 – 2.16 (m, 1H), 2.11 – 2.03 (m, 1H), 1.87 – 1.77 (m, 3H); ^{13}C NMR (100 MHz, $CDCl_3$) δ 139.4, 134.6, 133.6, 133.1, 132.9, 131.7, 129.0, 128.1, 127.9, 127.6, 126.8, 126.2, 126.0, 125.7, 123.7, 68.0, 58.4, 53.4, 31.9, 22.2. HRMS (ESI) calculated for $C_{23}H_{24}N$ [$(M+H)^+$]: 314.1903, found: 314.1896.

HPLC (Daicel Chiralpak AD-H column, *n*-hexane/isopropanol = 90/10, flow rate 1.0 mL/min, 254 nm): t_R (major) = 4.6 min, t_R (minor) = 5.4 min.

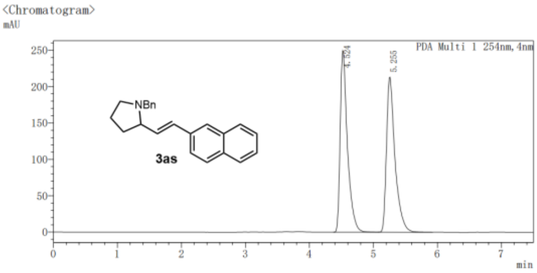

<Peak Table>
PDA Ch1 254nm

No.	Ret.Time(min)	Height(uAU)	Height%	Area(uAU*min)	Area%
1	4.524	249300	53.907	1971681	50.049
2	5.255	213167	46.093	1967831	49.951
Total		462467	100.000	3939513	100.000

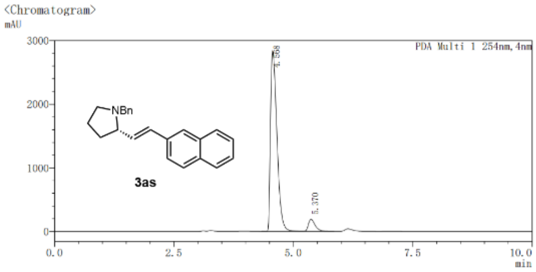

<Peak Table>
PDA Ch1 254nm

No.	Ret.Time(min)	Height(uAU)	Height%	Area(uAU*min)	Area%
1	4.568	2841536	93.706	26801518	93.413
2	5.370	190850	6.294	1889904	6.587
Total		3032386	100.000	28691422	100.000

(*S,E*)-1-benzyl-2-(2-(furan-2-yl)vinyl)pyrrolidine (3at):

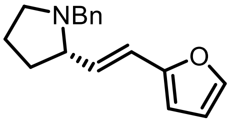

Yellow solid (34.0 mg, 45% yield), 96.5:3.5 *er*. m.p. 43-44 ^oC; $[\alpha]_D^{20}$ = -21.0 (*c* 0.63, $CHCl_3$); ^1H NMR (400 MHz, $CDCl_3$) δ 7.38 – 7.34 (m, 1H), 7.34 – 7.25 (m, 4H), 7.25 – 7.20 (m, 1H), 6.41 (d, *J* = 15.8 Hz, 1H), 6.37 (dd, *J* = 3.1, 1.9 Hz, 1H), 6.23 (d, *J* = 3.2 Hz, 1H), 6.15 (dd, *J* = 15.8, 8.3 Hz, 1H), 4.08 (d, *J* = 13.0 Hz, 1H), 3.14 (d, *J* = 13.0 Hz, 1H), 3.03 – 2.91 (m, 2H), 2.22 – 2.13 (m, 1H), 2.06 – 1.98 (m, 1H), 1.85 – 1.68 (m, 3H); ^{13}C NMR (100 MHz, $CDCl_3$) δ 152.8, 141.7, 139.5, 131.4, 128.9, 128.1, 126.7, 120.0, 111.2, 107.1, 67.5, 58.3, 53.3, 31.9, 22.2. HRMS (ESI) calculated for $C_{17}H_{20}NO$ [$(M+H)^+$]: 254.1539, found: 254.1537. HPLC (Daicel Chiralpak AD-H column, *n*-hexane/isopropanol = 90/10, flow rate 1.0 mL/min, 254 nm): t_R (major) = 3.6 min, t_R (minor) = 4.5 min.

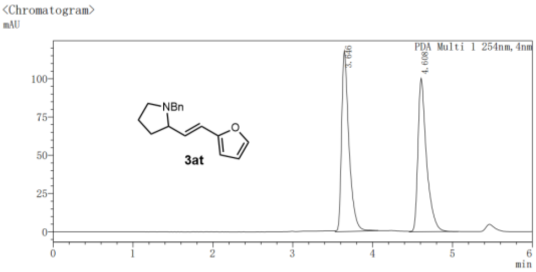

<Peak Table>
PDA Ch1 254nm

No.	Ret.Time(min)	Height(uAU)	Height%	Area(uAU*min)	Area%
1	3.646	118030	54.109	758795	50.301
2	4.608	100104	45.891	749705	49.699
Total		218135	100.000	1508500	100.000

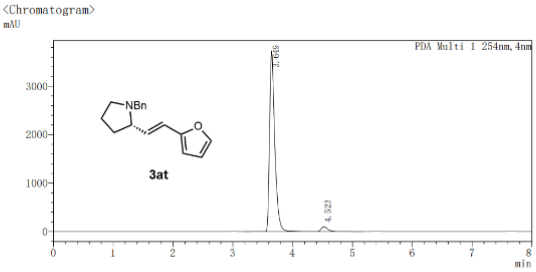

<Peak Table>
PDA Ch1 254nm

No.	Ret.Time(min)	Height(uAU)	Height%	Area(uAU*min)	Area%
1	3.649	3735973	97.427	22581522	96.701
2	4.523	98664	2.573	770463	3.299
Total		3834637	100.000	23351985	100.000

(*S,E*)-2-(2-(benzofuran-2-yl)vinyl)-1-benzylpyrrolidine (3au):

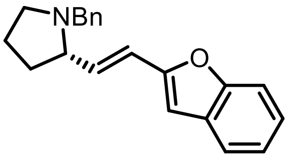

Yellow solid (62.9 mg, 69% yield) with 92.5:7.5 *er*. m.p. 78-79 °C; $[\alpha]_D^{20}$ = +89.7 (*c* 0.27, $CHCl_3$); ^1H NMR (400 MHz, $CDCl_3$) δ 7.54 (dd, *J* = 7.6, 0.6 Hz, 1H), 7.48 (d, *J* = 8.1 Hz, 1H), 7.39 – 7.32 (m, 4H), 7.30 – 7.20 (m, 3H), 6.58 (s, 1H), 6.57 (d, *J* = 15.7 Hz, 2H), 6.50 (dd, *J* = 15.8, 7.3 Hz, 1H), 4.12 (d, *J* = 13.0 Hz, 1H), 3.21 (d, *J* = 13.0 Hz, 1H), 3.12 – 2.98 (m, 2H), 2.27 – 2.18 (m, 1H), 2.13 – 2.03 (m, 1H), 1.91 – 1.74 (m, 3H); ^{13}C NMR (100 MHz, $CDCl_3$) δ 154.7, 154.6, 139.4, 135.1, 128.9, 128.8, 128.1, 126.7, 124.2, 122.7, 120.7, 119.8, 110.8, 103.7, 67.3, 58.4, 53.3, 31.9, 22.3. HRMS (ESI) calculated for $C_{21}H_{22}NO$ [(M+H)$^+$]: 304.1696, found: 304.1688. HPLC (Daicel Chiralpak AD-H column, *n*-hexane/isopropanol = 90/10, flow rate 1.0 mL/min, 254 nm): t_R (major) = 4.0 min, t_R (minor) = 4.5 min.

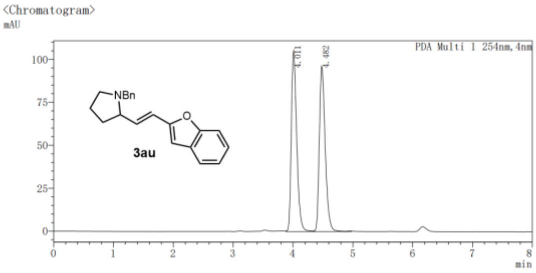

<Peak Table>
PDA Ch1 254nm

No.	Ret. Time(min)	Height(uAU)	Height%	Area(uAU*min)	Area%
1	4.011	105327	52.258	662830	50.033
2	4.482	96226	47.742	661966	49.967
Total		201553	100.000	1324795	100.000

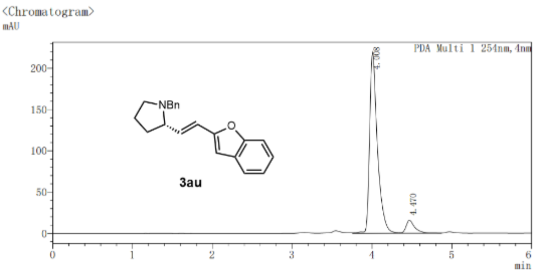

<Peak Table>
PDA Ch1 254nm

No.	Ret. Time(min)	Height(uAU)	Height%	Area(uAU*min)	Area%
1	4.008	219067	93.277	1490205	92.417
2	4.470	15791	6.723	122269	7.583
Total		234858	100.000	1612473	100.000

***(S,E)*-1-benzyl-2-(2-(2,3-dihydrobenzo[b][1,4]dioxin-6-yl)vinyl)pyrrolidine (3av):**

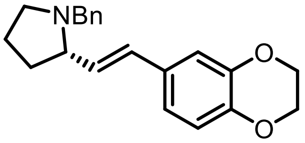

Yellow oil (58.1 mg, 60% yield), 93.5:6.5 *er*. $[\alpha]_D^{20}$ = -7.7 (*c* 0.73, $CHCl_3$); ^1H NMR (400 MHz, $CDCl_3$) δ 7.37 – 7.27 (m, 4H), 7.25 – 7.18 (m, 1H), 6.94 (s, 1H), 6.90 (d, *J* = 8.4 Hz, 1H), 6.82 (d, *J* = 8.3 Hz, 1H), 6.45 (d, *J* = 15.8 Hz, 1H), 6.05 (dd, *J* = 15.8, 8.3 Hz, 1H), 4.25 (s, 4H), 4.07 (d, *J* = 12.9 Hz, 1H), 3.13 (d, *J* = 12.9 Hz, 1H), 3.03 – 2.89 (m, 2H), 2.20 – 2.13 (m, 1H), 2.06 – 1.98 (m, 1H), 1.84 – 1.68 (m, 3H); ^{13}C NMR (100 MHz, $CDCl_3$) δ 143.5, 143.0, 139.3, 131.1, 131.0, 130.9, 129.0, 128.1, 126.7, 119.7, 117.2, 114.8, 67.8, 64.4, 64.3, 58.2, 53.3, 31.8, 22.1. HRMS (ESI) calculated for $C_{21}H_{24}NO_2$ [(M+H)$^+$]: 322.1802, found: 322.1796. HPLC (Daicel Chiralpak AD-H column, *n*-hexane/isopropanol = 90/10, flow rate 1.0 mL/min, 254 nm): t_R (major) = 5.2 min, t_R (minor) = 6.9 min.

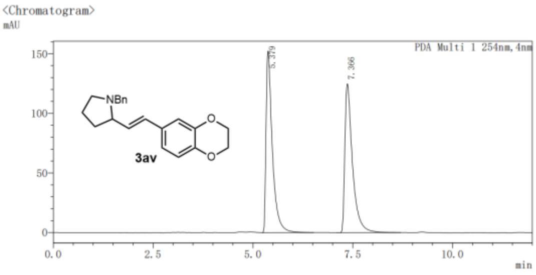

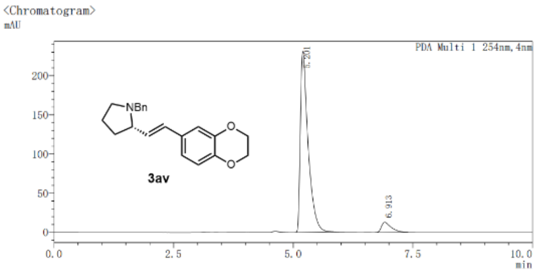

<Peak Table>
PDA Ch1 254nm

No.	Ret. Time (min)	Height (uAU)	Height%	Area (uAU*min)	Area%
1	5.379	151951	54.925	1676883	50.071
2	7.366	124699	45.075	1672101	49.929
Total		276650	100.000	3348983	100.000

<Peak Table>
PDA Ch1 254nm

No.	Ret. Time (min)	Height (uAU)	Height%	Area (uAU*min)	Area%
1	5.201	231044	94.687	2619998	93.486
2	6.913	12965	5.313	182552	6.514
Total		244009	100.000	2802550	100.000

***(S,E)*-1-benzyl-2-(2-(thiophen-2-yl)vinyl)pyrrolidine (3aw):**

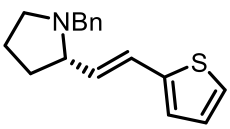

Yellow solid (63.7 mg, 79% yield), 93:7 *er*. m.p. 47-48 °C; $[\alpha]_D^{20}$ = -110.1 (*c* 0.98, $CHCl_3$); ^1H NMR (400 MHz, $CDCl_3$) δ 7.36 – 7.28 (m, 4H), 7.26 – 7.19 (m, 1H), 7.15 (d, *J* = 4.3 Hz, 1H), 7.02 – 6.91 (m, 2H), 6.71 (d, *J* = 15.7 Hz, 1H), 6.06 (dd, *J* = 15.6, 8.2 Hz, 1H), 4.09 (d, *J* = 13.0 Hz, 1H), 3.16 (d, *J* = 13.0 Hz, 1H), 3.03 – 2.92 (m, 2H), 2.23 – 2.14 (m, 1H), 2.08 – 1.98 (m, 1H), 1.88 – 1.71 (m, 3H); ^{13}C NMR (100 MHz, $CDCl_3$) δ 142.4, 139.5, 132.5, 129.0, 128.2, 127.3, 126.8, 125.1, 124.7, 123.9, 67.6, 58.4, 53.4, 31.9, 22.2. HRMS (ESI) calculated for $C_{17}H_{20}NS$ [$(M+H)^+$]: 270.1311, found: 270.1310. HPLC (Daicel Chiralpak AD-H column, *n*-hexane/isopropanol = 90/10, flow rate 1.0 mL/min, 254 nm): t_R (major) = 3.9 min, t_R (minor) = 4.8 min.

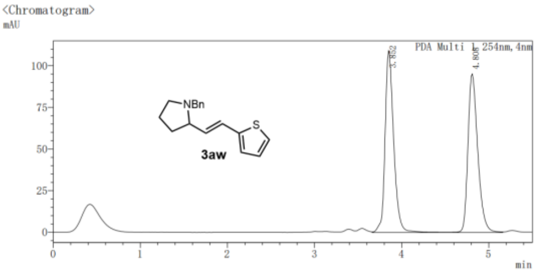

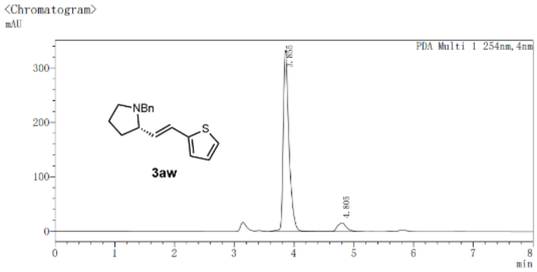

<Peak Table>
PDA Ch1 254nm

No.	Ret. Time (min)	Height (uAU)	Height%	Area (uAU*min)	Area%
1	3.852	108963	53.374	752303	50.218
2	4.808	95185	46.626	745762	49.782
Total		204148	100.000	1498065	100.000

<Peak Table>
PDA Ch1 254nm

No.	Ret. Time (min)	Height (uAU)	Height%	Area (uAU*min)	Area%
1	3.855	332702	95.449	2216802	93.077
2	4.805	15864	4.551	164883	6.923
Total		348566	100.000	2381685	100.000

***(S,E)*-1-(2-bromobenzyl)-2-styrylpyrrolidine (3ba):**

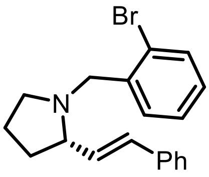

Yellow oil (86.7 mg, 85% yield), 91:9 *er*. $[\alpha]_D^{20}$ = +4.7 (*c* 0.60, $CHCl_3$); ^1H NMR (400 MHz, $CDCl_3$) δ 7.52 (d, *J* = 7.8 Hz, 2H), 7.40 (d, *J* = 7.7 Hz, 2H), 7.35 – 7.26 (m, 3H), 7.25 – 7.19 (m, 1H), 7.11 – 7.04 (m, 1H), 6.58 (d, *J* = 15.8 Hz, 1H), 6.20 (dd, *J* = 15.8, 8.3 Hz,

1H), 4.07 (d, *J* = 14.1 Hz, 1H), 3.42 (d, *J* = 14.1 Hz, 1H), 3.15 – 3.06 (m, 2H), 2.31 – 2.22 (m, 1H), 2.10 – 1.98 (m, 1H), 1.89 – 1.74 (m, 3H); ^{13}C NMR (100 MHz, $CDCl_3$) δ 139.0, 137.1, 132.7, 132.6, 131.5, 130.8, 128.5, 128.1, 127.3, 127.1, 126.3, 124.2, 68.2, 57.5, 53.7, 31.9, 22.4. HRMS (ESI) calculated for $C_{19}H_{21}BrN$ [(M+H)$^+$]: 342.0852, found: 342.0852. HPLC (Daicel Chiralpak OJ-H column, *n*-hexane/isopropanol = 99/1, flow rate 0.5 mL/min, 254 nm): t_R (minor) = 13.6 min, t_R (major) = 18.4 min.

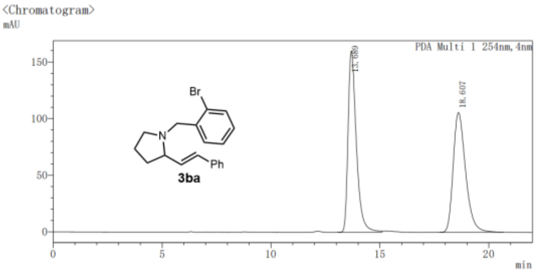

<Peak Table>

PDA Ch1 254nm

No.	Ret.Time(min)	Height(uAU)	Height%	Area(uAU*min)	Area%
1	13.689	160245	60.229	4248928	50.330
2	18.607	105816	39.771	4193161	49.670
Total		266061	100.000	8442089	100.000

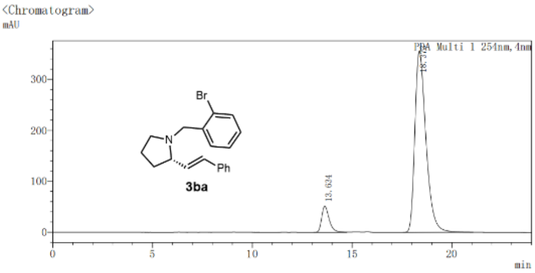

<Peak Table>

PDA Ch1 254nm

No.	Ret.Time(min)	Height(uAU)	Height%	Area(uAU*min)	Area%
1	13.634	51538	12.647	1355382	8.933
2	18.373	355966	87.353	13817580	91.067
Total		407504	100.000	15172961	100.000

***(S,E)*-1-(2-methylbenzyl)-2-styrylpyrrolidine (3ca):**

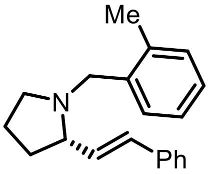

Orange oil (70.0 mg, 84% yield), 94:6 *er*. $[\alpha]_D^{20}$ = -3.3 (*c* 0.68, $CHCl_3$); ^{1}H NMR (400 MHz, $CDCl_3$) δ 7.45 (d, *J* = 7.7 Hz, 2H), 7.40 – 7.33 (m, 3H), 7.29 (d, *J* = 7.1 Hz, 1H), 7.22 – 7.14 (m, 3H), 6.62 (d, *J* = 15.8 Hz, 1H), 6.25 (dd, *J* = 15.8, 8.4 Hz, 1H), 4.11 (d, *J* = 13.1 Hz, 1H), 3.14 (d, *J* = 13.1 Hz, 1H), 3.10 – 2.99 (m, 2H), 2.40 (s, 3H), 2.27 – 2.18 (m, 1H), 2.13 – 2.02 (m, 1H), 1.91 – 1.75 (m, 3H); ^{13}C NMR (100 MHz, $CDCl_3$) δ 137.9, 137.2, 136.8, 132.8, 131.5, 130.1, 129.4, 128.5, 127.2, 126.7, 126.3, 125.4, 68.5, 56.2, 53.8, 31.9, 22.3, 19.3. HRMS (ESI) calculated for $C_{20}H_{24}N$ [(M+H)$^+$]: 278.1903, found: 278.1901. HPLC (Daicel Chiralpak OJ-H column, *n*-hexane/isopropanol = 99/1, flow rate 0.5 mL/min, 254 nm): t_R (minor) = 15.5 min, t_R (major) = 17.6 min.

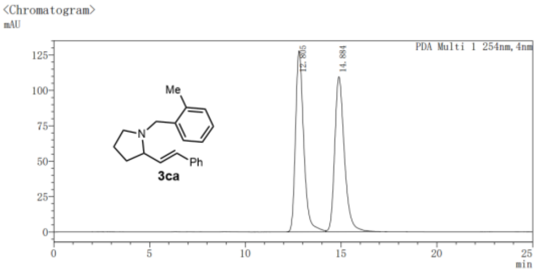

<Peak Table>
PDA Ch1 254nm

No.	Ret.Time(min)	Height(uAU)	Height%	Area(uAU*min)	Area%
1	12.805	127858	53.885	3818599	49.884
2	14.884	109423	46.115	3836391	50.116
Total		237281	100.000	7654990	100.000

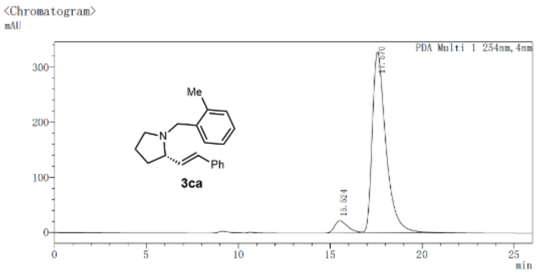

<Peak Table>
PDA Ch1 254nm

No.	Ret.Time(min)	Height(uAU)	Height%	Area(uAU*min)	Area%
1	15.524	22127	6.317	1112859	6.138
2	17.570	328130	93.683	17016528	93.862
Total		350258	100.000	18129387	100.000

***(S,E)*-1-(3-fluorobenzyl)-2-styrylpyrrolidine (3da):**

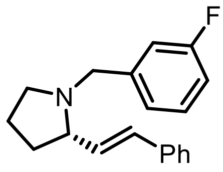

Yellow solid (78.1 mg, 93% yield), 93:7 *er*. m.p. 43-44 °C; $[\alpha]_D^{20}$ = -11.0 (*c* 0.63, $CHCl_3$); ^{1}H NMR (400 MHz, $CDCl_3$) δ 7.45 (d, *J* = 7.7 Hz, 2H), 7.40 – 7.33 (m, 2H), 7.32 – 7.26 (m, 2H), 7.17 – 7.09 (m, 2H), 6.99 – 6.90 (m, 1H), 6.60 (d, *J* = 15.8 Hz, 1H), 6.22 (dd, *J* = 15.8, 8.3 Hz, 1H), 4.10 (d, *J* = 13.3 Hz, 1H), 3.19 (d, *J* = 13.3 Hz, 1H), 3.09 – 2.99 (m, 2H), 2.26 – 2.16 (m, 1H), 2.13 – 2.03 (m, 1H), 1.92 – 1.77 (m, 3H); ^{13}C NMR (100 MHz, $CDCl_3$) δ 162.9 (d, *J* = 243.6 Hz), 142.4 (d, *J* = 7.0 Hz), 137.0, 132.4, 131.8, 129.5 (d, *J* = 8.3 Hz), 128.5, 127.4, 126.3, 124.3 (d, *J* = 2.5 Hz), 115.6 (d, *J* = 21.0 Hz), 113.5 (d, *J* = 21.1 Hz), 67.9, 57.8, 53.4, 31.8, 22.2; ^{19}F NMR (377 MHz, $CDCl_3$) δ -113.9. HRMS (ESI) calculated for $C_{19}H_{21}FN$ $[(M+H)^+]$: 282.1653, found: 282.1650. HPLC (Daicel Chiralpak AD-H column, *n*-hexane/isopropanol = 99/1, flow rate 0.5 mL/min, 254 nm): t_R (major) = 9.4 min, t_R (minor) = 10.7 min.

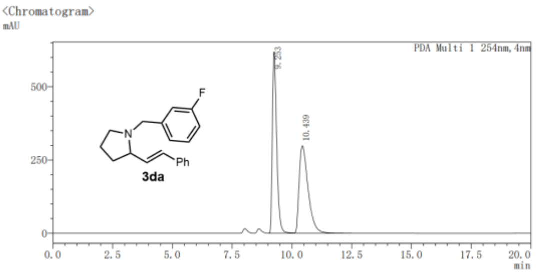

<Peak Table>
PDA Ch1 254nm

No.	Ret.Time(min)	Height(uAU)	Height%	Area(uAU*min)	Area%
1	9.253	618366	67.445	7793995	49.928
2	10.439	298484	32.555	7816568	50.072
Total		916850	100.000	15610563	100.000

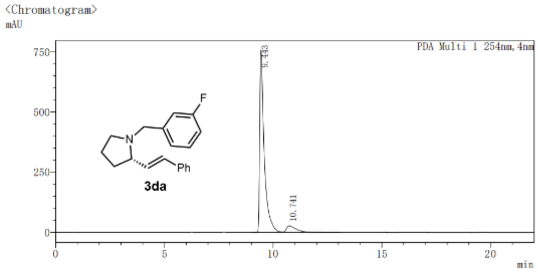

<Peak Table>
PDA Ch1 254nm

No.	Ret.Time(min)	Height(uAU)	Height%	Area(uAU*min)	Area%
1	9.443	755522	96.583	10896917	92.786
2	10.741	26729	3.417	847266	7.214
Total		782250	100.000	11744183	100.000

***(S,E)*-1-(3-methylbenzyl)-2-styrylpyrrolidine (3ea):**

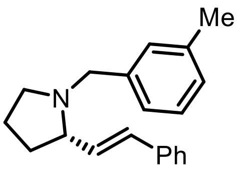

Orange oil (72.6 mg, 87% yield), 93:7 *er*. $[\alpha]_D^{20}$ = -39.5 (*c* 0.83, $CHCl_3$); ^1H NMR (400 MHz, $CDCl_3$) δ 7.46 (d, *J* = 7.7 Hz, 2H), 7.40 – 7.33 (m, 2H), 7.31 – 7.22 (m, 2H), 7.21 – 7.14 (m, 2H), 7.09 (d, *J* = 7.4 Hz, 1H), 6.61 (d, *J* = 15.8 Hz, 1H), 6.26 (dd, *J* = 15.8, 8.3 Hz, 1H), 4.09 (d, *J* = 12.9 Hz, 1H), 3.16 (d, *J* = 12.9 Hz, 1H), 3.09 – 2.99 (m, 2H), 2.39 (s, 3H), 2.27 – 2.18 (m, 1H), 2.13 – 2.03 (m, 1H), 1.93 – 1.77 (m, 3H); ^{13}C NMR (100 MHz, $CDCl_3$) δ 139.3, 137.7, 137.1, 132.6, 131.6, 129.7, 128.5, 128.0, 127.5, 127.3, 126.3, 126.1, 68.0, 58.4, 53.4, 31.8, 22.1, 21.4. HRMS (ESI) calculated for $C_{20}H_{24}N$ [(M+H)$^+$]: 278.1903, found: 278.1902. HPLC (Daicel Chiralpak OD-H column, *n*-hexane/isopropanol = 99/1, flow rate 0.5 mL/min, 254 nm): t_R (minor) = 8.9 min, t_R (major) = 11.3 min.

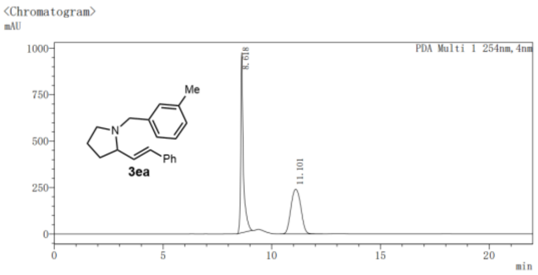

<Peak Table>

PDA Ch1 254nm

No.	Ret.Time(min)	Height(uAU)	Height%	Area(uAU*min)	Area%
1	8.618	970741	80.121	7938264	50.862
2	11.101	240848	19.879	7669215	49.138
Total		1211589	100.000	15607479	100.000

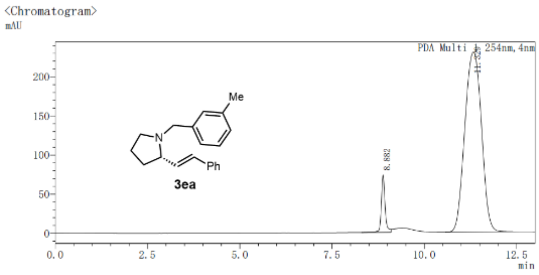

<Peak Table>

PDA Ch1 254nm

No.	Ret.Time(min)	Height(uAU)	Height%	Area(uAU*min)	Area%
1	8.882	73062	24.113	528550	6.827
2	11.329	229936	75.887	7213522	93.173
Total		302998	100.000	7742072	100.000

(*S,E*)-1-(4-bromobenzyl)-2-styrylpyrrolidine (3fa):

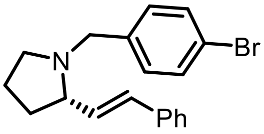

Orange oil (89.8 mg, 88% yield), 90.5:9.5 *er*. $[\alpha]_D^{20}$ = +42.9 (*c* 0.58, $CHCl_3$); ^1H NMR (400 MHz, $CDCl_3$) δ 7.47 – 7.37 (m, 4H), 7.36 – 7.30 (m, 2H), 7.27 – 7.18 (m, 3H), 6.56 (d, *J* = 15.8 Hz, 1H), 6.17 (dd, *J* = 15.8, 8.3 Hz, 1H), 4.01 (d, *J* = 13.2 Hz, 1H), 3.12 (d, *J* = 13.2 Hz, 1H), 3.03 – 2.94 (m, 2H), 2.21 – 2.12 (m, 1H), 2.09 – 2.00 (m, 1H), 1.88 – 1.71 (m, 3H); ^{13}C NMR (100 MHz, $CDCl_3$) δ 138.6, 137.0, 132.4, 131.8, 131.2, 130.6, 128.5, 127.4, 126.3, 120.5, 67.9, 57.6, 53.3, 31.8, 22.2. HRMS (ESI) calculated for $C_{19}H_{21}BrN$ [(M+H)$^+$]: 342.0852, found: 342.0846. HPLC (Daicel Chiralpak AD-H column, *n*-hexane/isopropanol = 90/10, flow rate 1.0 mL/min, 254 nm): t_R (major) = 4.0 min, t_R (minor) = 4.9 min.

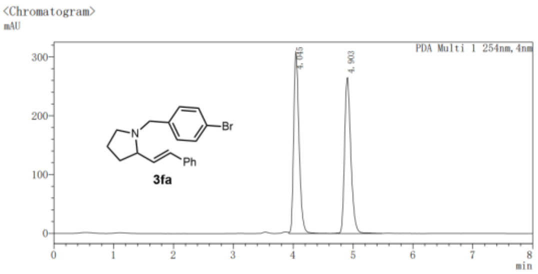

<Peak Table>
PDA Ch1 254nm

No.	Ret. Time (min)	Height (uAU)	Height%	Area (uAU*min)	Area%
1	4.045	308147	53.769	1927965	49.982
2	4.903	264950	46.231	1929389	50.018
Total		573098	100.000	3857355	100.000

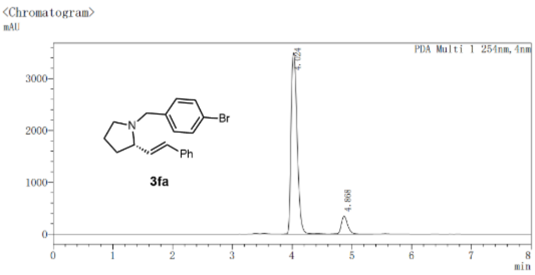

<Peak Table>
PDA Ch1 254nm

No.	Ret. Time (min)	Height (uAU)	Height%	Area (uAU*min)	Area%
1	4.024	3489699	90.894	24844329	90.498
2	4.868	349597	9.106	2608700	9.502
Total		3839296	100.000	27453029	100.000

(*S,E*)-1-(4-methoxybenzyl)-2-styrylpyrrolidine (3ga):

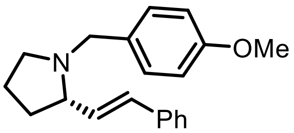

Yellow solid (72.4 mg, 82% yield), 92:8 *er*. m.p. 46-47 °C; $[\alpha]_D^{20}$ = +18.5 (*c* 0.56, $CHCl_3$); ^1H NMR (400 MHz, $CDCl_3$) δ 7.42 (d, *J* = 7.7 Hz, 2H), 7.37 – 7.30 (m, 2H), 7.24 (d, *J* = 8.2 Hz, 3H), 6.85 (d, *J* = 8.2 Hz, 2H), 6.56 (d, *J* = 15.8 Hz, 1H), 6.21 (dd, *J* = 15.8, 8.3 Hz, 1H), 4.01 (d, *J* = 12.8 Hz, 1H), 3.79 (s, 3H), 3.13 (d, *J* = 12.8 Hz, 1H), 3.03 – 2.92 (m, 2H), 2.23 – 2.14 (m, 1H), 2.05 – 2.00 (m, 1H), 1.88 – 1.70 (m, 3H); ^{13}C NMR (100 MHz, $CDCl_3$) δ 158.5, 137.1, 132.7, 131.5, 131.4, 130.1, 128.5, 127.3, 126.3, 113.5, 67.7, 57.6, 55.2, 53.2, 31.8, 22.1. HRMS (ESI) calculated for $C_{20}H_{24}NO$ [(M+H)$^+$]: 294.1852, found: 294.1851. HPLC (Daicel Chiralpak OJ-H column, *n*-hexane/isopropanol = 99/1, flow rate 0.5 mL/min, 254 nm): t_R (major) = 30.5 min, t_R (minor) = 40.2 min.

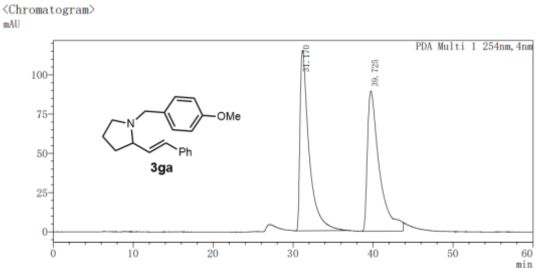

<Peak Table>
PDA Ch1 254nm

No.	Ret. Time (min)	Height (uAU)	Height%	Area (uAU*min)	Area%
1	31.170	114799	56.245	9102313	49.459
2	39.725	89305	43.755	9301313	50.541
Total		204104	100.000	18403626	100.000

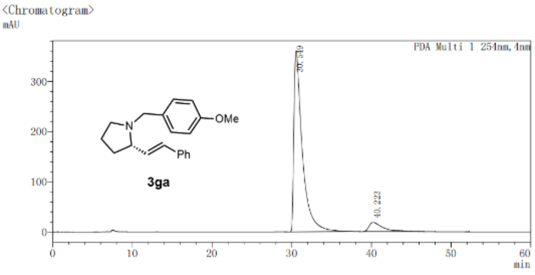

<Peak Table>
PDA Ch1 254nm

No.	Ret. Time (min)	Height (uAU)	Height%	Area (uAU*min)	Area%
1	30.549	360813	95.177	26689860	92.111
2	40.223	18283	4.823	2286025	7.889
Total		379096	100.000	28975884	100.000

(*S,E*)-1-(4-(*tert*-butyl)benzyl)-2-styrylpyrrolidine (3ha):

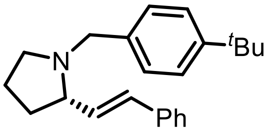

Yellow oil (84.3 mg, 88% yield), 95:5 *er*. $[\alpha]_D^{20}$ = +26.4 (*c* 0.59, $CHCl_3$); ^1H NMR (400 MHz, $CDCl_3$) δ 7.43 (d, *J* = 7.7 Hz, 2H), 7.38 – 7.32 (m, 4H), 7.31 – 7.25 (m, 3H), 6.59 (d, *J* = 15.8 Hz,

1H), 6.22 (dd, *J* = 15.8, 8.3 Hz, 1H), 4.05 (d, *J* = 13.0 Hz, 1H), 3.21 (d, *J* = 13.0 Hz, 1H), 3.11 – 2.98 (m, 2H), 2.28 – 2.19 (m, 1H), 2.08 – 2.02 (m, 1H), 1.91 – 1.74 (m, 3H), 1.34 (s, 9H); ^{13}C NMR (100 MHz, $CDCl_3$) δ 149.5, 137.2, 136.3, 132.8, 131.4, 128.7, 128.5, 127.3, 126.3, 125.0, 67.8, 57.9, 53.5, 34.4, 31.9, 31.4, 22.2. HRMS (ESI) calculated for $C_{23}H_{30}N$ [(M+H)$^+$]: 320.2373, found: 320.2367. HPLC (Daicel Chiralpak OJ-H+OJ-H column, *n*-hexane/isopropanol = 99/1, flow rate 0.5 mL/min, 254 nm): t_R (major) = 22.5 min, t_R (minor) = 32.1 min.

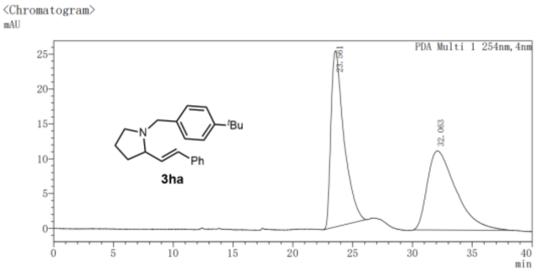

<Peak Table>

PDA Ch1 254nm

No.	Ret. Time (min)	Height (uAU)	Height%	Area (uAU*min)	Area%
1	23.561	25257	69.000	1919260	50.441
2	32.063	11348	31.000	1885670	49.559
Total		36605	100.000	3804930	100.000

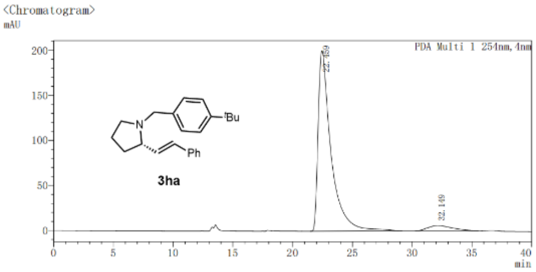

<Peak Table>

PDA Ch1 254nm

No.	Ret. Time (min)	Height (uAU)	Height%	Area (uAU*min)	Area%
1	22.459	200387	97.166	15042731	94.722
2	32.149	5845	2.834	838208	5.278
Total		206232	100.000	15880939	100.000

(*S,E*)-1-(3,5-dimethoxybenzyl)-2-styrylpyrrolidine (3ia):

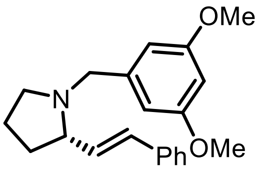

Yellow oil (80.5 mg, 83% yield), 94:6 *er*. $[\alpha]_D^{20}$ = +17.6 (*c* 0.62, $CHCl_3$); ^{1}H NMR (400 MHz, $CDCl_3$) δ 7.41 (d, *J* = 7.7 Hz, 2H), 7.36 – 7.30 (m, 2H), 7.26 – 7.22 (m, 1H), 6.57 (d, *J* = 15.8 Hz, 1H), 6.52 (s, 2H), 6.35 (s, 1H), 6.20 (dd, *J* = 15.8, 8.3 Hz, 1H), 4.02 (d, *J* = 13.1 Hz, 1H), 3.79 (s, 6H), 3.12 (d, *J* = 13.1 Hz, 1H), 3.09 – 2.94 (m, 2H), 2.24 – 2.16 (m, 1H), 2.07 – 2.00 (m, 1H), 1.89 – 1.69 (m, 3H); ^{13}C NMR (100 MHz, $CDCl_3$) δ 160.6, 141.9, 137.1, 132.6, 131.6, 128.5, 127.3, 126.3, 106.8, 98.8, 67.8, 58.4, 55.2, 53.4, 31.8, 22.2. HRMS (ESI) calculated for $C_{21}H_{26}NO_2$ [(M+H)$^+$]: 324.1958, found: 324.1956. HPLC (Daicel Chiralpak OD-H column, *n*-hexane/isopropanol = 99/1, flow rate 0.5 mL/min, 254 nm): t_R (minor) = 17.7 min, t_R (major) = 24.4 min.

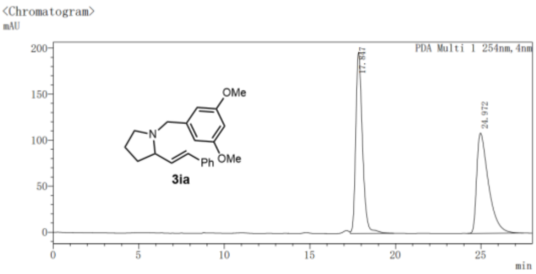

<Peak Table>
PDA Ch1 254nm

No.	Ret. Time (min)	Height (uAU)	Height%	Area (uAU*min)	Area%
1	17.847	196715	64.387	5362649	50.749
2	24.972	108803	35.613	5204305	49.251
Total		305518	100.000	10566954	100.000

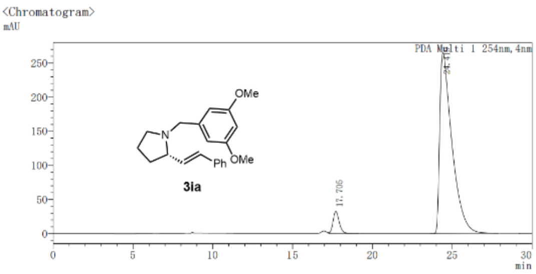

<Peak Table>
PDA Ch1 254nm

No.	Ret. Time (min)	Height (uAU)	Height%	Area (uAU*min)	Area%
1	17.705	32583	10.954	881560	5.859
2	24.419	264863	89.046	14163560	94.141
Total		297446	100.000	15045120	100.000

(*S,E*)-1-(naphthalen-1-ylmethyl)-2-styrylpyrrolidine (3ja):

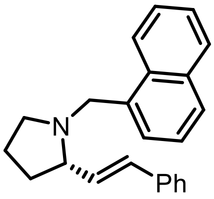

Yellow solid (82.8 mg, 88% yield), 95:5 *er*. m.p. 59-60 °C; $[\alpha]_D^{20}$ = +36.8 (*c* 0.58, $CHCl_3$); ^{1}H NMR (400 MHz, $CDCl_3$) δ 8.32 – 8.25 (m, 1H), 7.88 – 7.82 (m, 1H), 7.76 (d, *J* = 8.1 Hz, 1H), 7.54 – 7.44 (m, 5H), 7.42 (d, *J* = 8.0 Hz, 1H), 7.39 – 7.34 (m, 2H), 7.28 – 7.23 (m, 1H), 6.65 (d, *J* = 15.8 Hz, 1H), 6.33 (dd, *J* = 15.8, 8.4 Hz, 1H), 4.60 (d, *J* = 13.0 Hz, 1H), 3.49 (d, *J* = 13.1 Hz, 1H), 3.18 – 3.07 (m, 1H), 2.99 – 2.91 (m, 1H), 2.31 – 2.22 (m, 1H), 2.14 – 2.04 (m, 1H), 1.85 – 1.73 (m, 3H); ^{13}C NMR (100 MHz, $CDCl_3$) δ 137.2, 135.7, 133.8, 132.8, 132.2, 131.8, 128.5, 128.4, 127.6, 127.3, 126.8, 126.3, 125.7, 125.4, 125.2, 124.6, 68.7, 56.4, 54.0, 32.0, 22.3. HRMS (ESI) calculated for $C_{23}H_{24}N$ $[(M+H)^+]$: 314.1903, found: 314.1896. HPLC (Daicel Chiralpak OJ-H+OJ-H column, *n*-hexane/isopropanol = 99/1, flow rate 0.5 mL/min, 254 nm): t_R (minor) = 11.3 min, t_R (major) = 12.4 min.

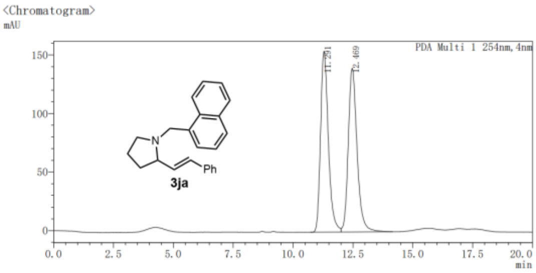

<Peak Table>
PDA Ch1 254nm

No.	Ret. Time (min)	Height (uAU)	Height%	Area (uAU*min)	Area%
1	11.291	154424	52.488	3500500	49.444
2	12.469	139785	47.512	3579286	50.556
Total		294209	100.000	7079787	100.000

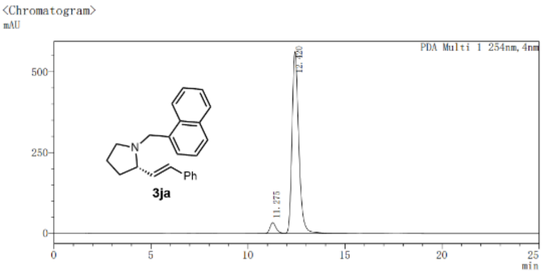

<Peak Table>
PDA Ch1 254nm

No.	Ret. Time (min)	Height (uAU)	Height%	Area (uAU*min)	Area%
1	11.275	33295	5.586	731189	5.086
2	12.420	562759	94.414	13644900	94.914
Total		596054	100.000	14376090	100.000

(*S,E*)-2-styryl-1-tosylpyrrolidine (3ka):

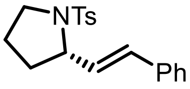

Purified by chromatography on silica gel, eluting with PE/EA = 10/1; yellow solid (87.0 mg, 89% yield), 94.5:5.5 *er*. m.p. 119-120 °C;

$[\alpha]_D^{20}$ = +5.3 (*c* 0.54, $CHCl_3$); ^1H NMR (400 MHz, $CDCl_3$) δ 7.72 (d, *J* = 8.2 Hz, 2H), 7.34 – 7.26 (m, 5H), 7.26 – 7.19 (m, 2H), 6.55 (dd, *J* = 15.8, 1.3 Hz, 1H), 6.05 (dd, *J* = 15.8, 6.7 Hz, 1H), 4.42 – 4.29 (m, 1H), 3.54 – 3.44 (m, 1H), 3.41 – 3.28 (m, 1H), 2.40 (s, 3H), 1.94 – 1.81 (m, 2H), 1.78 – 1.65 (m, 2H); ^{13}C NMR (100 MHz, $CDCl_3$) δ 143.2, 136.6, 135.6, 130.6, 129.9, 129.5, 128.4, 127.6, 127.5, 126.5, 61.6, 48.6, 32.8, 23.9, 21.4. HRMS (ESI) calculated for $C_{19}H_{21}NNaO_2S$ [(M+Na)$^+$]: 350.1158, found: 350.1182. HPLC (Daicel Chiralpak OD-H column, *n*-hexane/isopropanol = 90/10, flow rate 1.0 mL/min, 254 nm): t_R (major) = 10.3 min, t_R (minor) = 13.1 min.

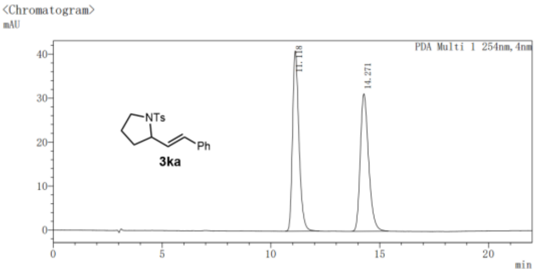

<Peak Table>
PDA Ch1 254nm

No.	Ret.Time(min)	Height(uAU)	Height%	Area(uAU*min)	Area%
1	11.118	40985	56.738	840099	50.225
2	14.271	31250	43.262	832579	49.775
Total		72235	100.000	1672678	100.000

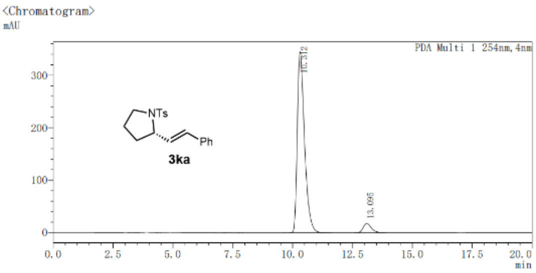

<Peak Table>
PDA Ch1 254nm

No.	Ret.Time(min)	Height(uAU)	Height%	Area(uAU*min)	Area%
1	10.312	344922	95.092	7231516	94.469
2	13.095	17802	4.908	423393	5.531
Total		362724	100.000	7654908	100.000

***(S,E)*-2-(3-methoxy-4-(methoxymethoxy)styryl)-1-((4-nitrophenyl)sulfonyl)pyrrolidine (3lx):**

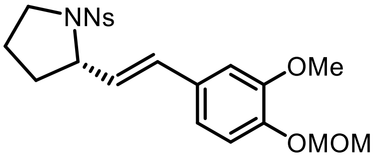

Purified by chromatography on silica gel, eluting with PE/EA/DCM = 10/1/1); yellow solid (698.1 mg, 87% yield), 95.5:4.5 *er*. m.p. 119-120 °C; $[\alpha]_D^{20}$ = -95.7 (*c* 0.62, $CHCl_3$); ^1H NMR (400 MHz, $CDCl_3$) δ 8.27 (d, *J* = 8.4 Hz, 2H), 7.99 (d, *J* = 8.5 Hz, 2H), 7.07 (d, *J* = 8.1 Hz, 1H), 6.90 – 6.74 (m, 2H), 6.49 (d, *J* = 15.6 Hz, 1H), 5.79 (dd, *J* = 15.7, 7.4 Hz, 1H), 5.23 (s, 2H), 4.55 – 4.35 (m, 1H), 3.87 (s, 3H), 3.62 – 3.43 (m, 5H), 2.05 – 1.91 (m, 2H), 1.90 – 1.73 (m, 2H); ^{13}C NMR (100 MHz, $CDCl_3$) δ 149.83, 149.81, 146.5, 145.1, 131.5, 130.6, 128.6, 127.2, 124.1, 119.4, 116.3, 109.6, 95.5, 62.3, 56.2, 55.9, 48.7, 33.0, 24.1. HRMS (ESI) calculated for $C_{21}H_{24}N_2NaO_7S$ [(M+Na)$^+$]: 471.1196, found: 471.1207. HPLC (Daicel Chiralpak AD-H column, *n*-hexane/isopropanol = 90/10, flow rate 1.0 mL/min, 254 nm): t_R (minor) = 63.8 min, t_R (major) = 79.9 min.

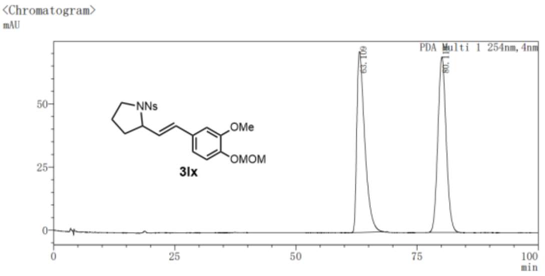

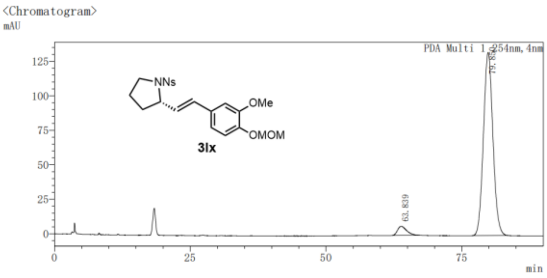

<Peak Table>
PDA Ch1 254nm

No.	Ret. Time (min)	Height (uAU)	Height%	Area (uAU*min)	Area%
1	63.109	71588	50.771	8207776	49.763
2	80.119	69414	49.229	8286048	50.237
Total		141001	100.000	16493824	100.000

<Peak Table>
PDA Ch1 254nm

No.	Ret. Time (min)	Height (uAU)	Height%	Area (uAU*min)	Area%
1	63.839	6613	4.748	770460	4.554
2	79.850	132667	95.252	16148560	95.446
Total		139280	100.000	16919020	100.000

(S,E)-2-(3-chlorostyryl)-1-((4-nitrophenyl)sulfonyl)pyrrolidine (3ly):

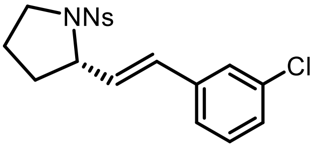

Purified by chromatography on silica gel, eluting with PE/EA/DCM = 20/1/1; yellow solid (708.0 mg, 90% yield), 94:6 *er*. m.p. 130-131 ºC; $[\alpha]_D^{20}$ = -127.2 (*c* 1.06, $CHCl_3$); ^{1}H NMR (400 MHz, $CDCl_3$) δ 8.28 (d, *J* = 8.4 Hz, 2H), 7.98 (d, *J* = 8.4 Hz, 2H), 7.27 – 7.18 (m, 3H), 7.13 (d, *J* = 6.6 Hz, 1H), 6.50 (d, *J* = 15.7 Hz, 1H), 5.95 (dd, *J* = 15.7, 7.2 Hz, 1H), 4.53 – 4.32 (m, 1H), 3.58 – 3.38 (m, 2H), 2.03 – 1.90 (m, 2H), 1.88 – 1.74 (m, 2H); ^{13}C NMR (100 MHz, $CDCl_3$) δ 149.9, 144.8, 137.9, 134.6, 130.4, 130.3, 129.8, 128.5, 127.9, 126.2, 124.7, 124.2, 61.9, 48.7, 32.9, 24.1. HRMS (ESI) calculated for $C_{18}H_{17}ClN_2NaO_4S$ [(M+Na)$^+$]: 415.0490, found: 415.0487. HPLC (Daicel Chiralpak AD-H column, *n*-hexane/isopropanol = 90/10, flow rate 1.0 mL/min, 254 nm): t_R (major) = 23.4 min, t_R (minor) = 29.2 min.

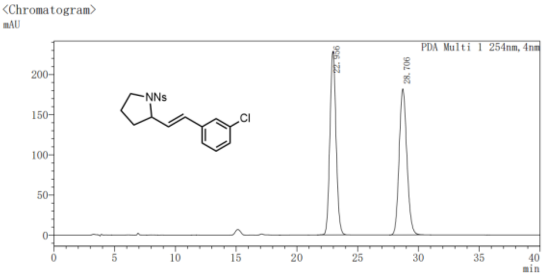

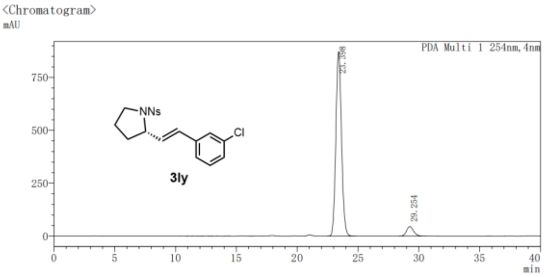

<Peak Table>
PDA Ch1 254nm

No.	Ret. Time (min)	Height (uAU)	Height%	Area (uAU*min)	Area%
1	22.956	228238	55.682	8044642	50.056
2	28.706	181659	44.318	8026742	49.944
Total		409897	100.000	16071385	100.000

<Peak Table>
PDA Ch1 254nm

No.	Ret. Time (min)	Height (uAU)	Height%	Area (uAU*min)	Area%
1	23.398	871846	95.070	28182313	93.838
2	29.254	45207	4.930	1850745	6.162
Total		917052	100.000	30033058	100.000

(S,E)-1-benzyl-2-styrylpiperidine (5aa):

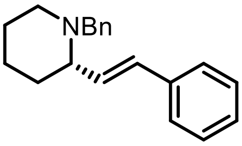

White solid (63.9 mg, 77% yield), 95:5 *er*. m.p. 63-64 ºC; $[\alpha]_D^{20}$ = -152.9 (*c* 0.64, $CHCl_3$); ^{1}H NMR (400 MHz, $CDCl_3$) δ 7.41 (d, *J* = 7.9 Hz, 2H), 7.38 – 7.27 (m, 6H), 7.27 – 7.19 (m, 2H), 6.58 (d,

J = 16.0 Hz, 1H), 6.32 (dd, J = 15.9, 8.6 Hz, 1H), 4.13 (d, J = 13.6 Hz, 1H), 3.15 (d, J = 13.6 Hz, 1H), 2.88 (t, J = 10.5 Hz, 2H), 1.98 (td, J = 11.2, 2.0 Hz, 1H), 1.84 – 1.71 (m, 2H), 1.66 – 1.49 (m, 3H), 1.43 – 1.31 (m, 1H); ^{13}C NMR (100 MHz, $CDCl_3$) δ 139.4, 137.2, 134.1, 130.7, 129.0, 128.5, 128.0, 127.3, 126.6, 126.2, 66.0, 60.2, 52.3, 33.8, 25.8, 24.0. HRMS (ESI) calculated for $C_{20}H_{24}N$ [$(M+H)^+$]: 278.1903, found: 278.1908. HPLC (Daicel Chiralpak AD-H column, *n*-hexane/isopropanol = 90/10, flow rate 1.0 mL/min, 254 nm): t_R (major) = 3.6 min, t_R (minor) = 4.2 min.

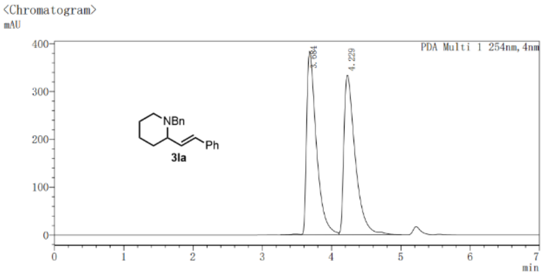

<Peak Table>
PDA Ch1 254nm

No.	Ret. Time (min)	Height (uAU)	Height%	Area (uAU*min)	Area%
1	3.684	383481	53.492	3776574	49.954
2	4.229	333418	46.508	3783499	50.046
Total		716899	100.000	7560073	100.000

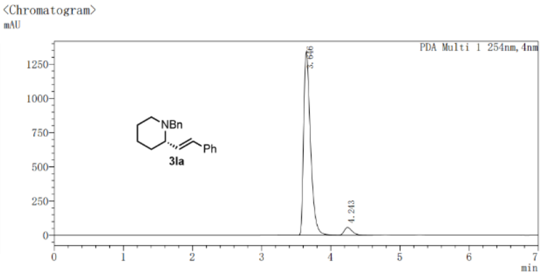

<Peak Table>
PDA Ch1 254nm

No.	Ret. Time (min)	Height (uAU)	Height%	Area (uAU*min)	Area%
1	3.646	1342032	95.927	9255088	95.138
2	4.243	56976	4.073	472937	4.862
Total		1399008	100.000	9728025	100.000

(*S,E*)-1-(4-methoxybenzyl)-2-styrylpiperidine (5ba):

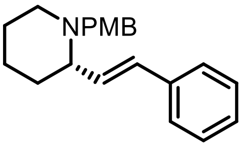

White solid (77.6 mg, 84% yield), 94:6 *er*. m.p. 85-86 ºC; $[\alpha]_D^{20}$ = -109.4 (*c* 1.08, $CHCl_3$); ^{1}H NMR (400 MHz, $CDCl_3$) δ 7.41 (d, J = 7.6 Hz, 2H), 7.38 – 7.29 (m, 2H), 7.29 – 7.19 (m, 3H), 6.90 – 6.81 (m, 2H), 6.57 (d, J = 16.0 Hz, 1H), 6.32 (dd, J = 16.1, 8.8 Hz, 1H), 4.04 (d, J = 13.4 Hz, 1H), 3.81 (s, 3H), 3.12 (d, J = 13.4 Hz, 1H), 2.94 – 2.80 (m, 2H), 2.02 – 1.89 (m, 1H), 1.82 – 1.68 (m, 2H), 1.66 – 1.47 (m, 3H), 1.42 – 1.30 (m, 1H); ^{13}C NMR (100 MHz, $CDCl_3$) δ 158.4, 137.2, 134.1, 131.1, 130.7, 130.2, 128.5, 127.2, 126.2, 113.4, 65.9, 59.5, 55.2, 52.1, 33.7, 25.8, 23.9. HRMS (ESI) calculated for $C_{21}H_{26}NO$ [$(M+H)^+$]: 308.2009, found: 308.2011. HPLC (Daicel Chiralpak OD-H column, *n*-hexane/isopropanol = 95/5, flow rate 0.5 mL/min, 254 nm): t_R (minor) = 9.5 min, t_R (major) = 11.2 min.

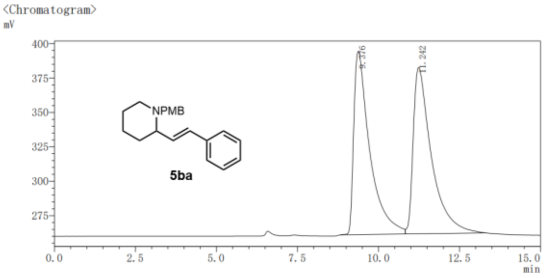

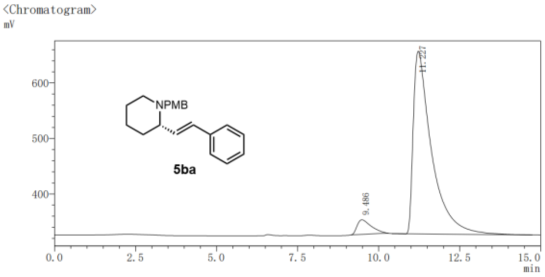

<Peak Table>
检测器A Ch2 254nm

No.	Ret. Time (min)	Height (uAU)	Height%	Area (uAU*min)	Area%
1	9.376	133133	52.415	4590413	49.236
2	11.242	120867	47.585	4732897	50.764
Total		254000	100.000	9323310	100.000

<Peak Table>
检测器A Ch2 254nm

No.	Ret. Time (min)	Height (uAU)	Height%	Area (uAU*min)	Area%
1	9.486	26285	7.396	802017	5.829
2	11.227	329127	92.604	12957058	94.171
Total		355412	100.000	13759075	100.000

(*S,E*)-2-(4-(benzyloxy)styryl)-1-(3-methylbenzyl)piperidine (5cz):

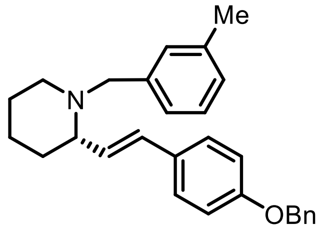

Yellow solid (101.6 mg, 85% yield), 93.5:6.5 *er*. m.p. 73-74 ºC; $[\alpha]_D^{20}$ = -81.7 (*c* 0.72, $CHCl_3$); ^1H NMR (400 MHz, $CDCl_3$) δ 7.46 – 7.41 (m, 2H), 7.41 – 7.36 (m, 2H), 7.36 – 7.28 (m, 3H), 7.22 – 7.15 (m, 1H), 7.13 (s, 1H), 7.11 (d, *J* = 7.8 Hz, 1H), 7.04 (d, *J* = 7.4 Hz, 1H), 6.96 – 6.90 (m, 2H), 6.48 (d, *J* = 15.9 Hz, 1H), 6.15 (dd, *J* = 15.9, 8.6 Hz, 1H), 5.08 (s, 2H), 4.07 (d, *J* = 13.5 Hz, 1H), 3.06 (d, *J* = 13.5 Hz, 1H), 2.92 – 2.84 (m, 1H), 2.85 – 2.74 (m, 1H), 2.34 (s, 3H), 1.98 – 1.88 (m, 1H), 1.79 – 1.69 (m, 3H), 1.63 – 1.49 (m, 3H); ^{13}C NMR (100 MHz, $CDCl_3$) δ 158.2, 139.3, 138.1, 137.6, 137.0, 132.1, 130.4, 130.2, 129.9, 128.6, 128.0, 127.9, 127.4, 127.4, 126.3, 115.0, 70.1, 66.3, 60.3, 52.5, 33.9, 25.9, 24.1, 21.4. HRMS (ESI) calculated for $C_{28}H_{32}NO$ $[(M+H)^+]$: 398.2478, found: 398.2486. HPLC (Daicel Chiralpak OD-H column, *n*-hexane/isopropanol = 95/5, flow rate 0.5 mL/min, 254 nm): t_R (major) = 11.9 min, t_R (minor) = 13.7 min.

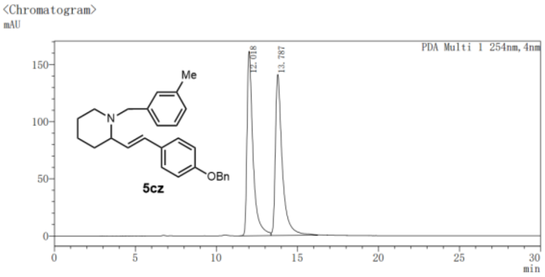

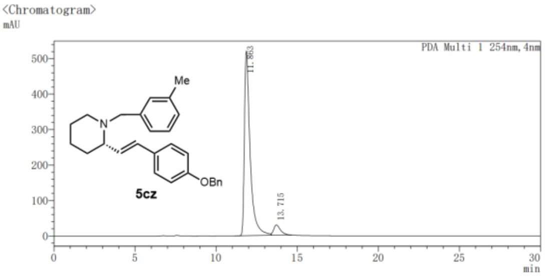

<Peak Table>
PDA Ch1 254nm

No.	Ret. Time (min)	Height (uAU)	Height%	Area (uAU*min)	Area%
1	12.018	161419	53.369	4157987	49.860
2	13.787	141038	46.631	4181257	50.140
Total		302457	100.000	8339244	100.000

<Peak Table>
PDA Ch1 254nm

No.	Ret. Time (min)	Height (uAU)	Height%	Area (uAU*min)	Area%
1	11.863	520106	94.655	12757287	93.663
2	13.715	29370	5.345	863151	6.337
Total		549476	100.000	13620437	100.000

(*S,E*)-2-(4-(benzyloxy)styryl)-1-(naphthalen-1-ylmethyl)piperidine (5dz):

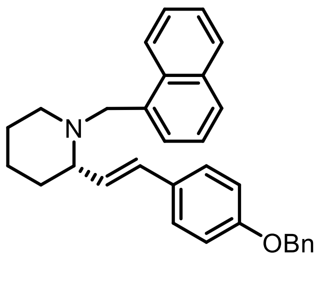

Yellow oil (100.7 mg, 77% yield), 95:5 *er*. $[\alpha]_D^{20}$ = -84.7 (*c* 1.09, $CHCl_3$); ^1H NMR (400 MHz, $CDCl_3$) δ 8.39 (d, *J* = 7.8 Hz, 1H), 7.87 (d, *J* = 6.9 Hz, 1H), 7.78 (d, *J* = 8.0 Hz, 1H), 7.63 – 7.47 (m, 3H), 7.50 – 7.30 (m, 8H), 7.01 – 6.92 (m, 2H), 6.61 (dd, *J* = 15.9, 3.4 Hz, 1H), 6.35 (ddt, *J* = 15.8, 8.7, 2.5 Hz, 1H), 5.10 (s, 2H), 4.63 (dd, *J* = 13.8, 3.8 Hz, 1H), 3.47 (dd, *J* = 13.7, 3.2 Hz, 1H), 3.05 – 2.91 (m, 1H), 2.92 – 2.83 (m, 1H), 2.08 – 1.93 (m, 1H), 1.90 – 1.73 (m, 2H), 1.74 – 1.63 (m, 1H), 1.62 – 1.34 (m, 3H); ^{13}C NMR (100 MHz, $CDCl_3$) δ 158.3, 137.1, 135.6, 133.9, 132.6, 131.7, 130.5, 130.4, 128.6, 128.5, 128.0, 127.5, 127.4, 127.2, 125.6, 125.5, 125.3, 124.8, 115.1, 70.1, 67.2, 58.5, 52.6, 33.9, 26.0, 24.1. HRMS (ESI) calculated for $C_{31}H_{32}NO$ $[(M+H)^+]$: 434.2478, found: 434.2492. HPLC (Daicel Chiralpak OD-H column, *n*-hexane/isopropanol = 90/10, flow rate 1.0 mL/min, 254 nm): t_R (major) = 6.8 min, t_R (minor) = 9.8 min.

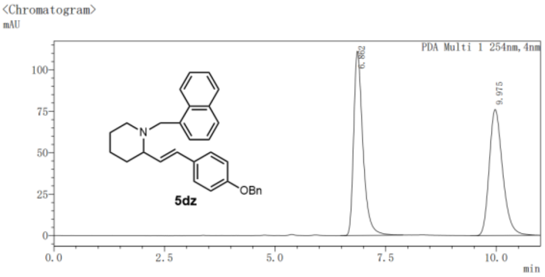

<Peak Table>
PDA Ch1 254nm

No.	Ret.Time(min)	Height(uAU)	Height%	Area(uAU*min)	Area%
1	6.862	111064	59.351	1616499	50.032
2	9.975	76066	40.649	1614437	49.968
Total		187130	100.000	3230936	100.000

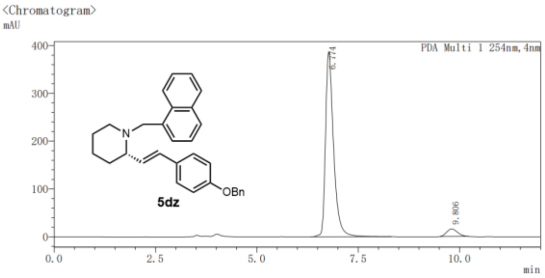

<Peak Table>
PDA Ch1 254nm

No.	Ret.Time(min)	Height(uAU)	Height%	Area(uAU*min)	Area%
1	6.774	386654	96.129	5366940	94.910
2	9.806	15571	3.871	287843	5.090
Total		402225	100.000	5654783	100.000

(*S,E*)-1-((4-nitrophenyl)sulfonyl)-2-styrylpiperidine (5ea):

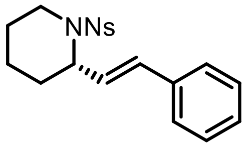

Purified by chromatography on silica gel, eluting with PE/EA/DCM = 20/1/1; yellow solid (90.3 mg, 81% yield), 93.5:6.5 *er*. m.p. 123-124 °C; $[\alpha]_D^{20}$ = -76.7 (*c* 1.07, $CHCl_3$); ^1H NMR (400 MHz, $CDCl_3$) δ 8.23 – 8.15 (m, 2H), 7.98 – 7.90 (m, 2H), 7.30 – 7.25 (m, 1H), 7.25 – 7.19 (m, 2H), 7.17 – 7.12 (m, 2H), 6.41 (d, *J* = 16.3 Hz, 1H), 5.98 (dd, *J* = 16.0, 6.9 Hz, 1H), 4.90 – 4.70 (m, 1H), 3.89 – 3.72 (m, 1H), 3.17 – 2.95 (m, 1H), 1.88 – 1.76 (m, 2H), 1.74 – 1.66 (m, 1H), 1.66 – 1.59 (m, 1H), 1.59 – 1.47 (m, 2H); ^{13}C NMR (100 MHz, $CDCl_3$) δ 149.5, 146.1, 135.8, 132.9, 128.6, 128.5, 128.0, 126.1, 124.9, 124.0, 55.7, 42.2, 30.7, 25.1, 18.9. HRMS (ESI) calculated for $C_{19}H_{20}N_2NaO_4S$

[(M+Na)$^+$]: 395.1036, found: 395.1034. HPLC (Daicel Chiralpak OD-H column, *n*-hexane/isopropanol = 90/10, flow rate 1.0 mL/min, 254 nm): t_R (major) = 22.5 min, t_R (minor) = 29.5 min.

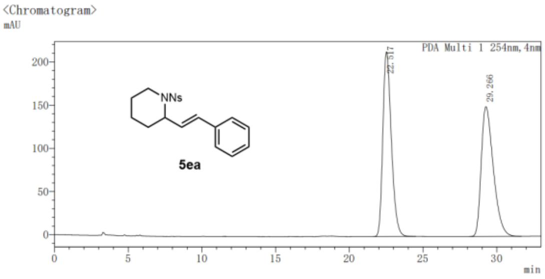

<Peak Table>
PDA Ch1 254nm

No.	Ret. Time(min)	Height(uAU)	Height%	Area(uAU*min)	Area%
1	22.517	213866	58.727	8588411	50.082
2	29.266	150303	41.273	8560156	49.918
Total		364169	100.000	17148568	100.000

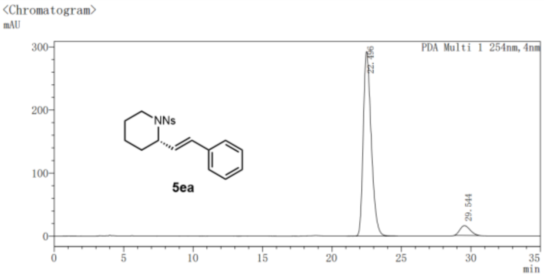

<Peak Table>
PDA Ch1 254nm

No.	Ret. Time(min)	Height(uAU)	Height%	Area(uAU*min)	Area%
1	22.496	292475	94.949	11710632	93.632
2	29.544	15559	5.051	796417	6.368
Total		308034	100.000	12507049	100.000

(*S,E*)-1-benzyl-2-(4-bromostyryl)piperidine (5ab):

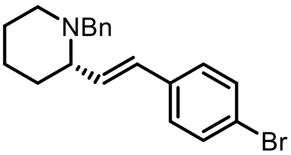

Yellow oil (95.2 mg, 89% yield), 92:8 *er*. $[\alpha]_D^{20}$ = -89.7 (*c* 1.22, $CHCl_3$); ^1H NMR (400 MHz, $CDCl_3$) δ 7.46 – 7.44 (m, 1H), 7.43 – 7.41 (m, 1H), 7.36 – 7.29 (m, 4H), 7.27 – 7.20 (m, 3H), 6.49 (d, *J* = 15.9 Hz, 1H), 6.28 (dd, *J* = 16.0, 8.5 Hz, 1H), 4.07 (d, *J* = 13.6 Hz, 1H), 3.13 (d, *J* = 13.6 Hz, 1H), 2.93 – 2.79 (m, 2H), 1.96 (td, *J* = 11.3, 3.2 Hz, 1H), 1.82 – 1.67 (m, 2H), 1.65 – 1.50 (m, 3H), 1.43 – 1.32 (m, 1H); ^{13}C NMR (100 MHz, $CDCl_3$) δ 139.4, 136.2, 135.0, 131. 6, 129.5, 128.9, 128.0, 127.7, 126.6, 120.9, 66.0, 60.3, 52.4, 33.7, 25.8, 23.9. HRMS (ESI) calculated for $C_{20}H_{23}BrN$ [(M+H)$^+$]: 356.1008, found: 356.1010. HPLC (Daicel Chiralpak AD-H column, *n*-hexane/isopropanol = 90/10, flow rate 1.0 mL/min, 254 nm): t_R (major) = 4.2 min, t_R (minor) = 5.0 min.

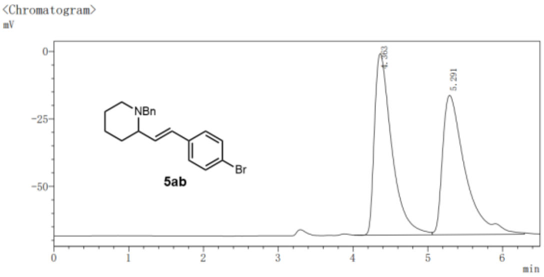

<Peak Table>
检测器A Ch2 254nm

No.	Ret. Time(min)	Height(uAU)	Height%	Area(uAU*min)	Area%
1	4.363	67208	56.536	1079564	49.817
2	5.291	51669	43.464	1087490	50.183
Total		118877	100.000	2167055	100.000

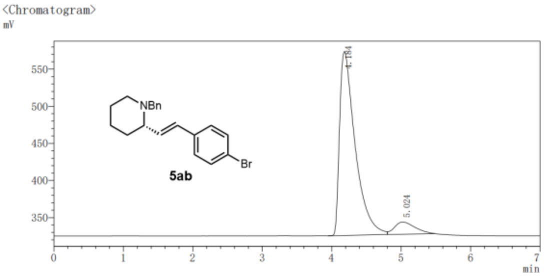

<Peak Table>
检测器A Ch2 254nm

No.	Ret. Time(min)	Height(uAU)	Height%	Area(uAU*min)	Area%
1	4.184	247864	93.750	3866902	91.937
2	5.024	16523	6.250	339145	8.063
Total		264387	100.000	4206048	100.000

(*S,E*)-1-benzyl-2-(4-phenoxystyryl)piperidine (5ae):

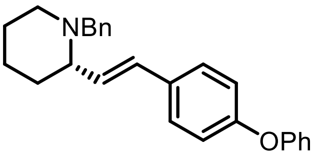

Yellow solid (99.8 mg, 90% yield), 95:5 *er*. m.p. 78-79 ºC; $[\alpha]_D^{20}$ = -98.9 (*c* 1.20, $CHCl_3$); ^{1}H NMR (400 MHz, $CDCl_3$) δ 7.42 – 7.29 (m, 8H), 7.29 – 7.21 (m, 1H), 7.16 – 7.09 (m, 1H), 7.06 – 7.02 (m, 2H), 7.02 – 6.96 (m, 2H), 6.55 (d, *J* = 15.9 Hz, 1H), 6.24 (dd, *J* = 16.0, 8.6 Hz, 1H), 4.14 (d, *J* = 13.6 Hz, 1H), 3.15 (d, *J* = 13.6 Hz, 1H), 2.98 – 2.80 (m, 2H), 1.98 (td, *J* = 11.2, 3.3 Hz, 1H), 1.84 – 1.71 (m, 2H), 1.68 – 1.51 (m, 3H), 1.46 – 1.32 (m, 1H); ^{13}C NMR (100 MHz, $CDCl_3$) δ 157.2, 156.4, 139.4, 133.2, 132.5, 129.9, 129.7, 129.0, 128.0, 127.5, 126.6, 123.1, 119.0, 118.7, 66.1, 60.2, 52.3, 33.8, 25.8, 24.0. HRMS (ESI) calculated for $C_{26}H_{28}NO$ [$(M+H)^+$]: 370.2165, found: 370.2173. HPLC (Daicel Chiralpak AD-H column, *n*-hexane/isopropanol = 90/10, flow rate 1.0 mL/min, 254 nm): t_R (major) = 4.5 min, t_R (minor) = 7.0 min.

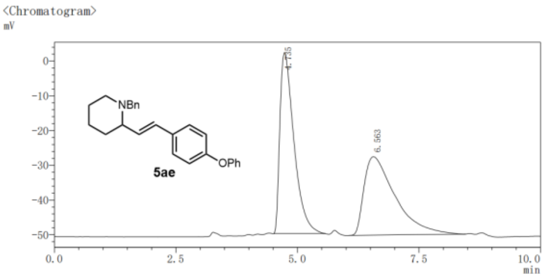

<Peak Table>
检測器A Ch2 254nm

No.	Ret.Time(min)	Height(uAU)	Height%	Area(uAU*min)	Area%
1	4.735	52074	69.767	1051094	51.020
2	6.563	22566	30.233	1009067	48.980
Total		74640	100.000	2060161	100.000

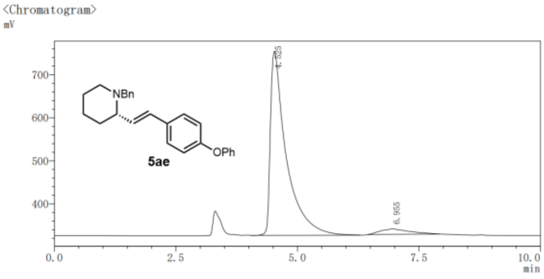

<Peak Table>
检測器A Ch2 254nm

No.	Ret.Time(min)	Height(uAU)	Height%	Area(uAU*min)	Area%
1	4.525	427201	97.192	9714355	94.892
2	6.955	12342	2.808	522935	5.108
Total		439543	100.000	10237290	100.000

(*S,E*)-1-benzyl-2-(4-(methylthio)styryl)piperidine (5af):

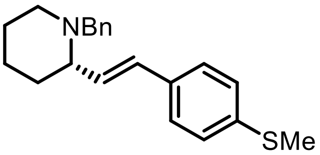

Yellow solid (76.8 mg, 79% yield), 94.5:5.5 *er*. m.p. 84-85 ºC; $[\alpha]_D^{20}$ = -105.0 (*c* 0.94, $CHCl_3$); ^{1}H NMR (400 MHz, $CDCl_3$) δ 7.35 – 7.27 (m, 6H), 7.25 – 7.18 (m, 3H), 6.50 (d, *J* = 16.0 Hz, 1H), 6.25 (dd, *J* = 15.9, 8.6 Hz, 1H), 4.09 (d, *J* = 13.6 Hz, 1H), 3.12 (d, *J* = 13.6 Hz, 1H), 2.97 – 2.78 (m, 2H), 2.48 (s, 3H), 1.95 (td, *J* = 11.3, 3.2 Hz, 1H), 1.81 – 1.67 (m, 2H), 1.65 – 1.45 (m, 3H), 1.42 – 1.29 (m, 1H); ^{13}C NMR (100 MHz, $CDCl_3$) δ 139.4, 137.3, 134.3, 133.7, 130.1, 129.0, 128.0, 126.8, 126.6 (overlap), 66.1, 60.2, 52.4, 33.8, 25.8, 24.0, 16.0. HRMS (ESI) calculated for $C_{21}H_{26}NS$ [$(M+H)^+$]: 324.1780, found: 324.1780. HPLC (Daicel Chiralpak AD-H column, *n*-hexane/isopropanol = 90/10, flow rate 1.0 mL/min, 254 nm): t_R (major) = 4.7 min, t_R (minor) = 7.1 min.

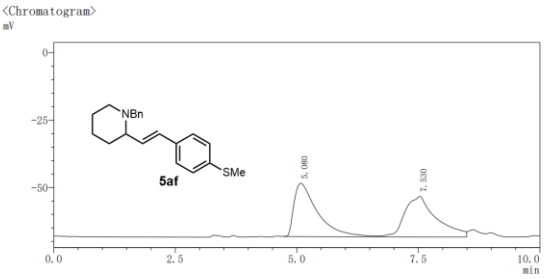

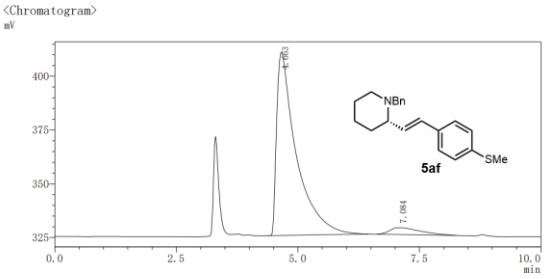

<Peak Table>
检测器A Ch2 254nm

No.	Ret. Time(min)	Height(uAU)	Height%	Area(uAU*min)	Area%
1	5. 080	19754	56. 779	662778	49. 558
2	7. 530	15037	43. 221	674609	50. 442
Total		34791	100. 000	1337387	100. 000

<Peak Table>
检测器A Ch2 254nm

No.	Ret. Time(min)	Height(uAU)	Height%	Area(uAU*min)	Area%
1	4. 663	85214	96. 441	2393485	94. 436
2	7. 084	3144	3. 559	141022	5. 564
Total		88359	100. 000	2534507	100. 000

(*S,E*)-2-(2-([1,1'-biphenyl]-4-yl)vinyl)-1-benzylpiperidine (5ag):

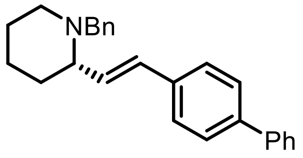

Yellow solid (63.2 mg, 60% yield), 95.5:4.5 *er*. m.p. 110-111 ºC; $[\alpha]_D^{20}$ = -116.7 (*c* 0.59, $CHCl_3$); ^{1}H NMR (400 MHz, $CDCl_3$) δ 7.62 – 7.58 (m, 2H), 7.58 – 7.54 (m, 2H), 7.50 – 7.41 (m, 4H), 7.38 – 7.28 (m, 5H), 7.26 – 7.20 (m, 1H), 6.59 (d, *J* = 16.0 Hz, 1H), 6.35 (dd, *J* = 16.0, 8.6 Hz, 1H), 4.12 (d, *J* = 13.6 Hz, 1H), 3.15 (d, *J* = 13.6 Hz, 1H), 3.00 – 2.79 (m, 2H), 1.97 (td, *J* = 11.2, 3.3 Hz, 1H), 1.83 – 1.68 (m, 2H), 1.68 – 1.47 (m, 3H), 1.43 – 1.31 (m, 1H); ^{13}C NMR (100 MHz, $CDCl_3$) δ 140.8, 140.1, 139.4, 136.3, 134.3, 130.3, 129.1, 128.8, 128.0, 127.2, 126.9, 126.6 (overlap), 66.1, 60.3, 52.4, 33.8 25.8, 24.0. HRMS (ESI) calculated for $C_{26}H_{28}N$ $[(M+H)^+]$: 354.2216, found: 354.2216. HPLC (Daicel Chiralpak AD-H column, *n*-hexane/isopropanol = 90/10, flow rate 1.0 mL/min, 254 nm): t_R (major) = 5.0 min, t_R (minor) = 7.8 min.

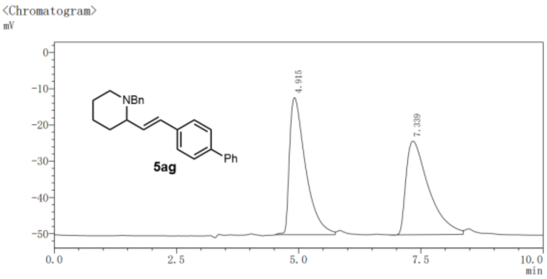

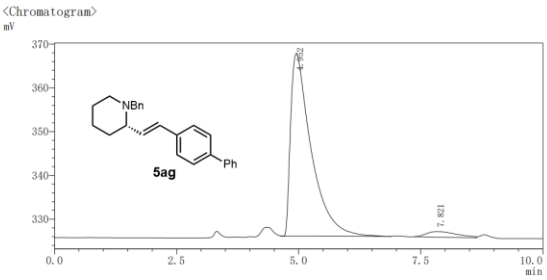

<Peak Table>
检测器A Ch2 254nm

No.	Ret. Time(min)	Height(uAU)	Height%	Area(uAU*min)	Area%
1	4. 915	37823	59. 418	868813	50. 847
2	7. 339	25832	40. 582	839872	49. 153
Total		63655	100. 000	1708685	100. 000

<Peak Table>
检测器A Ch2 254nm

No.	Ret. Time(min)	Height(uAU)	Height%	Area(uAU*min)	Area%
1	4. 952	41766	97. 047	1226783	95. 689
2	7. 821	1271	2. 953	55271	4. 311
Total		43037	100. 000	1282054	100. 000

(*S,E*)-1-benzyl-2-(3,4,5-trimethoxystyryl)piperidine (5ap):

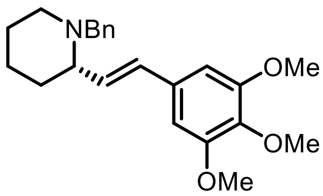

Yellow solid (93.3 mg, 85% yield), 94.5:5.5 *er*. m.p. 54-55 ºC; $[\alpha]_D^{20}$ = -92.3 (*c* 1.18, $CHCl_3$); ^{1}H NMR (400 MHz, $CDCl_3$) δ 7.36 – 7.27 (m, 4H), 7.25 – 7.19 (m, 1H), 6.61 (s,

2H), 6.48 (d, J = 15.9 Hz, 1H), 6.19 (dd, J = 15.8, 8.6 Hz, 1H), 4.10 (d, J = 13.7 Hz, 1H), 3.88 (s, 6H), 3.85 (s, 3H), 3.14 (d, J = 13.7 Hz, 1H), 2.94 – 2.77 (m, 2H), 1.95 (td, J = 11.3, 3.1 Hz, 1H), 1.83 – 1.68 (m, 2H), 1.67 – 1.49 (m, 3H), 1.41 – 1.29 (m, 1H); ^{13}C NMR (100 MHz, $CDCl_3$) δ 153.2, 139.3, 137.6, 133.6, 132.9, 130.5, 128.9, 127.9, 126.5, 103.2, 66.1, 60.8, 60.2, 56.0, 52.4, 33.8, 25.8, 23.9. HRMS (ESI) calculated for $C_{23}H_{30}NO_3$ [$(M+H)^+$]: 368.2220, found: 368.2223. HPLC (Daicel Chiralpak AD-H column, *n*-hexane/isopropanol = 90/10, flow rate 1.0 mL/min, 254 nm): t_R (major) = 6.3 min, t_R (minor) = 11.0 min.

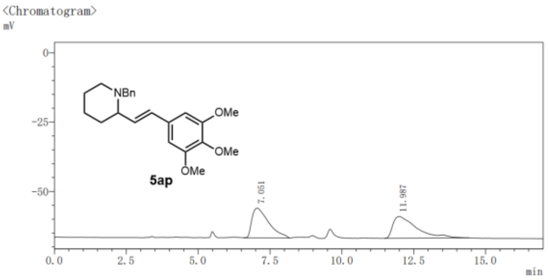

<Peak Table>
検測器A Ch2 254nm

No.	Ret. Time (min)	Height (uAU)	Height%	Area (uAU*min)	Area%
1	7.051	10763	57.968	436464	49.256
2	11.987	7805	42.032	449658	50.744
Total		18568	100.000	886122	100.000

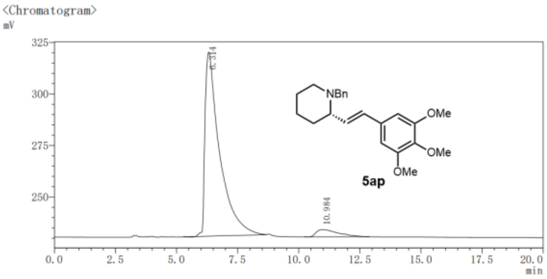

<Peak Table>
検測器A Ch2 254nm

No.	Ret. Time (min)	Height (uAU)	Height%	Area (uAU*min)	Area%
1	6.314	89323	96.190	3720770	94.427
2	10.984	3538	3.810	219593	5.573
Total		92861	100.000	3940363	100.000

(*S,E*)-1-benzyl-2-(2-(naphthalen-1-yl)vinyl)piperidine (5ar):

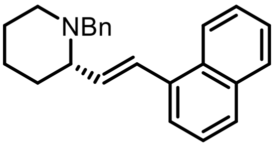

Yellow oil (49.6 mg, 51% yield), 95:5 *er*. $[\alpha]_D^{20}$ = -58.5 (*c* 1.22, $CHCl_3$); ^{1}H NMR (400 MHz, $CDCl_3$) δ 8.16 (d, J = 7.9 Hz, 1H), 7.88 (d, J = 8.6 Hz, 1H), 7.80 (d, J = 8.2 Hz, 1H), 7.62 (d, J = 7.1 Hz, 1H), 7.58 – 7.44 (m, 3H), 7.43 – 7.38 (m, 2H), 7.38 – 7.31 (m, 3H), 7.31 – 7.26 (m, 1H), 6.38 (ddd, J = 15.8, 8.7, 1.5 Hz, 1H), 4.23 (d, J = 13.5 Hz, 1H), 3.26 (d, J = 13.6 Hz, 1H), 3.12 – 3.00 (m, 1H), 2.98 – 2.88 (m, 1H), 2.06 (td, J = 11.1, 3.3 Hz, 1H), 1.90 – 1.79 (m, 2H), 1.76 – 1.54 (m, 3H), 1.51 – 1.38 (m, 1H); ^{13}C NMR (100 MHz, $CDCl_3$) δ 139.4, 137.3, 135.0, 133.6, 131.1, 129.1, 128.5, 128.1, 127.9, 127.6, 126.6, 125.9, 125.7, 125.6, 123.8 (overlap), 66.2, 60.3, 52.4, 33.9, 25.9, 24.0. HRMS (ESI) calculated for $C_{24}H_{26}N$ [$(M+H)^+$]: 328.2060, found: 328.2055. HPLC (Daicel Chiralpak OD-H column, *n*-hexane/isopropanol = 90/10, flow rate 1.0 mL/min, 254 nm): t_R (minor) = 4.6 min, t_R (major) = 4.9 min.

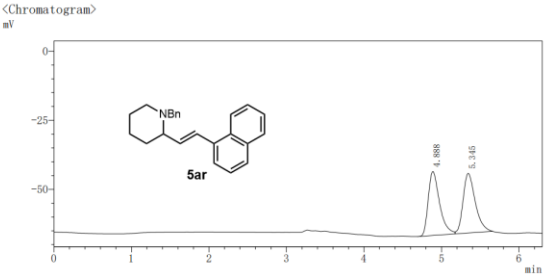

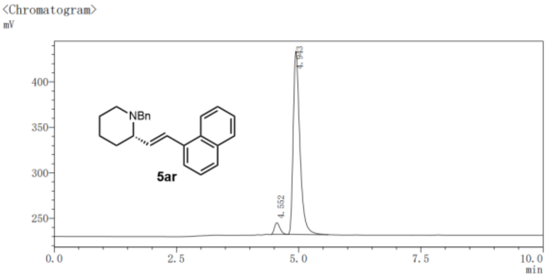

<Peak Table>
检测器A Ch2 254nm

No.	Ret. Time(min)	Height(uAU)	Height%	Area(uAU*min)	Area%
1	4.888	23149	51.767	235060	49.836
2	5.345	21569	48.233	236608	50.164
Total		44717	100.000	471668	100.000

<Peak Table>
检测器A Ch2 254nm

No.	Ret. Time(min)	Height(uAU)	Height%	Area(uAU*min)	Area%
1	4.552	12732	5.953	100599	4.990
2	4.943	201130	94.047	1915293	95.010
Total		213862	100.000	2015892	100.000

(*S,E*)-1-benzyl-2-(2-(furan-2-yl)vinyl)piperidine (5at):

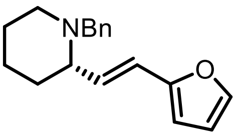

Yellow oil (60.1 mg, 75% yield), 94:6 *er*. $[\alpha]_D^{20}$ = -112.6 (*c* 1.17, $CHCl_3$); ^1H NMR (400 MHz, $CDCl_3$) δ 7.38 – 7.27 (m, 5H), 7.26 – 7.20 (m, 1H), 6.43 – 6.35 (m, 2H), 6.26 (dd, *J* = 16.0, 8.5 Hz, 1H), 6.21 (d, *J* = 3.3 Hz, 1H), 4.10 (d, *J* = 13.5 Hz, 1H), 3.11 (d, *J* = 13.6 Hz, 1H), 2.93 – 2.77 (m, 2H), 1.95 (td, *J* = 11.1, 3.4 Hz, 1H), 1.81 – 1.68 (m, 2H), 1.66 – 1.49 (m, 3H), 1.43 – 1.31 (m, 1H); ^{13}C NMR (100 MHz, $CDCl_3$) δ 152.8, 141.6, 139.4, 132.8, 129.0, 128.0, 126.6, 119.4, 111.1, 107.0, 65.7, 60.2, 52.1, 33.7, 25.8, 23.9. HRMS (ESI) calculated for $C_{18}H_{22}NO$ $[(M+H)^+]$: 268.1696, found: 268.1701. HPLC (Daicel Chiralpak AD-H column, *n*-hexane/isopropanol = 90/10, flow rate 1.0 mL/min, 254 nm): t_R (major) = 3.9 min, t_R (minor) = 5.0 min.

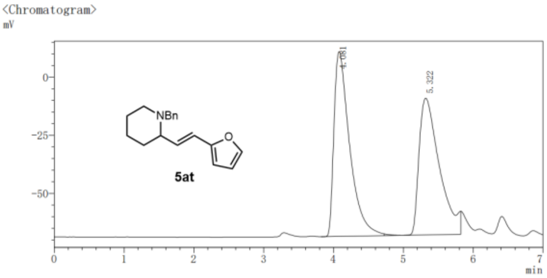

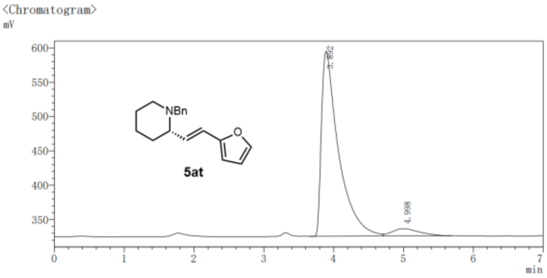

<Peak Table>
检测器A Ch2 254nm

No.	Ret. Time(min)	Height(uAU)	Height%	Area(uAU*min)	Area%
1	4.081	79541	57.476	1244306	51.053
2	5.322	58848	42.524	1192972	48.947
Total		138389	100.000	2437278	100.000

<Peak Table>
检测器A Ch2 254nm

No.	Ret. Time(min)	Height(uAU)	Height%	Area(uAU*min)	Area%
1	3.892	269113	96.248	4594237	94.121
2	4.998	10490	3.752	286980	5.879
Total		279603	100.000	4881218	100.000

(*S,E*)-1-benzyl-2-(2-(thiophen-2-yl)vinyl)piperidine (5aw):

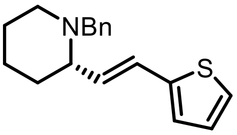

Yellow oil (55.8 mg, 66% yield), 95.5:4.5 *er*. $[\alpha]_D^{20}$ = -122.7 (*c* 0.64, $CHCl_3$); ^1H NMR (400 MHz, $CDCl_3$) δ 7.36 – 7.29 (m, 4H), 7.26 – 7.21 (m, 1H), 7.16 – 7.12 (m, 1H), 7.00 – 6.87 (m, 2H), 6.69 (d, *J* = 15.8 Hz, 1H), 6.15 (dd, *J* = 15.8, 8.6 Hz, 1H), 4.11 (d, *J* = 13.6 Hz, 1H), 3.12 (d,

J = 13.6 Hz, 1H), 2.93 – 2.71 (m, 2H), 1.95 (td, *J* = 11.3, 3.3 Hz, 1H), 1.81 – 1.68 (m, 2H), 1.65 – 1.49 (m, 3H), 1.42 – 1.31 (m, 1H); ^{13}C NMR (100 MHz, $CDCl_3$) δ 142.4, 139.4, 133.8, 129.0, 128.0, 127.2, 126.6, 124.9, 124.0, 123.8, 65.8, 60.2, 52.2, 33.7, 25.8, 23.9. HRMS (ESI) calculated for $C_{18}H_{22}NS$ [$(M+H)^+$]: 284.1467, found: 284.1473. HPLC (Daicel Chiralpak AD-H column, *n*-hexane/isopropanol = 95/5, flow rate 0.5 mL/min, 254 nm): t_R (major) = 8.2 min, t_R (minor) = 11.4 min.

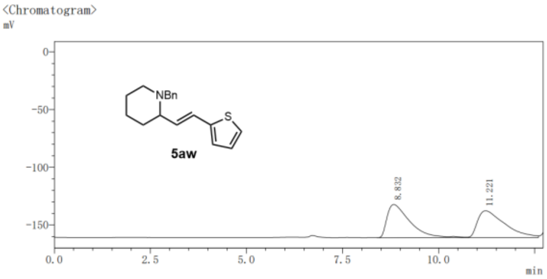

<Peak Table>
检测器A Ch2 254nm

No.	Ret. Time(min)	Height(uAU)	Height%	Area(uAU*min)	Area%
1	8.832	28774	55.261	1186213	50.853
2	11.221	23295	44.739	1146439	49.147
Total		52069	100.000	2332651	100.000

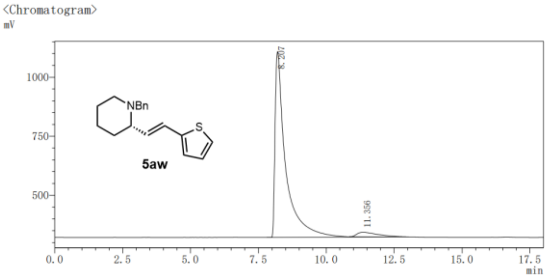

<Peak Table>
检测器A Ch2 254nm

No.	Ret. Time(min)	Height(uAU)	Height%	Area(uAU*min)	Area%
1	8.207	786044	97.648	21400680	95.475
2	11.356	18934	2.352	1014299	4.525
Total		804978	100.000	22414979	100.000

5. Gram-scale Reaction and Synthetic Applications

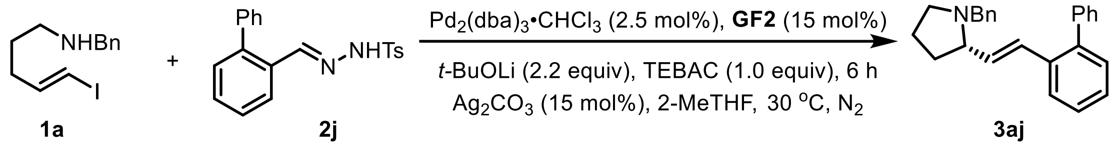

To a 100 mL flame-dried flask was added *N*-tosylhydrazones **2j** (9.6 mmol, 3.36 g), $Pd_2(dba)_3{\cdot}CHCl_3$ (0.15 mmol, 155.3 mg), **GF2** (0.9 mmol, 681.3 mg), *t*-BuOLi (13.2 mmol, 1.06 g), Ag_2CO_3 (0.9 mmol, 248.2 mg), and triethylbenzylammonium chloride (TEBAC, 6 mmol, 1.37 g) was added in the glove box. The reaction was evacuated and back-filled with N_2 three times and a solution of the vinyl iodide **1a** (6 mmol, 1.81 g) in 60 mL 2- MeTHF was added under nitrogen atmosphere. Then the reaction was stirred at 30 °C for 6 h. Upon completion, the reaction mixture was filtered through celite, evaporated *in vacuo* and purified by silica gel column chromatography (DCM to PE/Et_3N = 50:1) to give the product **3aj** (1.74 g, 85% yield, 95.5:4.5 *er*).

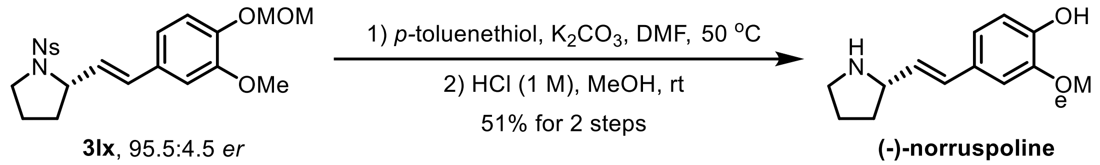

According to published literature,[5, 6] to a round-bottom flask was added **3lx** (0.2 mmol, 89.6 mg), *p*-toluenethiol (0.3 mmol, 37.3 mg) and K_2CO_3 (0.6 mmol, 82.9 mg) in DMF (2 mL). The reaction mixture was stirred at 50 °C overnight, then cooled to room temperature, diluted with water and extracted with EtOAc. The organic layer was dried over Na_2SO_4 and concentrated *in vacuo*. The residue was used in the next step without any further purification.

To a solution of the residue in MeOH (2 mL) at room temperature was added 1 M aqueous HCl (4 mL). The reaction mixture was stirred for 8 h, then added the saturated aqueous $NaHCO_3$ to basify the mixture to pH ≥ 7 and extracted with EtOAc. The organic layer was dried over Na_2SO_4 and concentrated *in vacuo*. Purification by thin-layer chromatography (DCM/MeOH = 3/1) gave (-)-norruspoline (22.2 mg, 51% yield) as an orange solid. m.p. 69-70 °C; $[\alpha]_D^{20}$ = -29.1 (*c* 0.76, $CHCl_3$); ^1H NMR (400 MHz, $CDCl_3$) δ 6.87 (s, 1H), 6.82 (s, 2H), 6.41 (d, *J* = 15.7 Hz, 1H), 6.03 (dd, *J* = 15.7, 7.3

Hz, 1H), 3.86 (s, 3H), 3.68 (q, *J* = 7.4 Hz, 1H), 3.16 – 3.05 (m, 1H), 2.99 – 2.89 (m, 1H), 2.09 – 1.93 (m, 1H), 1.92 – 1.76 (m, 2H), 1.64 – 1.50 (m, 1H); ^{13}C NMR (100 MHz, $CDCl_3$) δ 147.0, 145.7, 130.2, 129.4, 129.3, 120.0, 114.8, 108.3, 61.0, 55.8, 46.2, 32.3, 25.2. HRMS (ESI) calculated for $C_{13}H_{18}NO_2$ $[(M+H)^+]$: 220.1332, found: 220.1336.

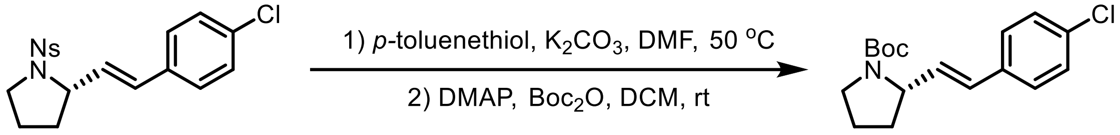

3ly, 94:6 *er* 67% for 2 steps **6**, 93.5:6.5 *er*

According to published literature,[5, 7] to a round-bottom flask was added **3ly** (0.15 mmol, 58.8 mg), *p*-toluenethiol (0.23 mmol, 27.9 mg) and K_2CO_3 (0.45 mmol, 62.2 mg) in DMF (2 mL). The following steps are the same as above to obtain the deprotected crude product.

To a solution of the crude product, 4-(dimethylamino) pyridine (0.015 mmol, 1.8 mg) in DCM (2 mL) at room temperature was added Boc_2O (0.17 mmol, 38 μL). The reaction mixture was stirred for 2 h. Upon completion, the mixture was concentrated *in vacuo* and purified by flash chromatography (PE/EA = 10/1) gave **6** (31.1 mg, 67% yield, 93.5:6.5 *er*) as colorless oil. $[\alpha]_D^{20}$ = -88.1 (*c* 1.03, $CHCl_3$); ^{1}H NMR (400 MHz, $CDCl_3$) δ 7.33 (s, 1H), 7.25 – 7.12 (m, 3H), 6.33 (d, *J* = 15.4 Hz, 1H), 6.20 – 6.02 (m, 1H), 4.73 – 4.26 (m, 2H), 3.45 (s, 2H), 2.09 (s, 1H), 1.99 – 1.68 (m, 3H), 1.42 (s, 9H); ^{13}C NMR (100 MHz, $CDCl_3$) δ 154.4, 138.8, 134.3, 132.2, 129.6, 128.0, 127.0, 126.0, 124.4, 79.1, 58.7, 46.2, 32.4, 28.4, 22.9. All spectroscopic data is in agreement with those previously reported.[8] HRMS (ESI) calculated for $C_{17}H_{22}ClNNaO_2$ $[(M+Na)^+]$: 330.1231, found: 330.1231. HPLC (Daicel Chiralpak AD-H column, *n*-hexane/isopropanol = 90/10, flow rate 1.0 mL/min, 254 nm): t_R (minor) = 4.6 min, t_R (major) = 5.6 min.

<Chromatogram>

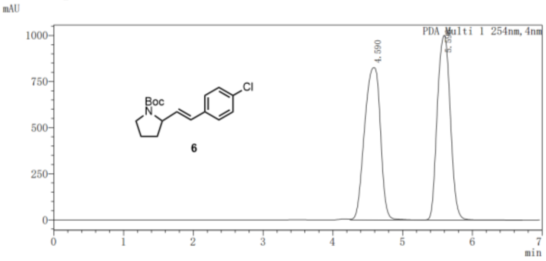

<Peak Table>

PDA Ch1 254nm

No.	Ret.Time(min)	Height(uAU)	Height%	Area(uAU*min)	Area%
1	4.590	826404	45.232	13051970	50.124
2	5.596	1000619	54.768	12987415	49.876
Total		1827023	100.000	26039384	100.000

<Chromatogram>

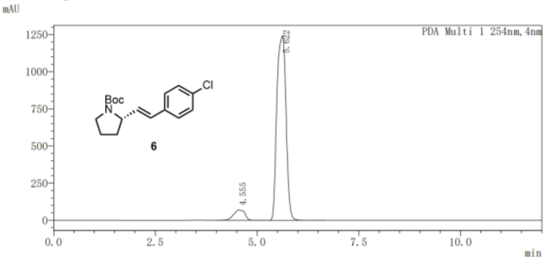

<Peak Table>

PDA Ch1 254nm

No.	Ret.Time(min)	Height(uAU)	Height%	Area(uAU*min)	Area%
1	4.555	69659	5.310	1328620	6.398
2	5.622	1242207	94.690	19436732	93.602
Total		1311866	100.000	20765352	100.000

6. Nonlinear Effect Study

In general, nonlinear effect study began with standard conditions (0.1 mmol), but the ratio of chiral ligands **GF2** and ***ent*-GF2** is various. They were weighed accurately on the analytical balance and the reaction was carried out according to general procedure. The results are listed below.

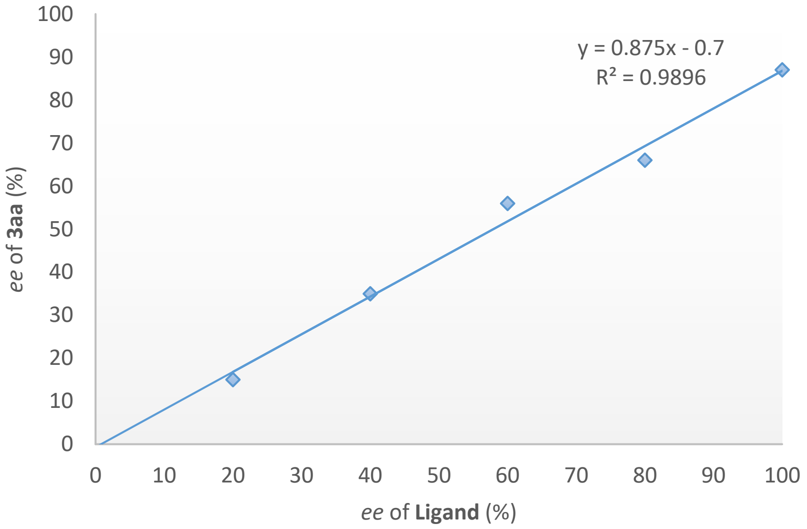

entry	1	2	3	4	5
**GF2/*ent*-GF2** (ratio)	0.6/0.4	0.7/0.3	0.8/0.2	0.9/0.1	1
ee of **Ligand** (%)	20	40	60	80	100
ee of **3aa** (%)	15	35	56	66	87

7. References

1. C.-L. Ma, X.-L. Yu, X.-L. Zhu, Y.-Z. Hu, X.-W. Dong, B. Tan, X.-Y. Liu, *Adv. Synth. Catal.* **2015**, *357*, 569-575.

2. H. Lu, C. Li, H. Jiang, C. L. Lizardi, X. P. Zhang, *Angew. Chem. Int. Ed.* **2014**, *53*, 7028-7032.

3. A. Khanna, C. Maung, K. R. Johnson, T. T. Luong, Van Vranken, D. L. *Org. Lett.* **2012**, *14*, 3233-3235.

4. A.-J. Xia, T.-R. Kang, L. He, L.-M. Chen, W.-T. Li, J.-L. Yang, Q.-Z. Liu, *Angew. Chem. Int. Ed.* **2016**, *55*, 1441-1444.

5. A. Becker, C. P. Grugel, B. Breit, *Org. Lett.* **2021**, *23*, 3788-3792.

6. C. E. Sear, P. Pieper, M. Amaral, M. M. Romanelli, T. A. Costa-Silva, M. M. Haugland, J. A. Tate, J. H. G. Lago, A. G. Tempone, E. A. Anderson, *ACS Infect. Dis.* **2020**, *6*, 2872-2878.

7. N. J. Taylor, E. Emer, S. Preshlock, M. Schedler, M. Tredwell, S. Verhoog, J. Mercier, C. Genicot, V. Gouverneur, *J. Am. Chem. Soc.* **2017**, *139*, 8267-8276.

8. H. Zhang, C. Huang, X.-A. Yuan, S. Yu, *J. Am. Chem. Soc.* **2022**, *144*, 10958-10967.

8. X-ray Single Crystal Data for Compound 3ac

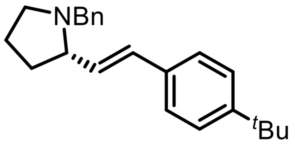

≡

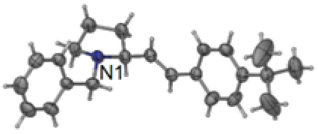

(S)-**3ac** CCDC 2129469

Identification code	exp_2405
Empirical formula	$C_{23}H_{29}N$
Formula weight	319.47
Temperature/K	173.00(10)
Crystal system	monoclinic
Space group	$P2_1$
a/Å	10.9102(3)
b/Å	6.1038(2)
c/Å	14.7452(4)
α/°	90
β/°	99.500(3)
γ/°	90
Volume/Å^3	968.47(5)
Z	2
ρ_{calc}g/cm^3	1.096
μ/mm^{-1}	0.467
F(000)	348.0
Crystal size/mm^3	0.38 × 0.24 × 0.12
Radiation	CuKα (λ = 1.54184)
2Θ range for data collection/°	8.216 to 134.116
Index ranges	$-13 \leq h \leq 13$, $-7 \leq k \leq 7$, $-17 \leq l \leq 17$
Reflections collected	19273
Independent reflections	3408 [R_{int} = 0.0629, R_{sigma} = 0.0387]
Data/restraints/parameters	3408/1/220
Goodness-of-fit on F^2	1.039
Final R indexes [I>=2σ (I)]	R_1 = 0.0465, wR_2 = 0.1281
Final R indexes [all data]	R_1 = 0.0491, wR_2 = 0.1301
Largest diff. peak/hole / e Å^{-3}	0.37/-0.17
Flack parameter	-0.1(4)

9. ^{1}H, ^{13}C, ^{19}F NMR Spectra

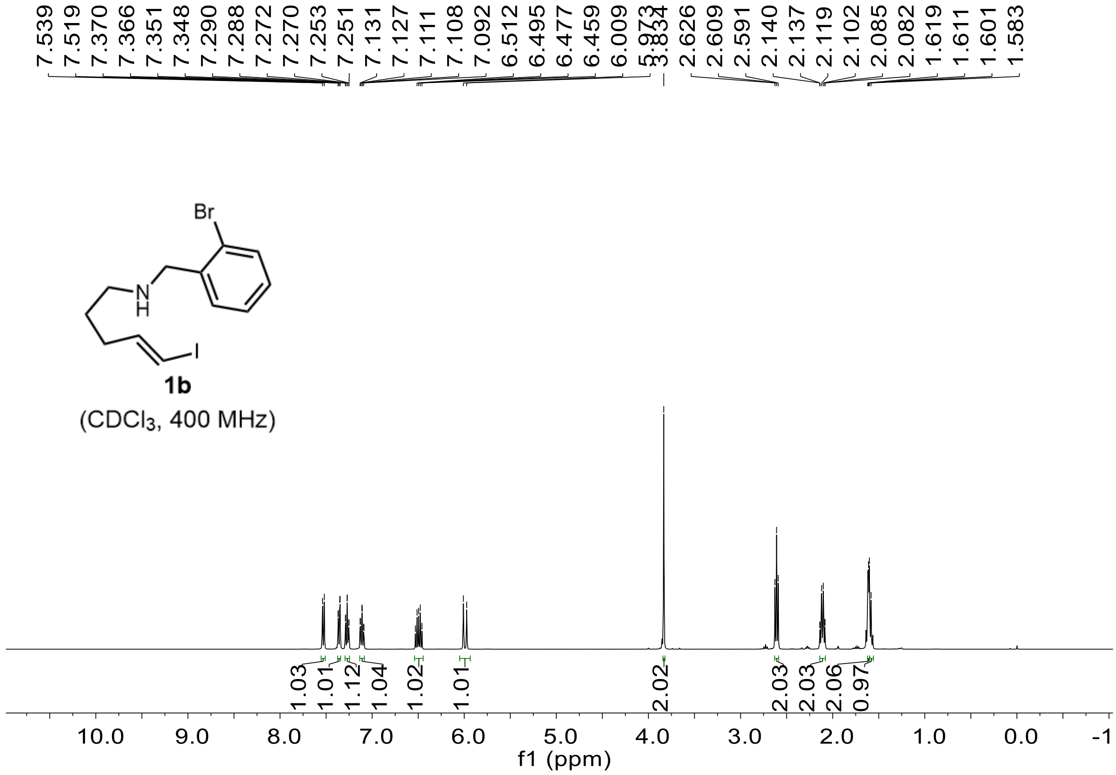

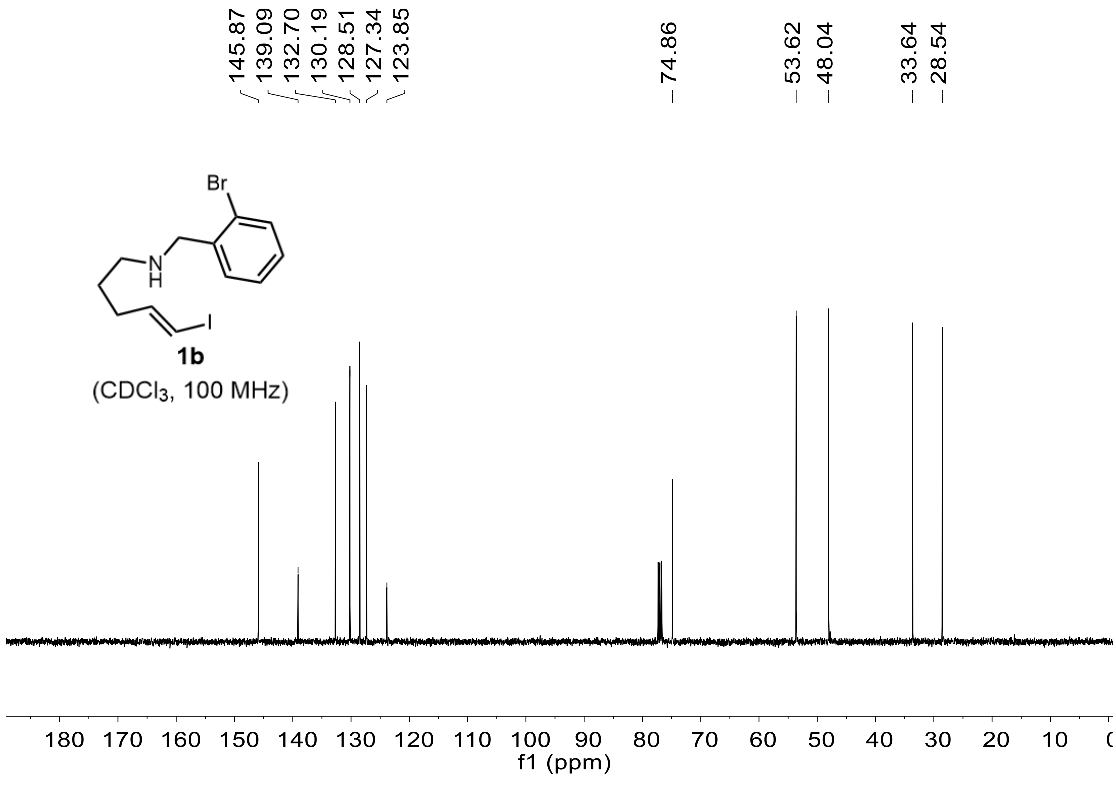

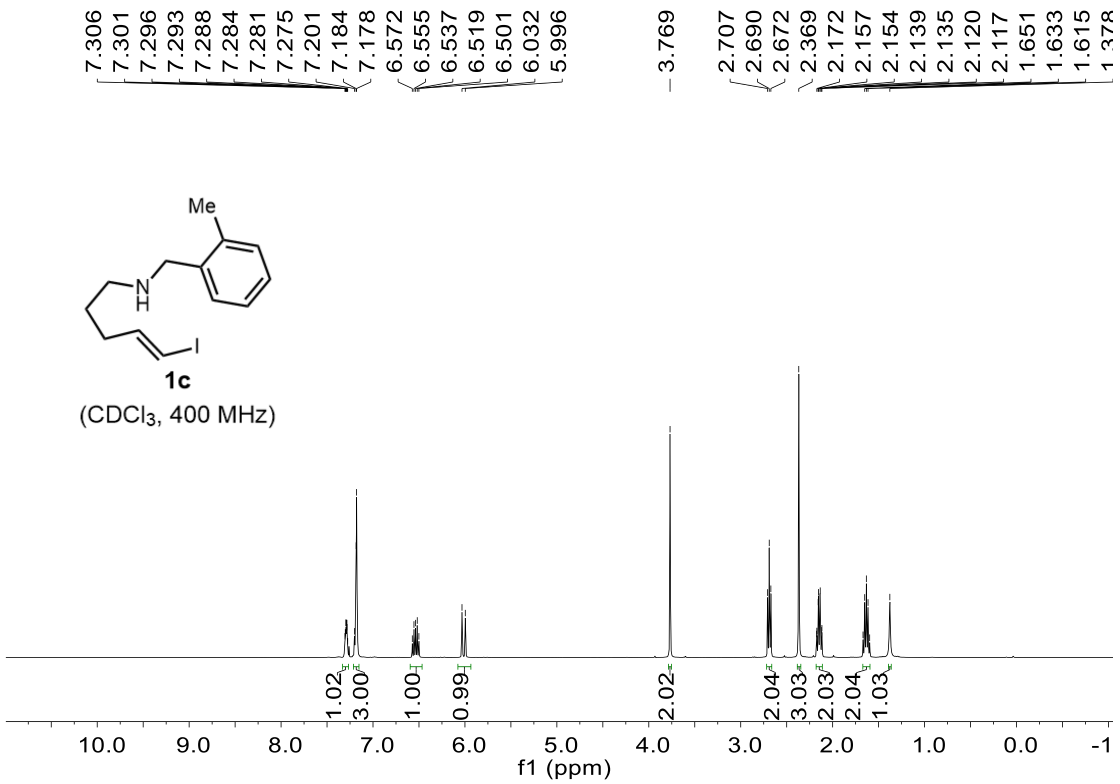

7.306
7.301
7.296
7.293
7.288
7.284
7.281
7.275
7.201
7.184
7.178
6.572
6.555
6.537
6.519
6.501
6.032
5.996
3.769
2.707
2.690
2.672
2.369
2.172
2.157
2.154
2.139
2.135
2.120
2.117
1.651
1.633
1.615
Me
N
H
I
1c
(CDCl3, 400 MHz)
1.02
3.00
1.00
0.99
2.02
2.04
3.03
2.03
2.04
1.03
10.0
9.0
8.0
7.0
6.0
5.0
4.0
3.0
2.0
1.0
0.0
f1 (ppm)

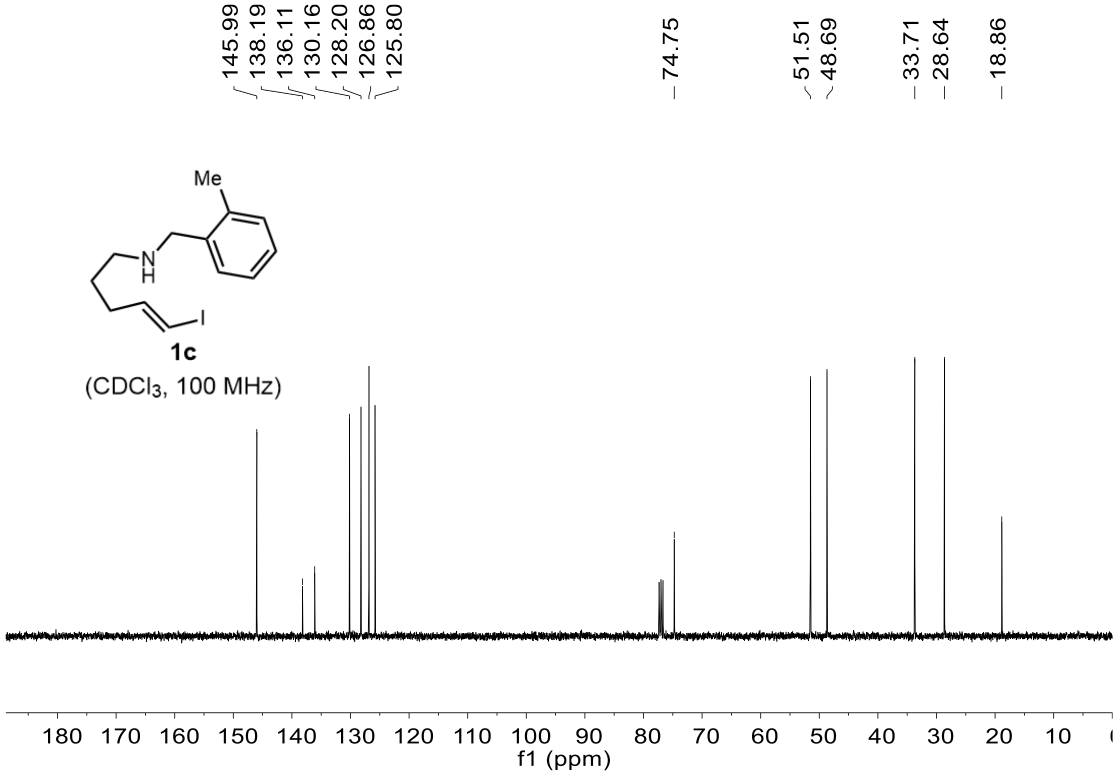

145.99
138.19
136.11
130.16
128.20
126.86
125.80
74.75
51.51
48.69
33.71
28.64
18.86
Me
N
H
I
1c
(CDCl3, 100 MHz)
180
170
160
150
140
130
120
110
100
90
80
70
60
50
40
30
20
10
f1 (ppm)

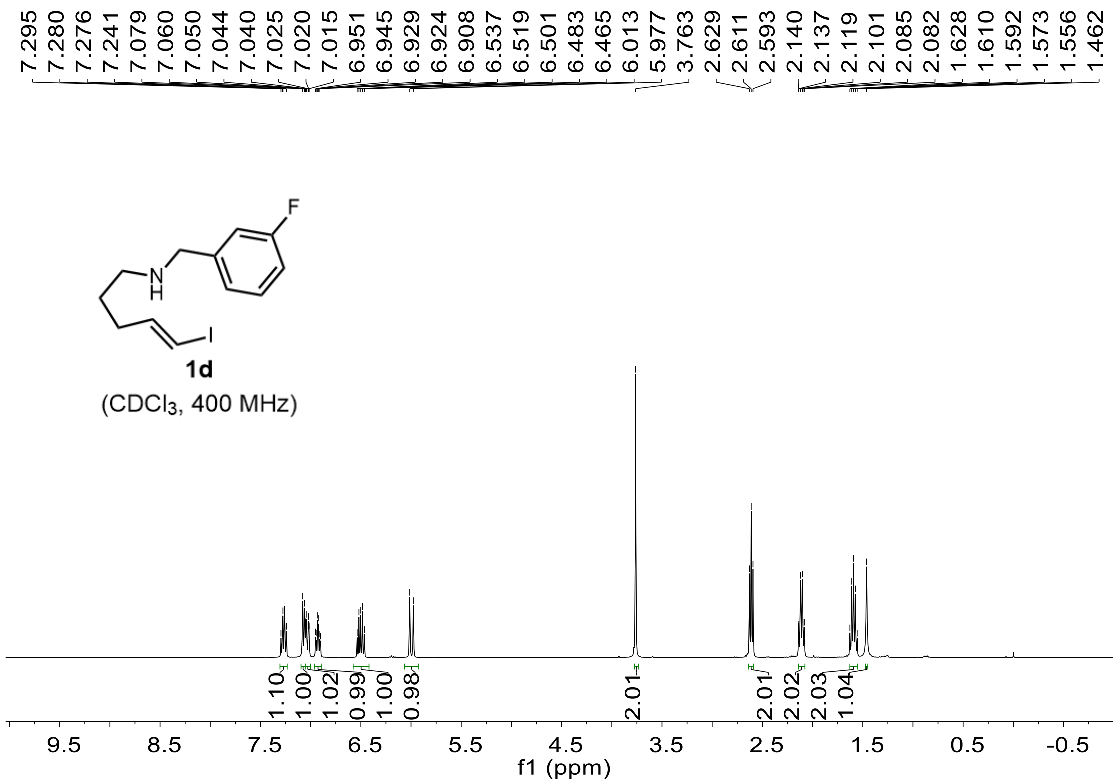
7.295 7.280 7.276 7.241 7.079 7.060 7.050 7.044 7.040 7.025 7.020 7.015 6.951 6.945 6.929 6.924 6.908 6.537 6.519 6.501 6.483 6.465 6.013 5.977 3.763 2.629 2.611 2.593 2.140 2.137 2.119 2.101 2.085 2.082 1.628 1.610 1.592 1.573 1.556 1.462
F
N
H
I
1d
(CDCl3, 400 MHz)
1.10 1.00 1.02 0.99 1.00 0.98 2.01 2.01 2.02 2.03 1.04
9.5 8.5 7.5 6.5 5.5 4.5 3.5 2.5 1.5 0.5 -0.5
f1 (ppm)

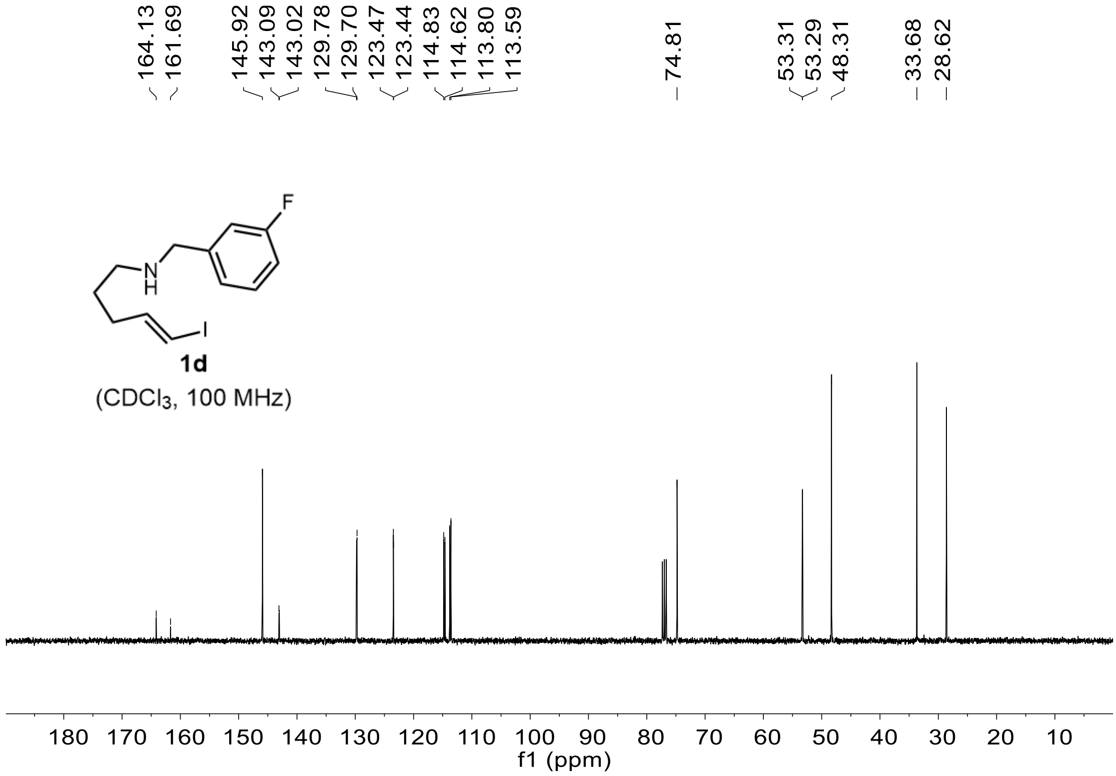
164.13 161.69 145.92 143.09 143.02 129.78 129.70 123.47 123.44 114.83 114.62 113.80 113.59 74.81 53.31 53.29 48.31 33.68 28.62
F
N
H
I
1d
(CDCl3, 100 MHz)
180 170 160 150 140 130 120 110 100 90 80 70 60 50 40 30 20 10
f1 (ppm)

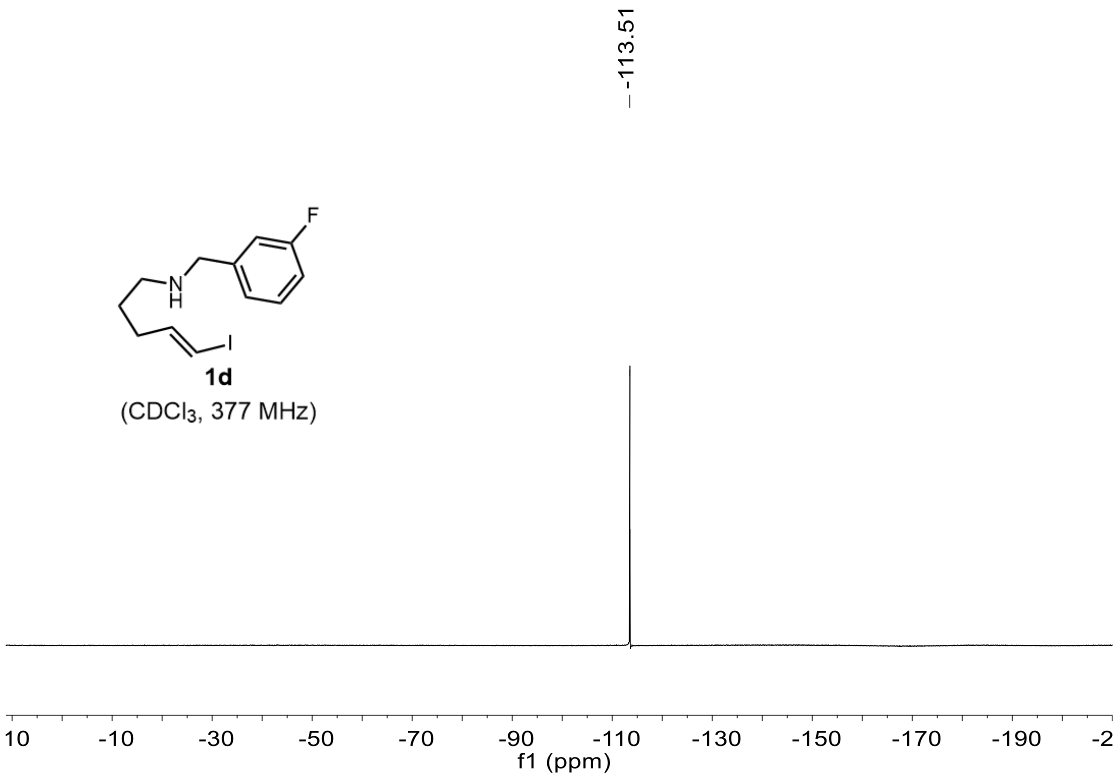

1d

($CDCl_3$, 377 MHz)

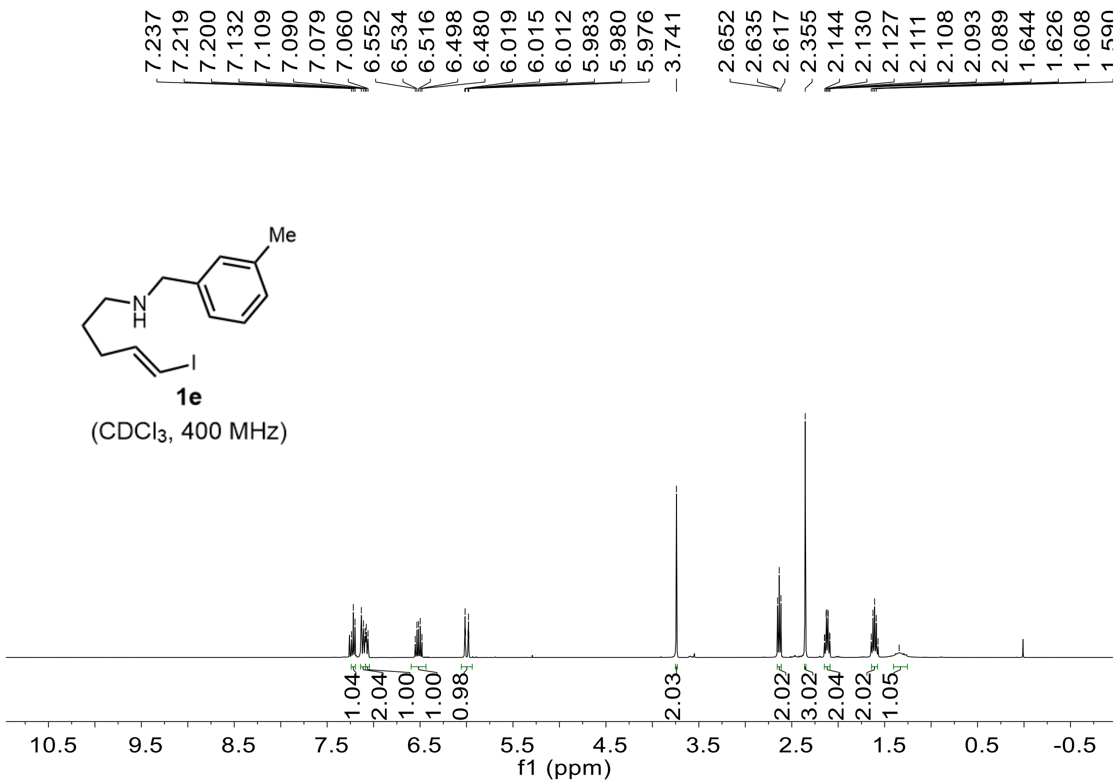
7.237
7.219
7.200
7.132
7.109
7.090
7.079
7.060
6.552
6.534
6.516
6.498
6.480
6.019
6.015
6.012
5.983
5.980
5.976
3.741
2.652
2.635
2.617
2.355
2.144
2.130
2.127
2.111
2.108
2.093
2.089
1.644
1.626
1.608
Me
N
H
I
1e
(CDCl3, 400 MHz)
1.04
2.04
1.00
1.00
0.98
2.03
2.02
3.02
2.04
2.02
1.05
10.5
9.5
8.5
7.5
6.5
5.5
4.5
3.5
2.5
1.5
0.5
-0.5
f1 (ppm)

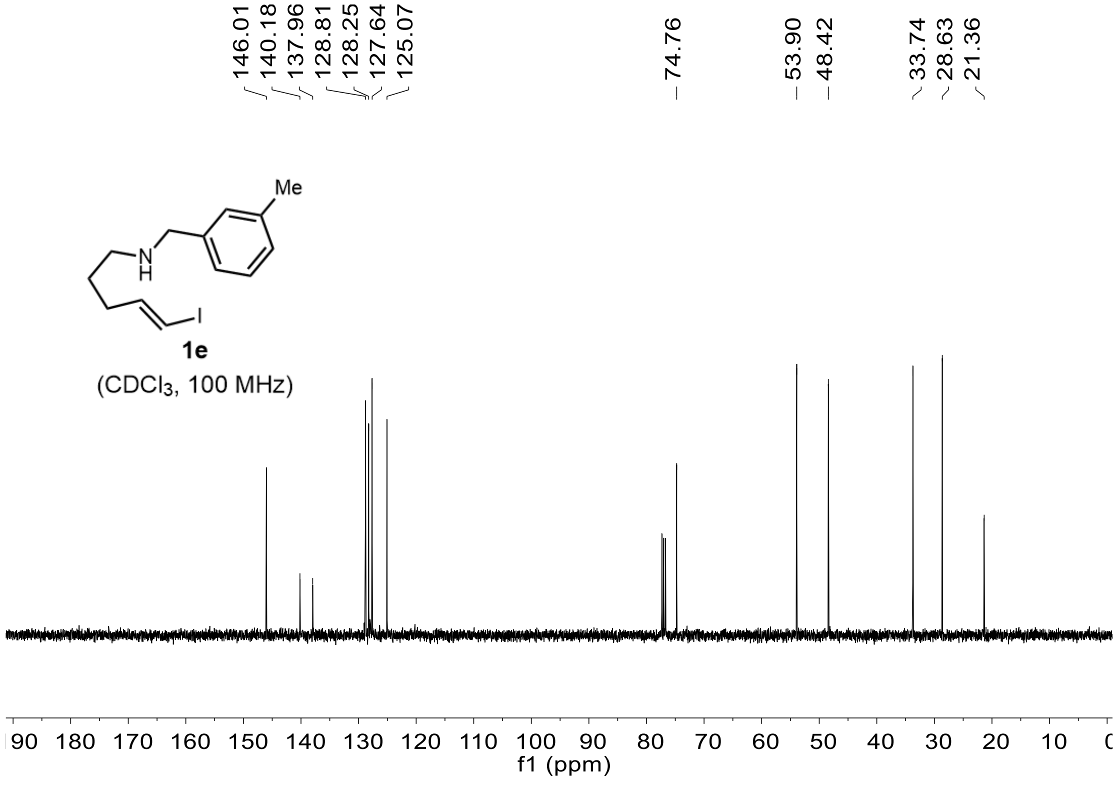
146.01
140.18
137.96
128.81
128.25
127.64
125.07
74.76
53.90
48.42
33.74
28.63
21.36
Me
N
H
I
1e
(CDCl3, 100 MHz)
180
170
160
150
140
130
120
110
100
90
80
70
60
50
40
30
20
10
f1 (ppm)

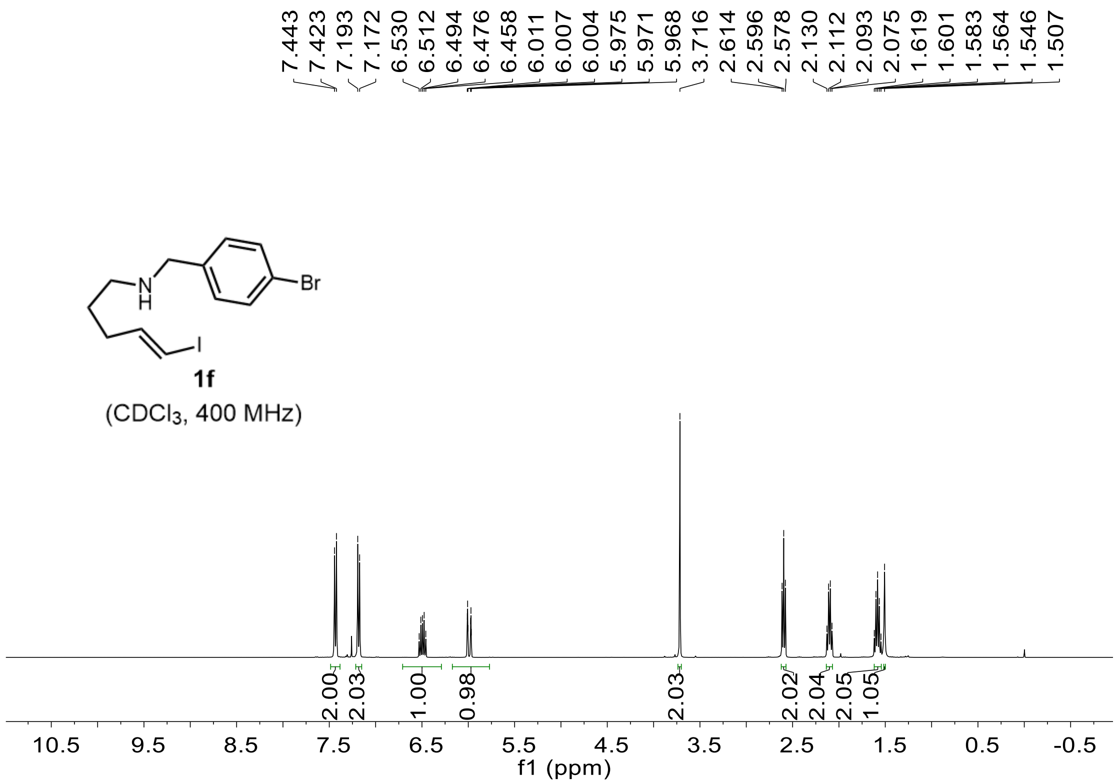
7.443
7.423
7.193
7.172
6.530
6.512
6.494
6.476
6.458
6.011
6.007
6.004
5.975
5.971
5.968
3.716
2.614
2.596
2.578
2.130
2.112
2.093
2.075
1.619
1.601
1.583
1.564
1.546
1.507
Br
N
H
I
1f
(CDCl3, 400 MHz)
2.00
2.03
1.00
0.98
2.03
2.02
2.04
2.05
1.05
10.5 9.5 8.5 7.5 6.5 5.5 4.5 3.5 2.5 1.5 0.5 -0.5
f1 (ppm)

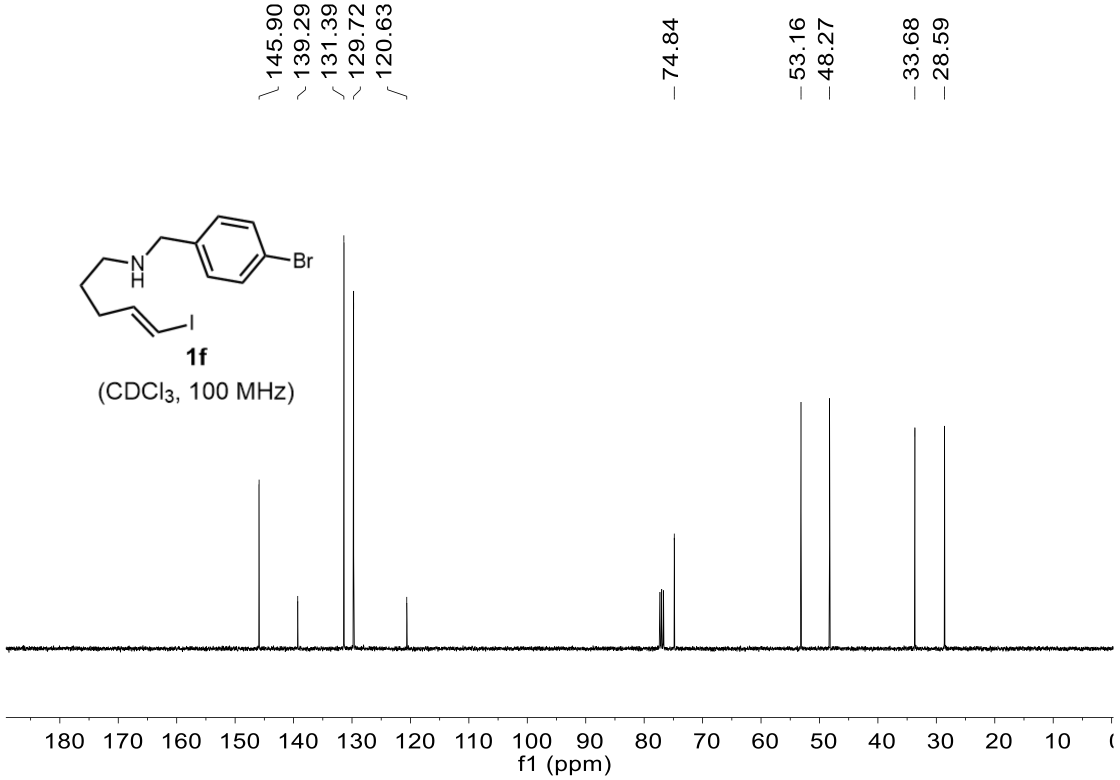
145.90
139.29
131.39
129.72
120.63
74.84
53.16
48.27
33.68
28.59
Br
N
H
I
1f
(CDCl3, 100 MHz)
180 170 160 150 140 130 120 110 100 90 80 70 60 50 40 30 20 10
f1 (ppm)

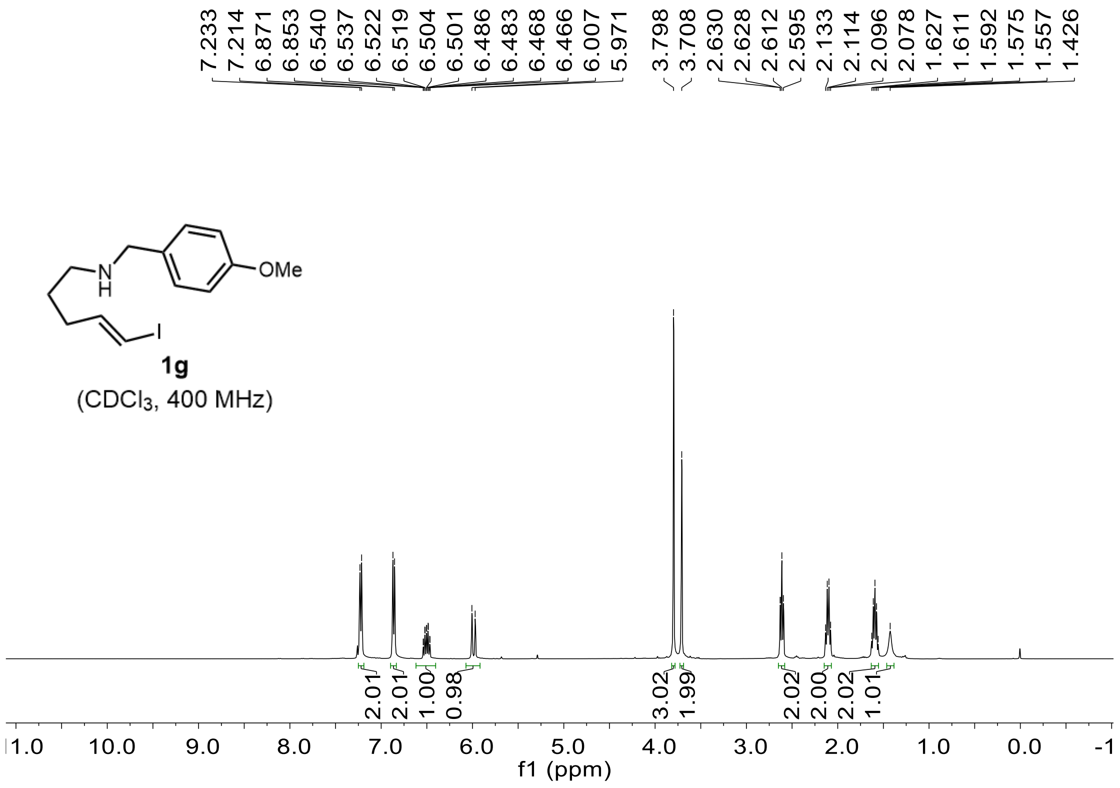
7.233
7.214
6.871
6.853
6.540
6.537
6.522
6.519
6.504
6.501
6.486
6.483
6.468
6.466
6.007
5.971
3.798
3.708
2.630
2.628
2.612
2.595
2.133
2.114
2.096
2.078
1.627
1.611
1.592
1.575
1.557
1.426
N
H
OMe
I
1g
(CDCl3, 400 MHz)
2.01
2.01
1.00
0.98
3.02
1.99
2.02
2.00
2.02
1.01
f1 (ppm)

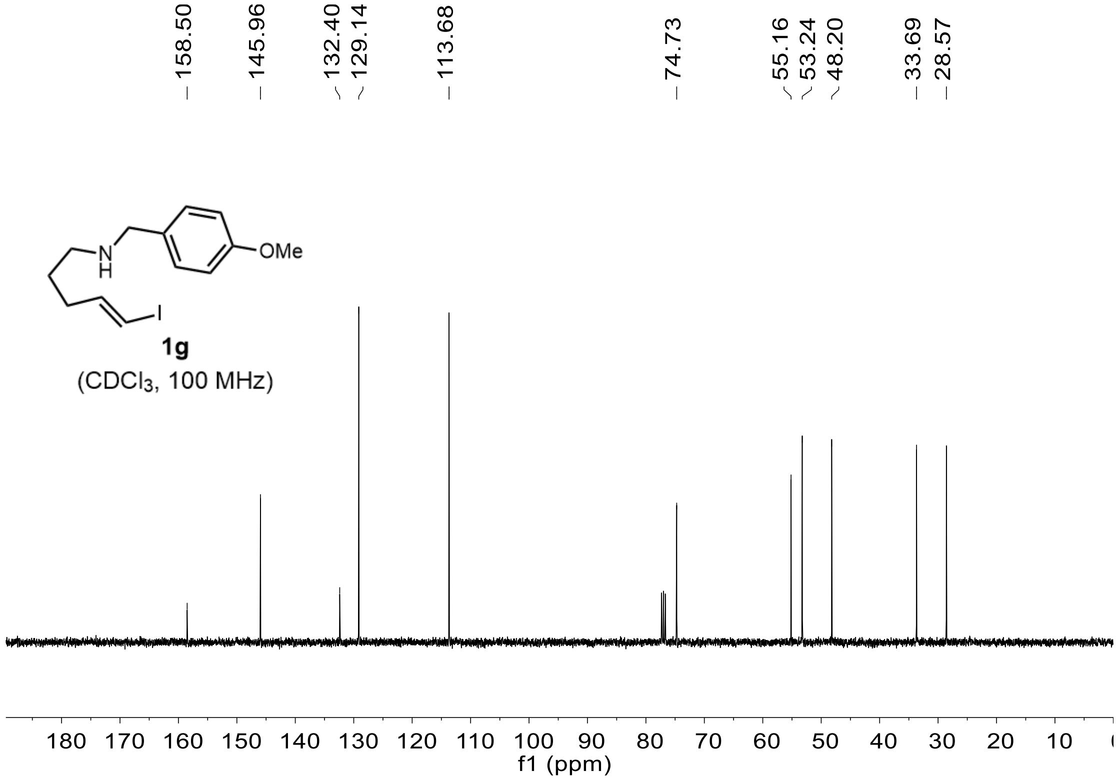
158.50
145.96
132.40
129.14
113.68
74.73
55.16
53.24
48.20
33.69
28.57
N
H
OMe
I
1g
(CDCl3, 100 MHz)
f1 (ppm)

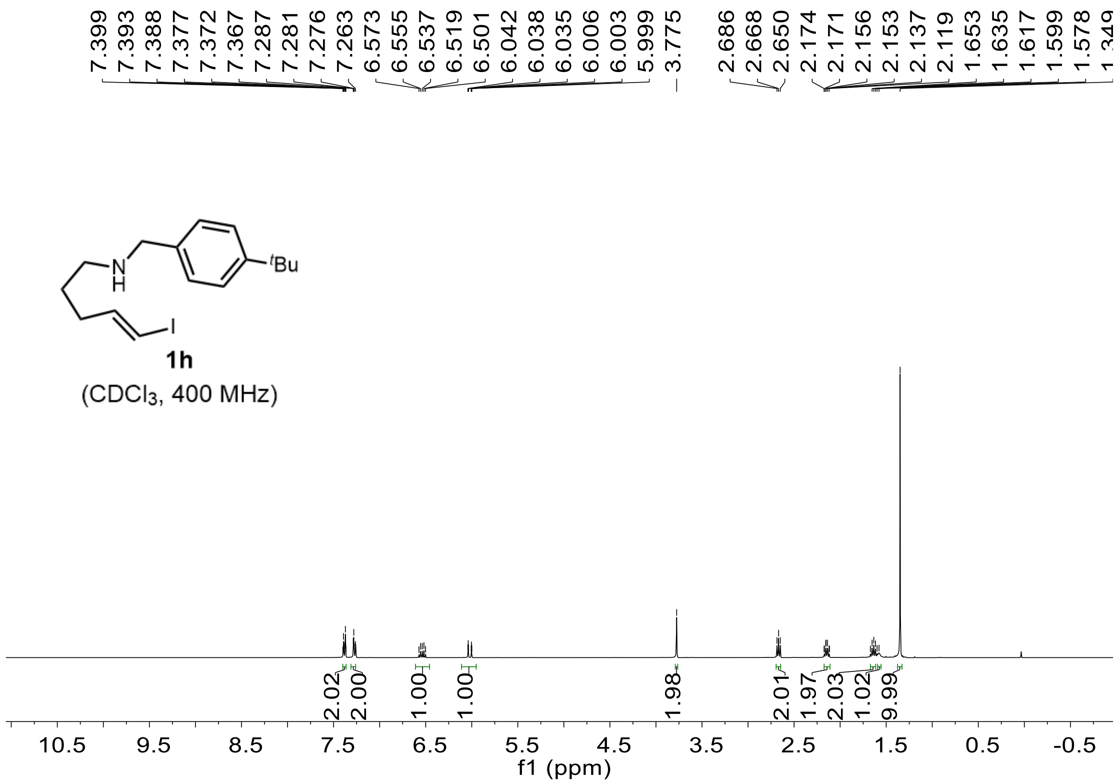
7.399 7.393 7.388 7.377 7.372 7.367 7.287 7.281 7.276 7.263 6.573 6.555 6.537 6.519 6.501 6.042 6.038 6.035 6.006 6.003 5.999 3.775 2.686 2.668 2.650 2.174 2.171 2.156 2.153 2.137 2.119 1.653 1.635 1.617 1.599 1.578
N
H
ᵗBu
I
1h
(CDCl3, 400 MHz)
2.02 2.00 1.00 1.00 1.98 2.01 1.97 2.03 1.02 9.99
10.5 9.5 8.5 7.5 6.5 5.5 4.5 3.5 2.5 1.5 0.5 -0.5
f1 (ppm)

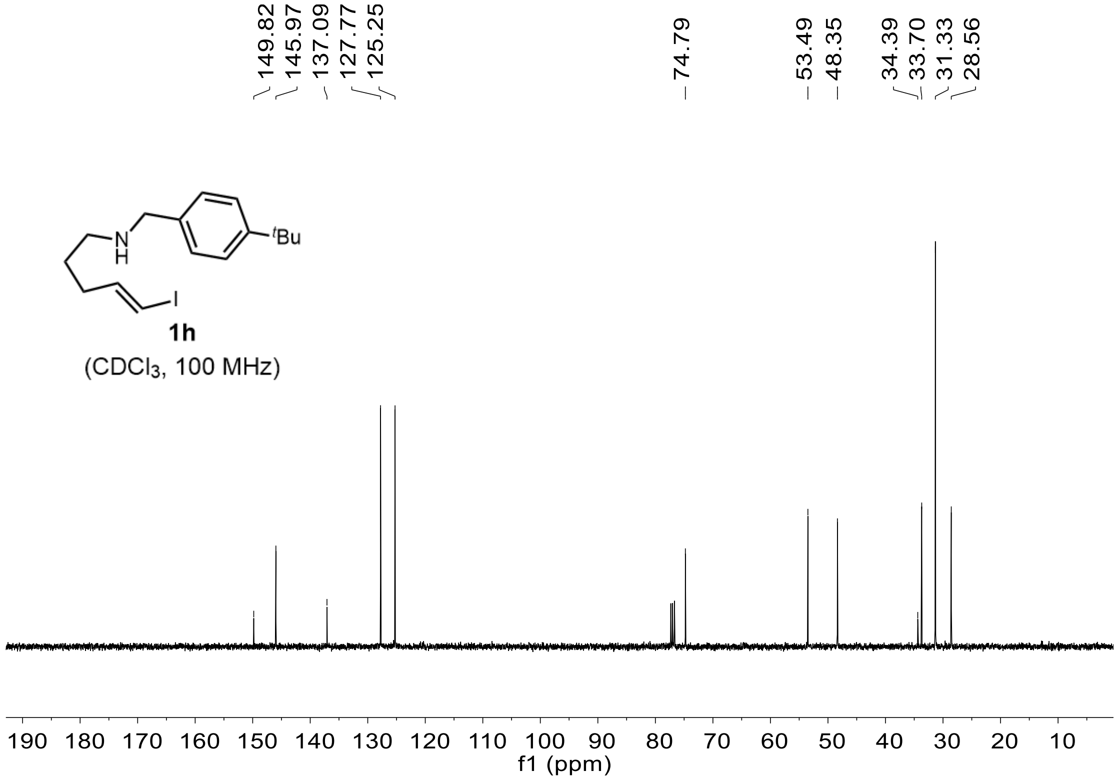
149.82 145.97 137.09 127.77 125.25 74.79 53.49 48.35 34.39 33.70 31.33 28.56
N
H
ᵗBu
I
1h
(CDCl3, 100 MHz)
190 180 170 160 150 140 130 120 110 100 90 80 70 60 50 40 30 20 10
f1 (ppm)

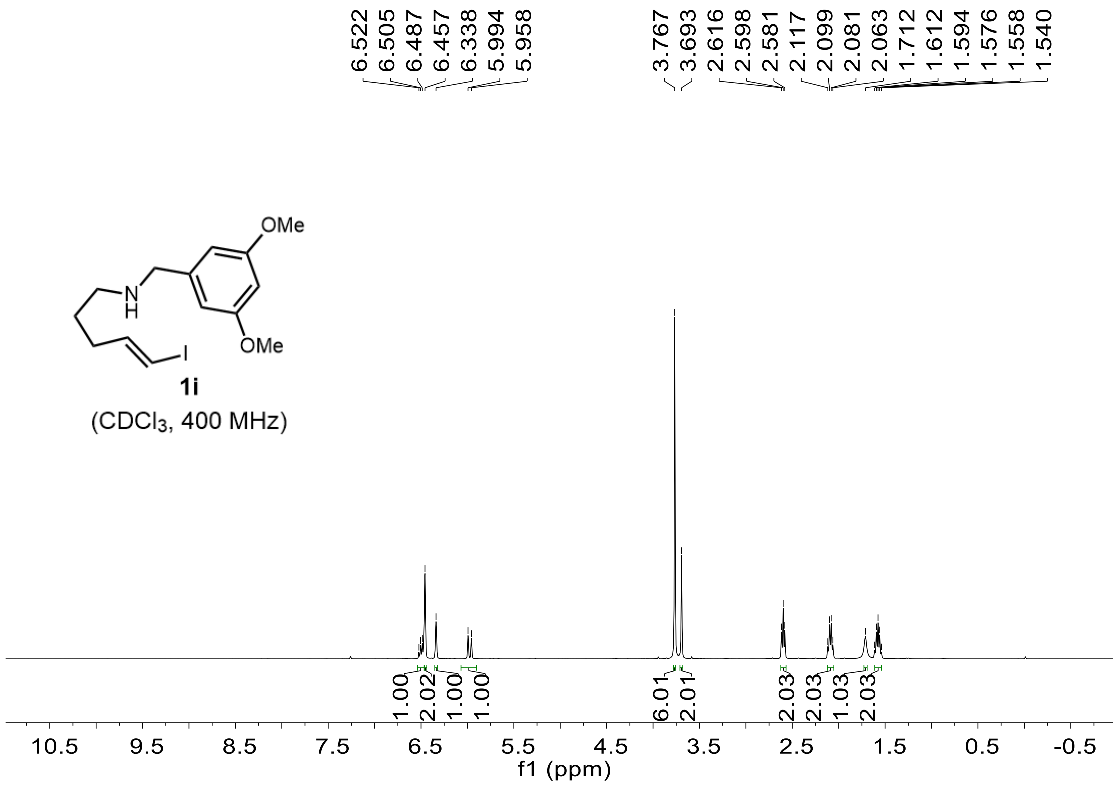
6.522 6.505 6.487 6.457 6.338 5.994 5.958
3.767 3.693 2.616 2.598 2.581 2.117 2.099 2.081 2.063 1.712 1.612 1.594 1.576 1.558 1.540
OMe
N
H
I
OMe
1i
(CDCl3, 400 MHz)
1.00 2.02 1.00 1.00
6.01 2.01
2.03 2.03 1.03 2.03
10.5 9.5 8.5 7.5 6.5 5.5 4.5 3.5 2.5 1.5 0.5 -0.5
f1 (ppm)

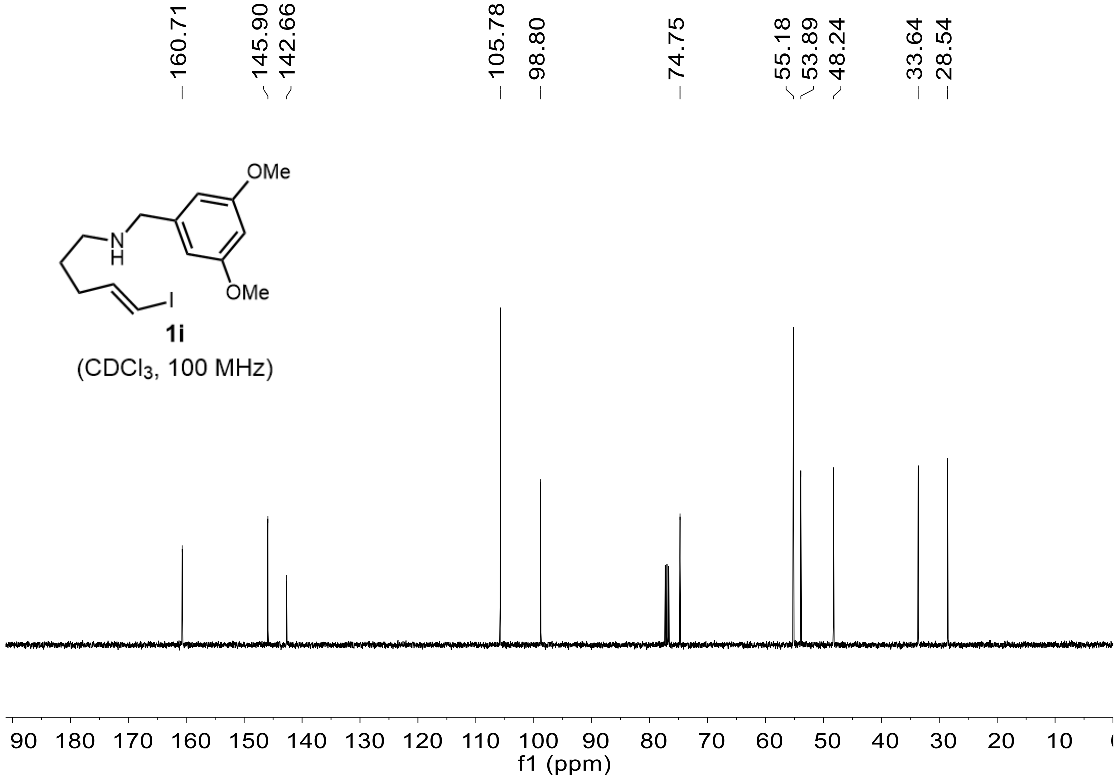
160.71
145.90 142.66
105.78 98.80
74.75
55.18 53.89 48.24
33.64 28.54
OMe
N
H
I
OMe
1i
(CDCl3, 100 MHz)
90 180 170 160 150 140 130 120 110 100 90 80 70 60 50 40 30 20 10
f1 (ppm)

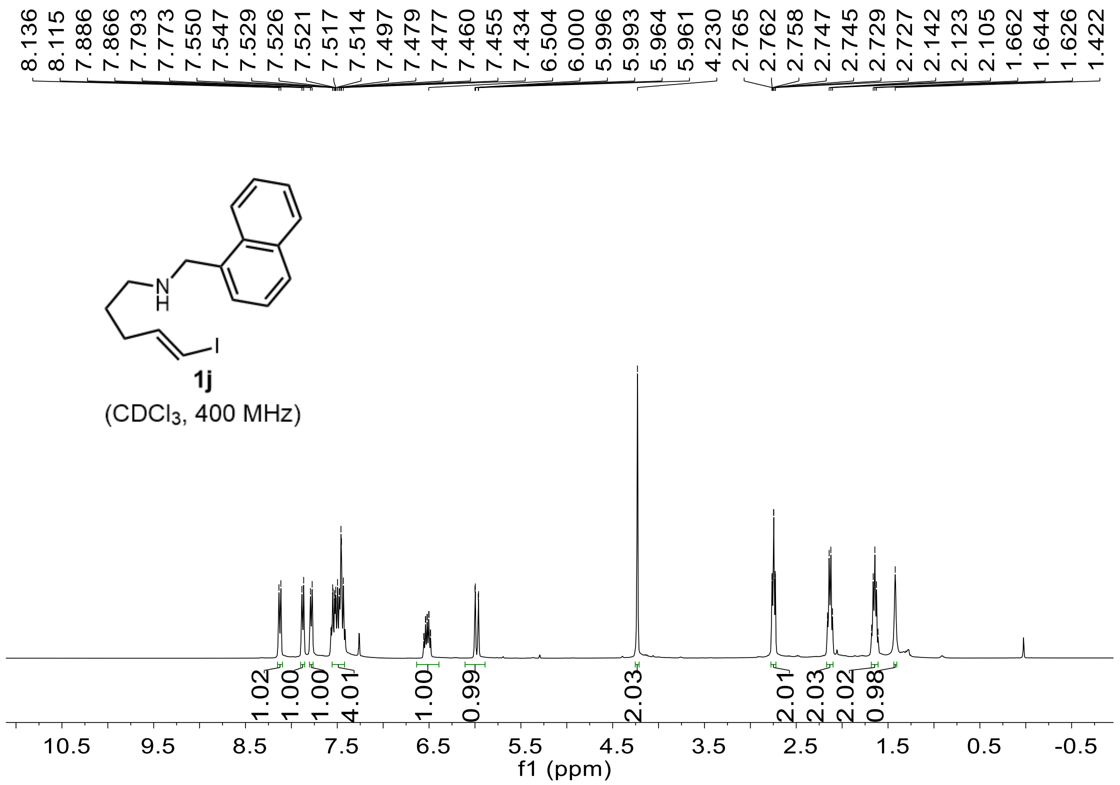
8.136 8.115 7.886 7.866 7.793 7.773 7.550 7.547 7.529 7.526 7.521 7.517 7.514 7.497 7.479 7.477 7.460 7.455 7.434 6.504 6.000 5.996 5.993 5.964 5.961 4.230 2.765 2.762 2.758 2.747 2.745 2.729 2.727 2.142 2.123 2.105 1.662 1.644 1.626 1.422
1j
(CDCl3, 400 MHz)
1.02 1.00 1.00 4.01 1.00 0.99 2.03 2.01 2.03 2.02 0.98
10.5 9.5 8.5 7.5 6.5 5.5 4.5 3.5 2.5 1.5 0.5 -0.5
f1 (ppm)

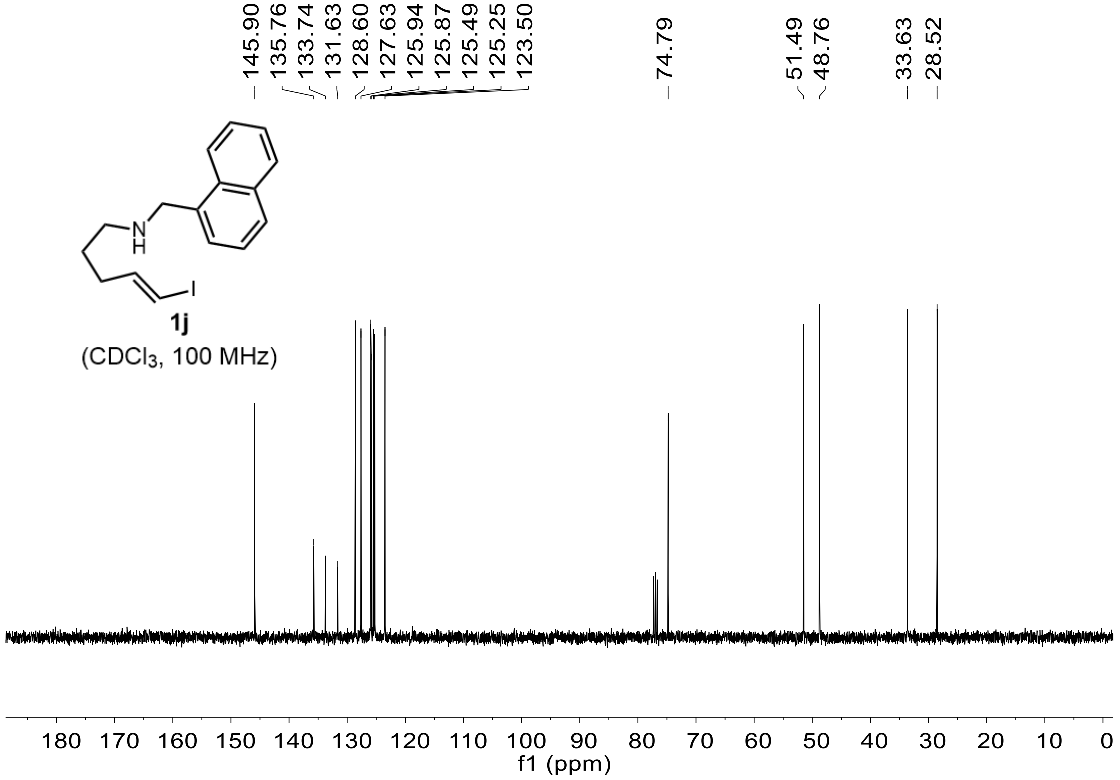
145.90 135.76 133.74 131.63 128.60 127.63 125.94 125.87 125.49 125.25 123.50 74.79 51.49 48.76 33.63 28.52
1j
(CDCl3, 100 MHz)
180 170 160 150 140 130 120 110 100 90 80 70 60 50 40 30 20 10 0
f1 (ppm)

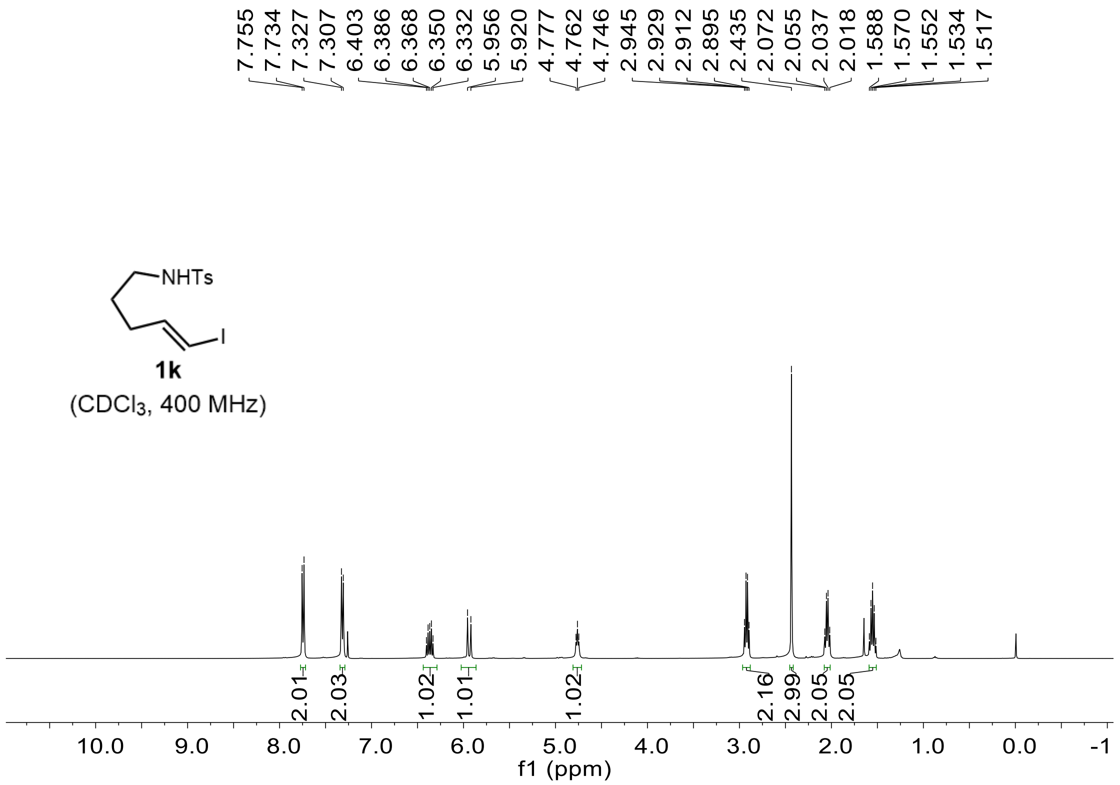
7.755 7.734 7.327 7.307 6.403 6.386 6.368 6.350 6.332 5.956 5.920 4.777 4.762 4.746 2.945 2.929 2.912 2.895 2.435 2.072 2.055 2.037 2.018 1.588 1.570 1.552 1.534 1.517
NHTs
I
1k
(CDCl3, 400 MHz)
2.01 2.03 1.02 1.01 1.02 2.16 2.99 2.05 2.05
10.0 9.0 8.0 7.0 6.0 5.0 4.0 3.0 2.0 1.0 0.0 -1
f1 (ppm)

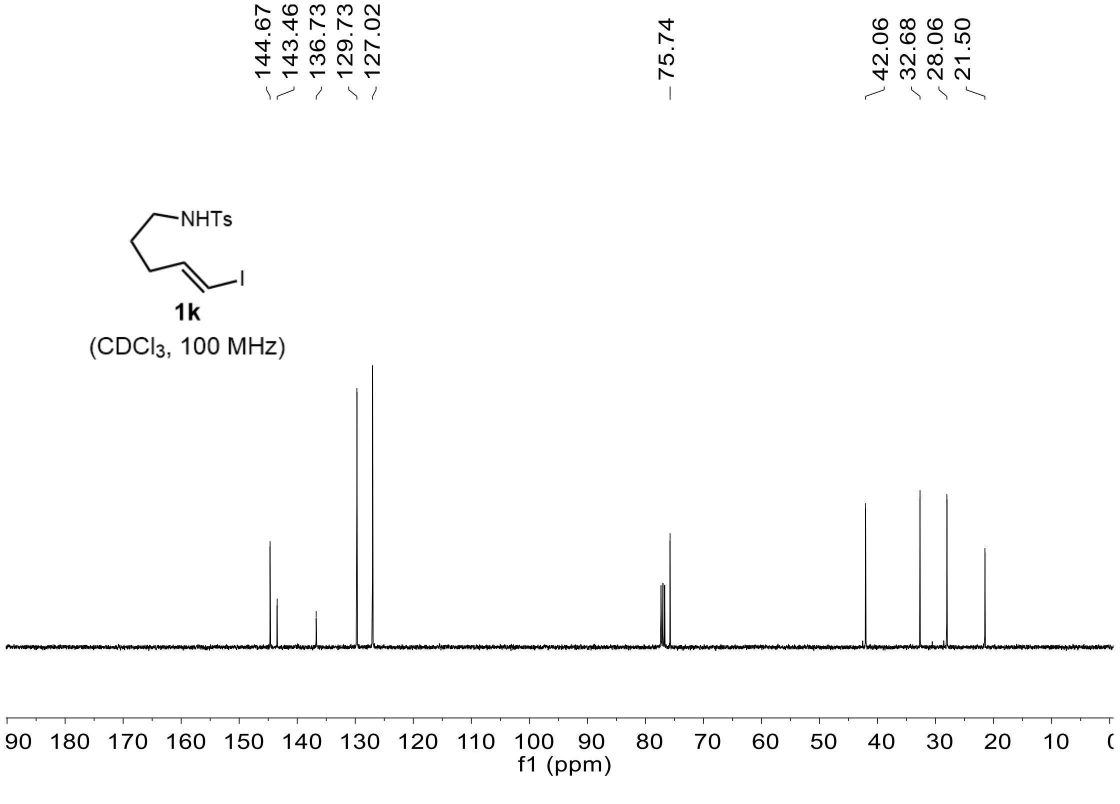
144.67 143.46 136.73 129.73 127.02
75.74
42.06 32.68 28.06 21.50
NHTs
I
1k
(CDCl3, 100 MHz)
90 180 170 160 150 140 130 120 110 100 90 80 70 60 50 40 30 20 10
f1 (ppm)

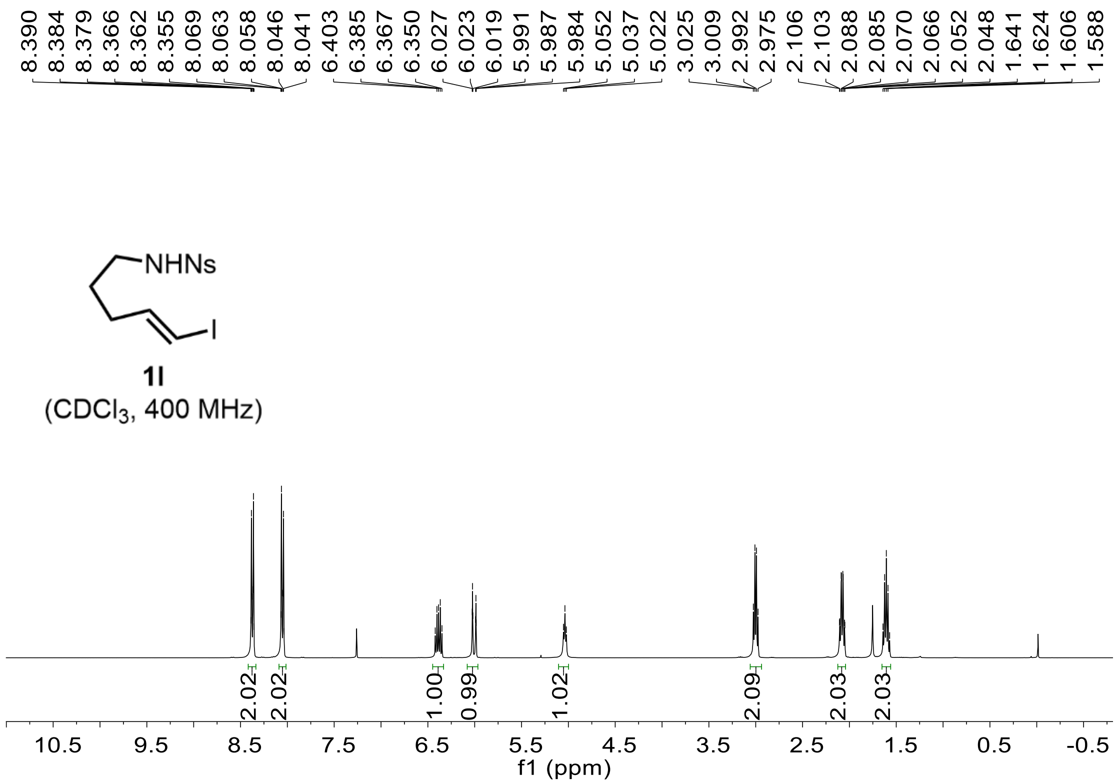

1l
($CDCl_3$, 400 MHz)

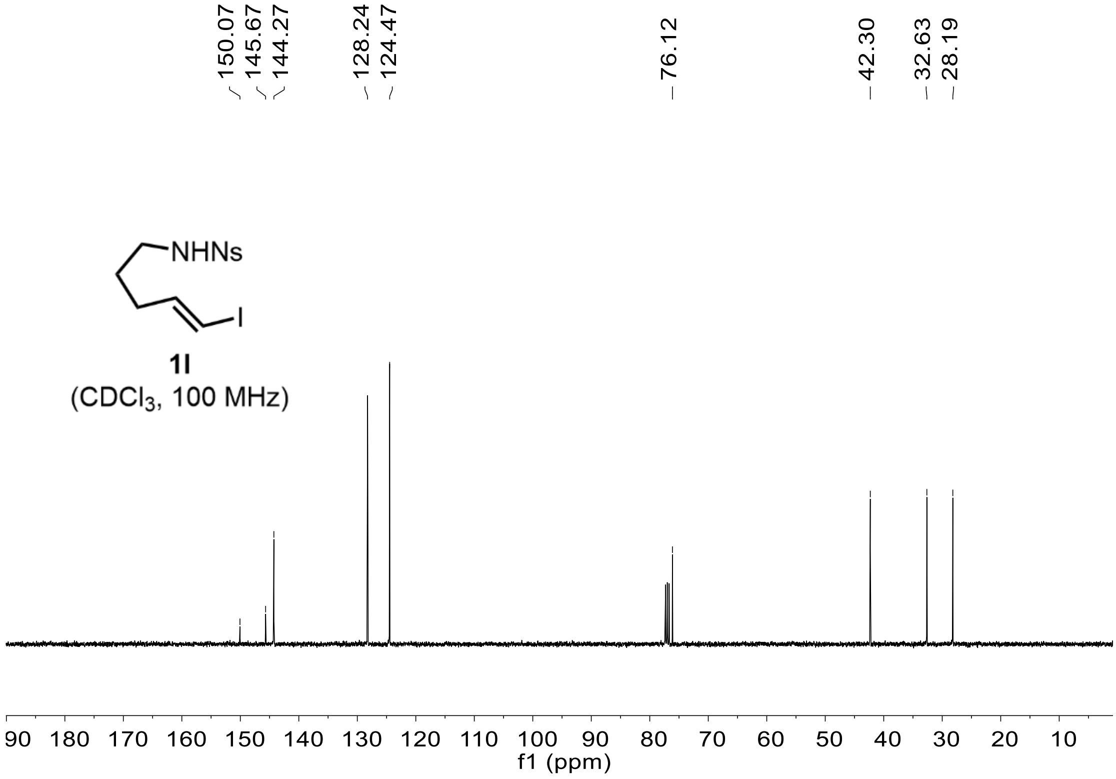

1l
($CDCl_3$, 100 MHz)

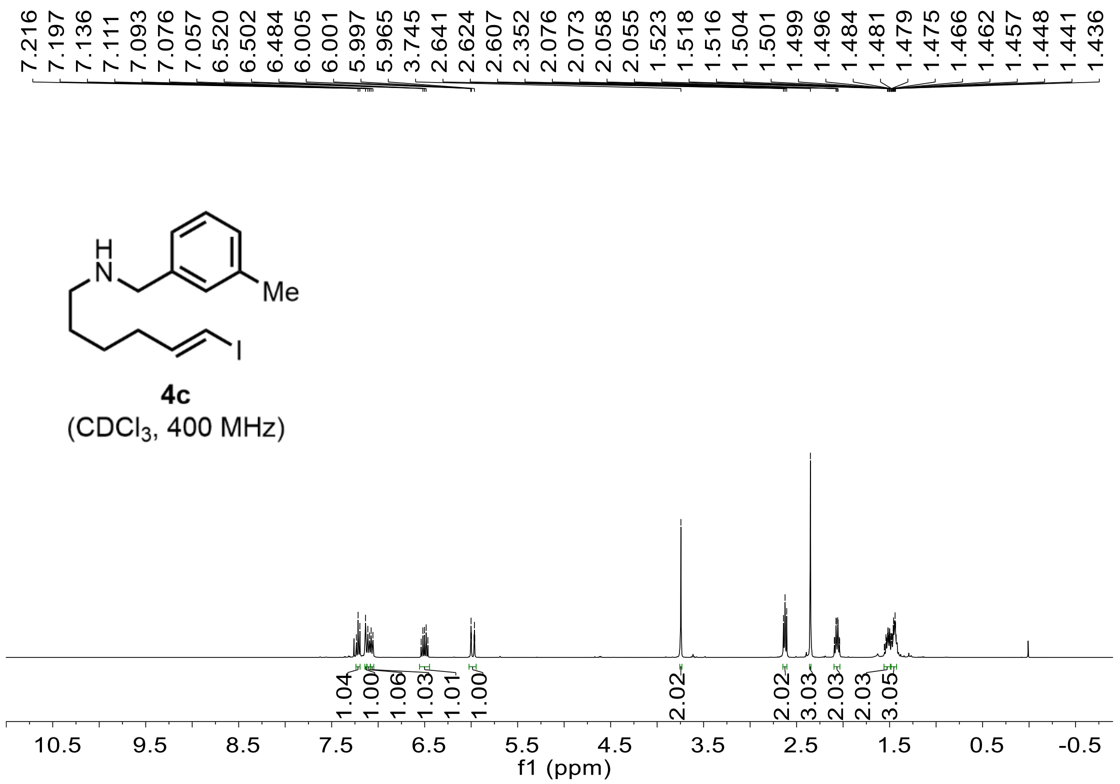
7.216
7.197
7.136
7.111
7.093
7.076
7.057
6.520
6.502
6.484
6.005
6.001
5.997
5.965
3.745
2.641
2.624
2.607
2.352
2.076
2.073
2.058
2.055
1.523
1.518
1.516
1.504
1.501
1.499
1.496
1.484
1.481
1.479
1.475
1.466
1.462
1.457
1.448
1.441
1.436
H
N
Me
I
4c
(CDCl3, 400 MHz)
1.04
1.00
1.06
1.03
1.01
1.00
2.02
2.02
3.03
2.03
2.03
3.05
10.5 9.5 8.5 7.5 6.5 5.5 4.5 3.5 2.5 1.5 0.5 -0.5
f1 (ppm)

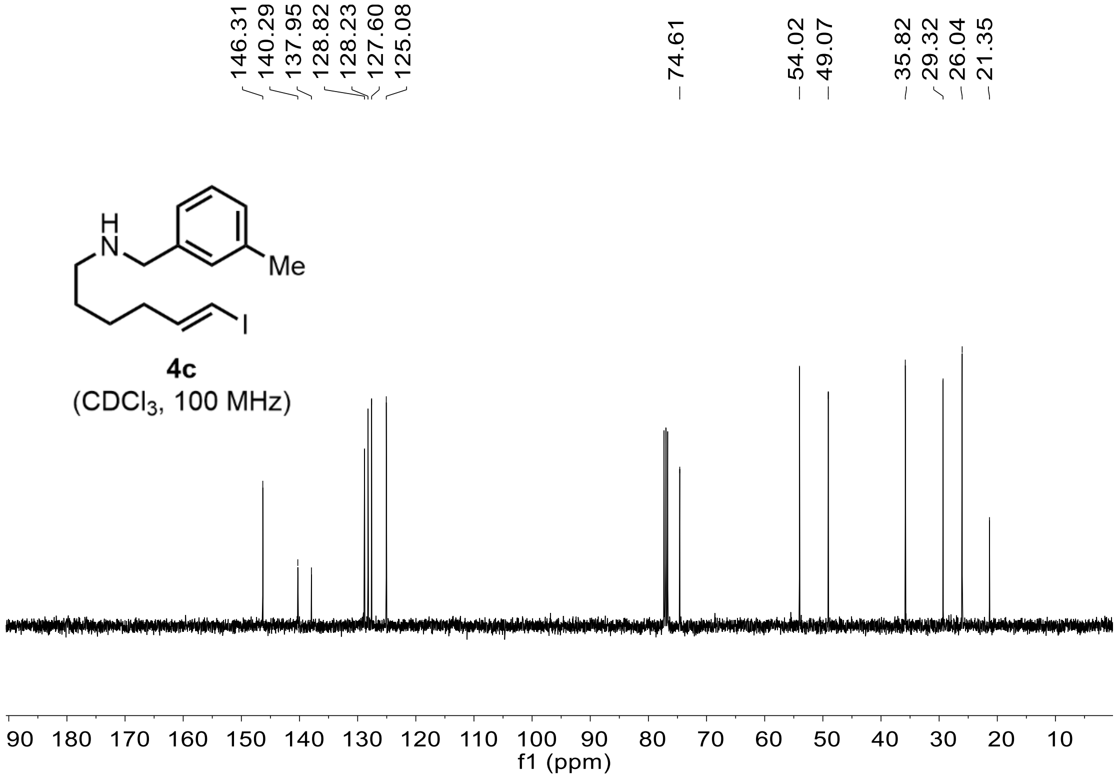
146.31
140.29
137.95
128.82
128.23
127.60
125.08
74.61
54.02
49.07
35.82
29.32
26.04
21.35
H
N
Me
I
4c
(CDCl3, 100 MHz)
90 180 170 160 150 140 130 120 110 100 90 80 70 60 50 40 30 20 10
f1 (ppm)

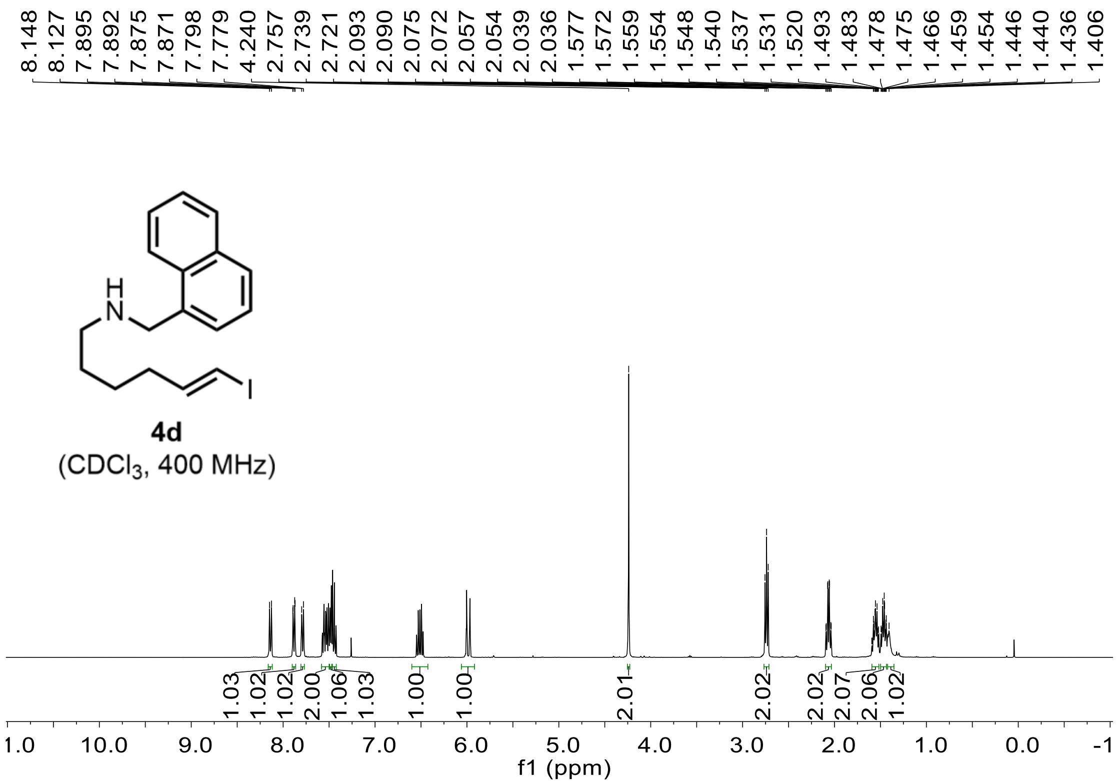
8.148
8.127
7.895
7.892
7.875
7.871
7.798
7.779
4.240
2.757
2.739
2.721
2.093
2.090
2.075
2.072
2.057
2.054
2.039
2.036
1.577
1.572
1.559
1.554
1.548
1.540
1.537
1.531
1.520
1.493
1.483
1.478
1.475
1.466
1.459
1.454
1.446
1.440
1.436
1.406
H
N
I
4d
(CDCl3, 400 MHz)
1.03
1.02
1.02
2.00
1.06
1.03
1.00
1.00
2.01
2.02
2.02
2.07
2.06
1.02
1.0
10.0
9.0
8.0
7.0
6.0
5.0
4.0
3.0
2.0
1.0
0.0
-1
f1 (ppm)

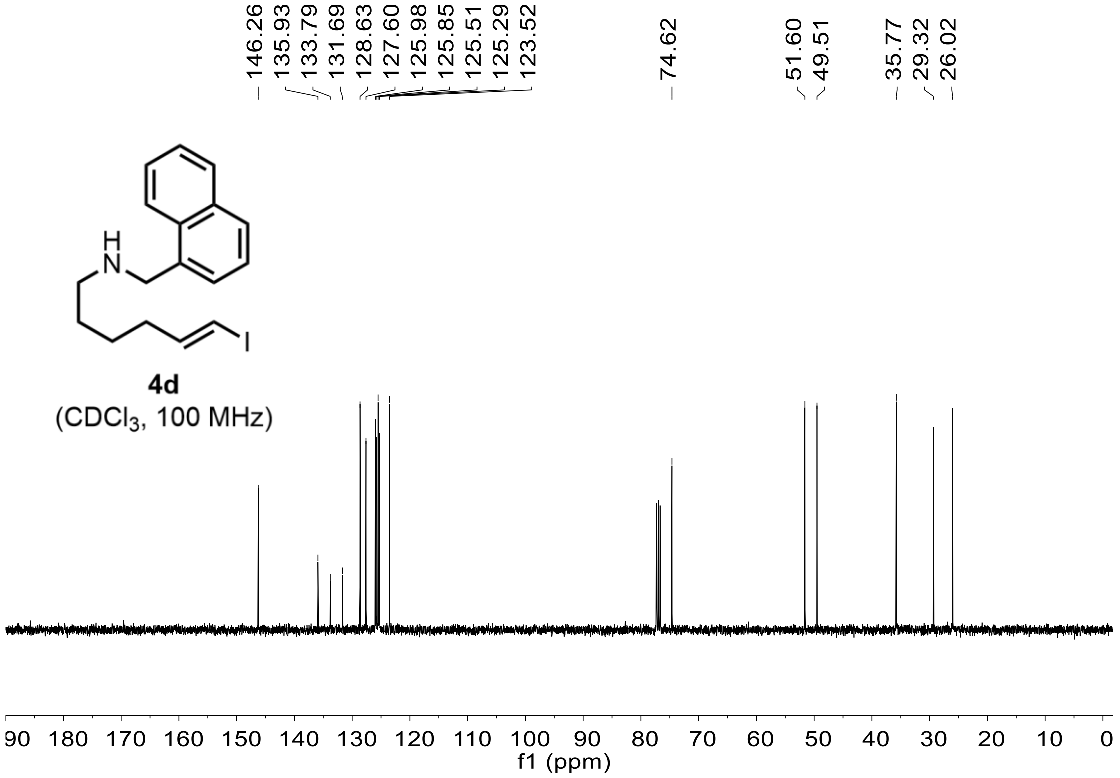
146.26
135.93
133.79
131.69
128.63
127.60
125.98
125.85
125.51
125.29
123.52
74.62
51.60
49.51
35.77
29.32
26.02
H
N
I
4d
(CDCl3, 100 MHz)
90
180
170
160
150
140
130
120
110
100
90
80
70
60
50
40
30
20
10
0
f1 (ppm)

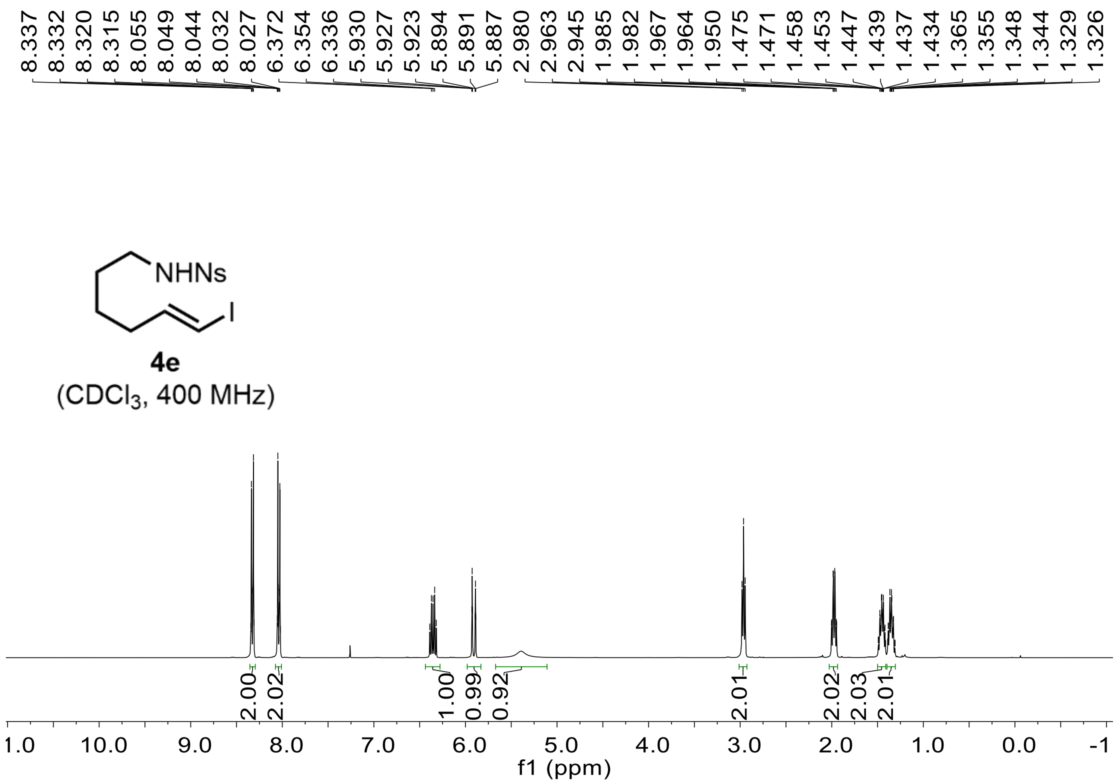

8.337
8.332
8.320
8.315
8.055
8.049
8.044
8.032
8.027
6.372
6.354
6.336
5.930
5.927
5.923
5.894
5.891
5.887
2.980
2.963
2.945
1.985
1.982
1.967
1.964
1.950
1.475
1.471
1.458
1.453
1.447
1.439
1.437
1.434
1.365
1.355
1.348
1.344
1.329
1.326
NHNs
I
4e
(CDCl3, 400 MHz)
2.00
2.02
1.00
0.99
0.92
2.01
2.02
2.03
2.01
f1 (ppm)

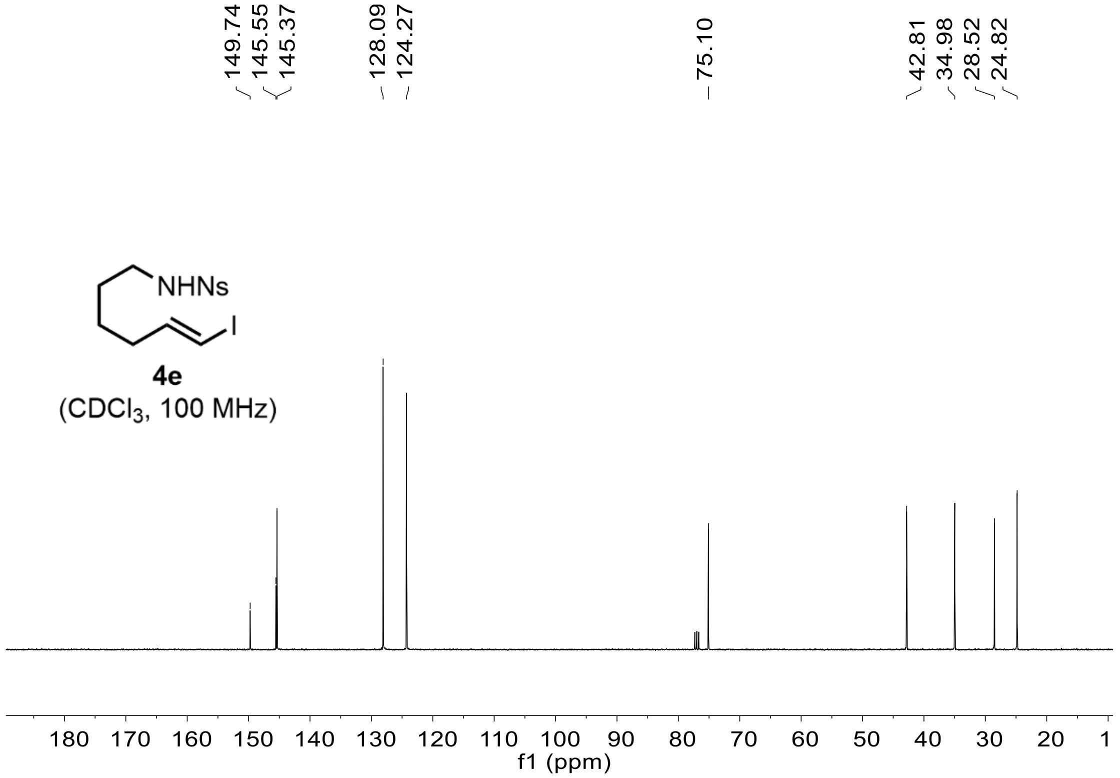

149.74
145.55
145.37
128.09
124.27
75.10
42.81
34.98
28.52
24.82
NHNs
I
4e
(CDCl3, 100 MHz)
f1 (ppm)

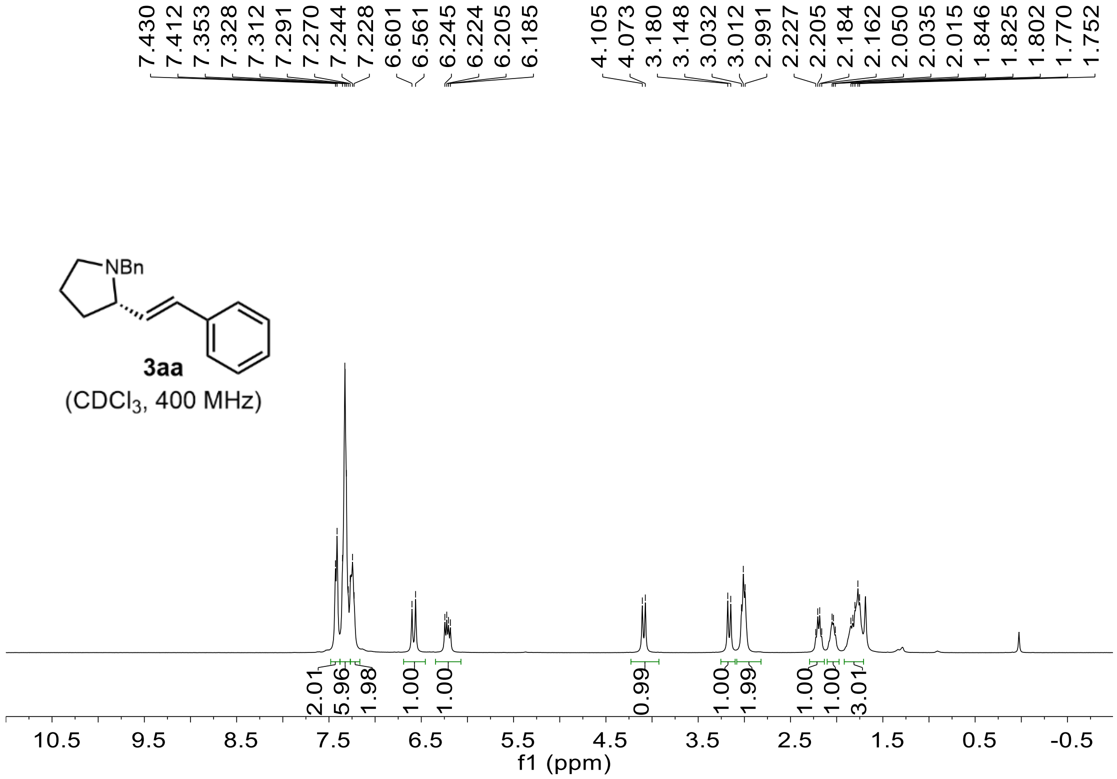
7.430 7.412 7.353 7.328 7.312 7.291 7.270 7.244 7.228 6.601 6.561 6.245 6.224 6.205 6.185 4.105 4.073 3.180 3.148 3.032 3.012 2.991 2.227 2.205 2.184 2.162 2.050 2.035 2.015 1.846 1.825 1.802 1.770 1.752
NBn
3aa
(CDCl$_3$, 400 MHz)
2.01 5.96 1.98 1.00 1.00 0.99 1.00 1.99 1.00 1.00 3.01
10.5 9.5 8.5 7.5 6.5 5.5 4.5 3.5 2.5 1.5 0.5 -0.5
f1 (ppm)

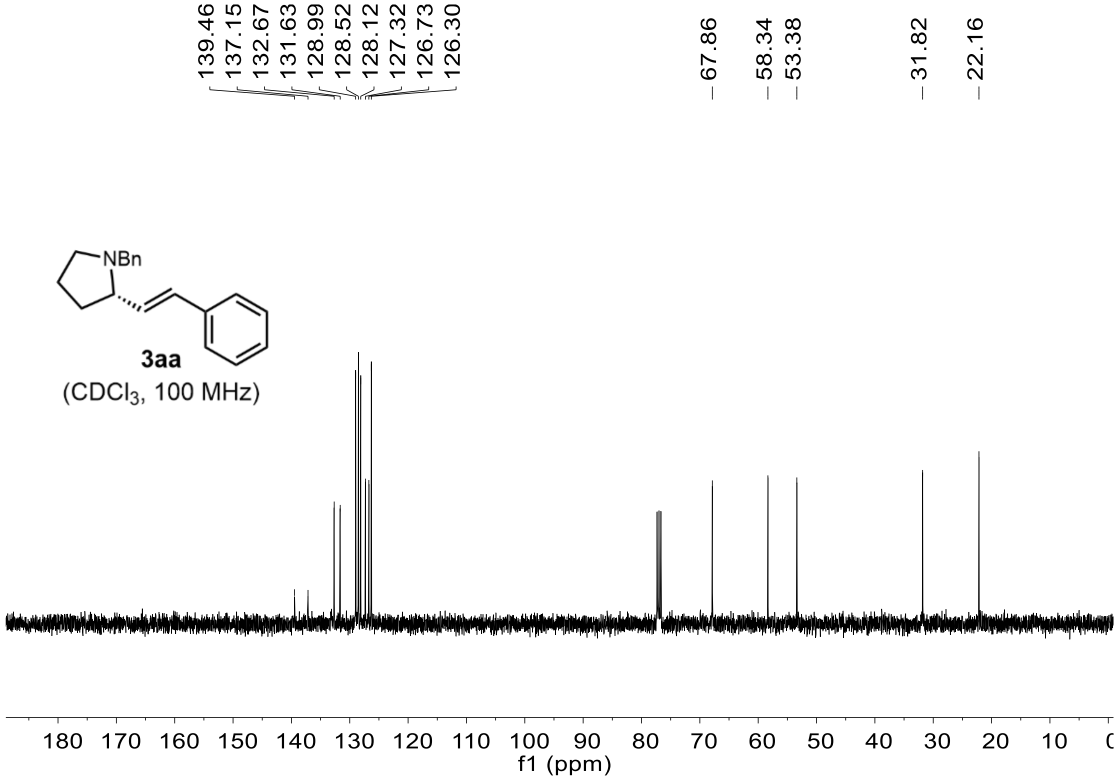
139.46 137.15 132.67 131.63 128.99 128.52 128.12 127.32 126.73 126.30
67.86 58.34 53.38 31.82 22.16
NBn
3aa
(CDCl$_3$, 100 MHz)
180 170 160 150 140 130 120 110 100 90 80 70 60 50 40 30 20 10
f1 (ppm)

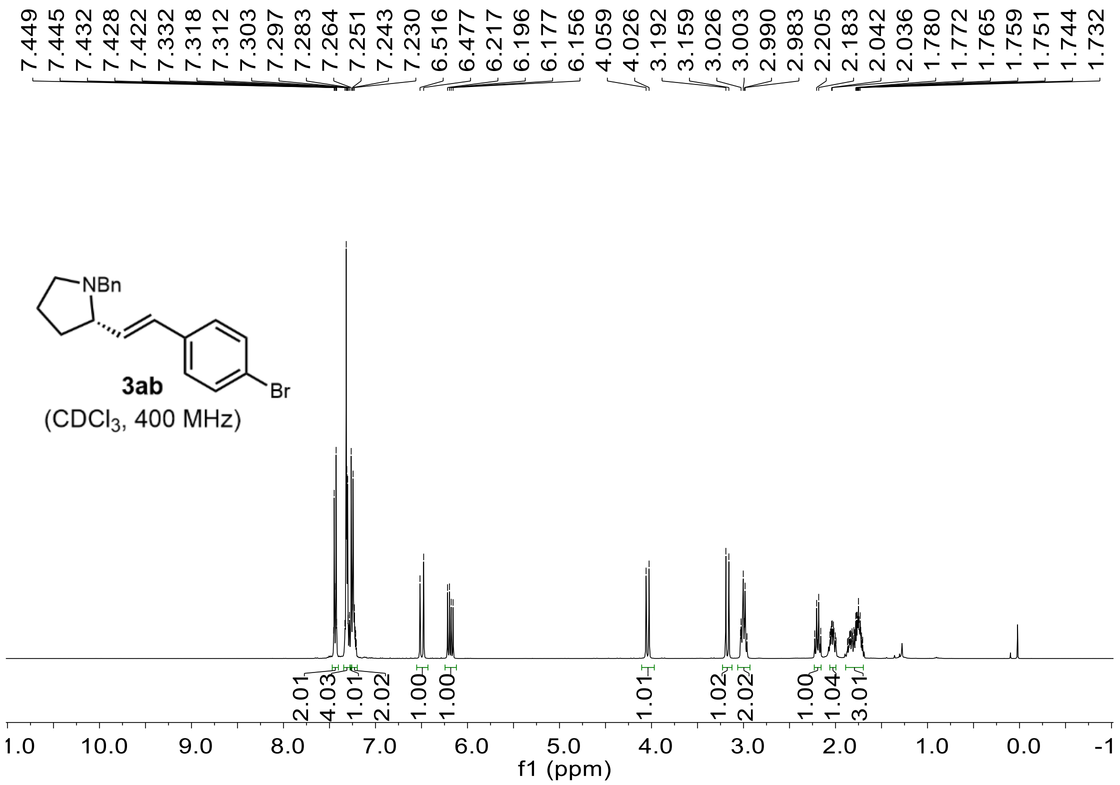
7.449 7.445 7.432 7.428 7.422 7.332 7.318 7.312 7.303 7.297 7.283 7.264 7.251 7.243 7.230 6.516 6.477 6.217 6.196 6.177 6.156 4.059 4.026 3.192 3.159 3.026 3.003 2.990 2.983 2.205 2.183 2.042 2.036 1.780 1.772 1.765 1.759 1.751 1.744 1.732
NBn
Br
3ab
(CDCl3, 400 MHz)
2.01 4.03 1.01 2.02 1.00 1.00 1.01 1.02 2.02 1.00 1.04 3.01
1.0 10.0 9.0 8.0 7.0 6.0 5.0 4.0 3.0 2.0 1.0 0.0 -1
f1 (ppm)

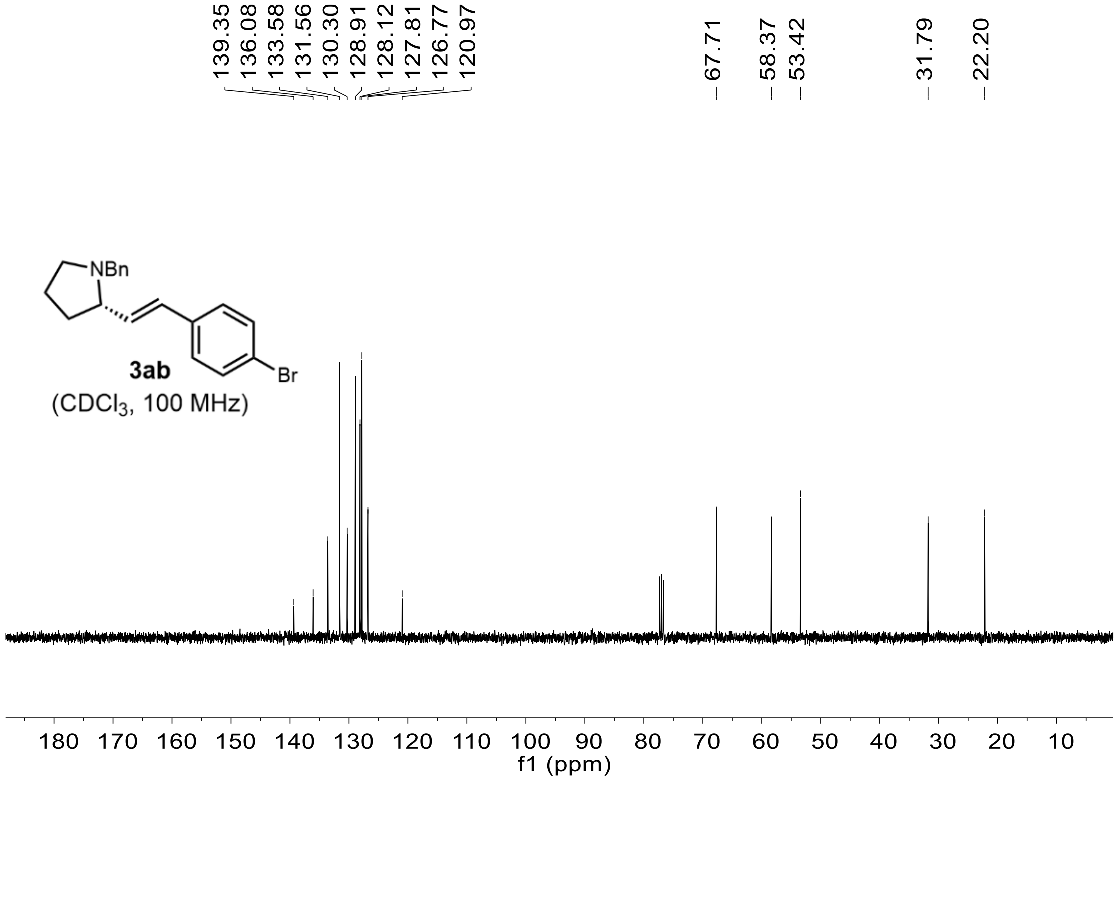
139.35 136.08 133.58 131.56 130.30 128.91 128.12 127.81 126.77 120.97
67.71 58.37 53.42 31.79 22.20
NBn
Br
3ab
(CDCl3, 100 MHz)
180 170 160 150 140 130 120 110 100 90 80 70 60 50 40 30 20 10
f1 (ppm)

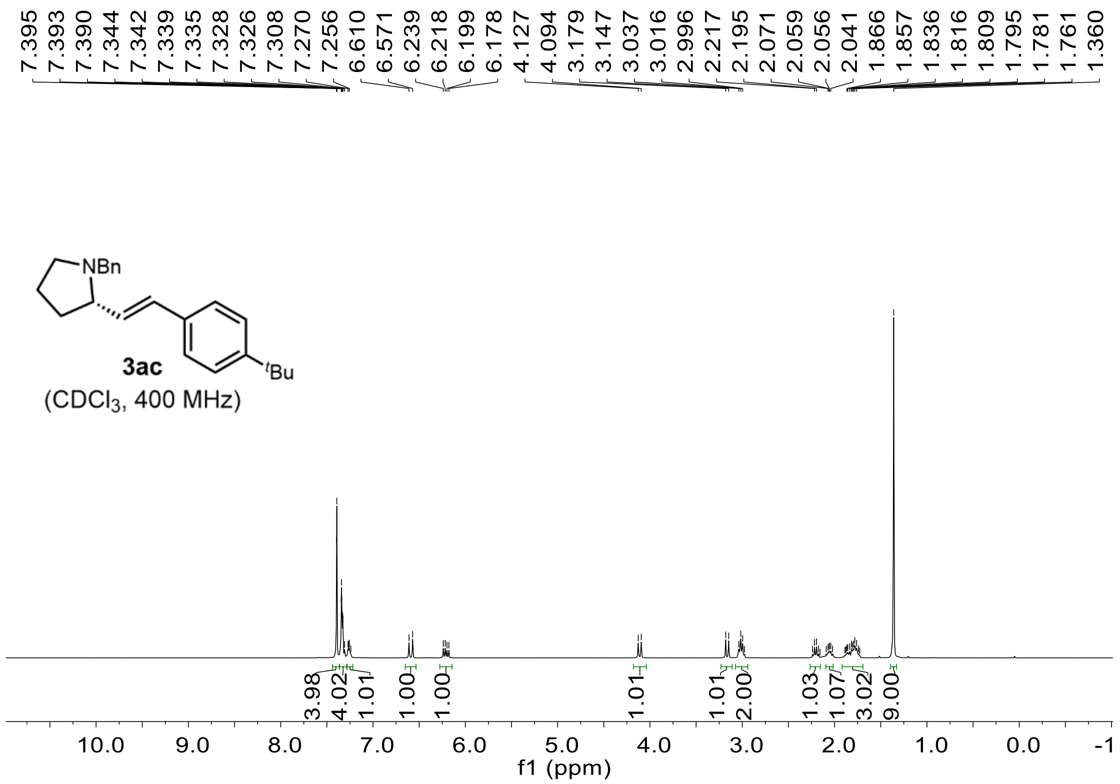
7.395 7.393 7.390 7.344 7.342 7.339 7.335 7.328 7.326 7.308 7.270 7.256 6.610 6.571 6.239 6.218 6.199 6.178 4.127 4.094 3.179 3.147 3.037 3.016 2.996 2.217 2.195 2.071 2.059 2.056 2.041 1.866 1.857 1.836 1.816 1.809 1.795 1.781 1.761 1.360
NBn
3ac
tBu
(CDCl3, 400 MHz)
3.98 4.02 1.01 1.00 1.00 1.01 1.01 2.00 1.03 1.07 3.02 9.00
10.0 9.0 8.0 7.0 6.0 5.0 4.0 3.0 2.0 1.0 0.0 -1
f1 (ppm)

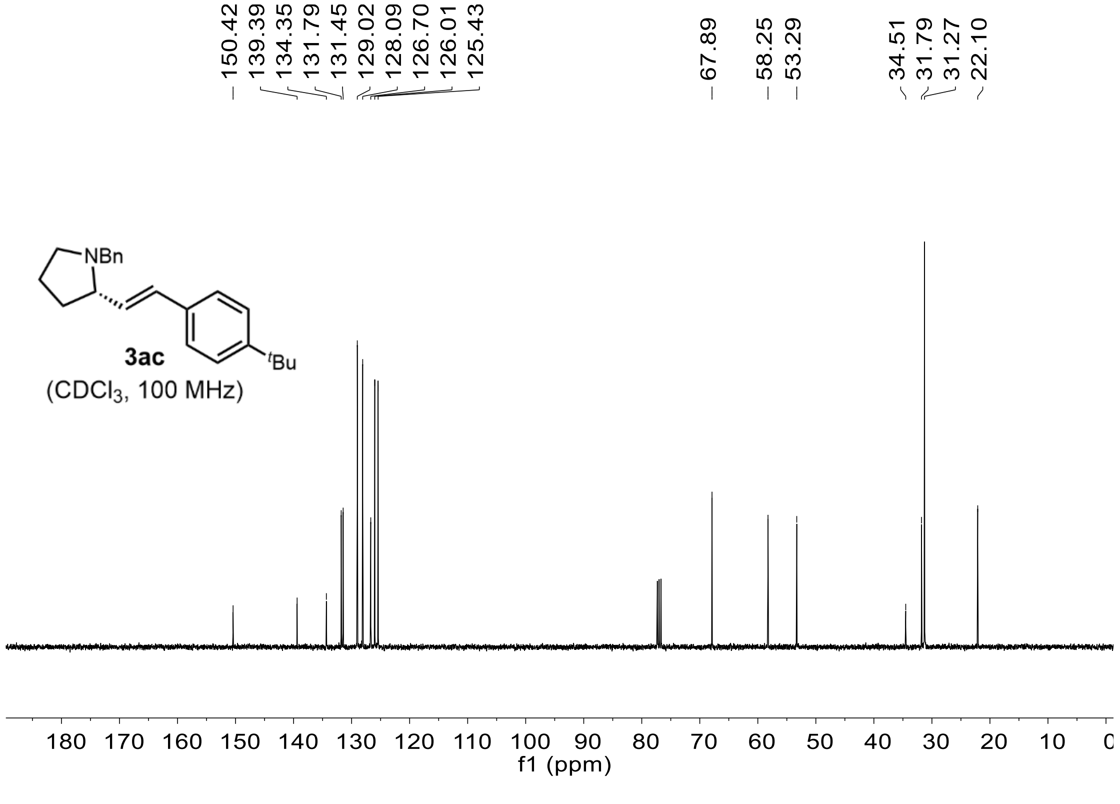
150.42 139.39 134.35 131.79 131.45 129.02 128.09 126.70 126.01 125.43 67.89 58.25 53.29 34.51 31.79 31.27 22.10
NBn
3ac
tBu
(CDCl3, 100 MHz)
180 170 160 150 140 130 120 110 100 90 80 70 60 50 40 30 20 10 0
f1 (ppm)

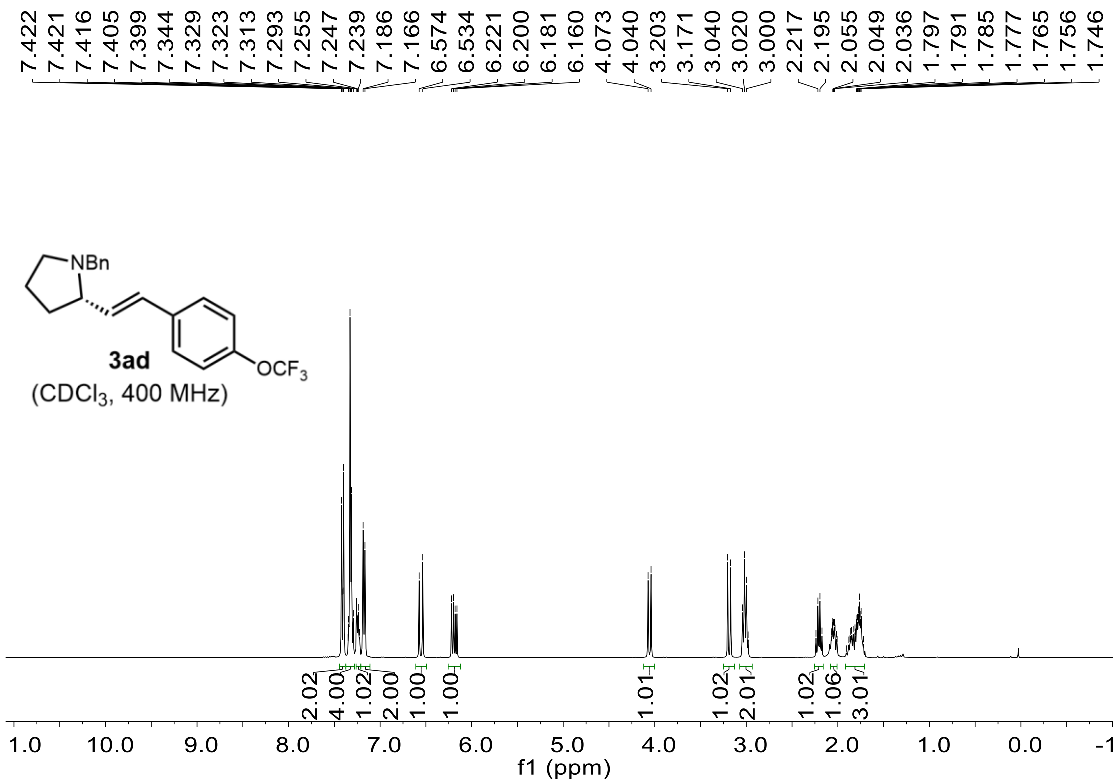
7.422
7.421
7.416
7.405
7.399
7.344
7.329
7.323
7.313
7.293
7.255
7.247
7.239
7.186
7.166
6.574
6.534
6.221
6.200
6.181
6.160
4.073
4.040
3.203
3.171
3.040
3.020
3.000
2.217
2.195
2.055
2.049
2.036
1.797
1.791
1.785
1.777
1.765
1.756
1.746
NBn
OCF3
3ad
(CDCl3, 400 MHz)
2.02
4.00
1.02
2.00
1.00
1.00
1.01
1.02
2.01
1.02
1.06
3.01
1.0
10.0
9.0
8.0
7.0
6.0
5.0
4.0
3.0
2.0
1.0
0.0
-1
f1 (ppm)

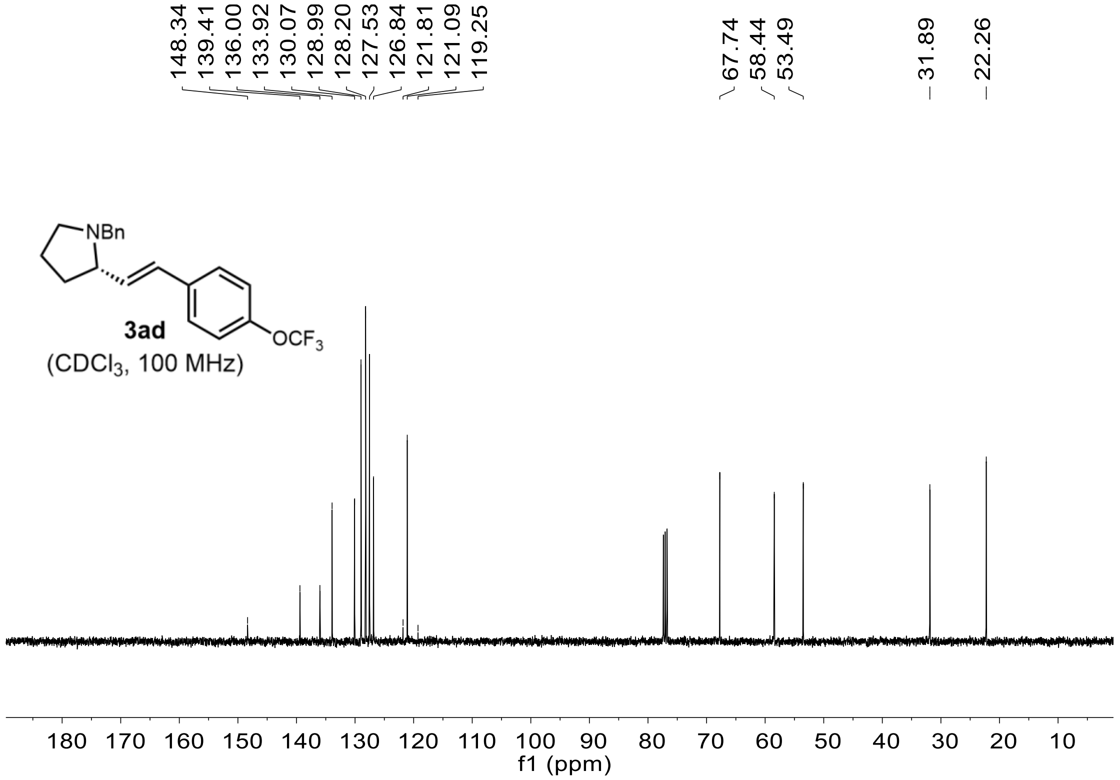
148.34
139.41
136.00
133.92
130.07
128.99
128.20
127.53
126.84
121.81
121.09
119.25
67.74
58.44
53.49
31.89
22.26
NBn
OCF3
3ad
(CDCl3, 100 MHz)
180
170
160
150
140
130
120
110
100
90
80
70
60
50
40
30
20
10
f1 (ppm)

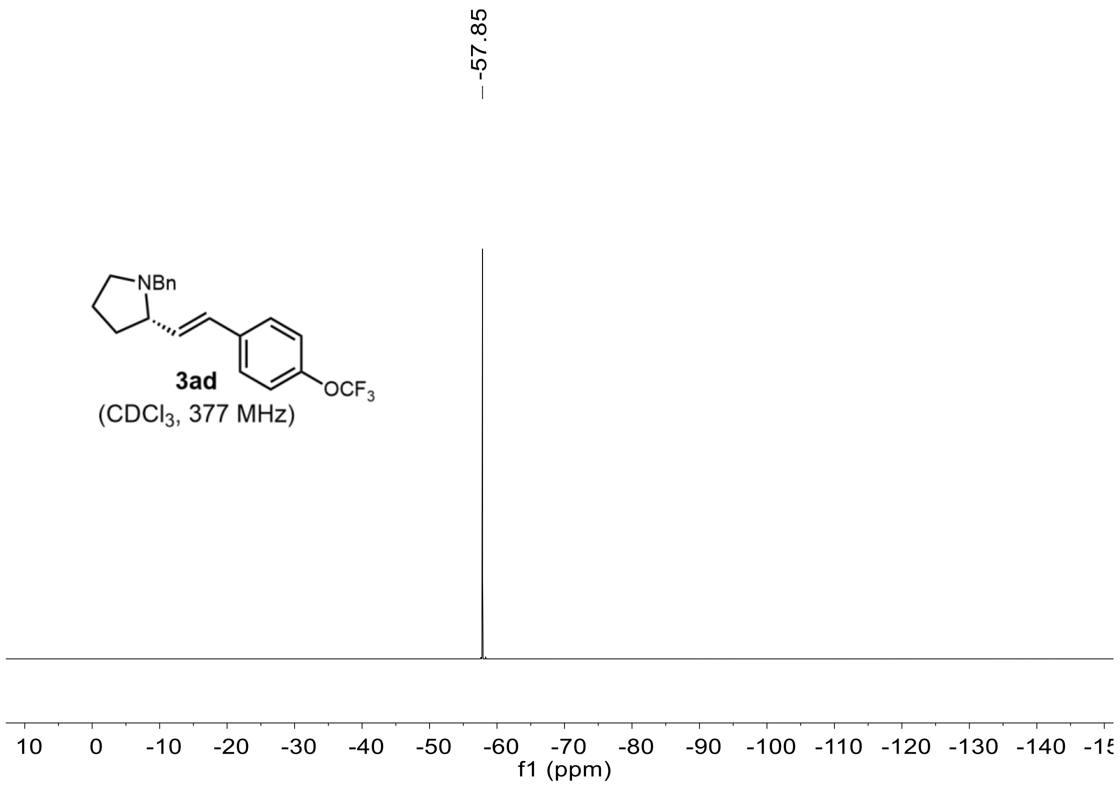
-57.85
NBn
3ad
OCF3
(CDCl3, 377 MHz)
10 0 -10 -20 -30 -40 -50 -60 -70 -80 -90 -100 -110 -120 -130 -140
f1 (ppm)

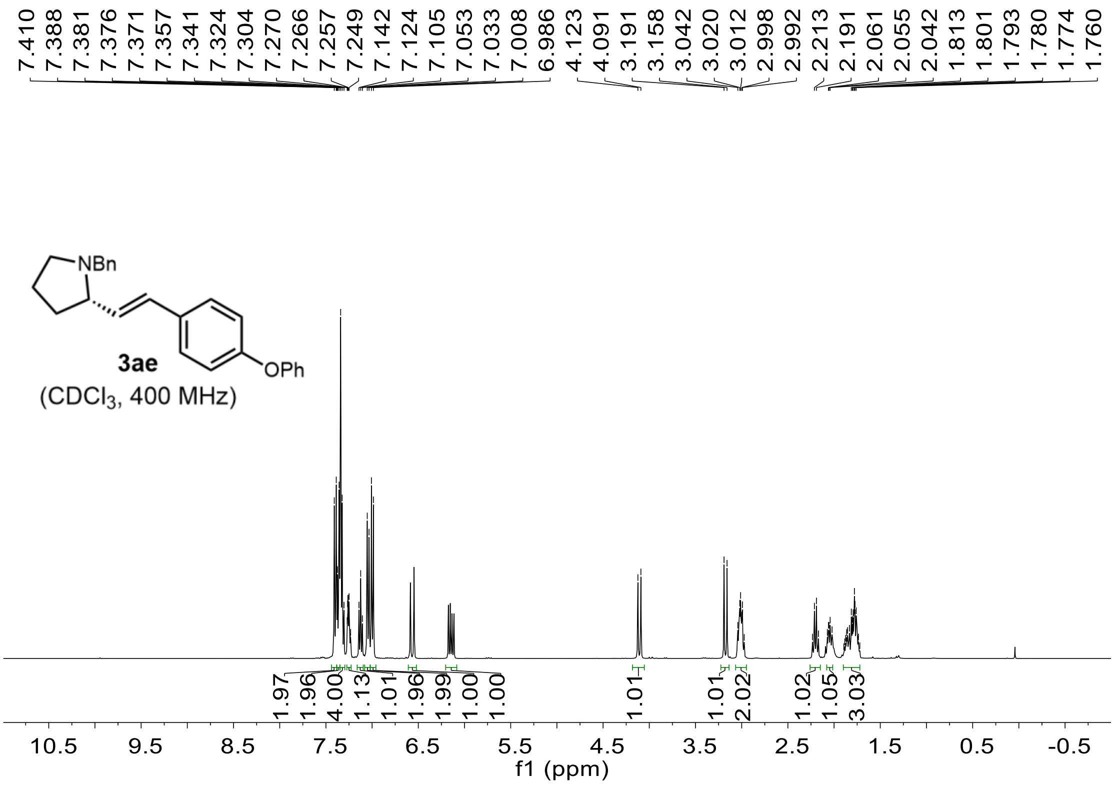
7.410 7.388 7.381 7.376 7.371 7.357 7.341 7.324 7.304 7.270 7.266 7.257 7.249 7.142 7.124 7.105 7.053 7.033 7.008 6.986 4.123 4.091 3.191 3.158 3.042 3.020 3.012 2.998 2.992 2.213 2.191 2.061 2.055 2.042 1.813 1.801 1.793 1.780 1.774 1.760
NBn
OPh
3ae
(CDCl$_3$, 400 MHz)
1.97 1.96 4.00 1.13 1.01 1.96 1.99 1.00 1.00 1.01 1.01 2.02 1.02 1.05 3.03
10.5 9.5 8.5 7.5 6.5 5.5 4.5 3.5 2.5 1.5 0.5 -0.5
f1 (ppm)

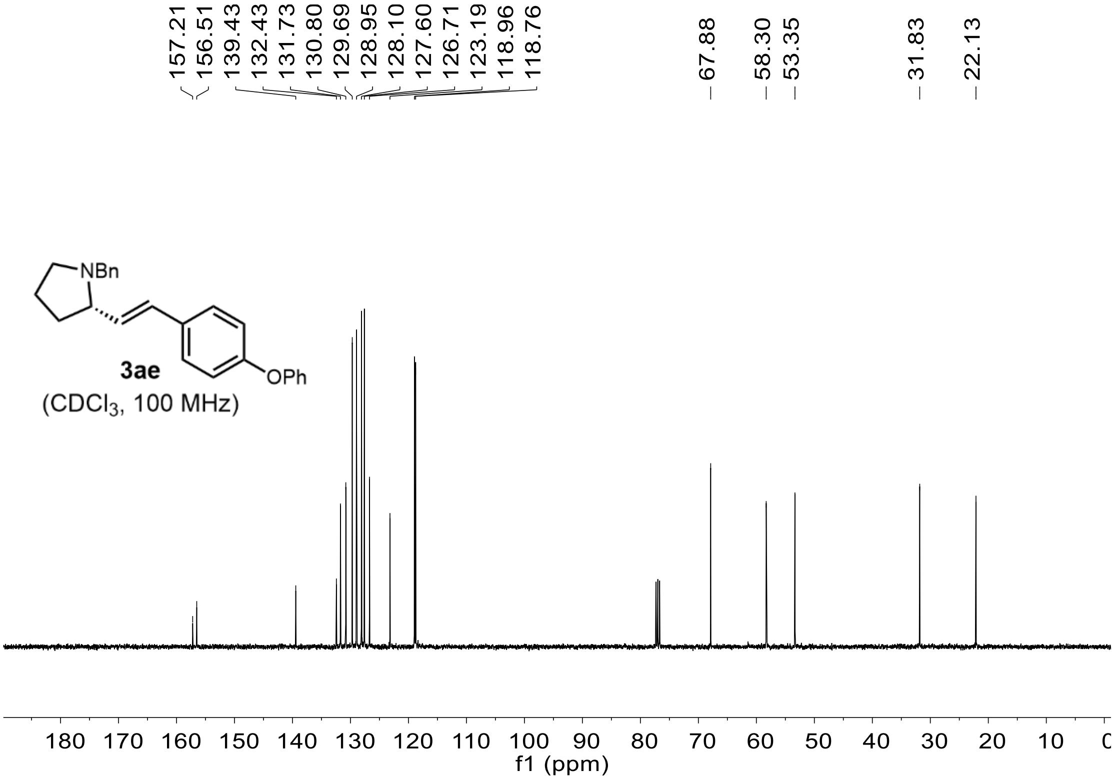
157.21 156.51 139.43 132.43 131.73 130.80 129.69 128.95 128.10 127.60 126.71 123.19 118.96 118.76 67.88 58.30 53.35 31.83 22.13
NBn
OPh
3ae
(CDCl$_3$, 100 MHz)
180 170 160 150 140 130 120 110 100 90 80 70 60 50 40 30 20 10 0
f1 (ppm)

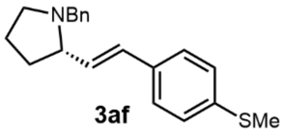

3af
($CDCl_3$, 400 MHz)

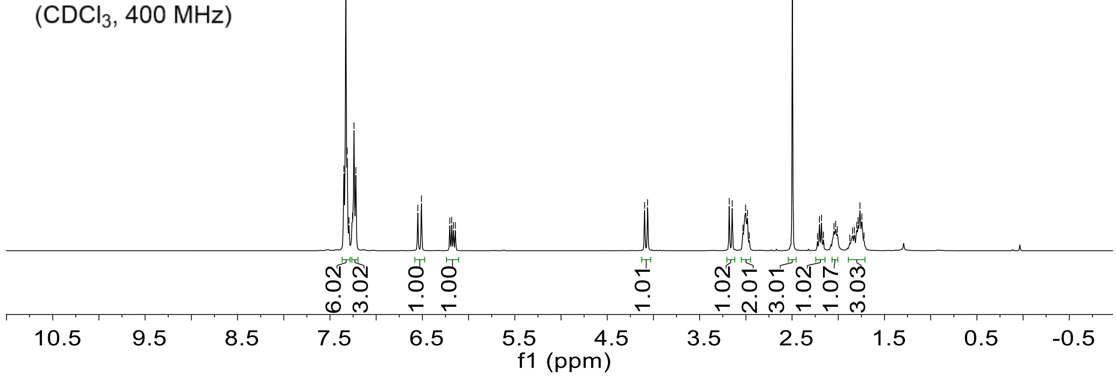

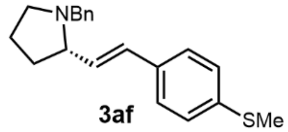

3af
($CDCl_3$, 100 MHz)

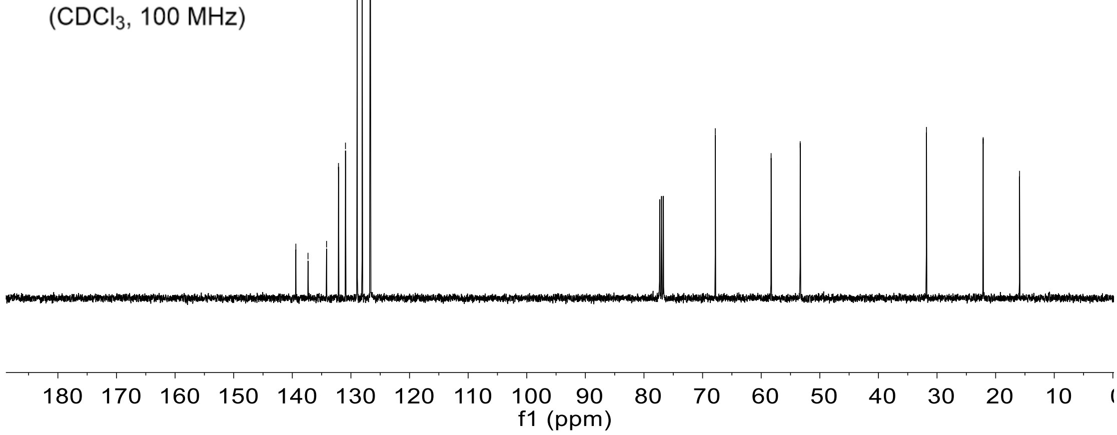

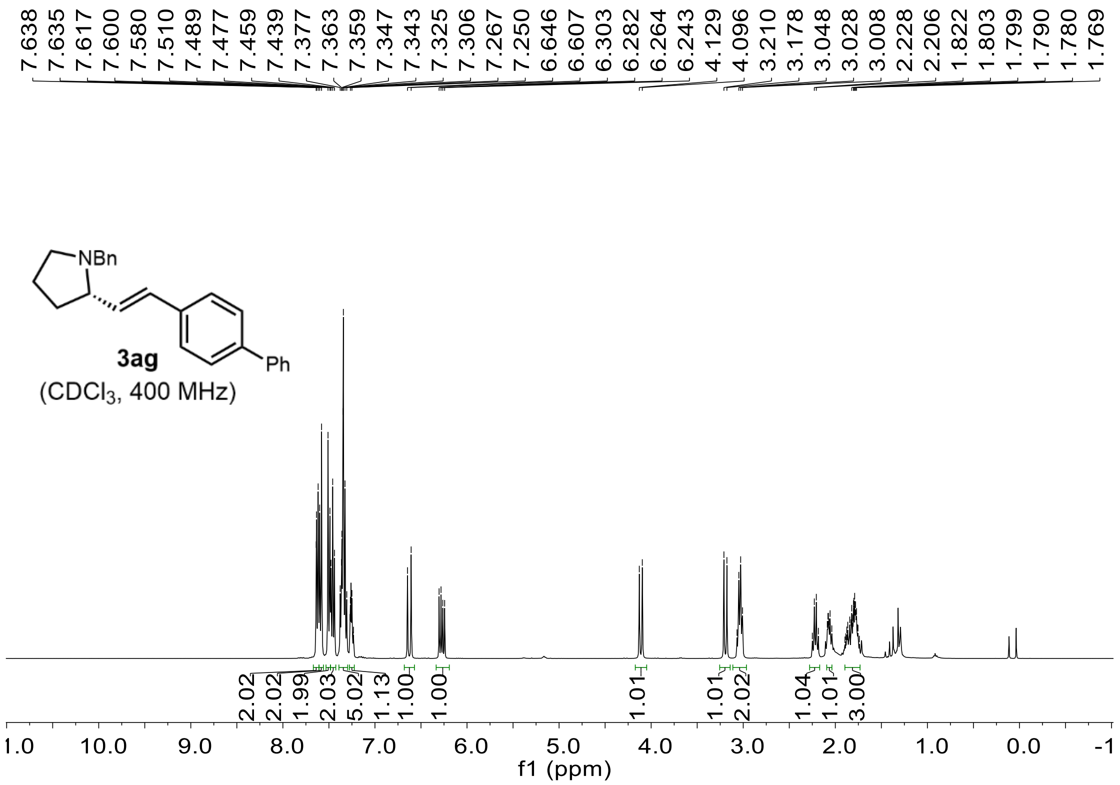
7.638 7.635 7.617 7.600 7.580 7.510 7.489 7.477 7.459 7.439 7.377 7.363 7.359 7.347 7.343 7.325 7.306 7.267 7.250 6.646 6.607 6.303 6.282 6.264 6.243 4.129 4.096 3.210 3.178 3.048 3.028 3.008 2.228 2.206 1.822 1.803 1.799 1.790 1.780 1.769
NBn
Ph
3ag
(CDCl3, 400 MHz)
2.02 2.02 1.99 2.03 5.02 1.13 1.00 1.00 1.01 1.01 2.02 1.04 1.01 3.00
f1 (ppm)

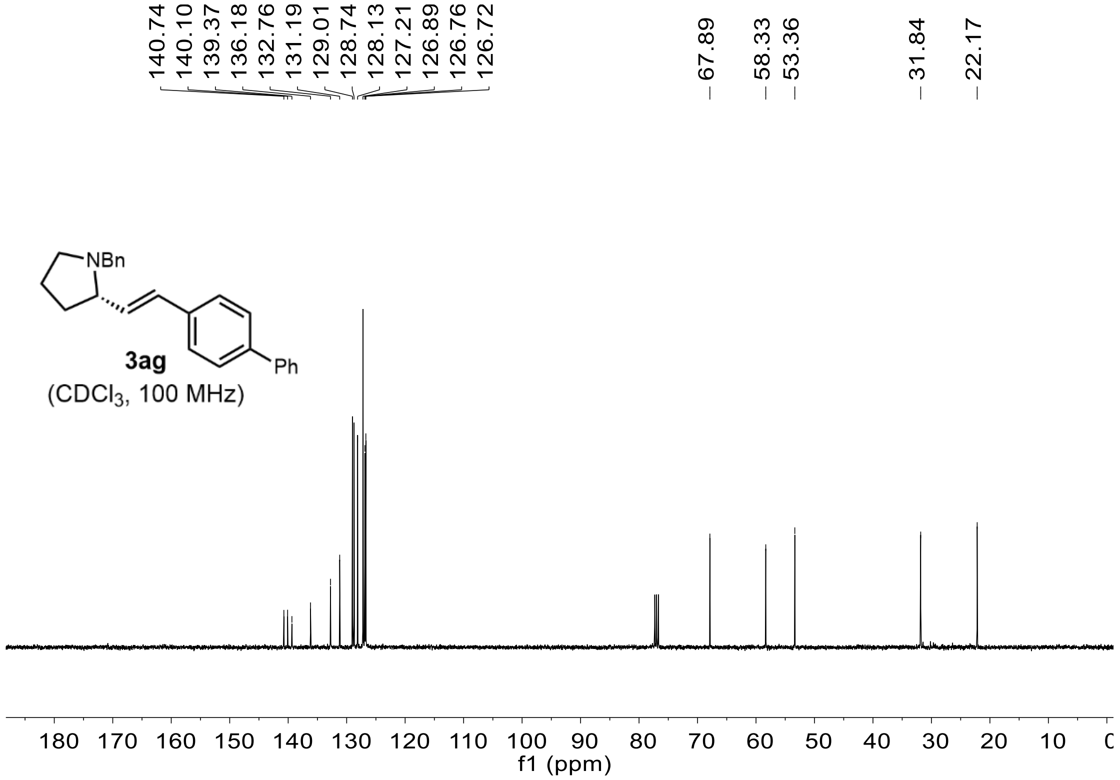
140.74 140.10 139.37 136.18 132.76 131.19 129.01 128.74 128.13 127.21 126.89 126.76 126.72
67.89 58.33 53.36 31.84 22.17
NBn
Ph
3ag
(CDCl3, 100 MHz)
f1 (ppm)

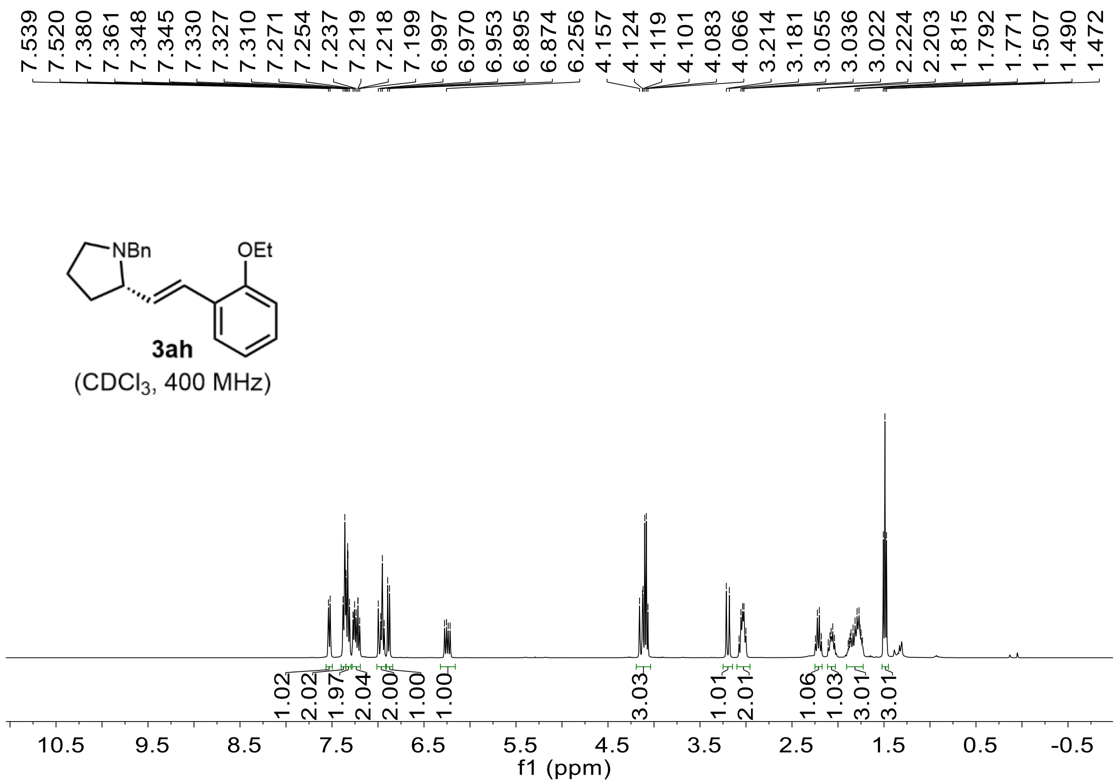
7.539 7.520 7.380 7.361 7.348 7.345 7.330 7.327 7.310 7.271 7.254 7.237 7.219 7.218 7.199 6.997 6.970 6.953 6.895 6.874 6.256 4.157 4.124 4.119 4.101 4.083 4.066 3.214 3.181 3.055 3.036 3.022 2.224 2.203 1.815 1.792 1.771 1.507 1.490 1.472
NBn
OEt
3ah
($CDCl_3$, 400 MHz)
1.02 2.02 1.97 2.04 2.00 1.00 1.00 3.03 1.01 2.01 1.06 1.03 3.01 3.01
10.5 9.5 8.5 7.5 6.5 5.5 4.5 3.5 2.5 1.5 0.5 -0.5
f1 (ppm)

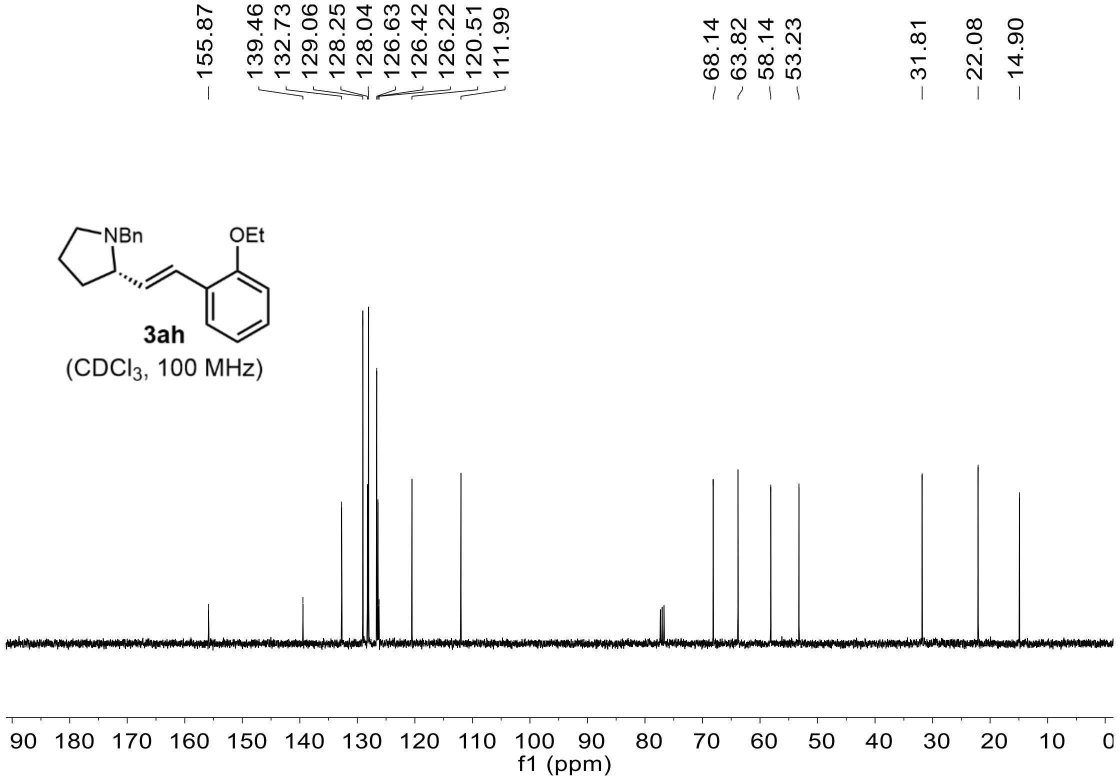
155.87 139.46 132.73 129.06 128.25 128.04 126.63 126.42 126.22 120.51 111.99 68.14 63.82 58.14 53.23 31.81 22.08 14.90
NBn
OEt
3ah
($CDCl_3$, 100 MHz)
90 180 170 160 150 140 130 120 110 100 90 80 70 60 50 40 30 20 10 0
f1 (ppm)

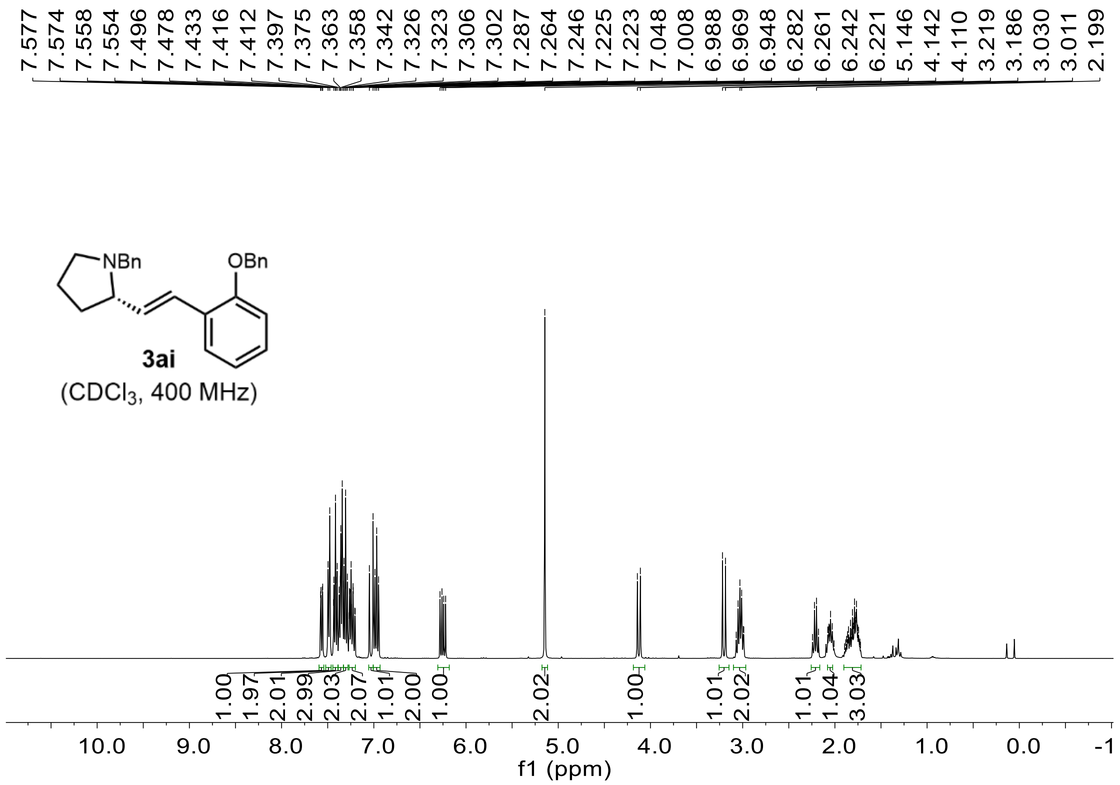
7.577
7.574
7.558
7.554
7.496
7.478
7.433
7.416
7.412
7.397
7.375
7.363
7.358
7.342
7.326
7.323
7.306
7.302
7.287
7.264
7.246
7.225
7.223
7.048
7.008
6.988
6.969
6.948
6.282
6.261
6.242
6.221
5.146
4.142
4.110
3.219
3.186
3.030
3.011
2.199
NBn
OBn
3ai
(CDCl3, 400 MHz)
1.00
1.97
2.01
2.99
2.03
2.07
1.01
2.00
1.00
2.02
1.00
1.01
2.02
1.01
1.04
3.03
10.0 9.0 8.0 7.0 6.0 5.0 4.0 3.0 2.0 1.0 0.0 -1
f1 (ppm)

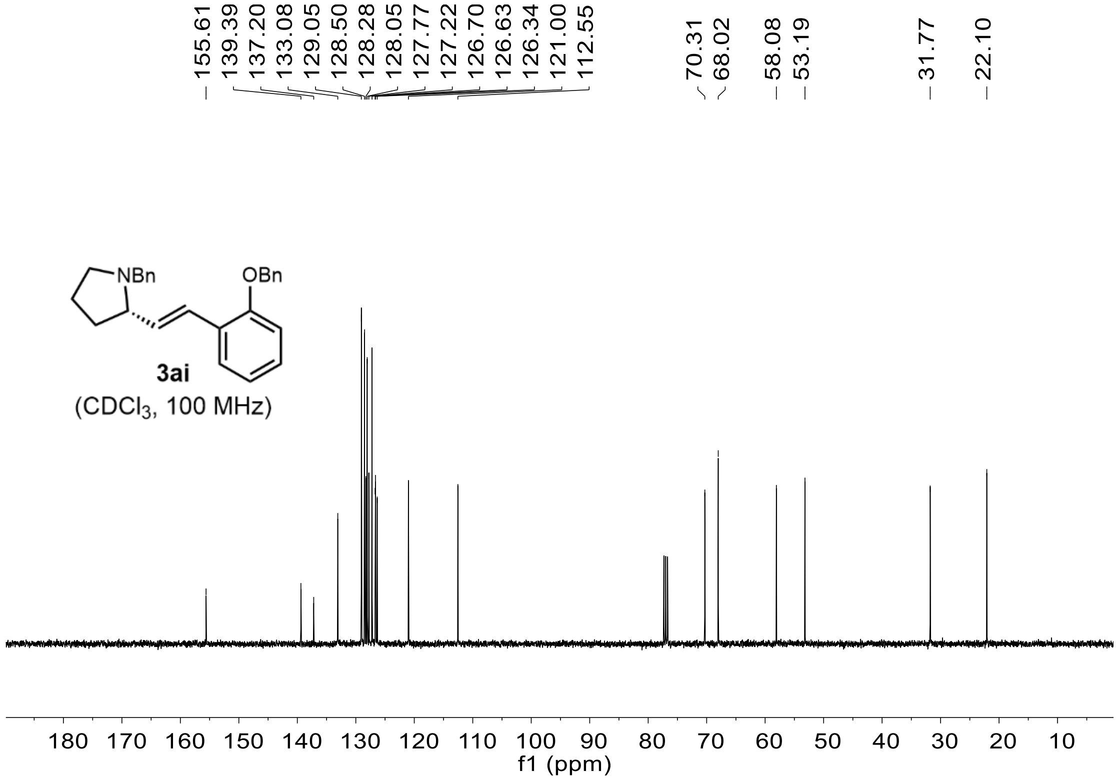
155.61
139.39
137.20
133.08
129.05
128.50
128.28
128.05
127.77
127.22
126.70
126.63
126.34
121.00
112.55
70.31
68.02
58.08
53.19
31.77
22.10
NBn
OBn
3ai
(CDCl3, 100 MHz)
180 170 160 150 140 130 120 110 100 90 80 70 60 50 40 30 20 10
f1 (ppm)

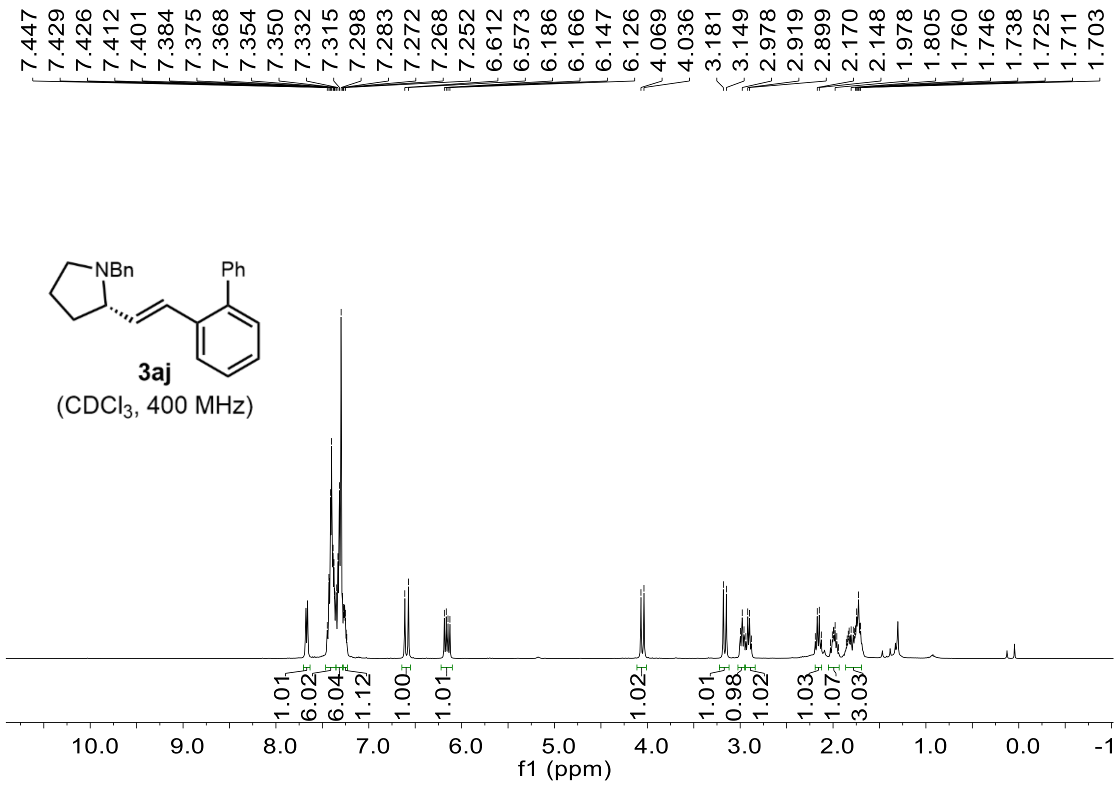
7.447 7.429 7.426 7.412 7.401 7.384 7.375 7.368 7.354 7.350 7.332 7.315 7.298 7.283 7.272 7.268 7.252 6.612 6.573 6.186 6.166 6.147 6.126 4.069 4.036 3.181 3.149 2.978 2.919 2.899 2.170 2.148 1.978 1.805 1.760 1.746 1.738 1.725 1.711 1.703
NBn
Ph
3aj
(CDCl3, 400 MHz)
1.01 6.02 6.04 1.12 1.00 1.01 1.02 1.01 0.98 1.02 1.03 1.07 3.03
10.0 9.0 8.0 7.0 6.0 5.0 4.0 3.0 2.0 1.0 0.0 -1
f1 (ppm)

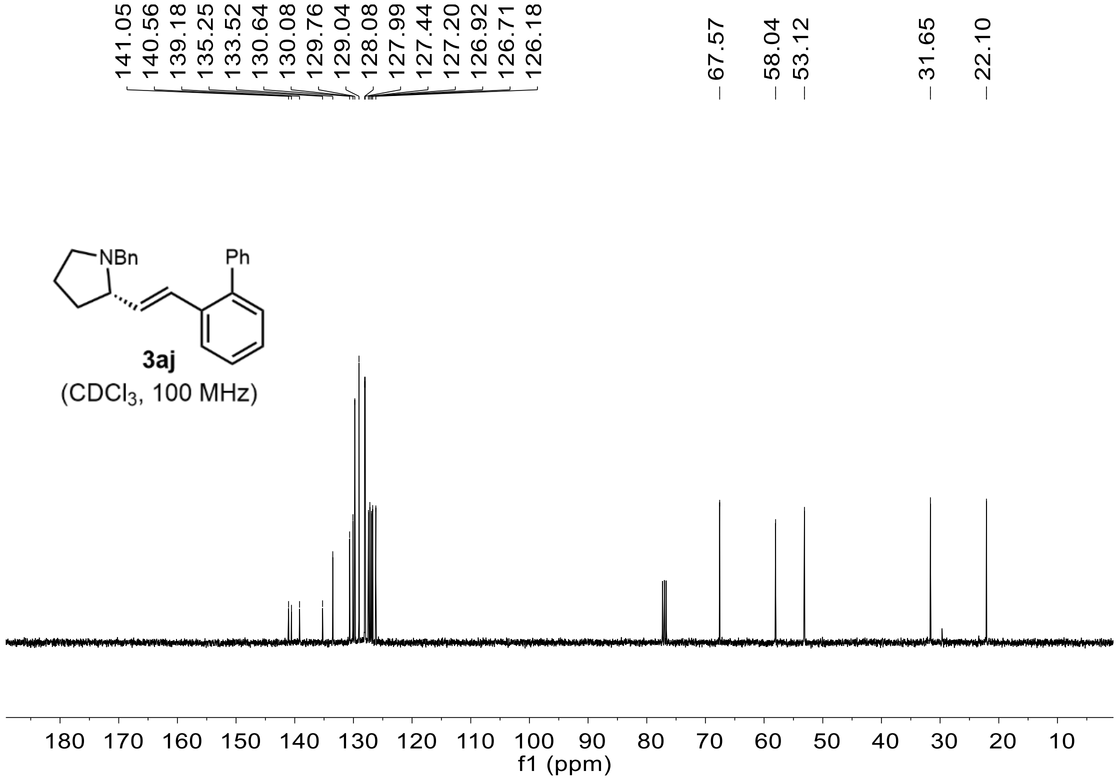
141.05 140.56 139.18 135.25 133.52 130.64 130.08 129.76 129.04 128.08 127.99 127.44 127.20 126.92 126.71 126.18
67.57 58.04 53.12 31.65 22.10
NBn
Ph
3aj
(CDCl3, 100 MHz)
180 170 160 150 140 130 120 110 100 90 80 70 60 50 40 30 20 10
f1 (ppm)

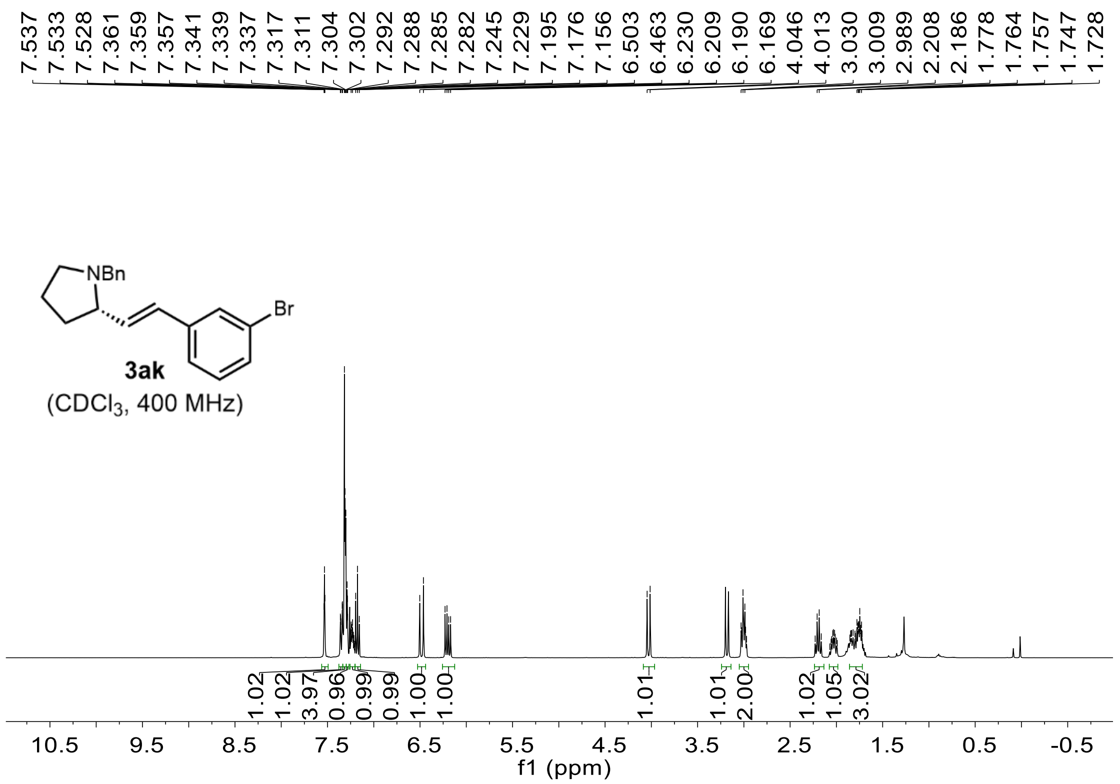
7.537 7.533 7.528 7.361 7.359 7.357 7.341 7.339 7.337 7.317 7.311 7.304 7.302 7.292 7.288 7.285 7.282 7.245 7.229 7.195 7.176 7.156 6.503 6.463 6.230 6.209 6.190 6.169 4.046 4.013 3.030 3.009 2.989 2.208 2.186 1.778 1.764 1.757 1.747 1.728
NBn
Br
3ak
(CDCl3, 400 MHz)
1.02 1.02 3.97 0.96 0.99 0.99 1.00 1.00 1.01 1.01 2.00 1.02 1.05 3.02
10.5 9.5 8.5 7.5 6.5 5.5 4.5 3.5 2.5 1.5 0.5 -0.5
f1 (ppm)

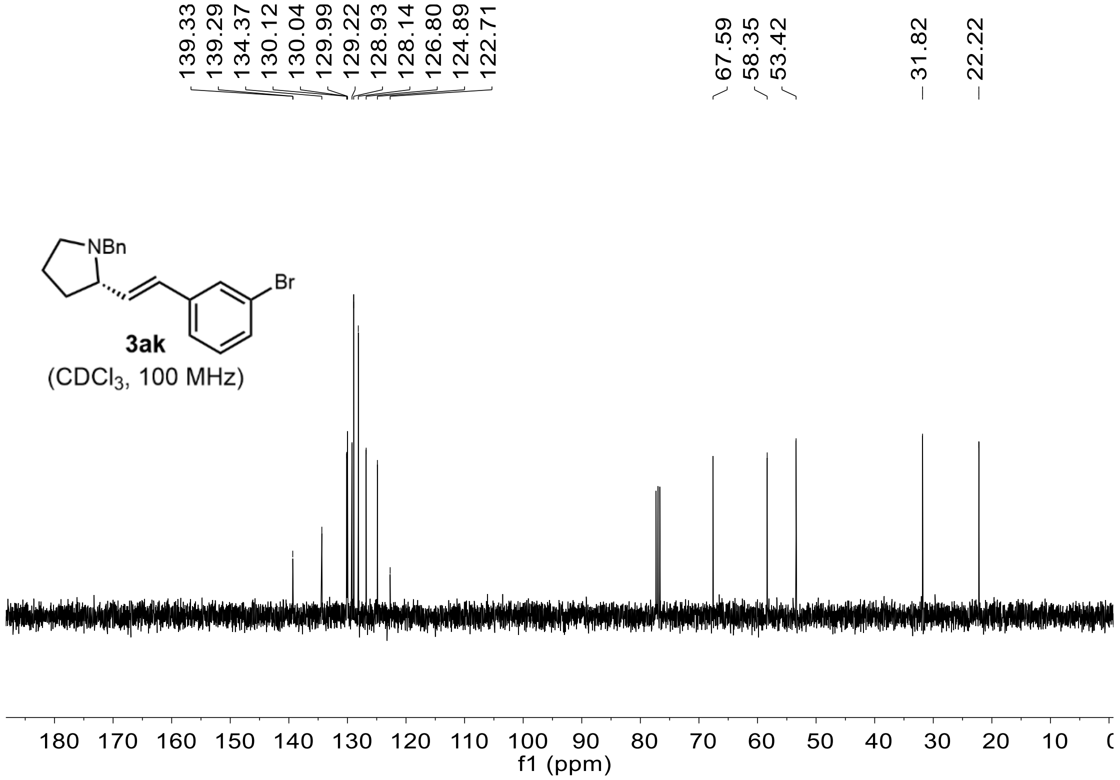
139.33 139.29 134.37 130.12 130.04 129.99 129.22 128.93 128.14 126.80 124.89 122.71
67.59 58.35 53.42
31.82
22.22
NBn
Br
3ak
(CDCl3, 100 MHz)
180 170 160 150 140 130 120 110 100 90 80 70 60 50 40 30 20 10
f1 (ppm)

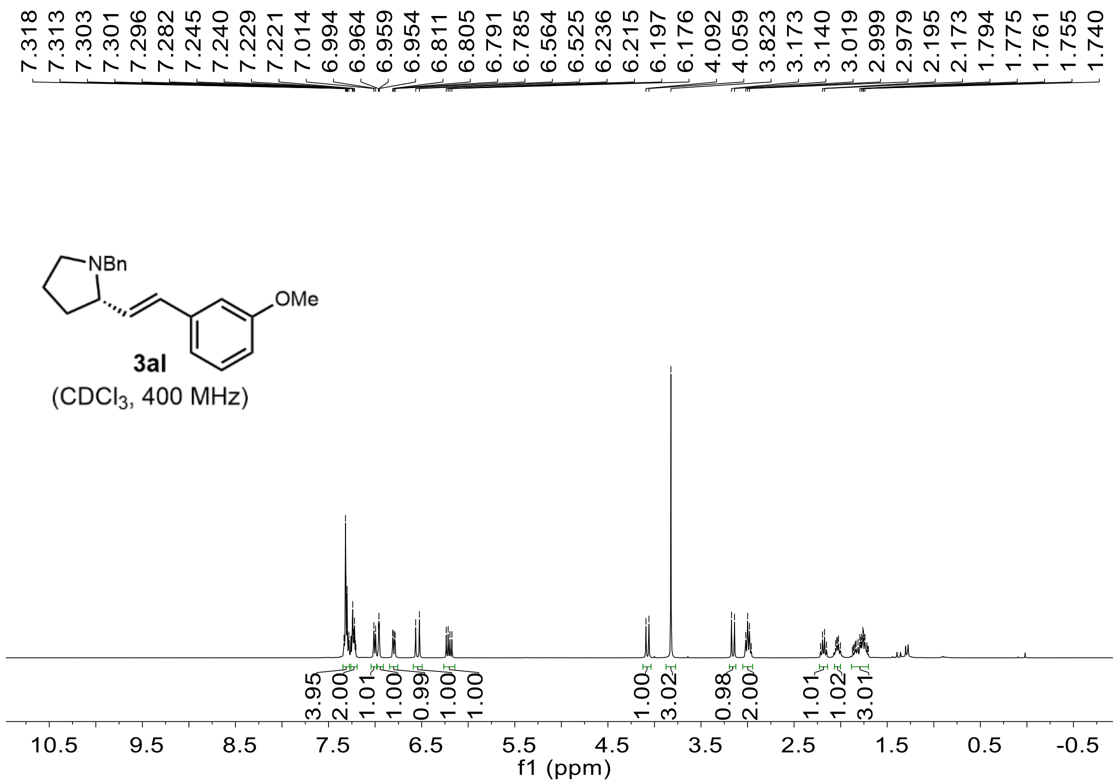
7.318 7.313 7.303 7.301 7.296 7.282 7.245 7.240 7.229 7.221 7.014 6.994 6.964 6.959 6.954 6.811 6.805 6.791 6.785 6.564 6.525 6.236 6.215 6.197 6.176 4.092 4.059 3.823 3.173 3.140 3.019 2.999 2.979 2.195 2.173 1.794 1.775 1.761 1.755 1.740
NBn
OMe
3al
(CDCl$_3$, 400 MHz)
3.95 2.00 1.01 1.00 0.99 1.00 1.00 1.00 3.02 0.98 2.00 1.01 1.02 3.01
10.5 9.5 8.5 7.5 6.5 5.5 4.5 3.5 2.5 1.5 0.5 -0.5
f1 (ppm)

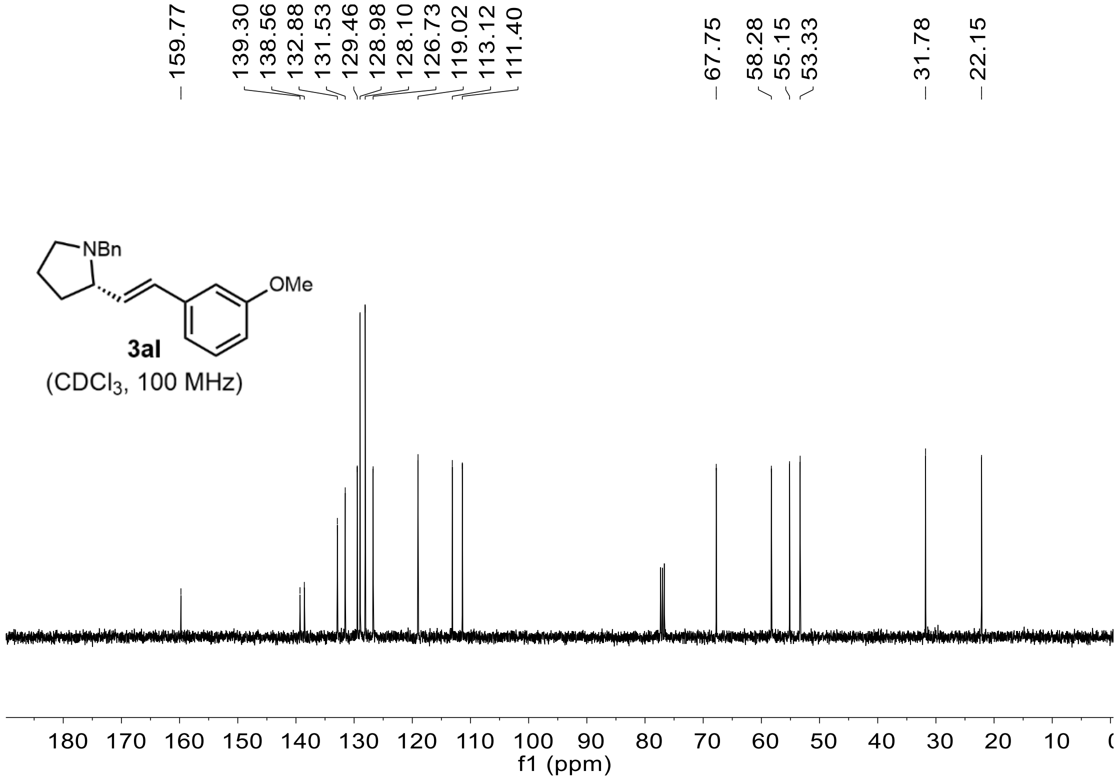
159.77 139.30 138.56 132.88 131.53 129.46 128.98 128.10 126.73 119.02 113.12 111.40 67.75 58.28 55.15 53.33 31.78 22.15
NBn
OMe
3al
(CDCl$_3$, 100 MHz)
180 170 160 150 140 130 120 110 100 90 80 70 60 50 40 30 20 10
f1 (ppm)

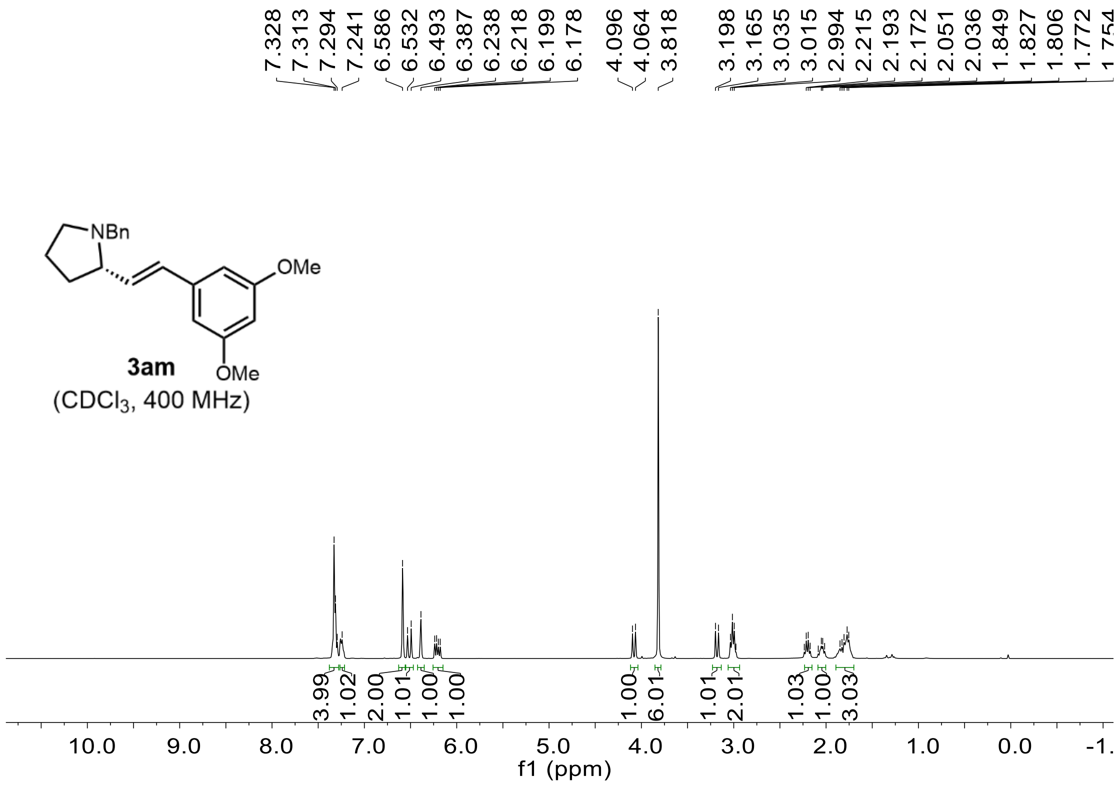
7.328
7.313
7.294
7.241
6.586
6.532
6.493
6.387
6.238
6.218
6.199
6.178
4.096
4.064
3.818
3.198
3.165
3.035
3.015
2.994
2.215
2.193
2.172
2.051
2.036
1.849
1.827
1.806
1.772
NBn
OMe
OMe
3am
(CDCl3, 400 MHz)
3.99
1.02
2.00
1.01
1.00
1.00
1.00
6.01
1.01
2.01
1.03
1.00
3.03
10.0
9.0
8.0
7.0
6.0
5.0
4.0
3.0
2.0
1.0
0.0
f1 (ppm)

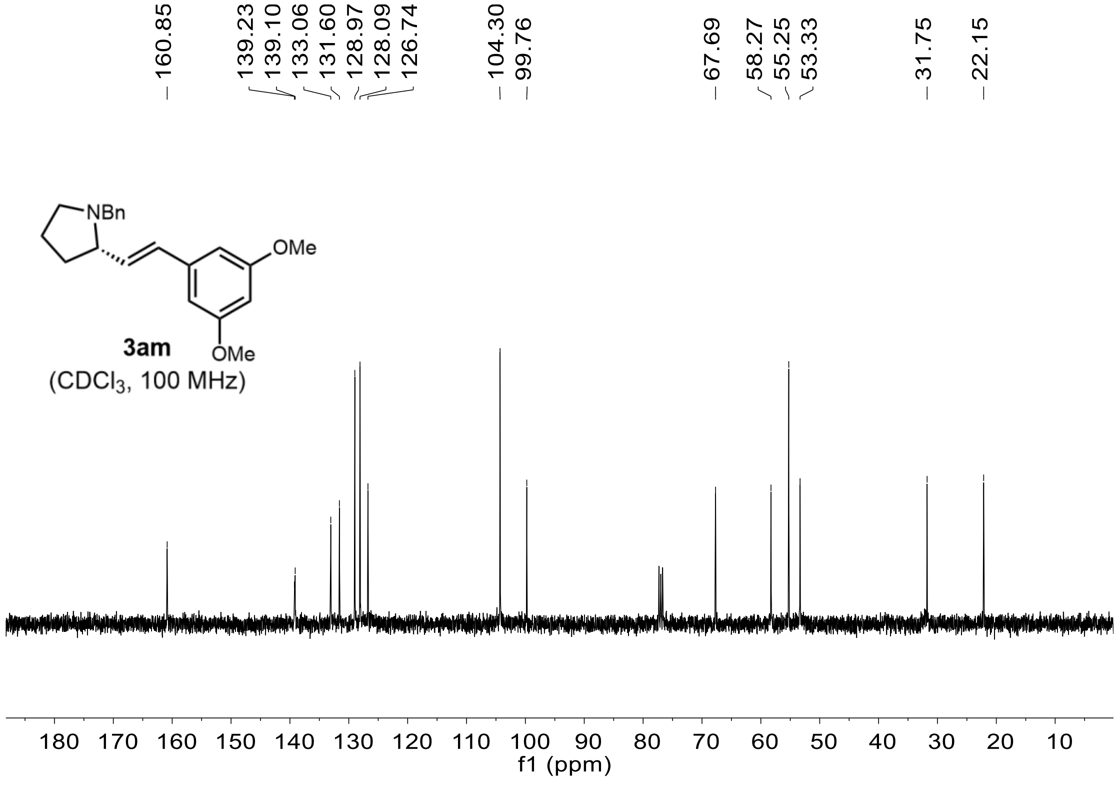
160.85
139.23
139.10
133.06
131.60
128.97
128.09
126.74
104.30
99.76
67.69
58.27
55.25
53.33
31.75
22.15
NBn
OMe
OMe
3am
(CDCl3, 100 MHz)
180
170
160
150
140
130
120
110
100
90
80
70
60
50
40
30
20
10
f1 (ppm)

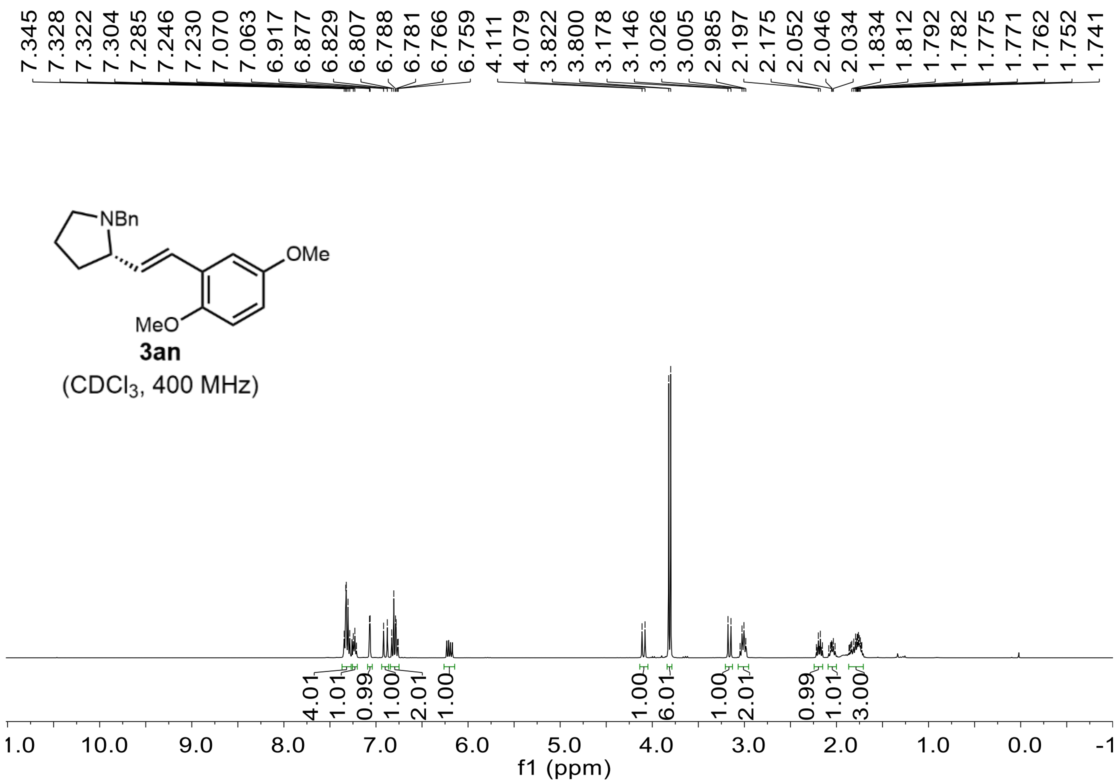
7.345
7.328
7.322
7.304
7.285
7.246
7.230
7.070
7.063
6.917
6.877
6.829
6.807
6.788
6.781
6.766
6.759
4.111
4.079
3.822
3.800
3.178
3.146
3.026
3.005
2.985
2.197
2.175
2.052
2.046
2.034
1.834
1.812
1.792
1.782
1.775
1.771
1.762
1.752
1.741
NBn
OMe
MeO
3an
(CDCl3, 400 MHz)
4.01
1.01
0.99
1.00
2.01
1.00
1.00
6.01
1.00
2.01
0.99
1.01
3.00
f1 (ppm)

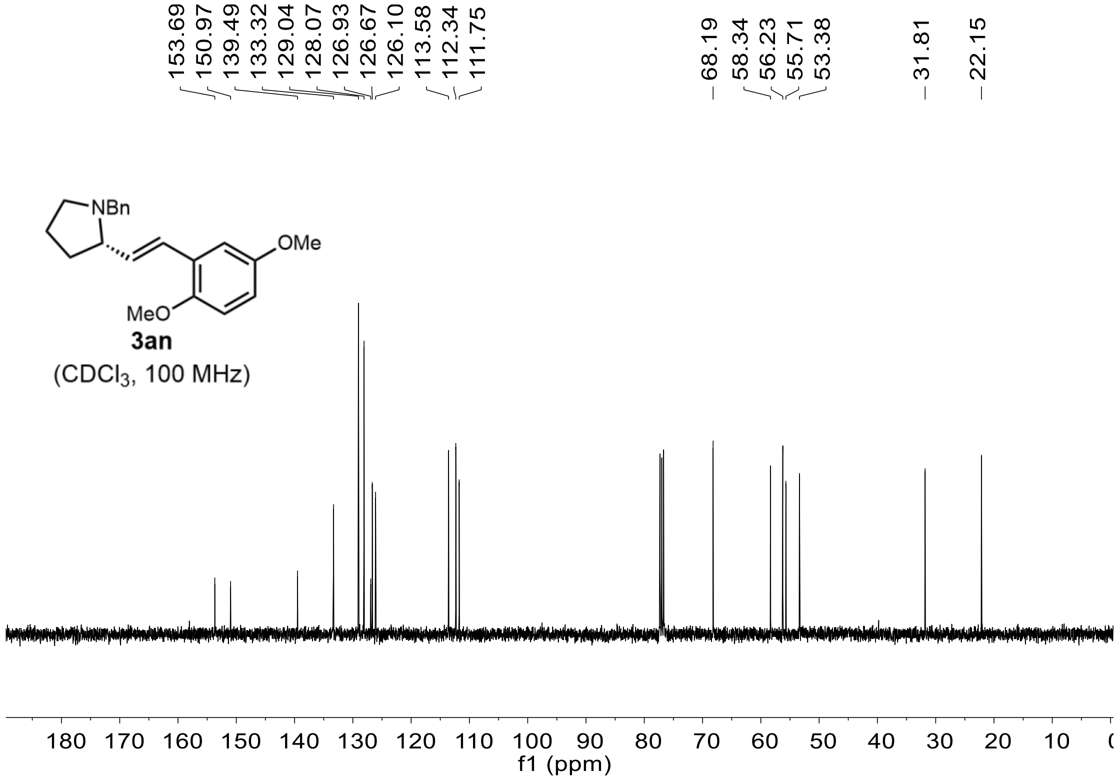
153.69
150.97
139.49
133.32
129.04
128.07
126.93
126.67
126.10
113.58
112.34
111.75
68.19
58.34
56.23
55.71
53.38
31.81
22.15
NBn
OMe
MeO
3an
(CDCl3, 100 MHz)
f1 (ppm)

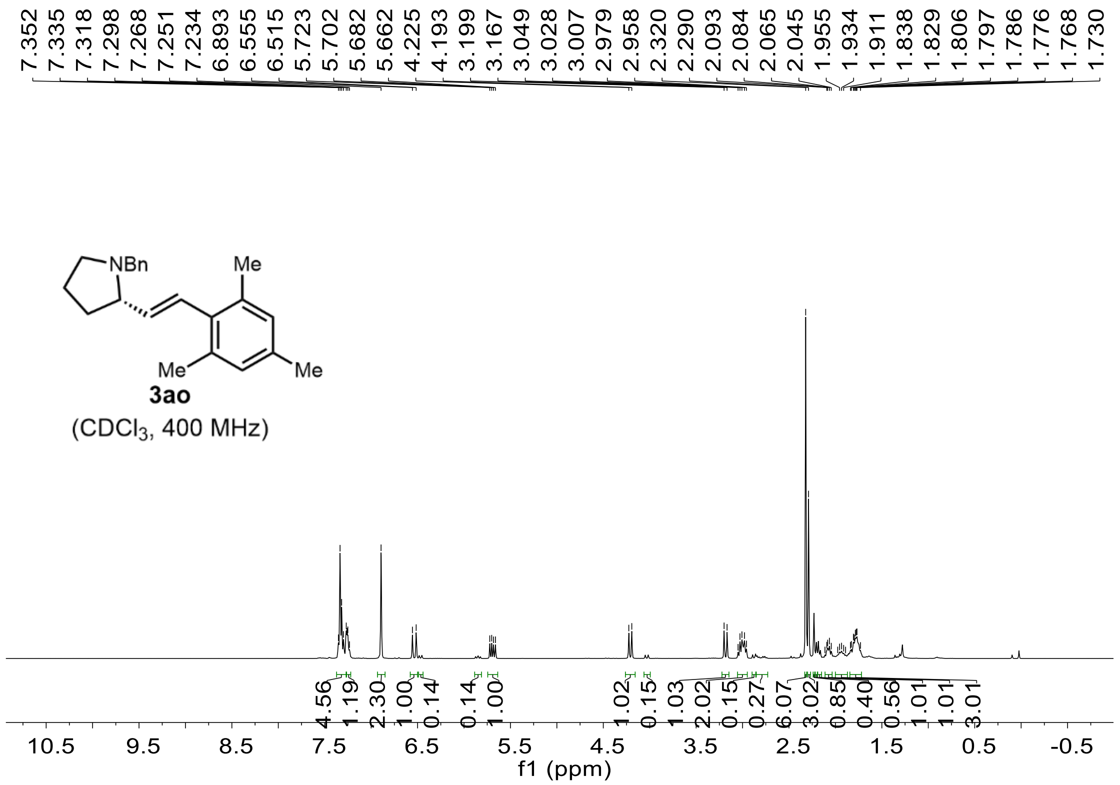
7.352
7.335
7.318
7.298
7.268
7.251
7.234
6.893
6.555
6.515
5.723
5.702
5.682
5.662
4.225
4.193
3.199
3.167
3.049
3.028
3.007
2.979
2.958
2.320
2.290
2.093
2.084
2.065
2.045
1.955
1.934
1.911
1.838
1.829
1.806
1.797
1.786
1.776
1.768
1.730
NBn
Me
Me
Me
3ao
(CDCl3, 400 MHz)
4.56
1.19
2.30
1.00
0.14
0.14
1.00
1.02
0.15
1.03
2.02
0.15
0.27
6.07
3.02
0.85
0.40
0.56
1.01
1.01
3.01
10.5
9.5
8.5
7.5
6.5
5.5
4.5
3.5
2.5
1.5
0.5
-0.5
f1 (ppm)

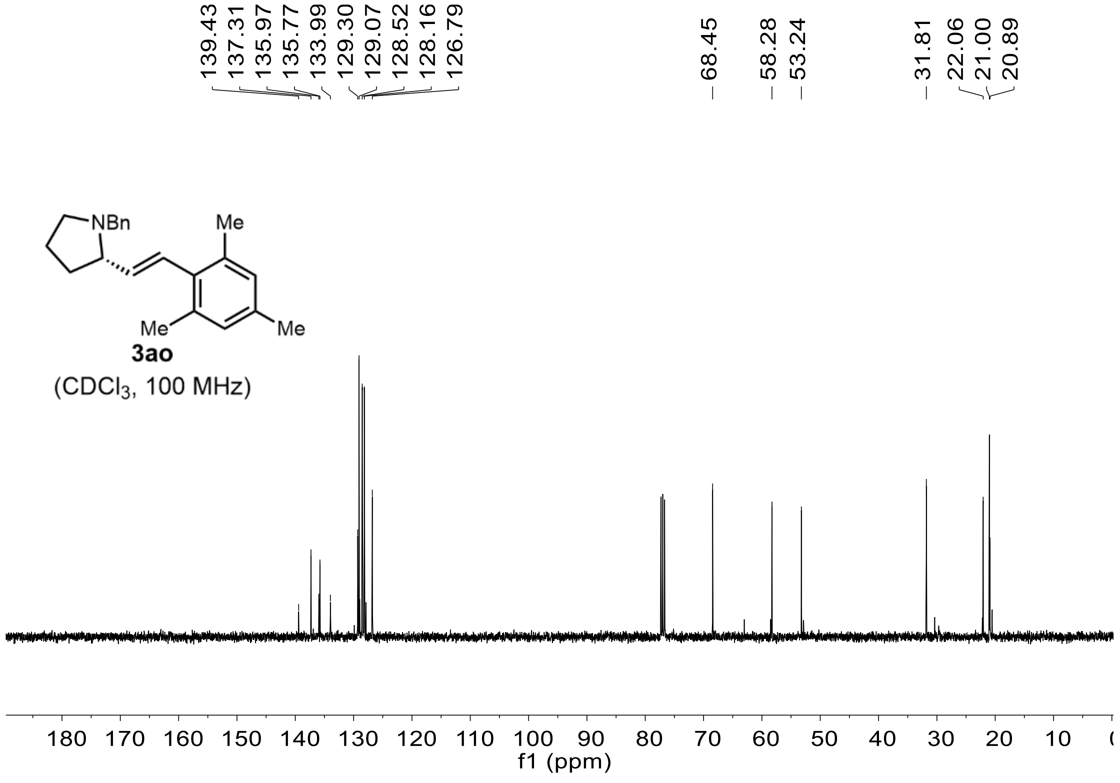
139.43
137.31
135.97
135.77
133.99
129.30
129.07
128.52
128.16
126.79
68.45
58.28
53.24
31.81
22.06
21.00
20.89
NBn
Me
Me
Me
3ao
(CDCl3, 100 MHz)
180
170
160
150
140
130
120
110
100
90
80
70
60
50
40
30
20
10
f1 (ppm)

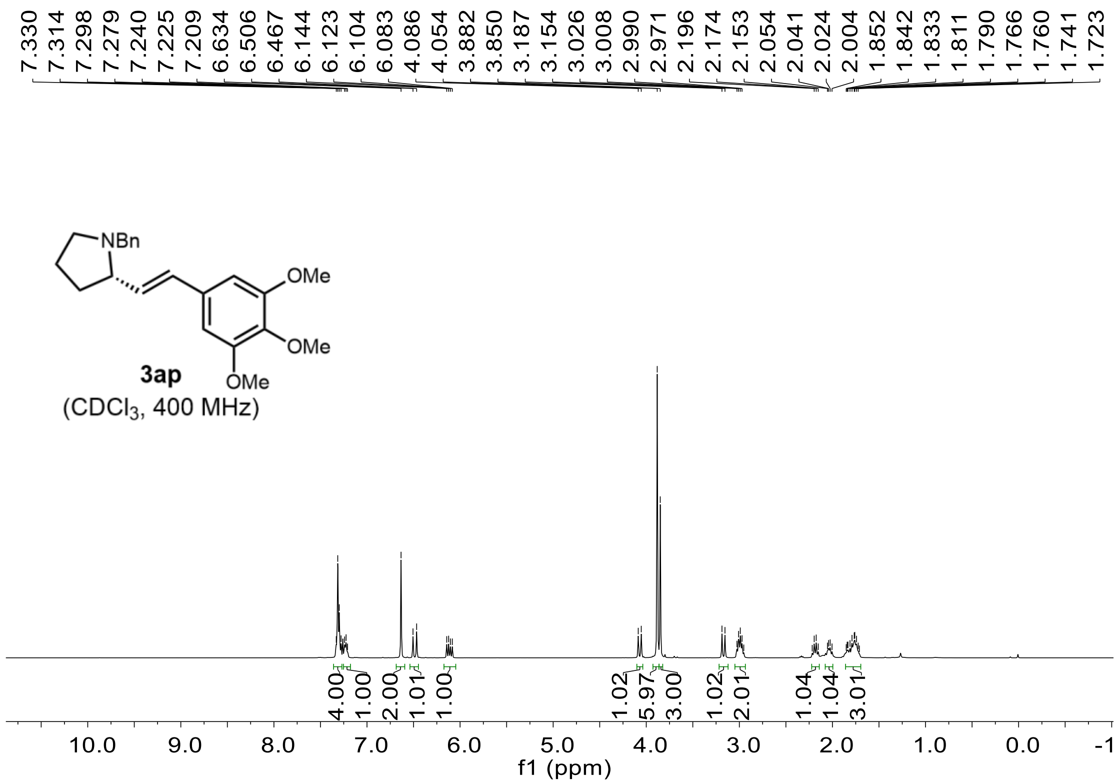
7.330 7.314 7.298 7.279 7.240 7.225 7.209 6.634 6.506 6.467 6.144 6.123 6.104 6.083 4.086 4.054 3.882 3.850 3.187 3.154 3.026 3.008 2.990 2.971 2.196 2.174 2.153 2.054 2.041 2.024 2.004 1.852 1.842 1.833 1.811 1.790 1.766 1.760 1.741 1.723
NBn
OMe
OMe
OMe
3ap
($CDCl_3$, 400 MHz)
4.00 1.00 2.00 1.01 1.00 1.02 5.97 3.00 1.02 2.01 1.04 1.04 3.01
10.0 9.0 8.0 7.0 6.0 5.0 4.0 3.0 2.0 1.0 0.0 -1
f1 (ppm)

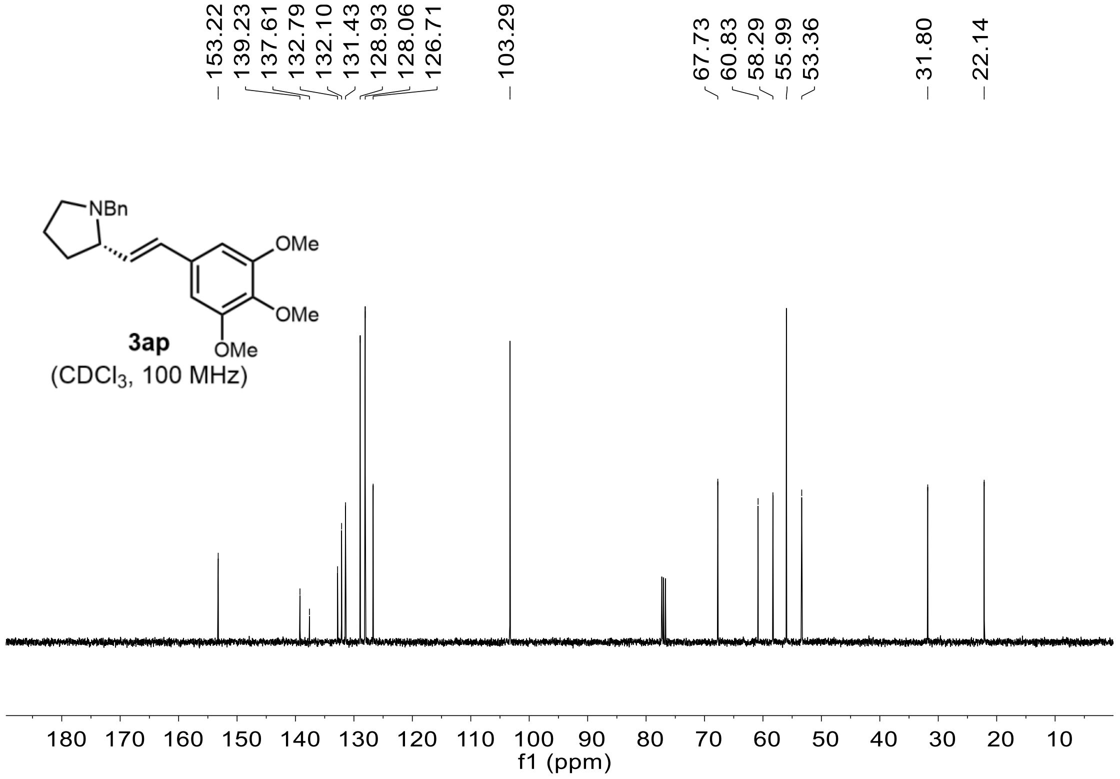
153.22 139.23 137.61 132.79 132.10 131.43 128.93 128.06 126.71 103.29 67.73 60.83 58.29 55.99 53.36 31.80 22.14
NBn
OMe
OMe
OMe
3ap
($CDCl_3$, 100 MHz)
180 170 160 150 140 130 120 110 100 90 80 70 60 50 40 30 20 10
f1 (ppm)

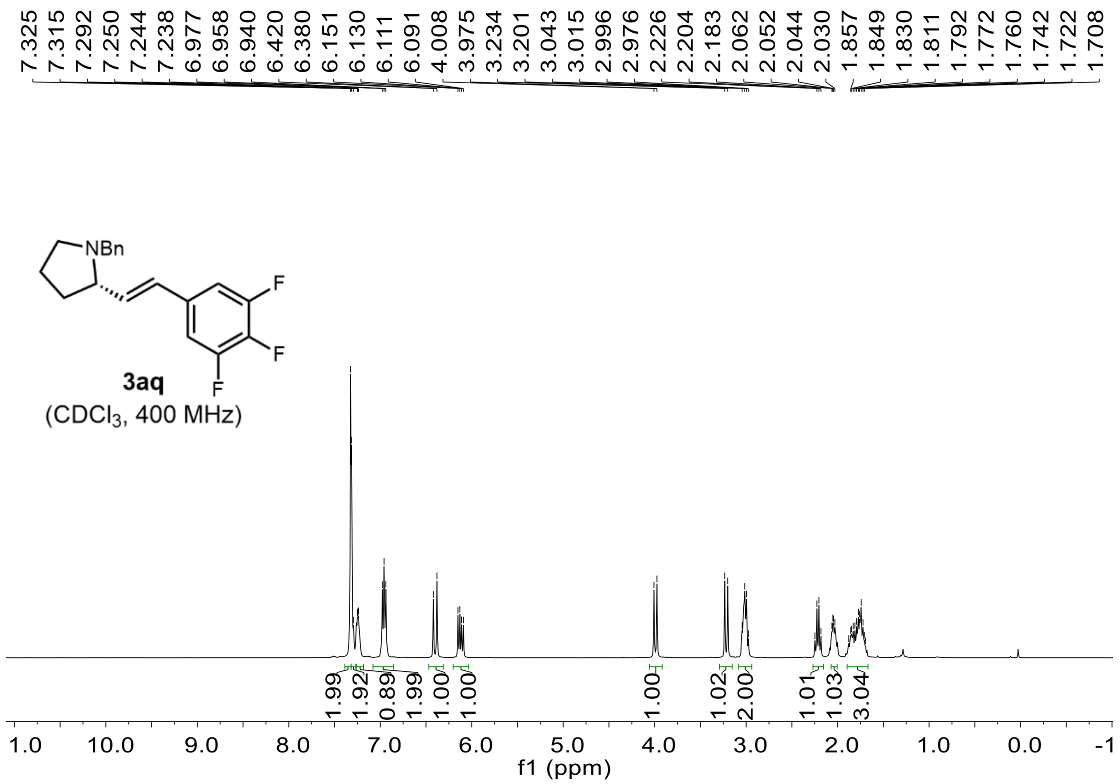

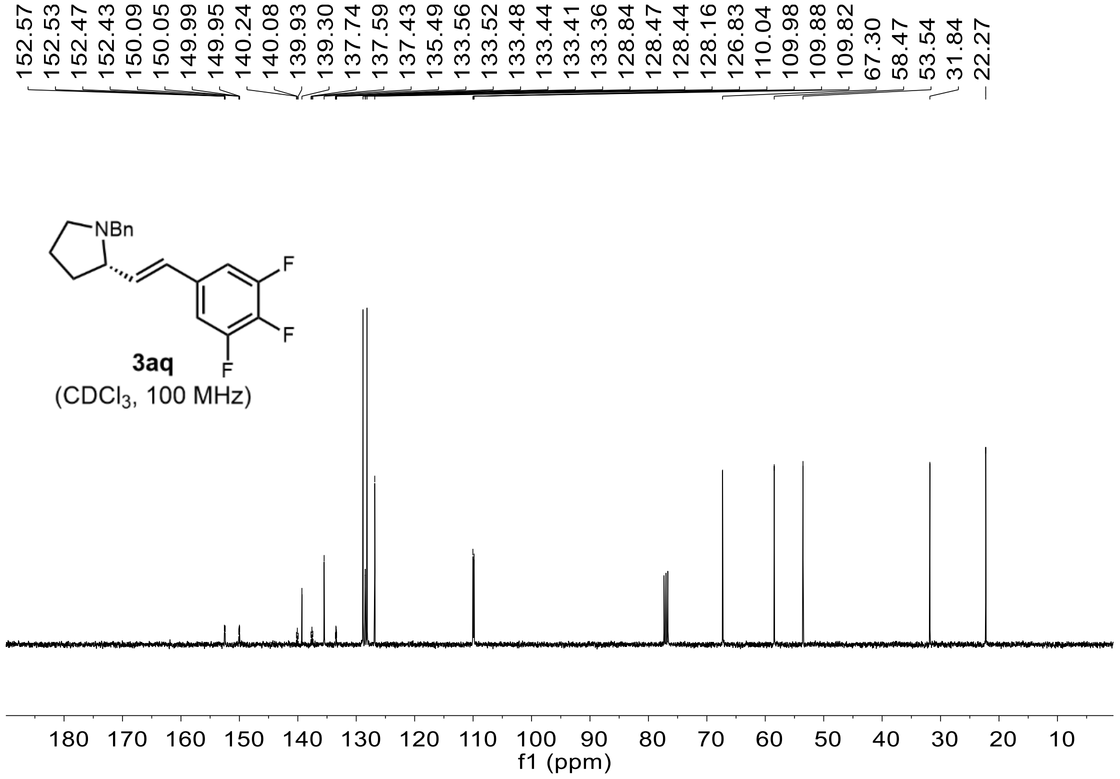

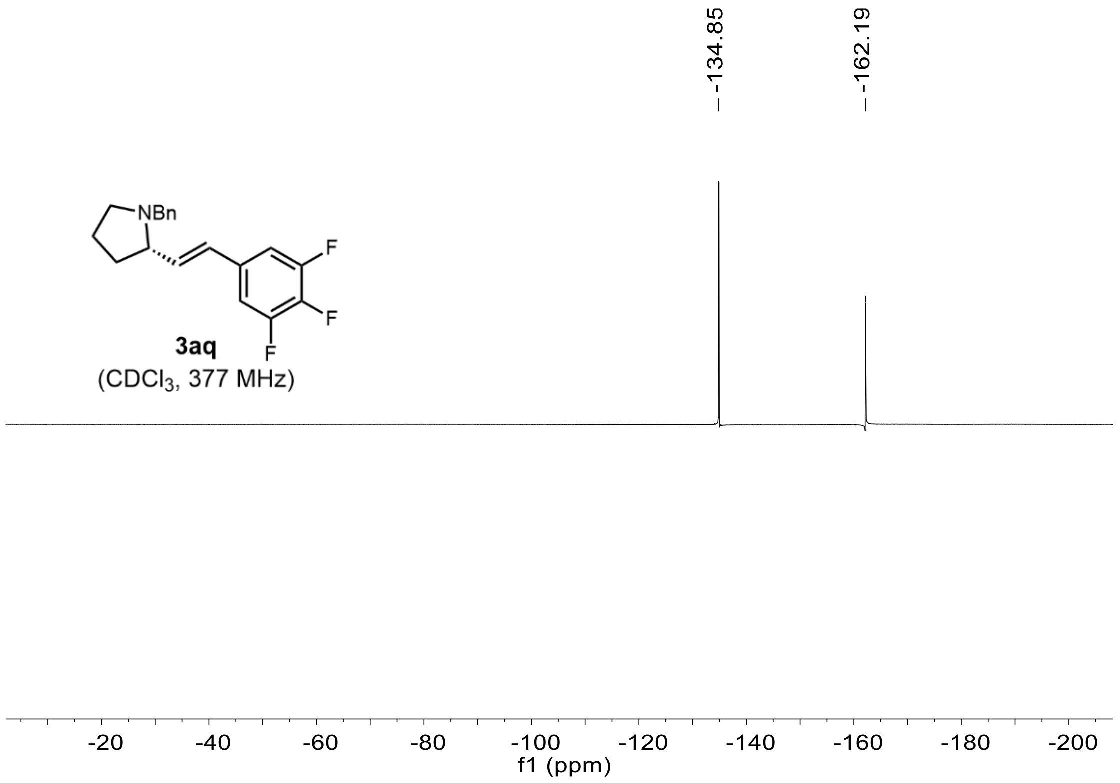
-134.85
-162.19
NBn
F
F
F
3aq
(CDCl$_3$, 377 MHz)
-20 -40 -60 -80 -100 -120 -140 -160 -180 -200
f1 (ppm)

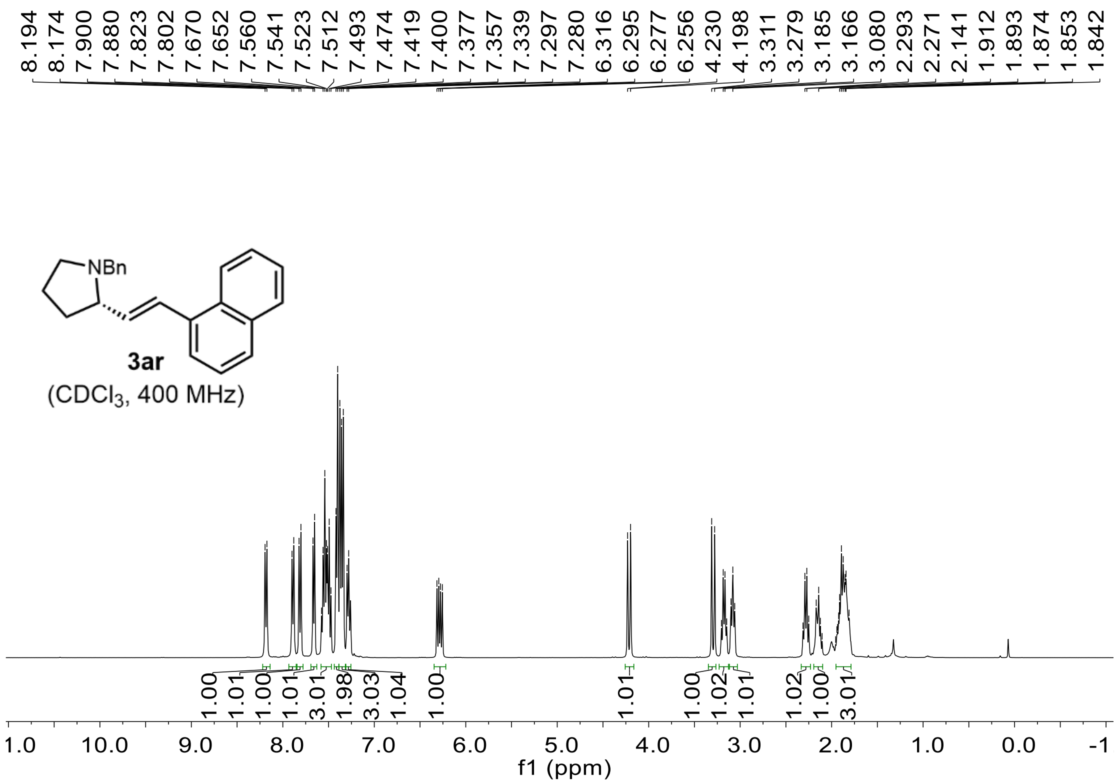
8.194 8.174 7.900 7.880 7.823 7.802 7.670 7.652 7.560 7.541 7.523 7.512 7.493 7.474 7.419 7.400 7.377 7.357 7.339 7.297 7.280 6.316 6.295 6.277 6.256 4.230 4.198 3.311 3.279 3.185 3.166 3.080 2.293 2.271 2.141 1.912 1.893 1.874 1.853 1.842
NBn
3ar
(CDCl$_3$, 400 MHz)
1.00 1.01 1.00 1.01 3.01 1.98 3.03 1.04 1.00 1.01 1.00 1.02 1.01 1.02 1.00 3.01
1.0 10.0 9.0 8.0 7.0 6.0 5.0 4.0 3.0 2.0 1.0 0.0 -1
f1 (ppm)

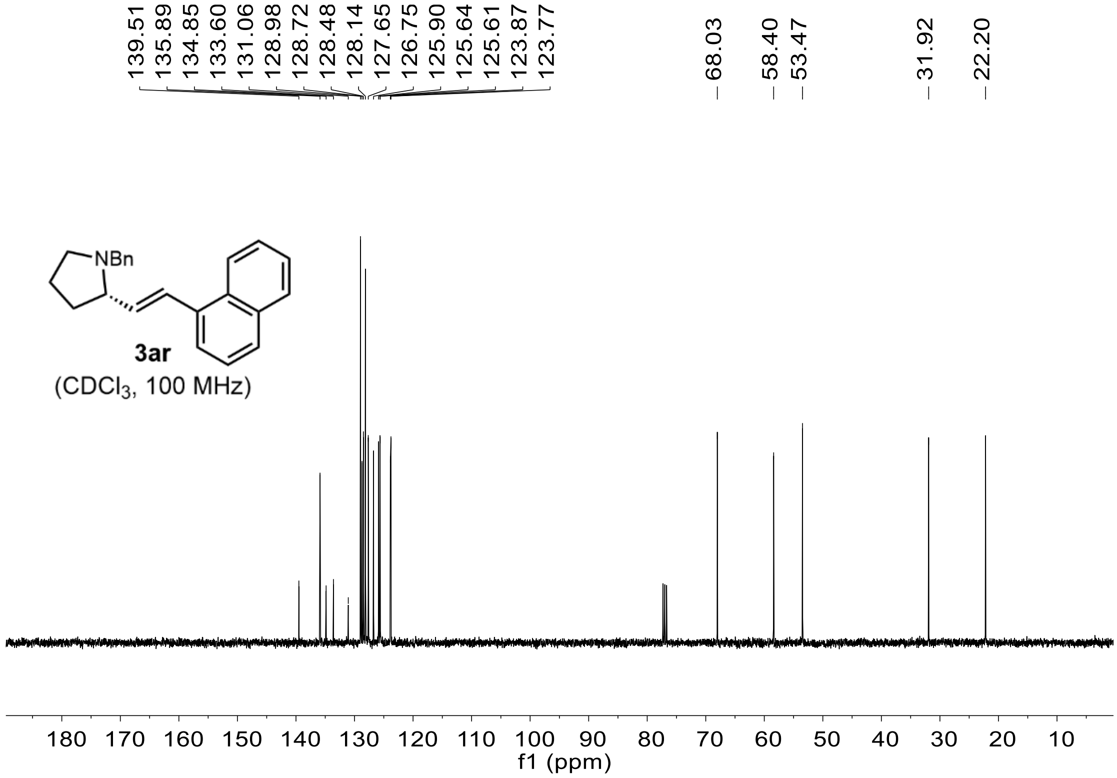
139.51 135.89 134.85 133.60 131.06 128.98 128.72 128.48 128.14 127.65 126.75 125.90 125.64 125.61 123.87 123.77
68.03 58.40 53.47 31.92 22.20
NBn
3ar
(CDCl$_3$, 100 MHz)
180 170 160 150 140 130 120 110 100 90 80 70 60 50 40 30 20 10
f1 (ppm)

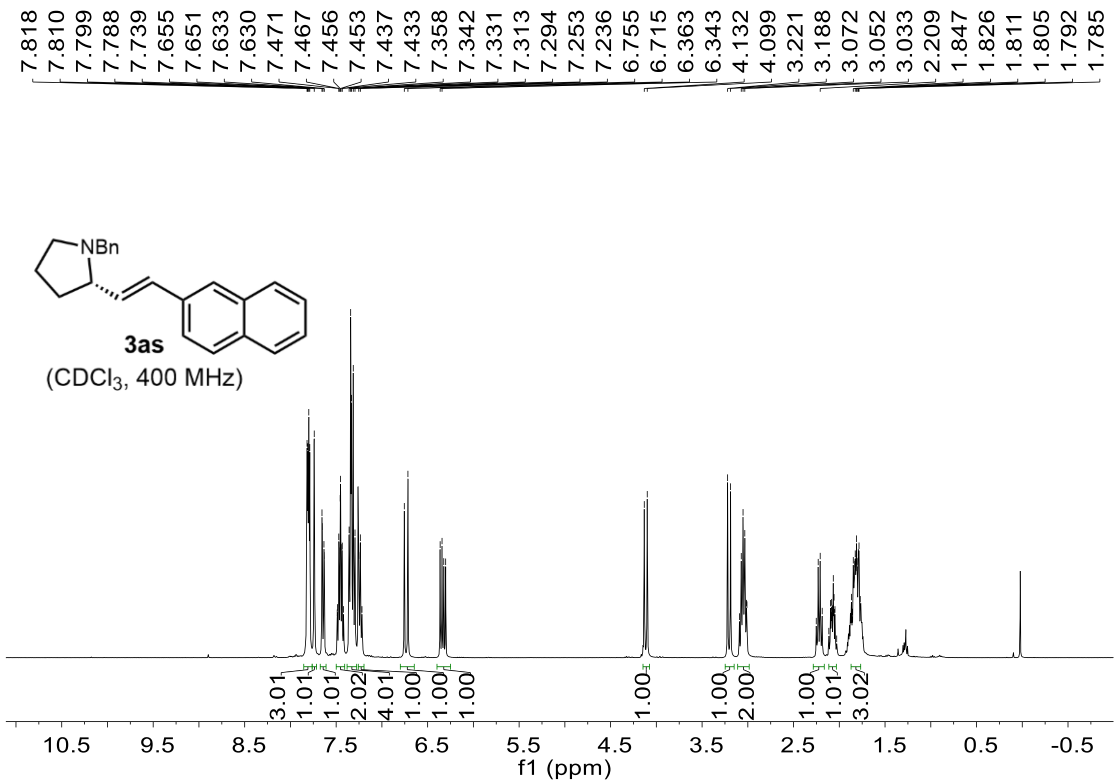
7.818 7.810 7.799 7.788 7.739 7.655 7.651 7.633 7.630 7.471 7.467 7.456 7.453 7.437 7.433 7.358 7.342 7.331 7.313 7.294 7.253 7.236 6.755 6.715 6.363 6.343 4.132 4.099 3.221 3.188 3.072 3.052 3.033 2.209 1.847 1.826 1.811 1.805 1.792 1.785
NBn
3as
(CDCl$_3$, 400 MHz)
3.01 1.01 1.01 2.02 4.01 1.00 1.00 1.00 1.00 1.00 2.00 1.00 1.01 3.02
10.5 9.5 8.5 7.5 6.5 5.5 4.5 3.5 2.5 1.5 0.5 -0.5
f1 (ppm)

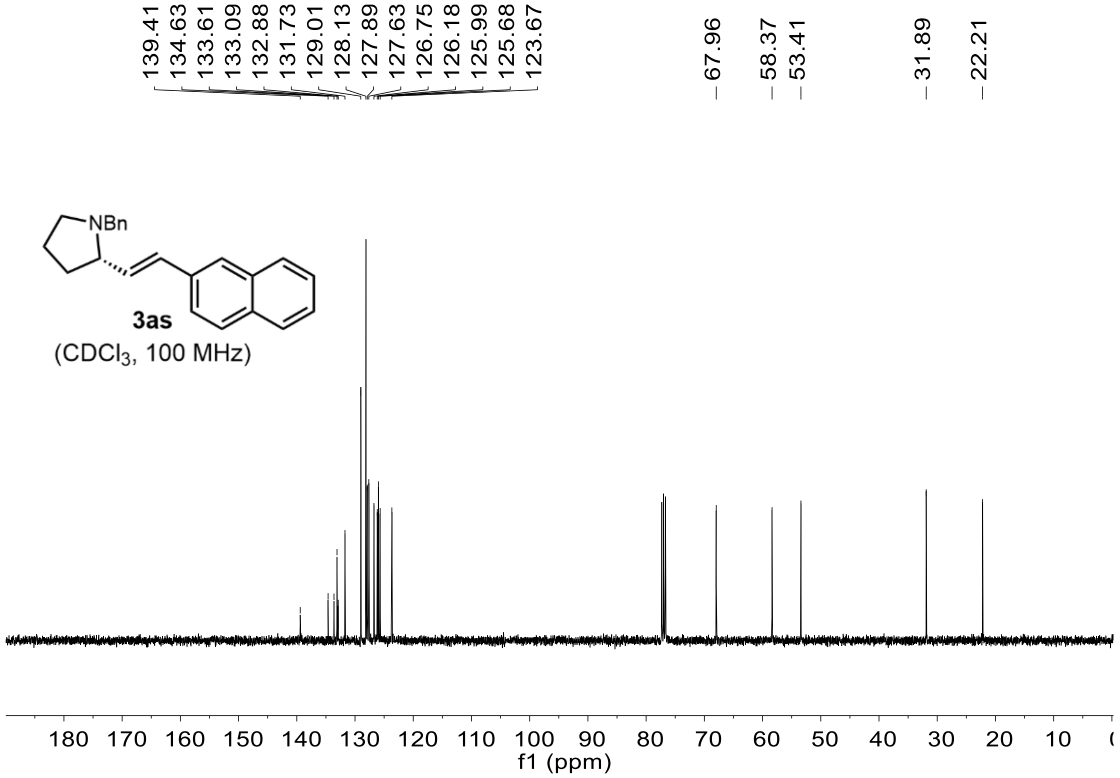
139.41 134.63 133.61 133.09 132.88 131.73 129.01 128.13 127.89 127.63 126.75 126.18 125.99 125.68 123.67
67.96 58.37 53.41 31.89 22.21
NBn
3as
(CDCl$_3$, 100 MHz)
180 170 160 150 140 130 120 110 100 90 80 70 60 50 40 30 20 10
f1 (ppm)

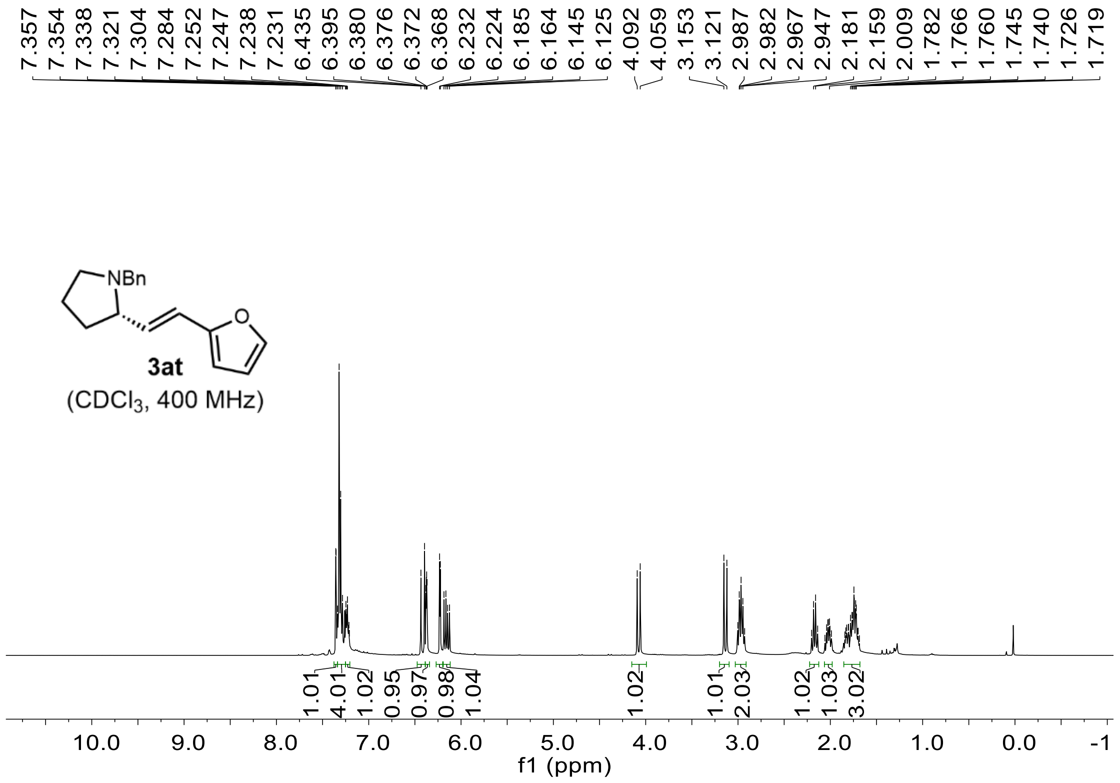

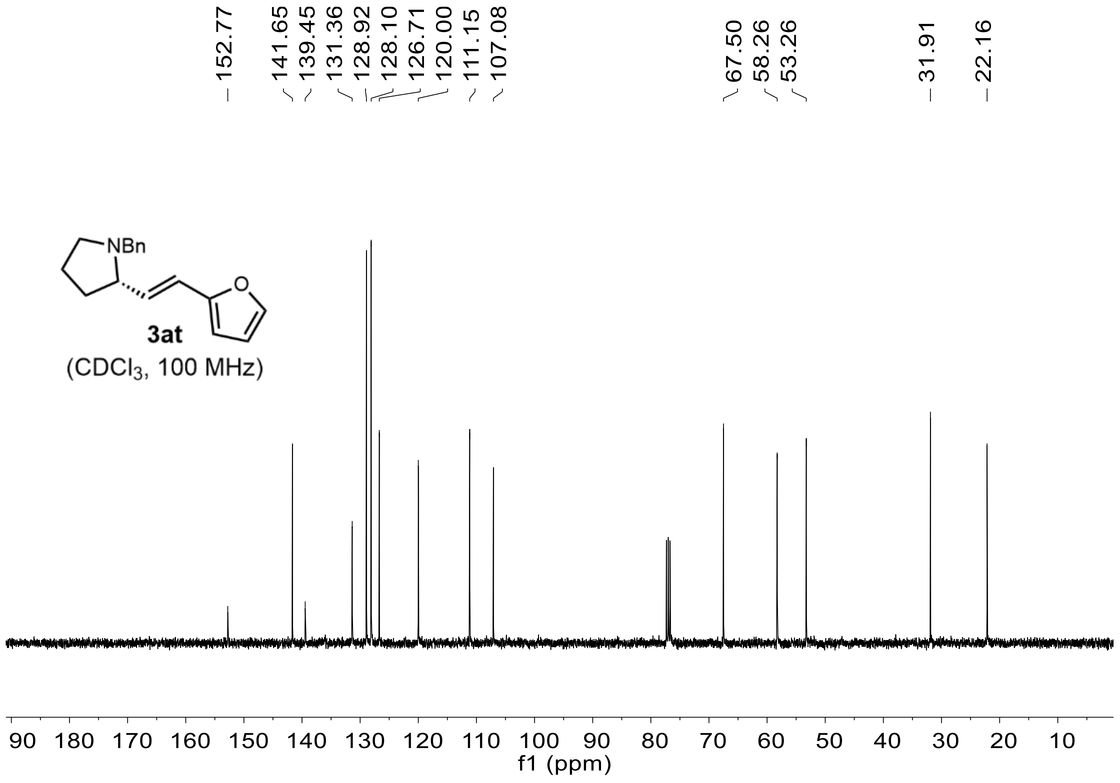

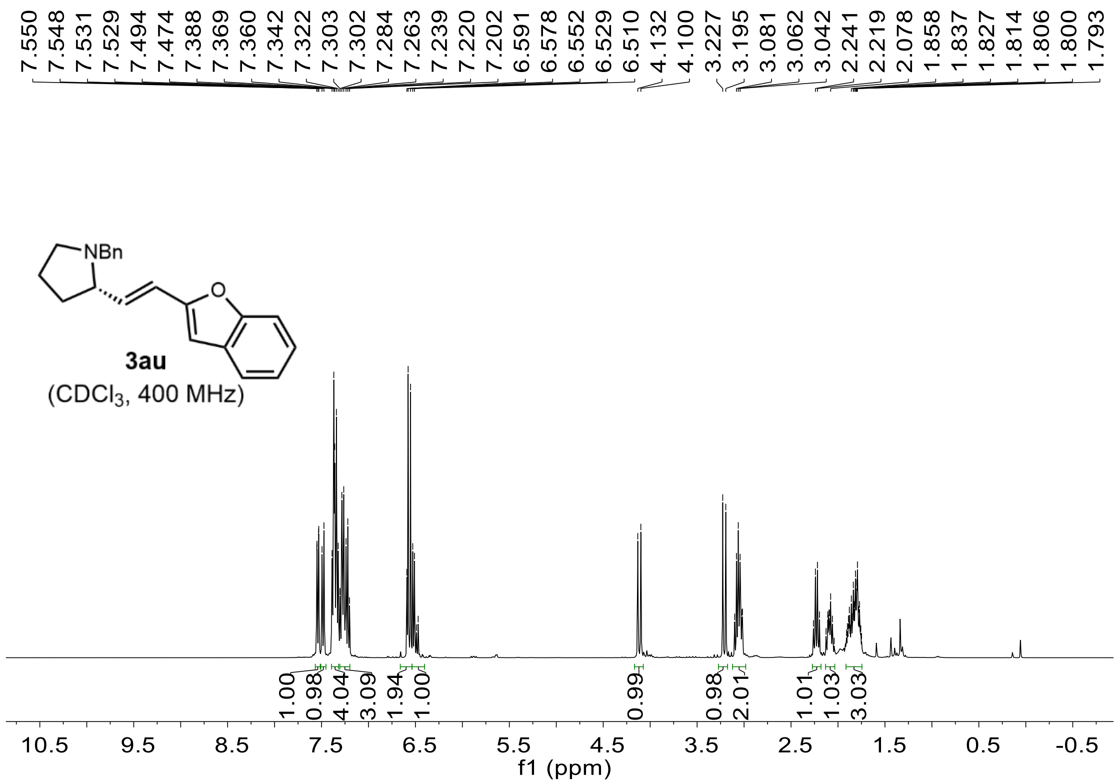
7.550
7.548
7.531
7.529
7.494
7.474
7.388
7.369
7.360
7.342
7.322
7.303
7.302
7.284
7.263
7.239
7.220
7.202
6.591
6.578
6.552
6.529
6.510
4.132
4.100
3.227
3.195
3.081
3.062
3.042
2.241
2.219
2.078
1.858
1.837
1.827
1.814
1.806
1.800
1.793
NBn
O
3au
(CDCl3, 400 MHz)
1.00
0.98
4.04
3.09
1.94
1.00
0.99
0.98
2.01
1.01
1.03
3.03
10.5
9.5
8.5
7.5
6.5
5.5
4.5
3.5
2.5
1.5
0.5
-0.5
f1 (ppm)

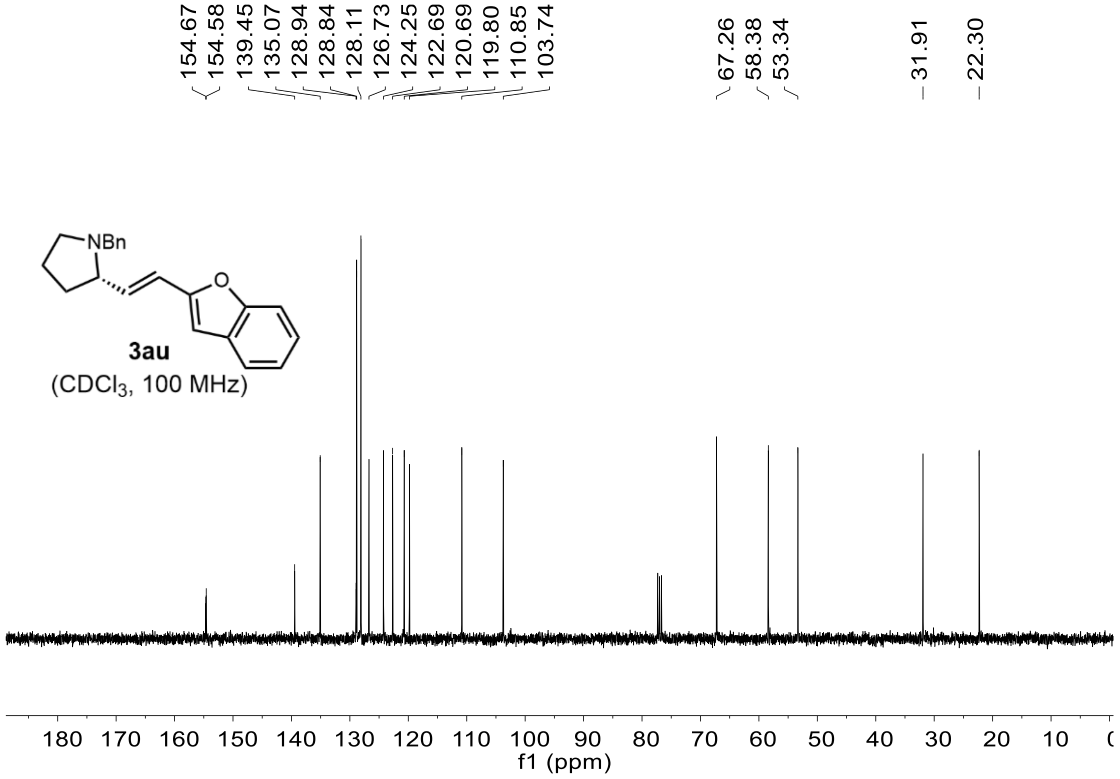
154.67
154.58
139.45
135.07
128.94
128.84
128.11
126.73
124.25
122.69
120.69
119.80
110.85
103.74
67.26
58.38
53.34
31.91
22.30
NBn
O
3au
(CDCl3, 100 MHz)
180
170
160
150
140
130
120
110
100
90
80
70
60
50
40
30
20
10
f1 (ppm)

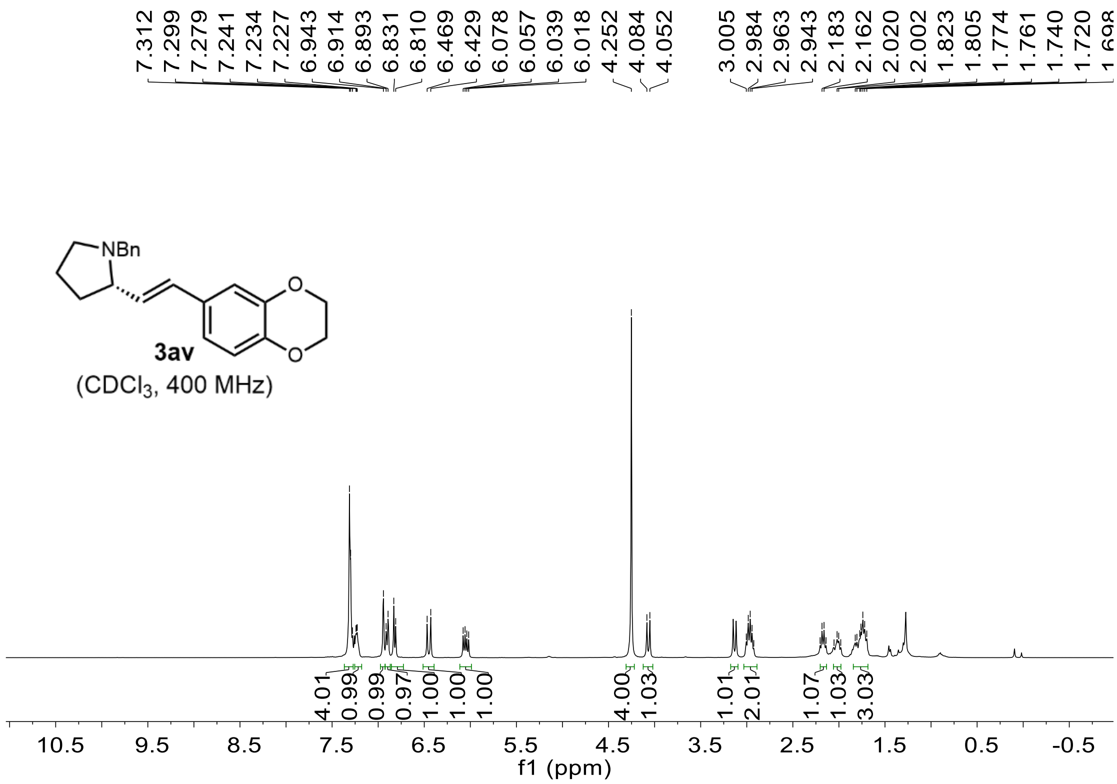

7.312
7.299
7.279
7.241
7.234
7.227
6.943
6.914
6.893
6.831
6.810
6.469
6.429
6.078
6.057
6.039
6.018
4.252
4.084
4.052
3.005
2.984
2.963
2.943
2.183
2.162
2.020
2.002
1.823
1.805
1.774
1.761
1.740
1.720
NBn
O
O
3av
(CDCl$_3$, 400 MHz)
4.01
0.99
0.99
0.97
1.00
1.00
1.00
4.00
1.03
1.01
2.01
1.07
1.03
3.03
10.5
9.5
8.5
7.5
6.5
5.5
4.5
3.5
2.5
1.5
0.5
-0.5
f1 (ppm)

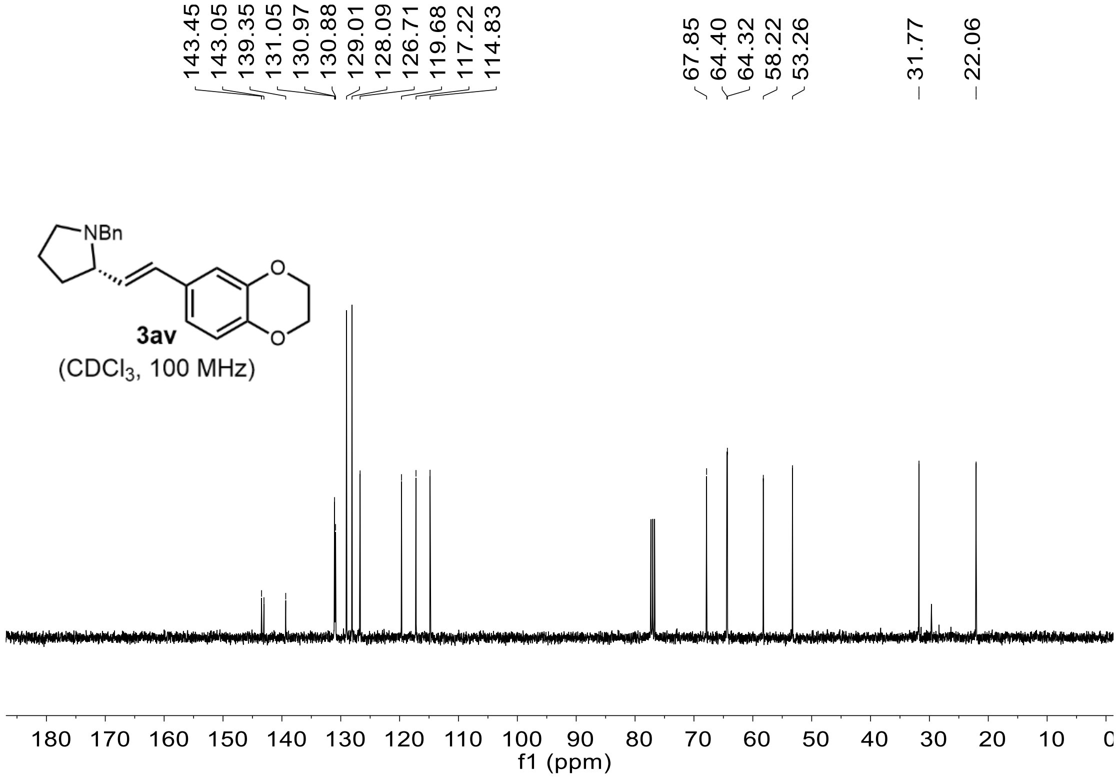

143.45
143.05
139.35
131.05
130.97
130.88
129.01
128.09
126.71
119.68
117.22
114.83
67.85
64.40
64.32
58.22
53.26
31.77
22.06
NBn
O
O
3av
(CDCl$_3$, 100 MHz)
180
170
160
150
140
130
120
110
100
90
80
70
60
50
40
30
20
10
f1 (ppm)

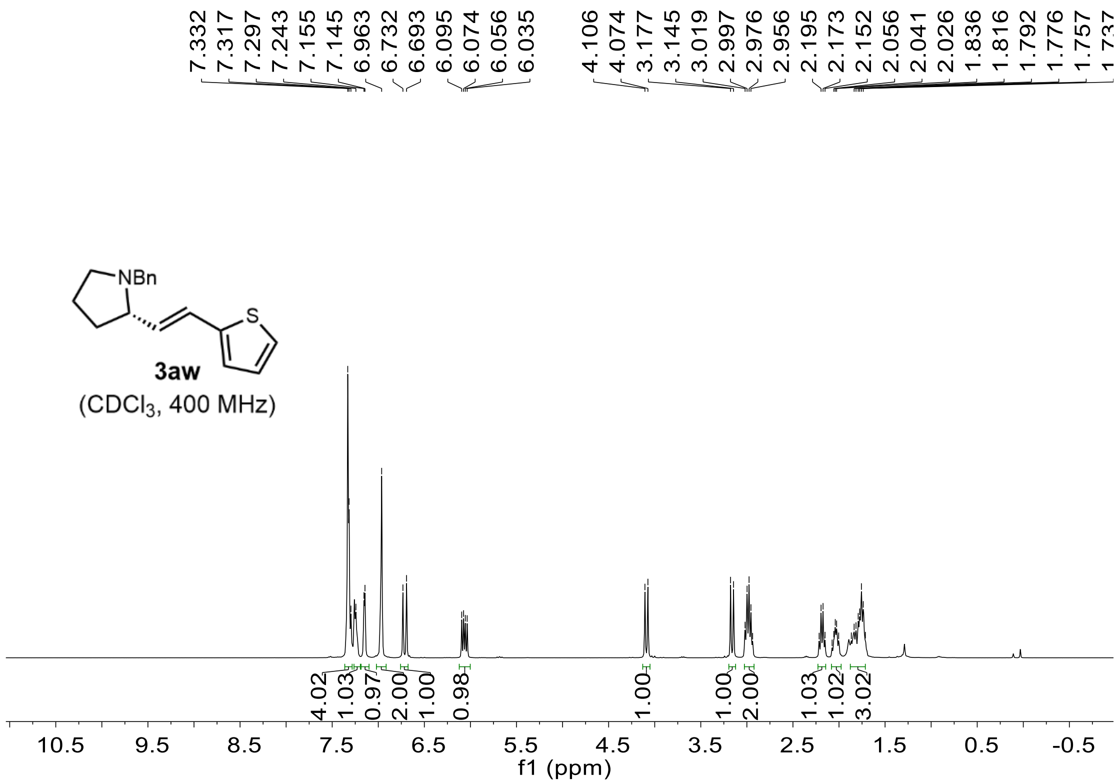
7.332
7.317
7.297
7.243
7.155
7.145
6.963
6.732
6.693
6.095
6.074
6.056
6.035
4.106
4.074
3.177
3.145
3.019
2.997
2.976
2.956
2.195
2.173
2.152
2.056
2.041
2.026
1.836
1.816
1.792
1.776
1.757
NBn
S
3aw
(CDCl3, 400 MHz)
4.02
1.03
0.97
2.00
1.00
0.98
1.00
1.00
2.00
1.03
1.02
3.02
f1 (ppm)

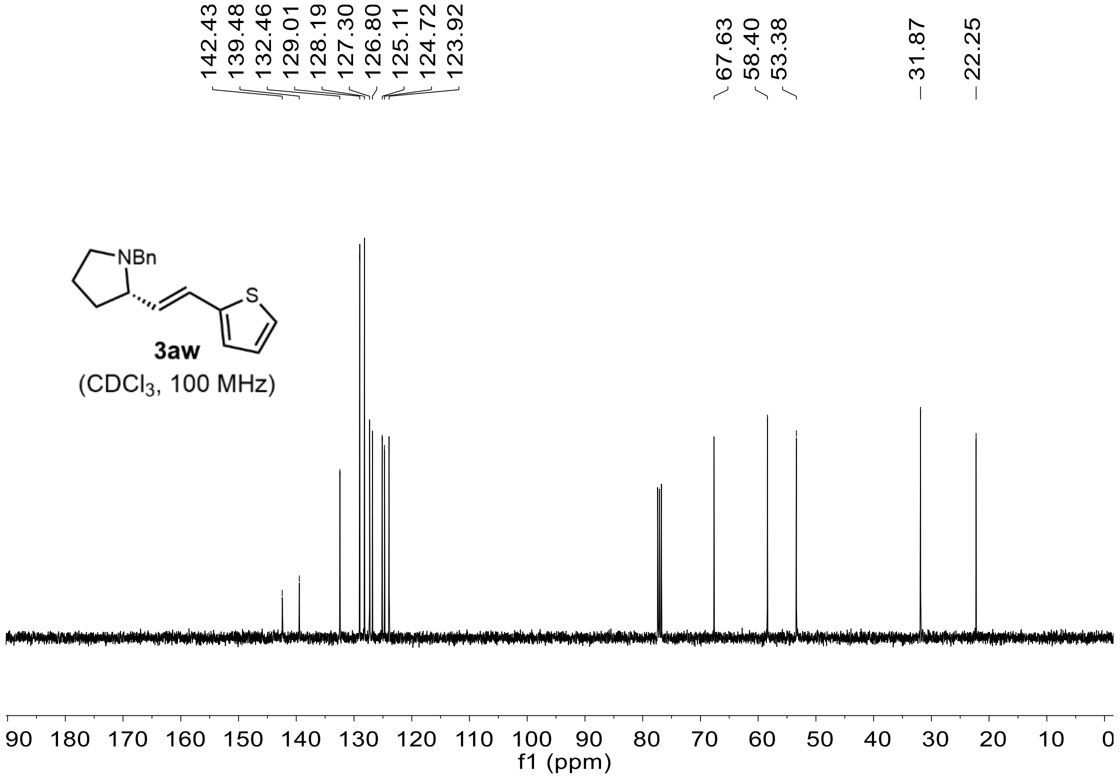
142.43
139.48
132.46
129.01
128.19
127.30
126.80
125.11
124.72
123.92
67.63
58.40
53.38
31.87
22.25
NBn
S
3aw
(CDCl3, 100 MHz)
f1 (ppm)

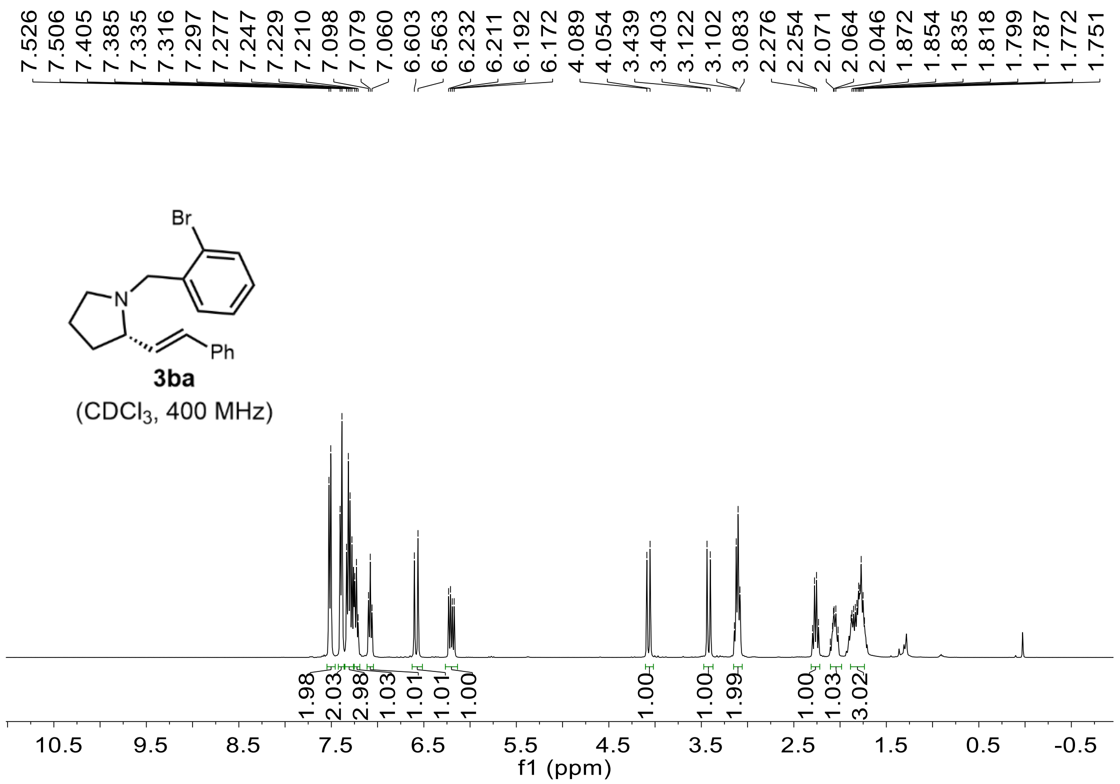
7.526 7.506 7.405 7.385 7.335 7.316 7.297 7.277 7.247 7.229 7.210 7.098 7.079 7.060 6.603 6.563 6.232 6.211 6.192 6.172 4.089 4.054 3.439 3.403 3.122 3.102 3.083 2.276 2.254 2.071 2.064 2.046 1.872 1.854 1.835 1.818 1.799 1.787 1.772 1.751
Br
N
Ph
3ba
(CDCl$_3$, 400 MHz)
1.98 2.03 2.98 1.03 1.01 1.01 1.00 1.00 1.00 1.99 1.00 1.03 3.02
10.5 9.5 8.5 7.5 6.5 5.5 4.5 3.5 2.5 1.5 0.5 -0.5
f1 (ppm)

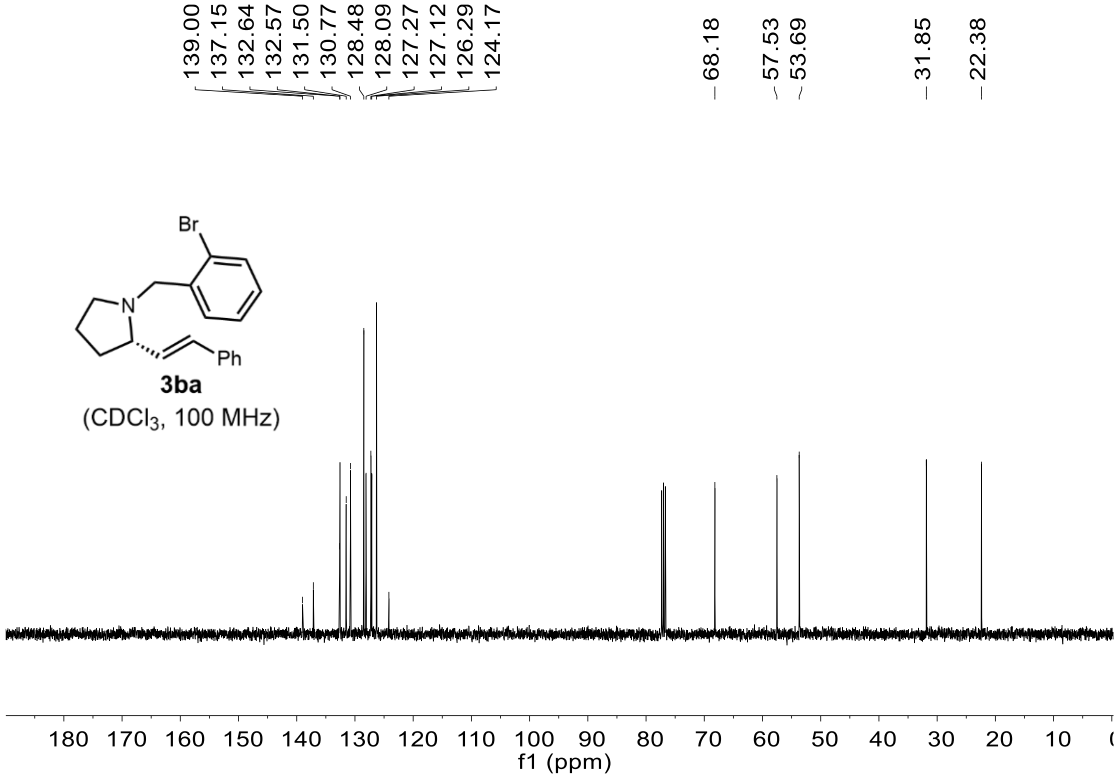
139.00 137.15 132.64 132.57 131.50 130.77 128.48 128.09 127.27 127.12 126.29 124.17 68.18 57.53 53.69 31.85 22.38
Br
N
Ph
3ba
(CDCl$_3$, 100 MHz)
180 170 160 150 140 130 120 110 100 90 80 70 60 50 40 30 20 10
f1 (ppm)

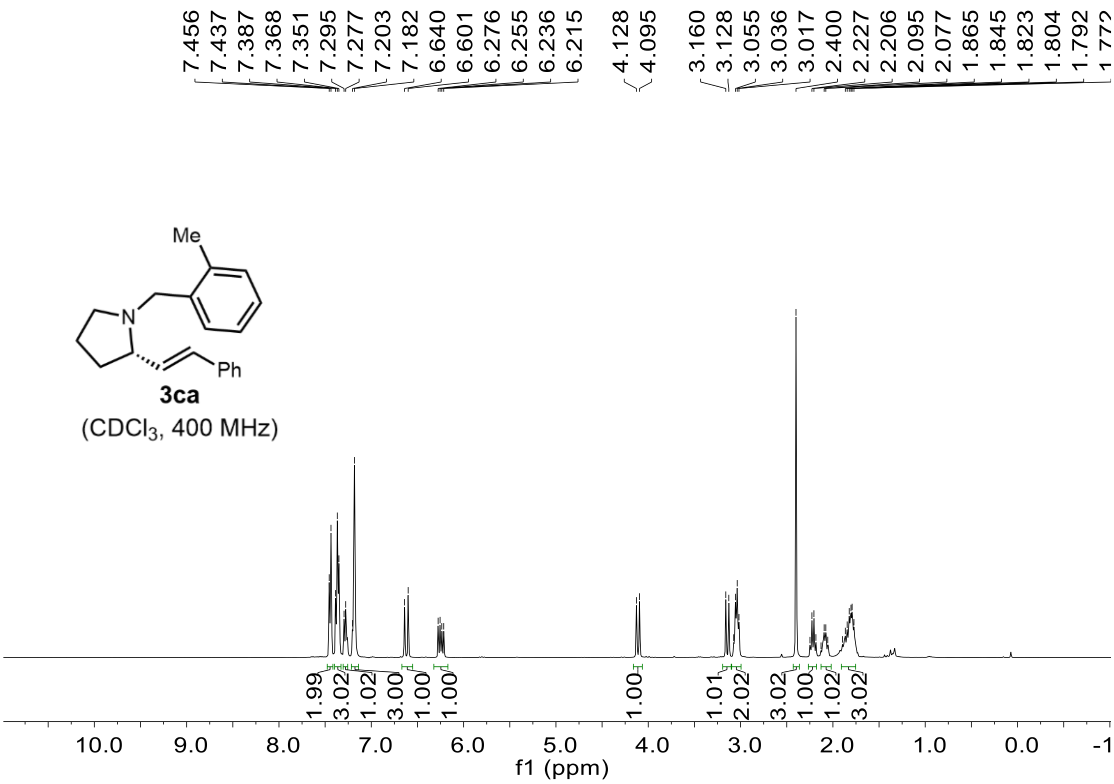
7.456
7.437
7.387
7.368
7.351
7.295
7.277
7.203
7.182
6.640
6.601
6.276
6.255
6.236
6.215
4.128
4.095
3.160
3.128
3.055
3.036
3.017
2.400
2.227
2.206
2.095
2.077
1.865
1.845
1.823
1.804
1.792
Me
N
Ph
3ca
(CDCl3, 400 MHz)
1.99
3.02
1.02
3.00
1.00
1.00
1.00
1.01
2.02
3.02
1.00
1.02
3.02
10.0
9.0
8.0
7.0
6.0
5.0
4.0
3.0
2.0
1.0
0.0
f1 (ppm)

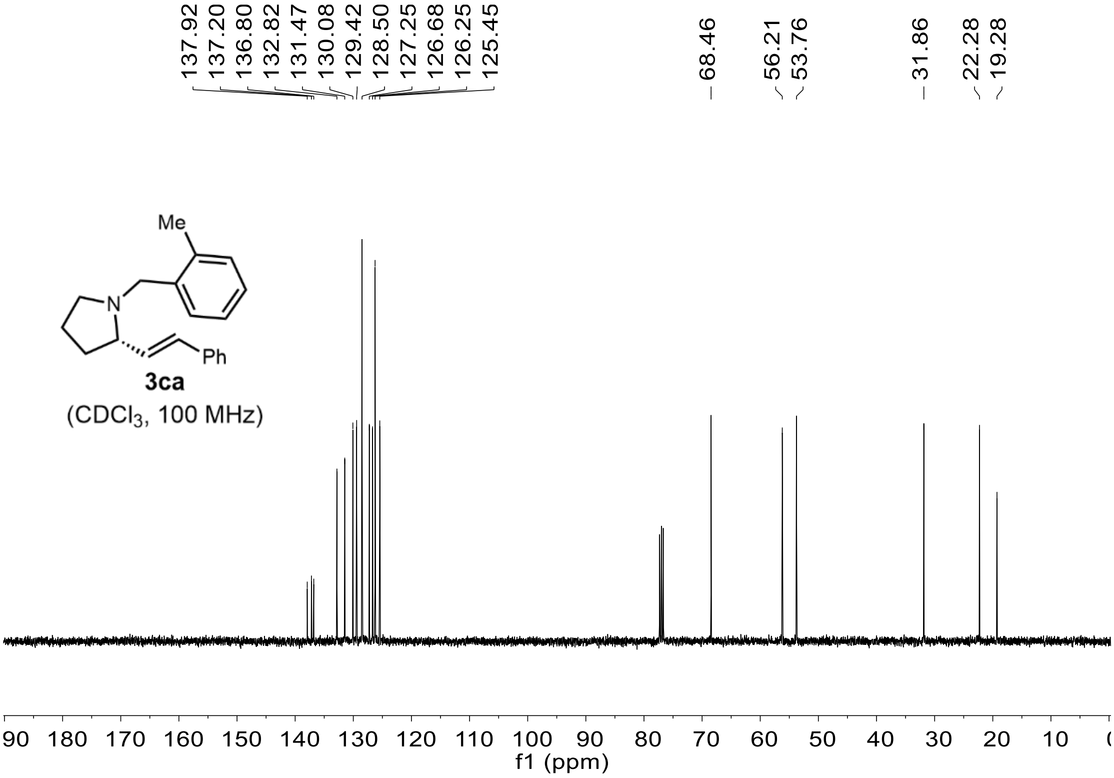
137.92
137.20
136.80
132.82
131.47
130.08
129.42
128.50
127.25
126.68
126.25
125.45
68.46
56.21
53.76
31.86
22.28
19.28
Me
N
Ph
3ca
(CDCl3, 100 MHz)
90
180
170
160
150
140
130
120
110
100
90
80
70
60
50
40
30
20
10
f1 (ppm)

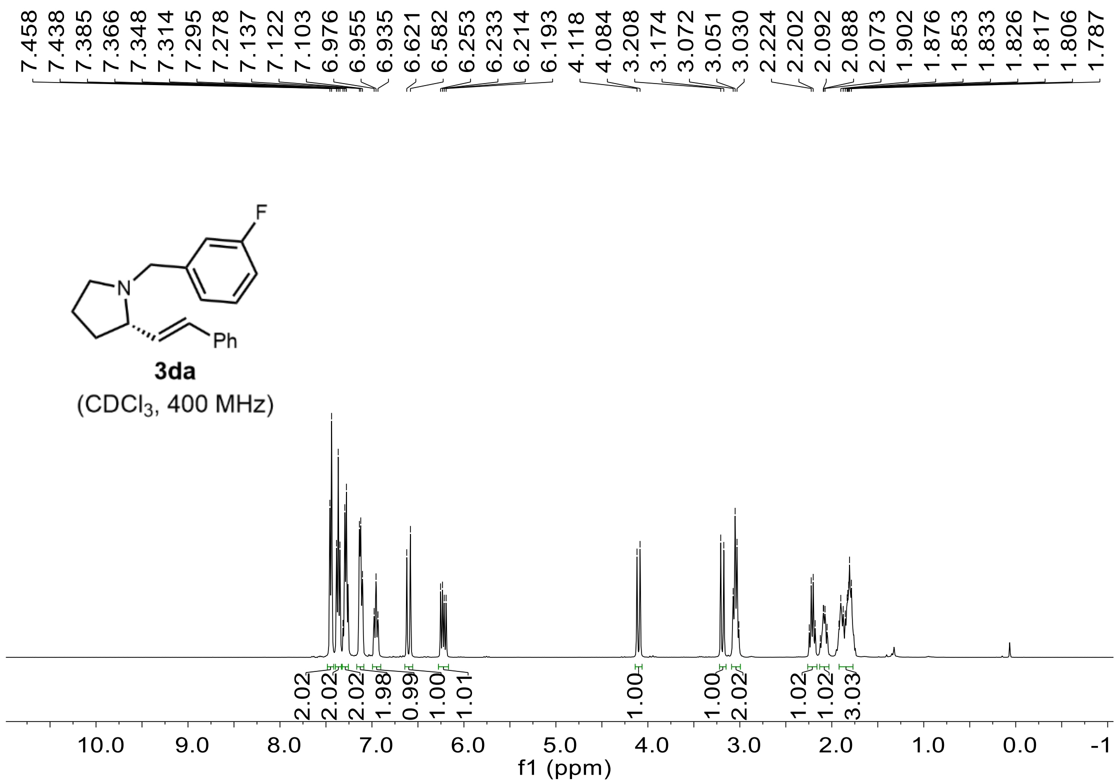
7.458 7.438 7.385 7.366 7.348 7.314 7.295 7.278 7.137 7.122 7.103 6.976 6.955 6.935 6.621 6.582 6.253 6.233 6.214 6.193 4.118 4.084 3.208 3.174 3.072 3.051 3.030 2.224 2.202 2.092 2.088 2.073 1.902 1.876 1.853 1.833 1.826 1.817 1.806 1.787
F
N
Ph
3da
(CDCl3, 400 MHz)
2.02 2.02 2.02 1.98 0.99 1.00 1.01
1.00
1.00 2.02
1.02 1.02 3.03
10.0 9.0 8.0 7.0 6.0 5.0 4.0 3.0 2.0 1.0 0.0 -1
f1 (ppm)

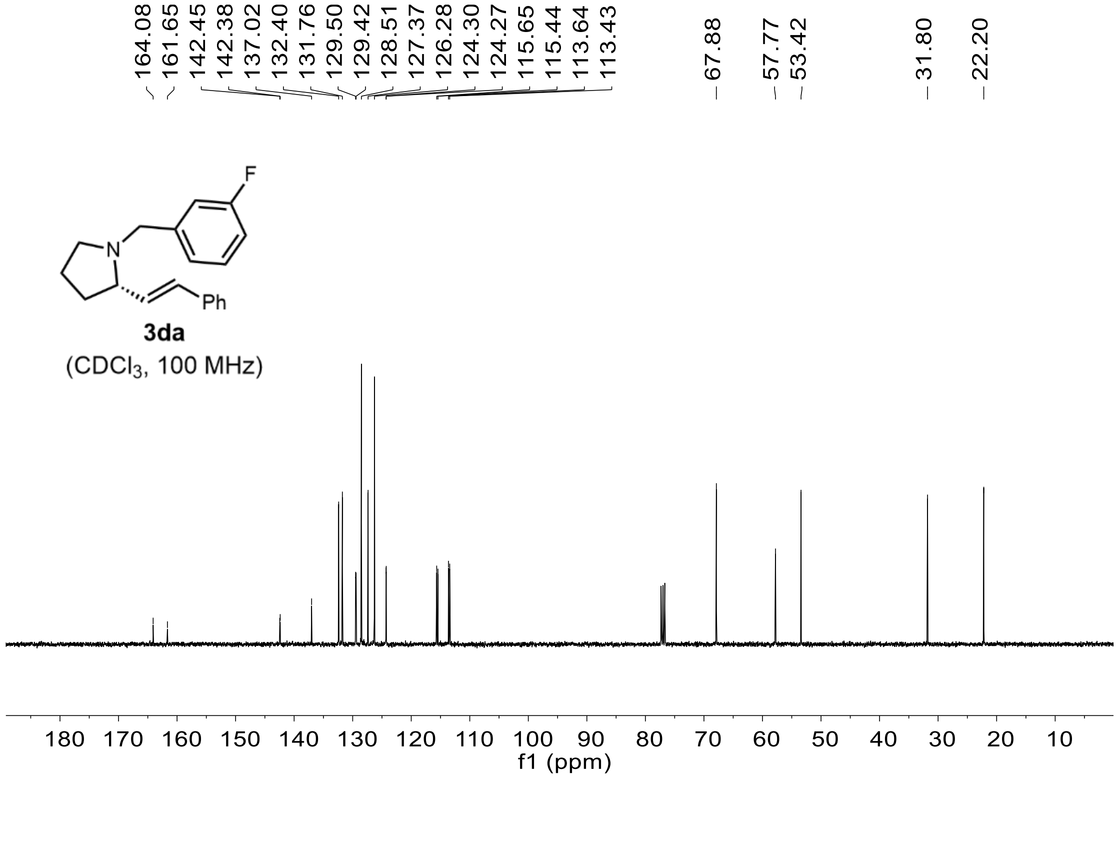
164.08 161.65 142.45 142.38 137.02 132.40 131.76 129.50 129.42 128.51 127.37 126.28 124.30 124.27 115.65 115.44 113.64 113.43
67.88
57.77 53.42
31.80
22.20
F
N
Ph
3da
(CDCl3, 100 MHz)
180 170 160 150 140 130 120 110 100 90 80 70 60 50 40 30 20 10
f1 (ppm)

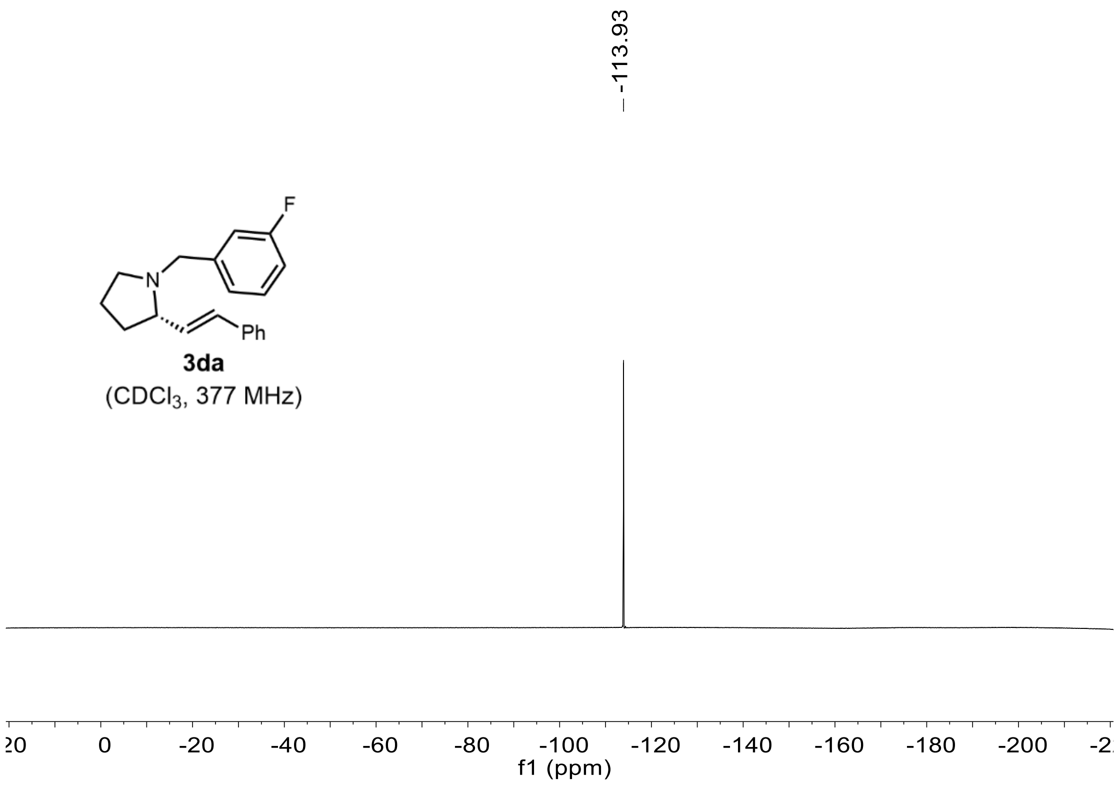
-113.93
F
N
Ph
3da
(CDCl3, 377 MHz)
20 0 -20 -40 -60 -80 -100 -120 -140 -160 -180 -200 -2
f1 (ppm)

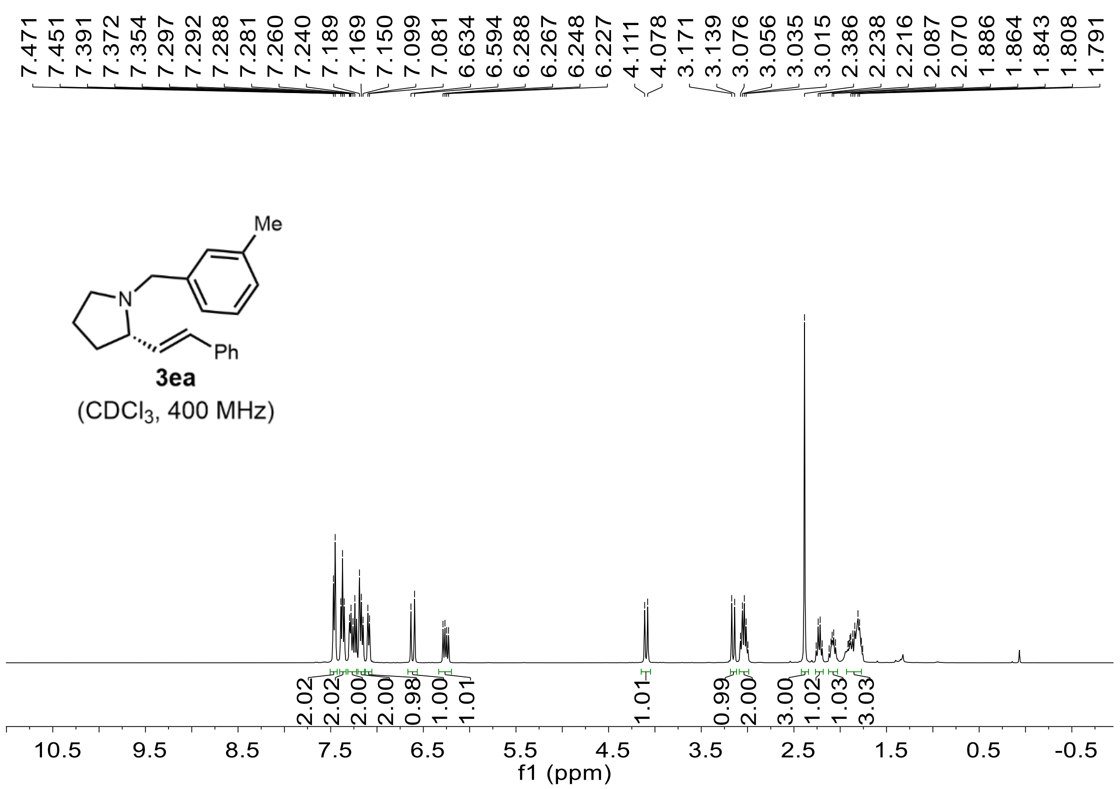
7.471 7.451 7.391 7.372 7.354 7.297 7.292 7.288 7.281 7.260 7.240 7.189 7.169 7.150 7.099 7.081 6.634 6.594 6.288 6.267 6.248 6.227 4.111 4.078 3.171 3.139 3.076 3.056 3.035 3.015 2.386 2.238 2.216 2.087 2.070 1.886 1.864 1.843 1.808 1.791
Me
N
Ph
3ea
(CDCl3, 400 MHz)
2.02 2.02 2.00 2.00 0.98 1.00 1.01 1.01 0.99 2.00 3.00 1.02 1.03 3.03
10.5 9.5 8.5 7.5 6.5 5.5 4.5 3.5 2.5 1.5 0.5 -0.5
f1 (ppm)

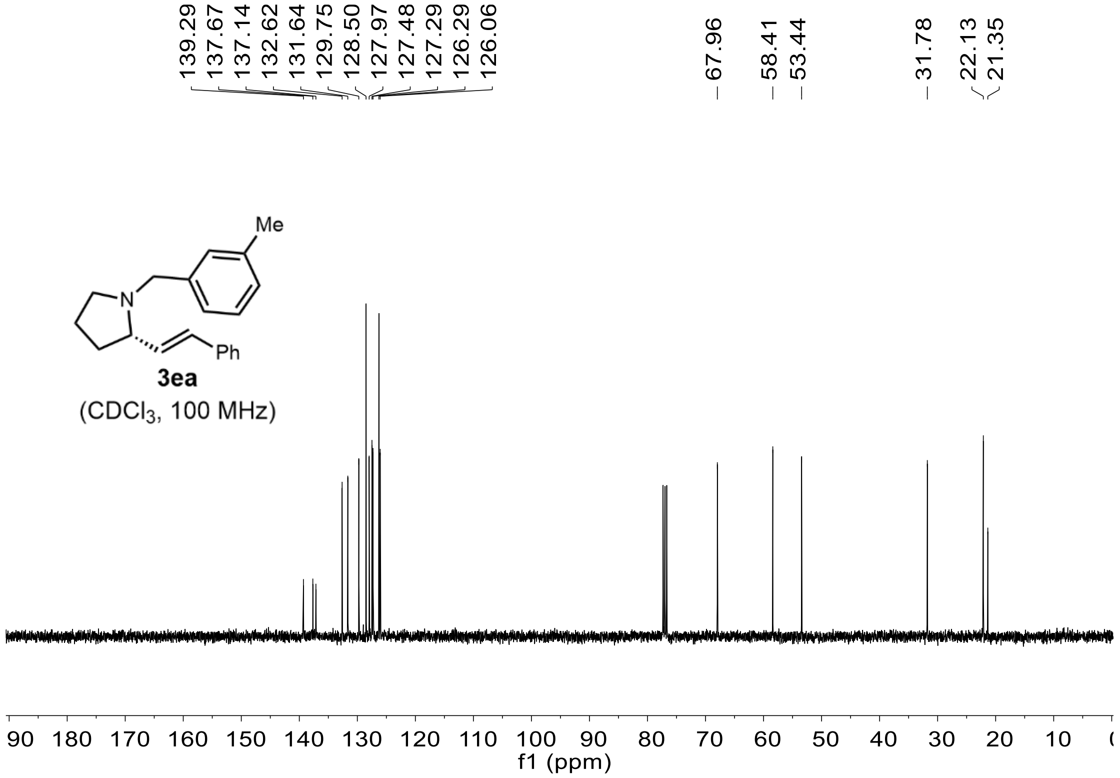
139.29 137.67 137.14 132.62 131.64 129.75 128.50 127.97 127.48 127.29 126.29 126.06 67.96 58.41 53.44 31.78 22.13 21.35
Me
N
Ph
3ea
(CDCl3, 100 MHz)
90 180 170 160 150 140 130 120 110 100 90 80 70 60 50 40 30 20 10
f1 (ppm)

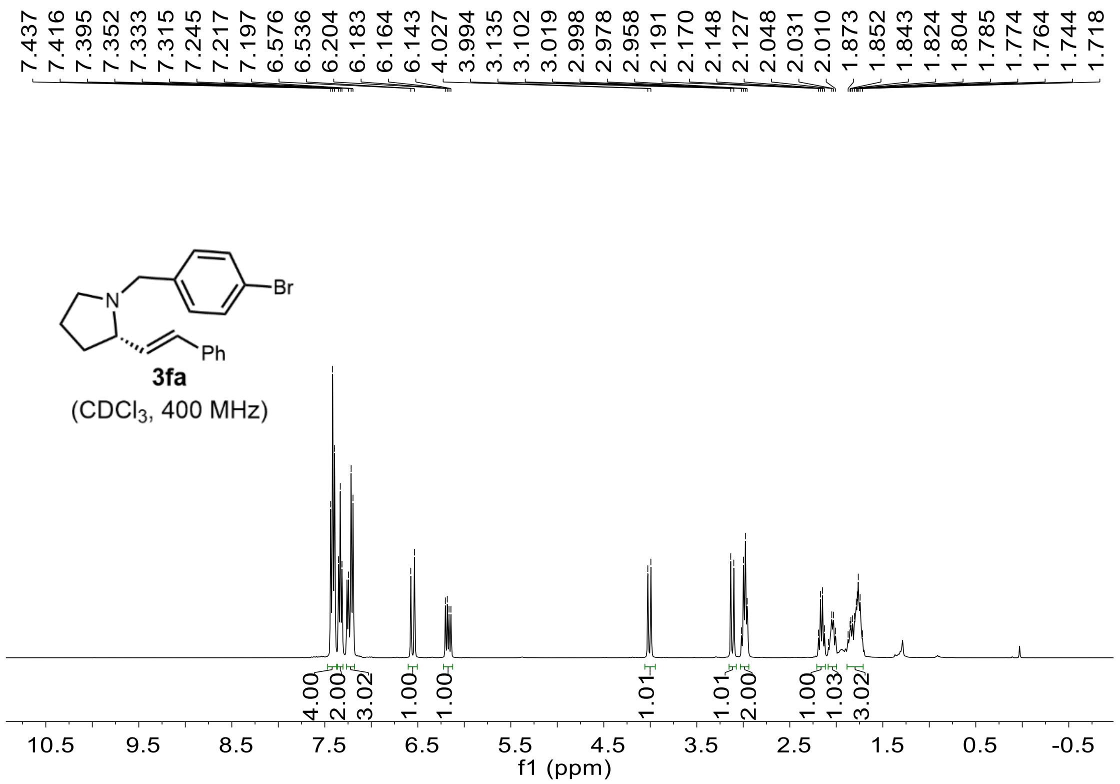
7.437 7.416 7.395 7.352 7.333 7.315 7.245 7.217 7.197 6.576 6.536 6.204 6.183 6.164 6.143 4.027 3.994 3.135 3.102 3.019 2.998 2.978 2.958 2.191 2.170 2.148 2.127 2.048 2.031 2.010 1.873 1.852 1.843 1.824 1.804 1.785 1.774 1.764 1.744 1.718
Br
N
Ph
3fa
(CDCl3, 400 MHz)
4.00 2.00 3.02 1.00 1.00 1.01 1.01 2.00 1.00 1.03 3.02
10.5 9.5 8.5 7.5 6.5 5.5 4.5 3.5 2.5 1.5 0.5 -0.5
f1 (ppm)

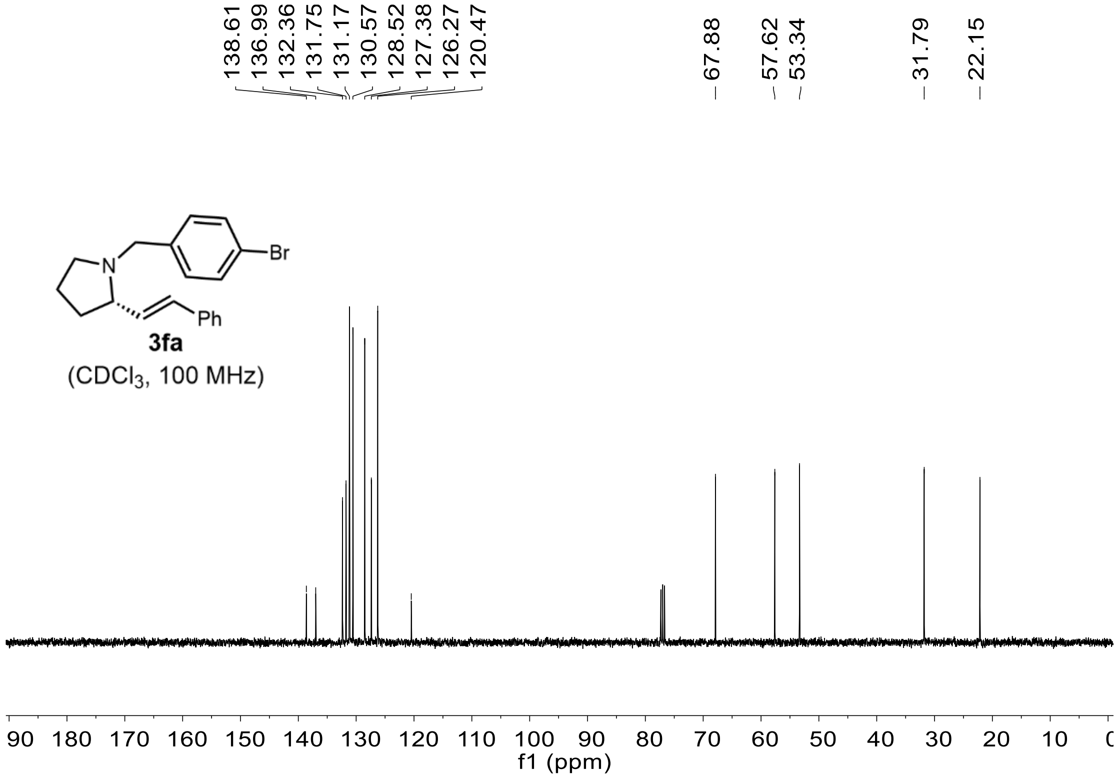
138.61 136.99 132.36 131.75 131.17 130.57 128.52 127.38 126.27 120.47
67.88
57.62 53.34
31.79
22.15
Br
N
Ph
3fa
(CDCl3, 100 MHz)
90 180 170 160 150 140 130 120 110 100 90 80 70 60 50 40 30 20 10
f1 (ppm)

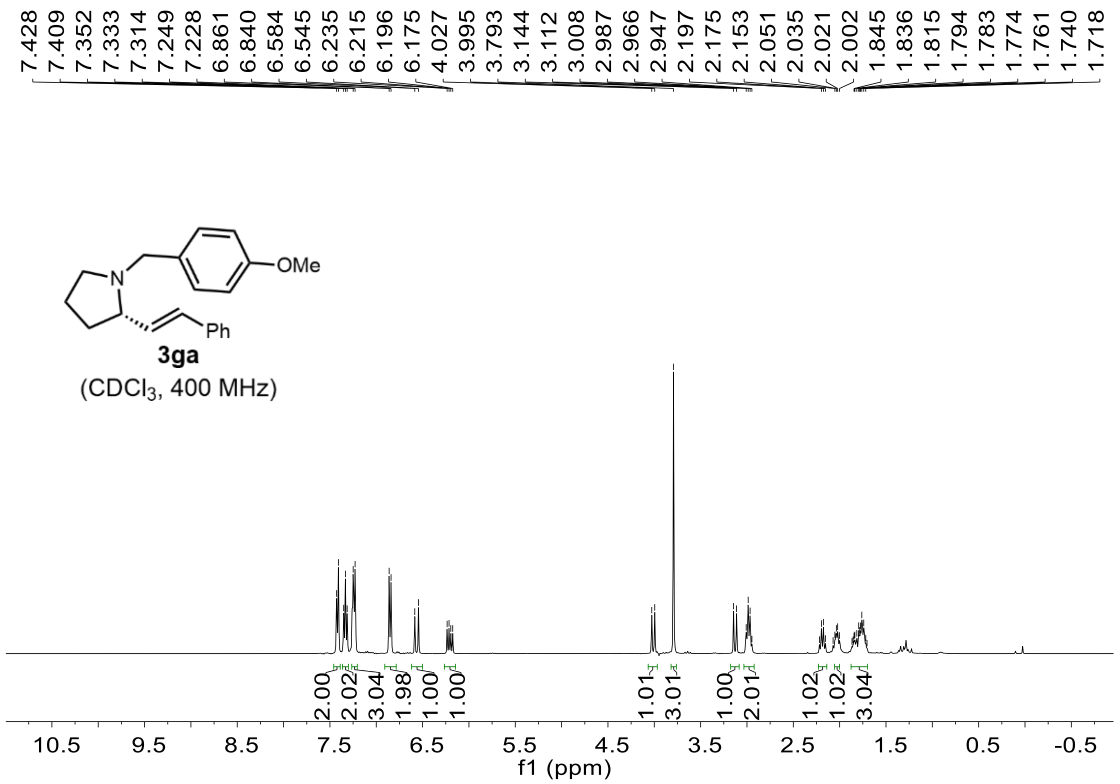
7.428 7.409 7.352 7.333 7.314 7.249 7.228 6.861 6.840 6.584 6.545 6.235 6.215 6.196 6.175 4.027 3.995 3.793 3.144 3.112 3.008 2.987 2.966 2.947 2.197 2.175 2.153 2.051 2.035 2.021 2.002 1.845 1.836 1.815 1.794 1.783 1.774 1.761 1.740 1.718
OMe
N
Ph
3ga
(CDCl3, 400 MHz)
2.00 2.02 3.04 1.98 1.00 1.00 1.01 3.01 1.00 2.01 1.02 1.02 3.04
10.5 9.5 8.5 7.5 6.5 5.5 4.5 3.5 2.5 1.5 0.5 -0.5
f1 (ppm)

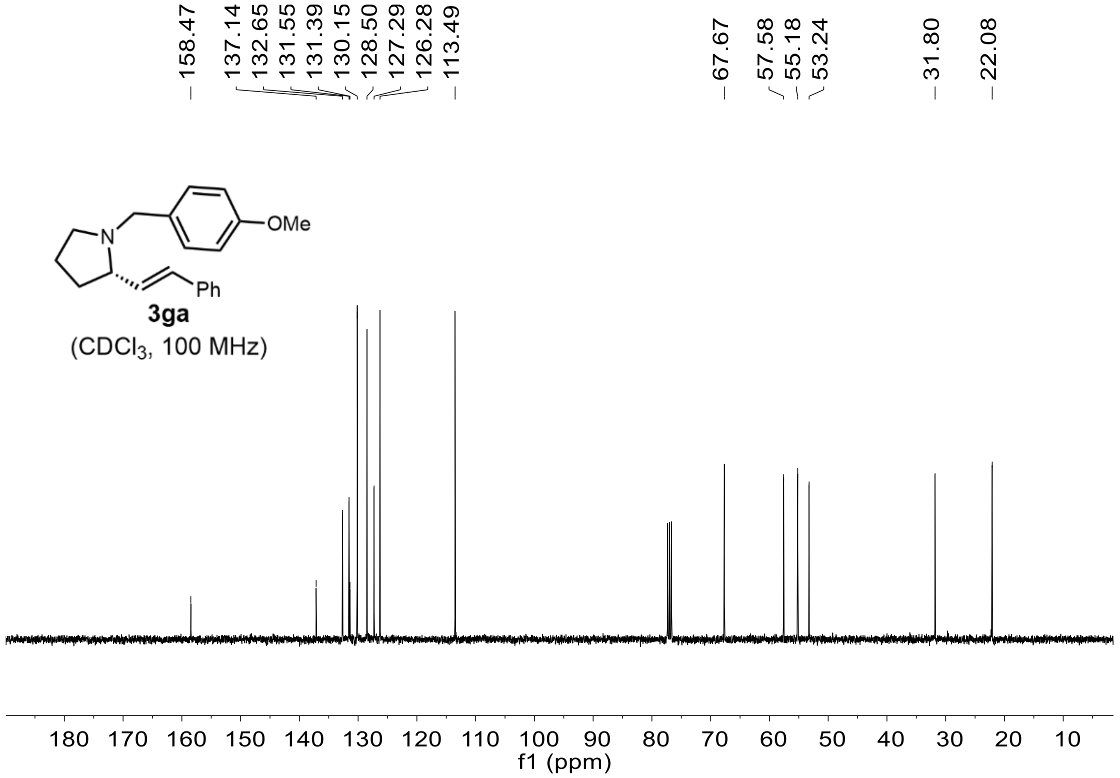
158.47 137.14 132.65 131.55 131.39 130.15 128.50 127.29 126.28 113.49 67.67 57.58 55.18 53.24 31.80 22.08
OMe
N
Ph
3ga
(CDCl3, 100 MHz)
180 170 160 150 140 130 120 110 100 90 80 70 60 50 40 30 20 10
f1 (ppm)

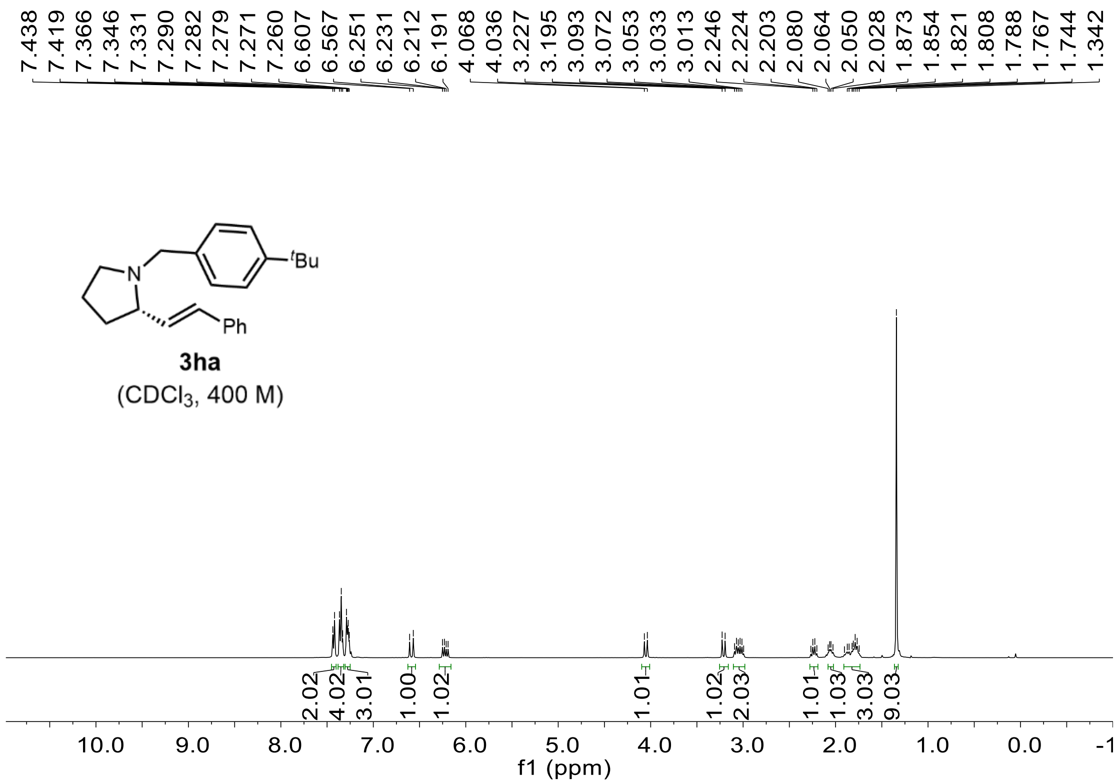
7.438 7.419 7.366 7.346 7.331 7.290 7.282 7.279 7.271 7.260 6.607 6.567 6.251 6.231 6.212 6.191 4.068 4.036 3.227 3.195 3.093 3.072 3.053 3.033 3.013 2.246 2.224 2.203 2.080 2.064 2.050 2.028 1.873 1.854 1.821 1.808 1.788 1.767 1.744 1.342
N
^{t}Bu
Ph
3ha
($CDCl_3$, 400 M)
2.02 4.02 3.01 1.00 1.02 1.01 1.02 2.03 1.01 1.03 3.03 9.03
10.0 9.0 8.0 7.0 6.0 5.0 4.0 3.0 2.0 1.0 0.0 -1
f1 (ppm)

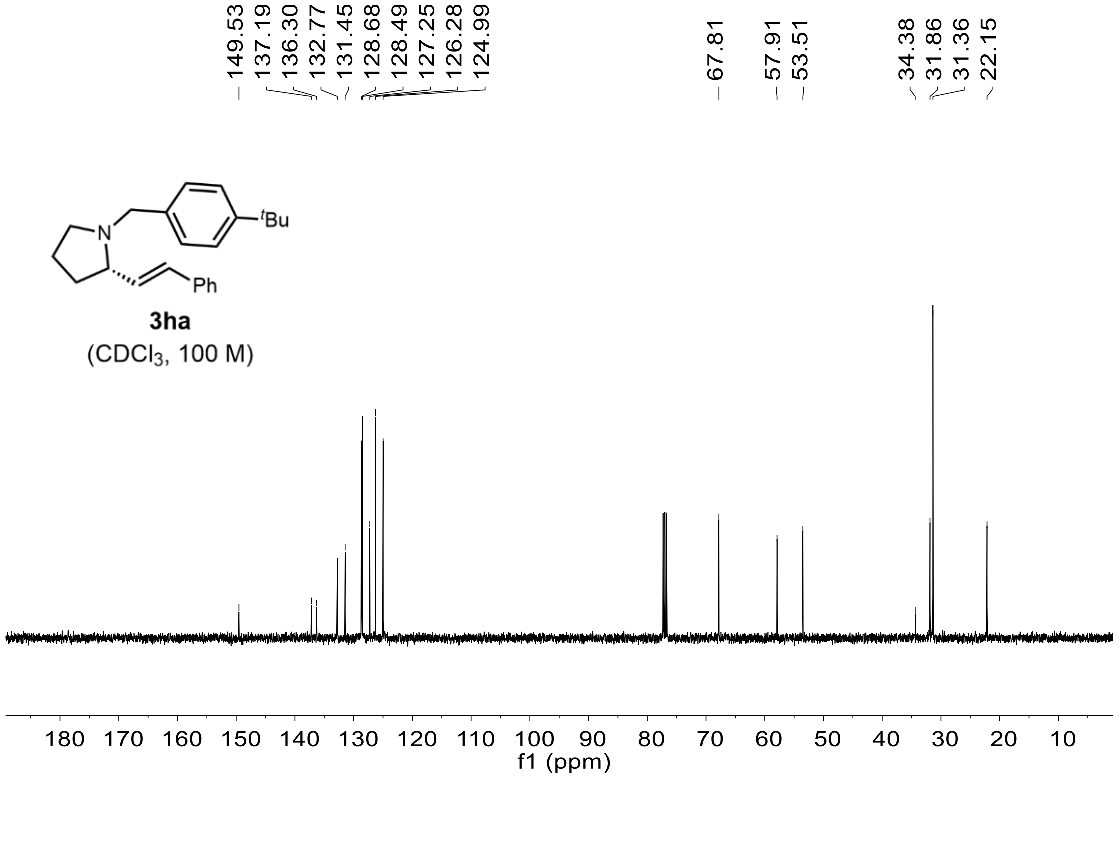
149.53 137.19 136.30 132.77 131.45 128.68 128.49 127.25 126.28 124.99 67.81 57.91 53.51 34.38 31.86 31.36 22.15
N
^{t}Bu
Ph
3ha
($CDCl_3$, 100 M)
180 170 160 150 140 130 120 110 100 90 80 70 60 50 40 30 20 10
f1 (ppm)

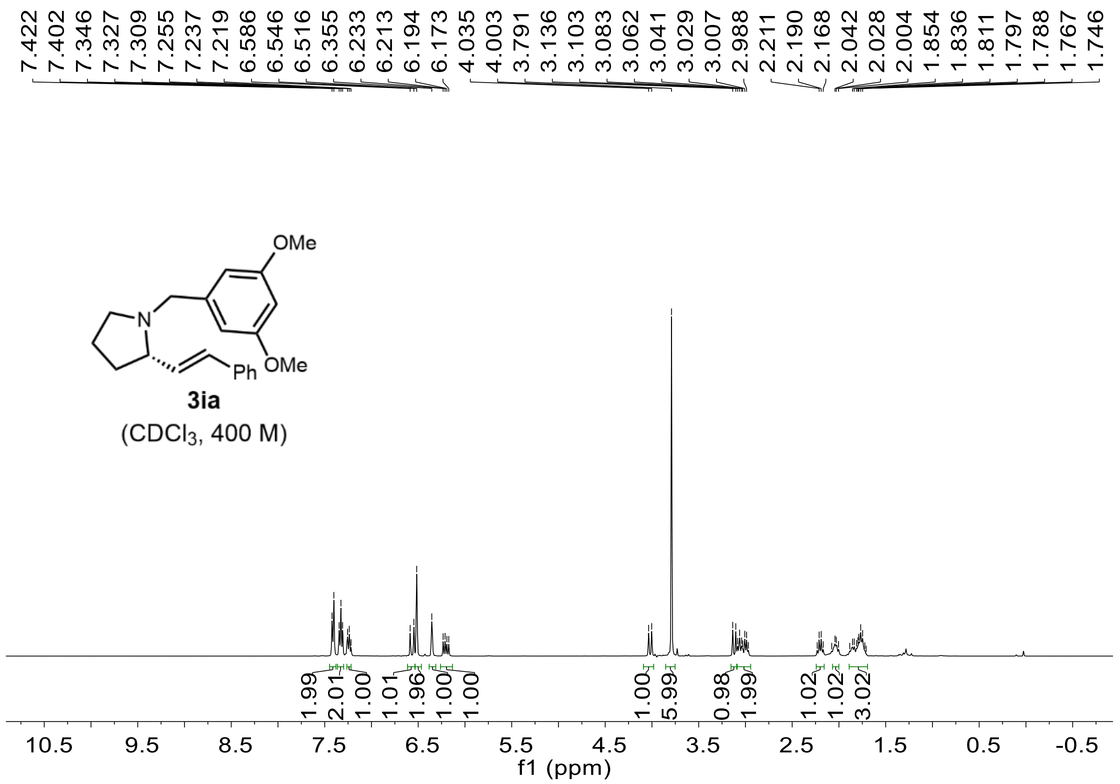
7.422 7.402 7.346 7.327 7.309 7.255 7.237 7.219 6.586 6.546 6.516 6.355 6.233 6.213 6.194 6.173 4.035 4.003 3.791 3.136 3.103 3.083 3.062 3.041 3.029 3.007 2.988 2.211 2.190 2.168 2.042 2.028 2.004 1.854 1.836 1.811 1.797 1.788 1.767 1.746
OMe
N
Ph
OMe
3ia
(CDCl3, 400 M)
1.99 2.01 1.00 1.01 1.96 1.00 1.00 1.00 5.99 0.98 1.99 1.02 1.02 3.02
10.5 9.5 8.5 7.5 6.5 5.5 4.5 3.5 2.5 1.5 0.5 -0.5
f1 (ppm)

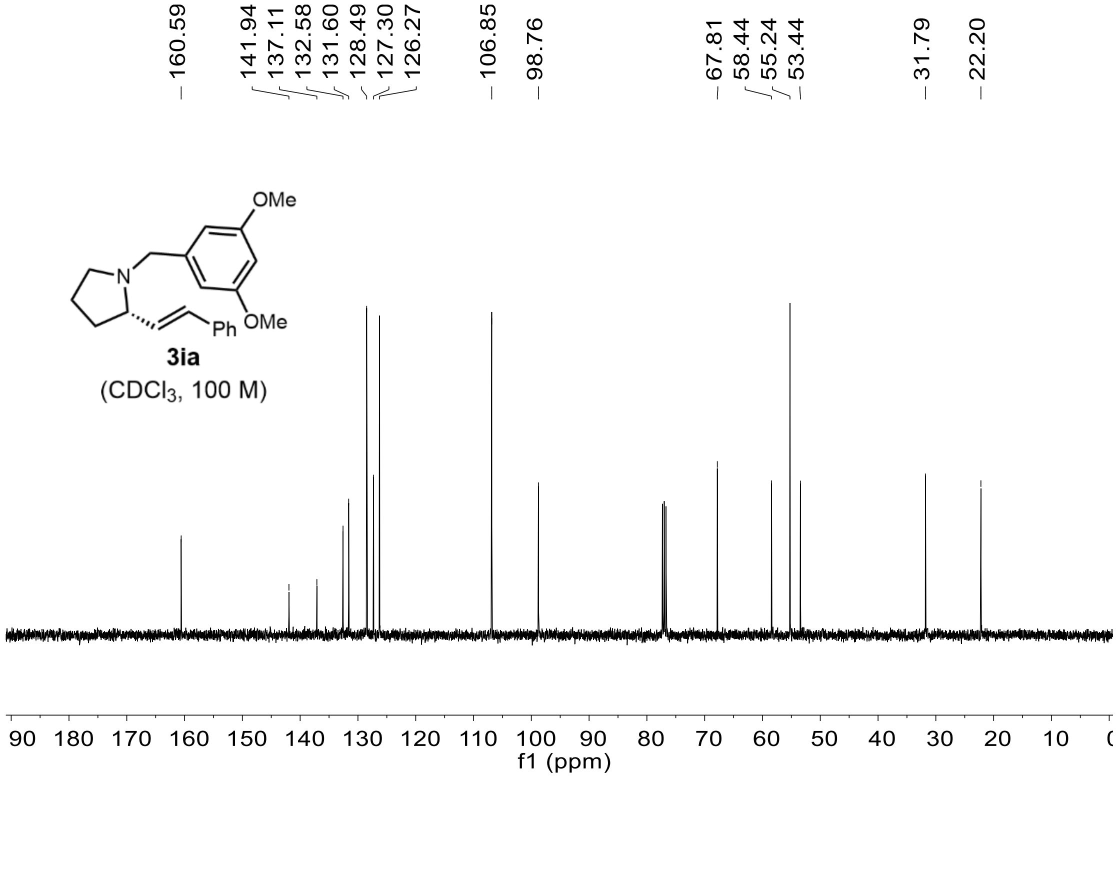
160.59 141.94 137.11 132.58 131.60 128.49 127.30 126.27 106.85 98.76 67.81 58.44 55.24 53.44 31.79 22.20
OMe
N
Ph
OMe
3ia
(CDCl3, 100 M)
90 180 170 160 150 140 130 120 110 100 90 80 70 60 50 40 30 20 10
f1 (ppm)

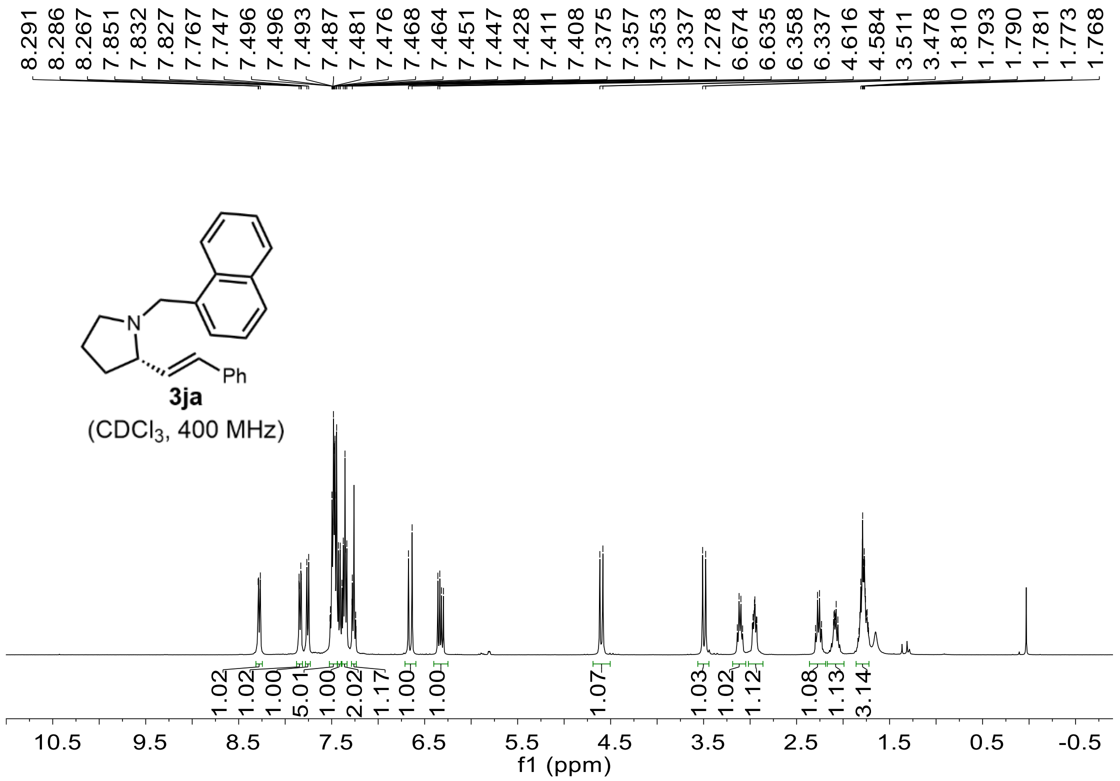
8.291 8.286 8.267 7.851 7.832 7.827 7.767 7.747 7.496 7.496 7.493 7.487 7.481 7.476 7.468 7.464 7.451 7.447 7.428 7.411 7.408 7.375 7.357 7.353 7.337 7.278 6.674 6.635 6.358 6.337 4.616 4.584 3.511 3.478 1.810 1.793 1.790 1.781 1.773 1.768
Ph
3ja
(CDCl3, 400 MHz)
1.02 1.02 1.00 5.01 1.00 2.02 1.17 1.00 1.00 1.07 1.03 1.02 1.12 1.08 1.13 3.14
10.5 9.5 8.5 7.5 6.5 5.5 4.5 3.5 2.5 1.5 0.5 -0.5
f1 (ppm)

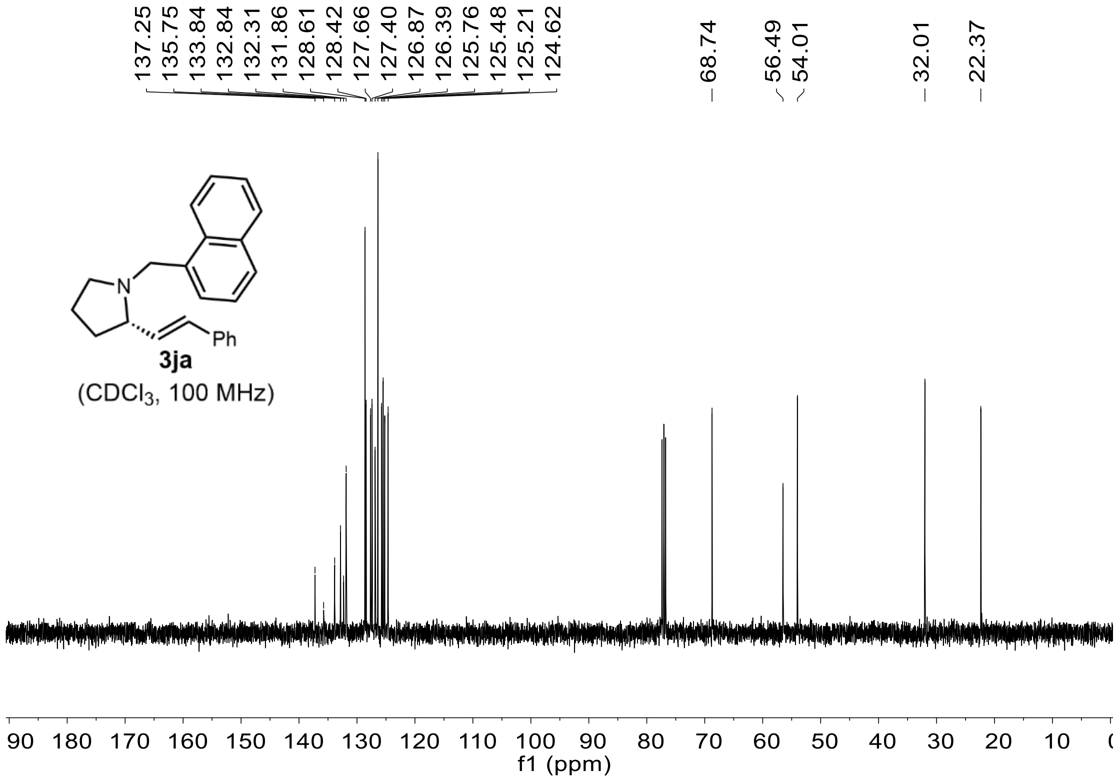
137.25 135.75 133.84 132.84 132.31 131.86 128.61 128.42 127.66 127.40 126.87 126.39 125.76 125.48 125.21 124.62
68.74
56.49 54.01
32.01
22.37
Ph
3ja
(CDCl3, 100 MHz)
90 180 170 160 150 140 130 120 110 100 90 80 70 60 50 40 30 20 10
f1 (ppm)

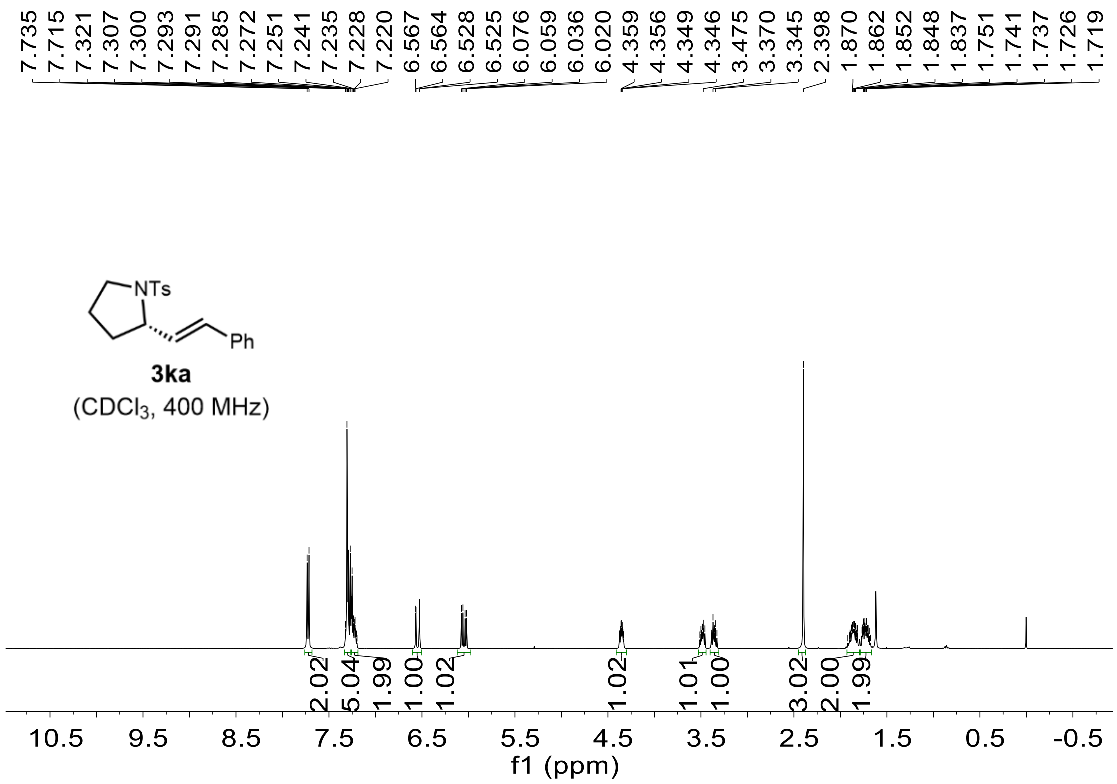
7.735 7.715 7.321 7.307 7.300 7.293 7.291 7.285 7.272 7.251 7.241 7.235 7.228 7.220 6.567 6.564 6.528 6.525 6.076 6.059 6.036 6.020 4.359 4.356 4.349 4.346 3.475 3.370 3.345 2.398 1.870 1.862 1.852 1.848 1.837 1.751 1.741 1.737 1.726 1.719
NTs
Ph
3ka
(CDCl3, 400 MHz)
2.02 5.04 1.99 1.00 1.02 1.02 1.01 1.00 3.02 2.00 1.99
10.5 9.5 8.5 7.5 6.5 5.5 4.5 3.5 2.5 1.5 0.5 -0.5
f1 (ppm)

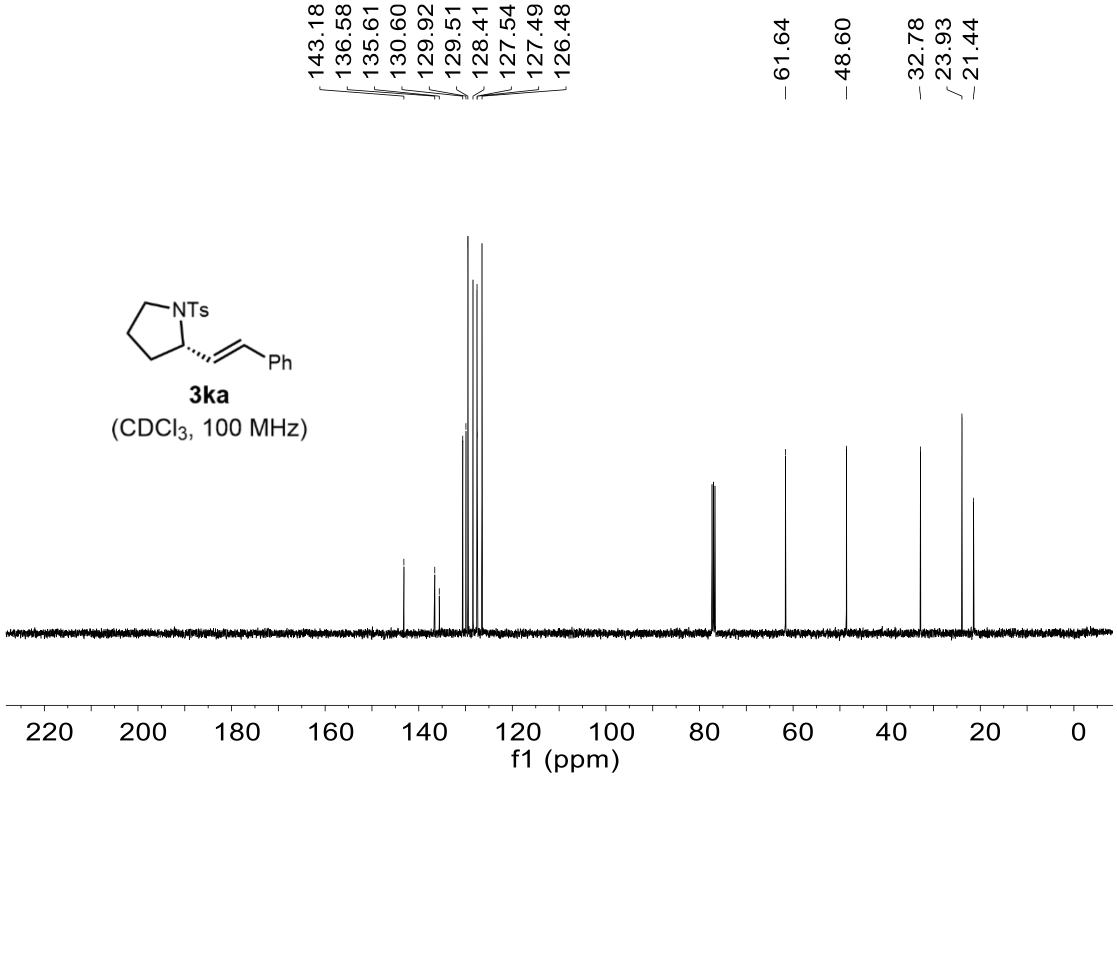
143.18 136.58 135.61 130.60 129.92 129.51 128.41 127.54 127.49 126.48
61.64 48.60 32.78 23.93 21.44
NTs
Ph
3ka
(CDCl3, 100 MHz)
220 200 180 160 140 120 100 80 60 40 20 0
f1 (ppm)

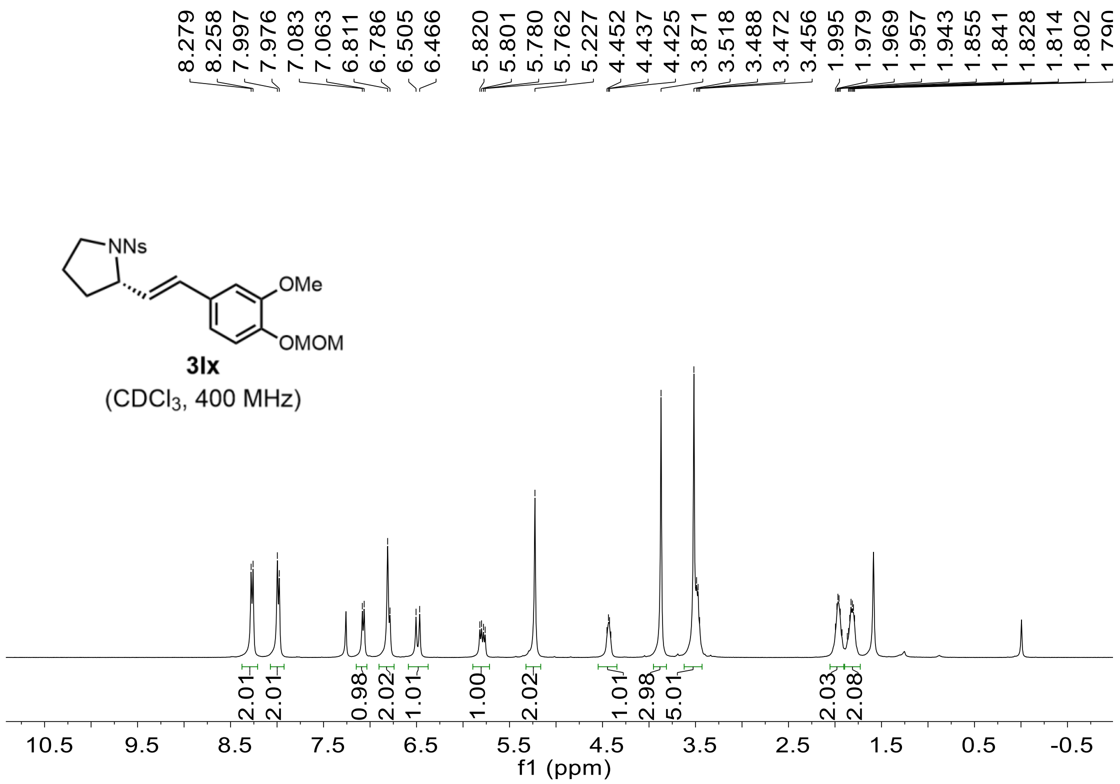
8.279
8.258
7.997
7.976
7.083
7.063
6.811
6.786
6.505
6.466
5.820
5.801
5.780
5.762
5.227
4.452
4.437
4.425
3.871
3.518
3.488
3.472
3.456
1.995
1.979
1.969
1.957
1.943
1.855
1.841
1.828
1.814
1.802
NNs
OMe
OMOM
3lx
(CDCl3, 400 MHz)
2.01
2.01
0.98
2.02
1.01
1.00
2.02
1.01
2.98
5.01
2.03
2.08
10.5
9.5
8.5
7.5
6.5
5.5
4.5
3.5
2.5
1.5
0.5
-0.5
f1 (ppm)

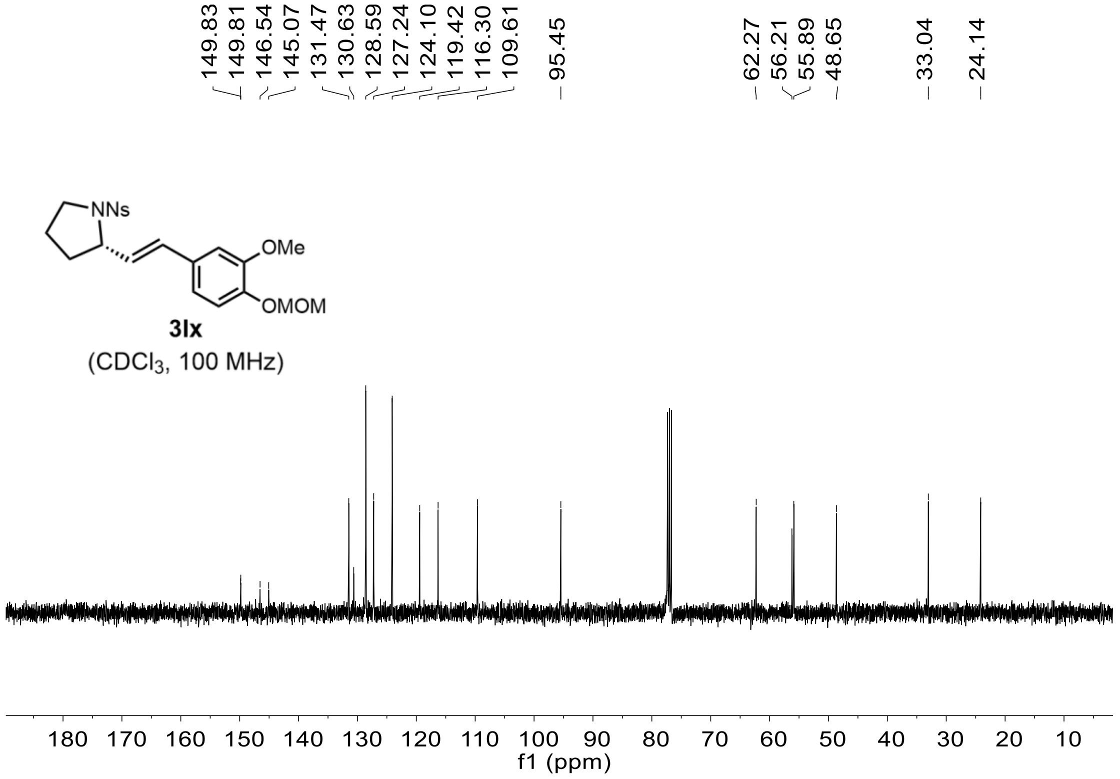
149.83
149.81
146.54
145.07
131.47
130.63
128.59
127.24
124.10
119.42
116.30
109.61
95.45
62.27
56.21
55.89
48.65
33.04
24.14
NNs
OMe
OMOM
3lx
(CDCl3, 100 MHz)
180
170
160
150
140
130
120
110
100
90
80
70
60
50
40
30
20
10
f1 (ppm)

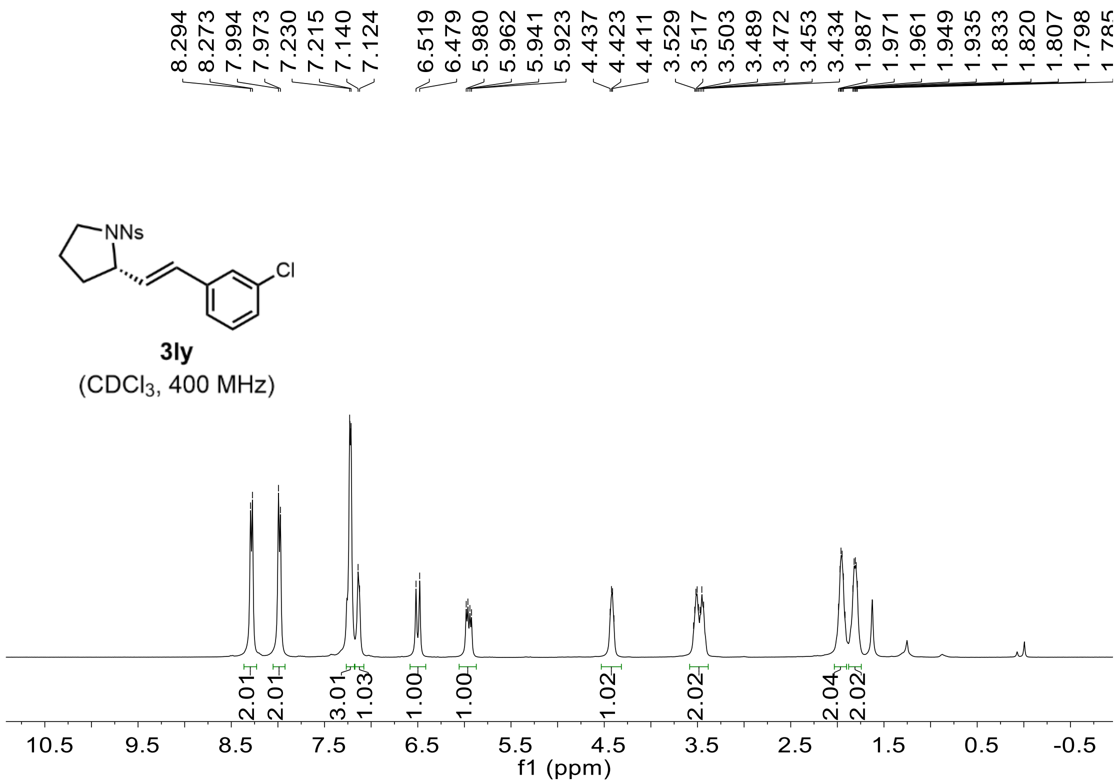
8.294 8.273 7.994 7.973 7.230 7.215 7.140 7.124 6.519 6.479 5.980 5.962 5.941 5.923 4.437 4.423 4.411 3.529 3.517 3.503 3.489 3.472 3.453 3.434 1.987 1.971 1.961 1.949 1.935 1.833 1.820 1.807 1.798
NNs
Cl
3ly
(CDCl3, 400 MHz)
2.01 2.01 3.01 1.03 1.00 1.00 1.02 2.02 2.04 2.02
10.5 9.5 8.5 7.5 6.5 5.5 4.5 3.5 2.5 1.5 0.5 -0.5
f1 (ppm)

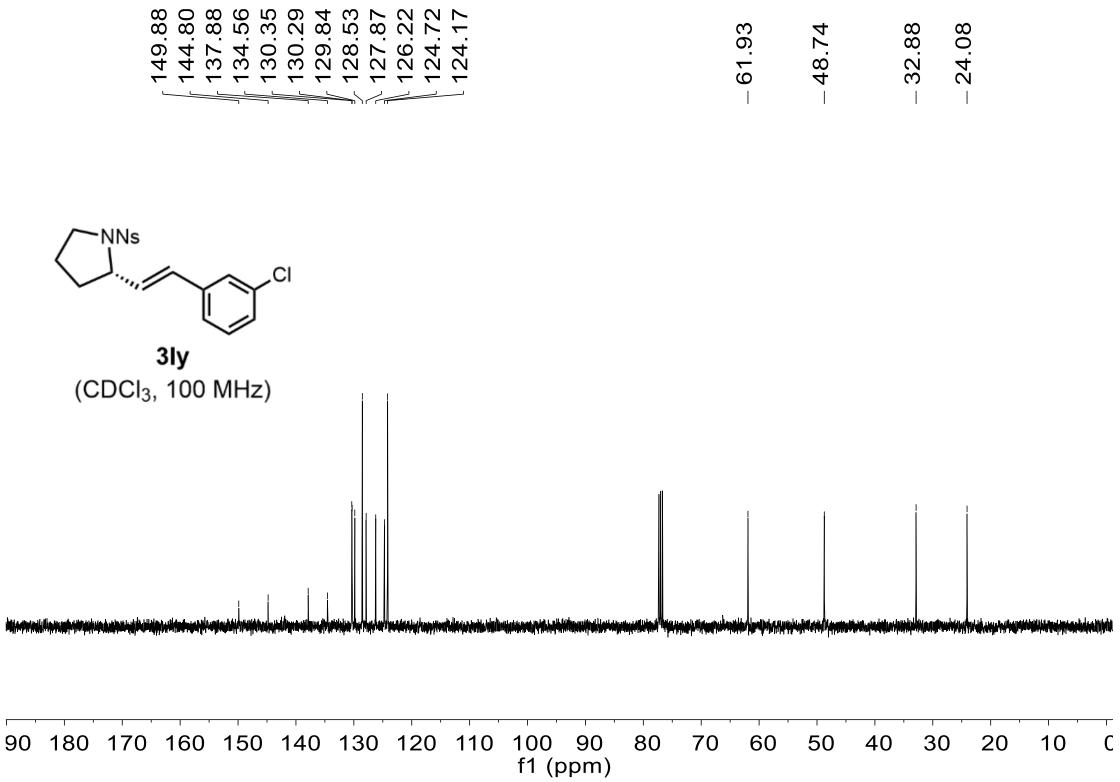
149.88 144.80 137.88 134.56 130.35 130.29 129.84 128.53 127.87 126.22 124.72 124.17
61.93 48.74 32.88 24.08
NNs
Cl
3ly
(CDCl3, 100 MHz)
90 180 170 160 150 140 130 120 110 100 90 80 70 60 50 40 30 20 10
f1 (ppm)

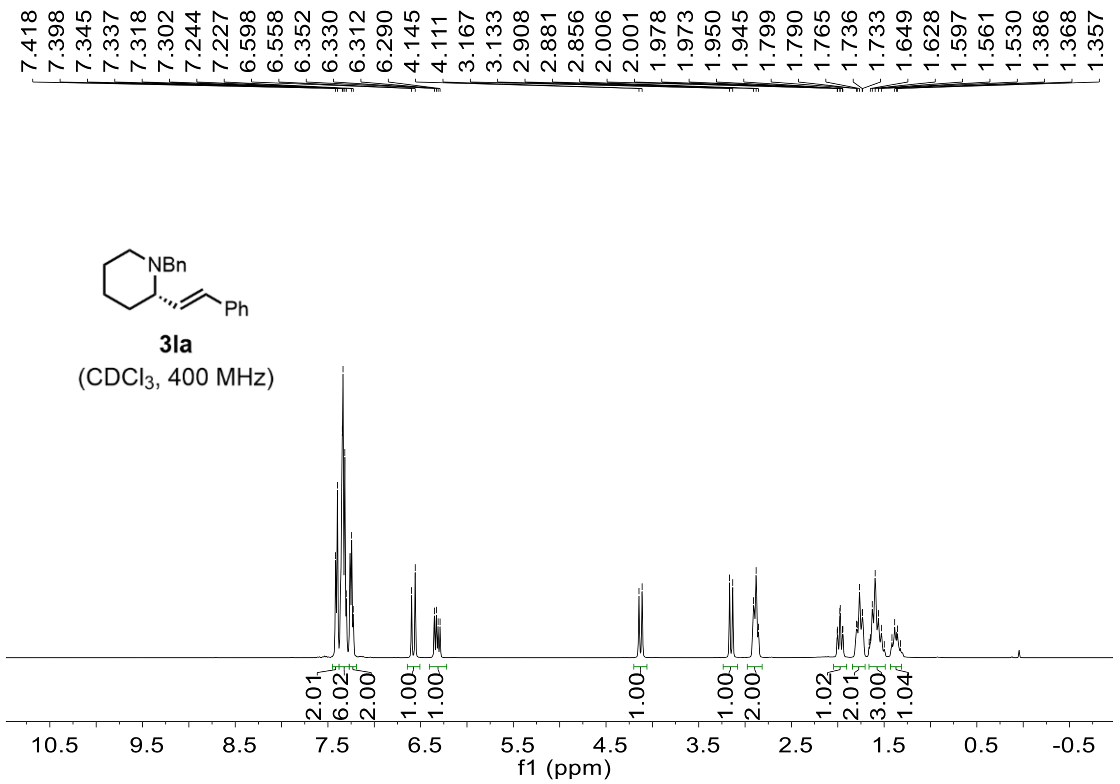
7.418
7.398
7.345
7.337
7.318
7.302
7.244
7.227
6.598
6.558
6.352
6.330
6.312
6.290
4.145
4.111
3.167
3.133
2.908
2.881
2.856
2.006
2.001
1.978
1.973
1.950
1.945
1.799
1.790
1.765
1.736
1.733
1.649
1.628
1.597
1.561
1.530
1.386
1.368
1.357
NBn
Ph
3la
(CDCl3, 400 MHz)
2.01
6.02
2.00
1.00
1.00
1.00
1.00
2.00
1.02
2.01
3.00
1.04
10.5
9.5
8.5
7.5
6.5
5.5
4.5
3.5
2.5
1.5
0.5
-0.5
f1 (ppm)

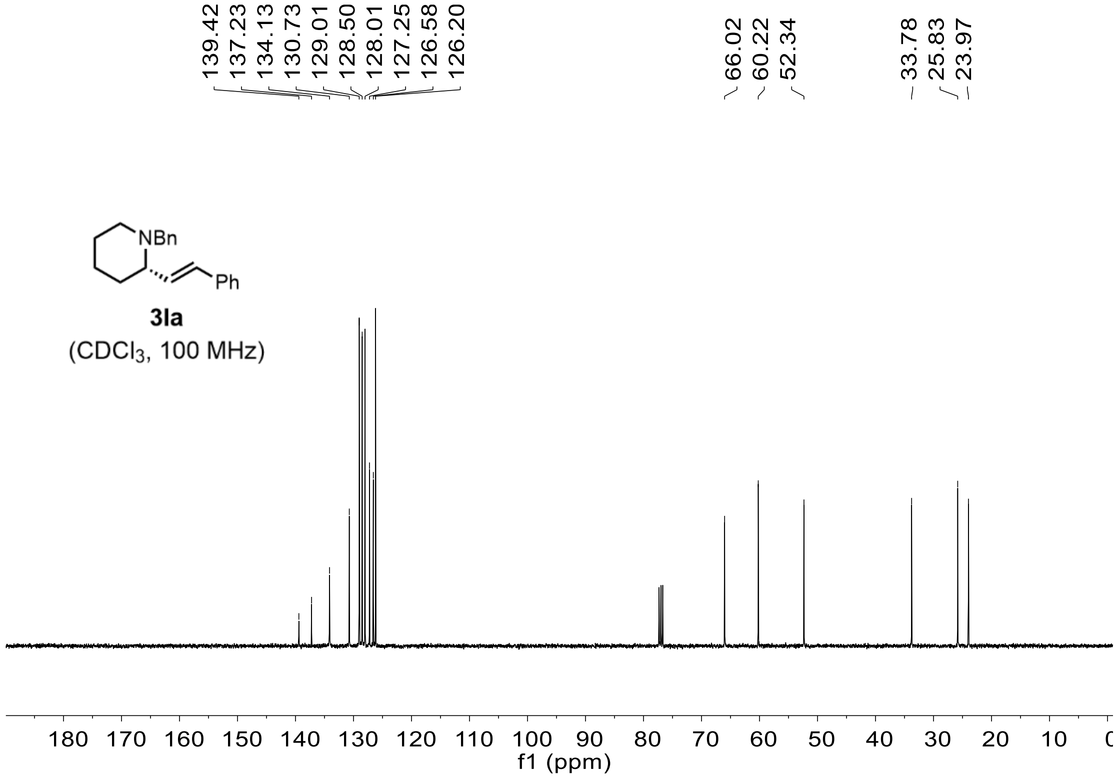
139.42
137.23
134.13
130.73
129.01
128.50
128.01
127.25
126.58
126.20
66.02
60.22
52.34
33.78
25.83
23.97
NBn
Ph
3la
(CDCl3, 100 MHz)
180
170
160
150
140
130
120
110
100
90
80
70
60
50
40
30
20
10
f1 (ppm)

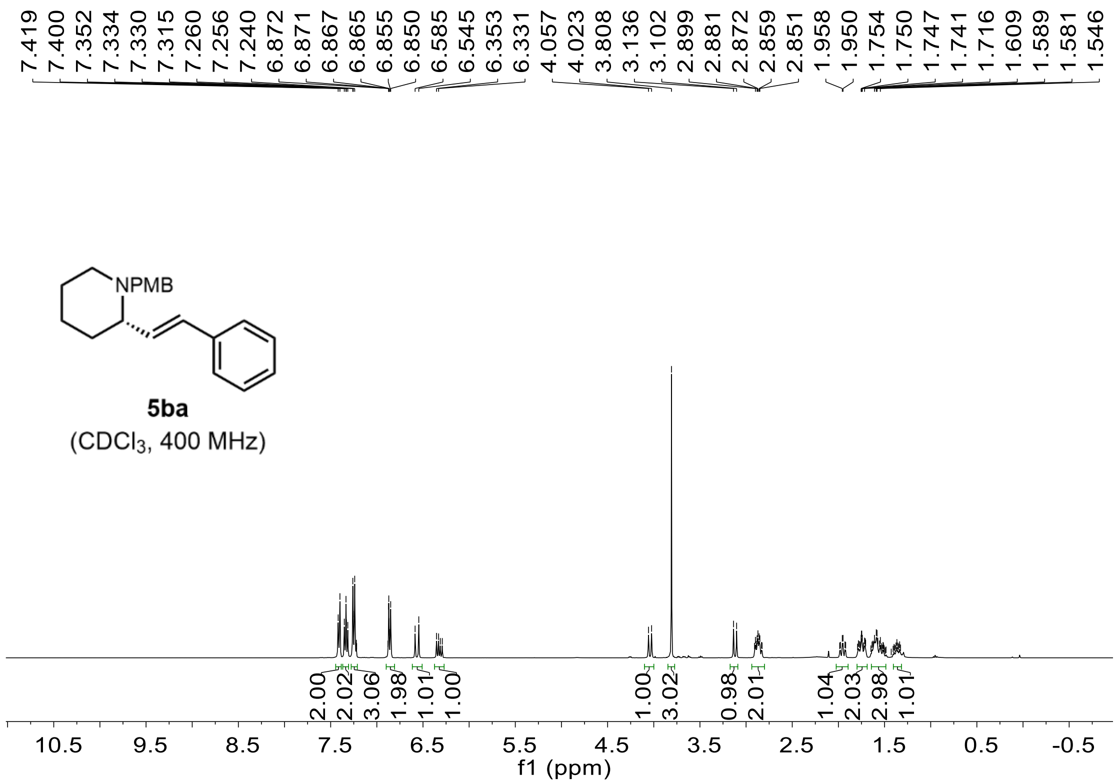
7.419 7.400 7.352 7.334 7.330 7.315 7.260 7.256 7.240 6.872 6.871 6.867 6.865 6.855 6.850 6.585 6.545 6.353 6.331 4.057 4.023 3.808 3.136 3.102 2.899 2.881 2.872 2.859 2.851 1.958 1.950 1.754 1.750 1.747 1.741 1.716 1.609 1.589 1.581 1.546
NPMB
5ba
(CDCl$_3$, 400 MHz)
2.00 2.02 3.06 1.98 1.01 1.00 1.00 3.02 0.98 2.01 1.04 2.03 2.98 1.01
10.5 9.5 8.5 7.5 6.5 5.5 4.5 3.5 2.5 1.5 0.5 -0.5
f1 (ppm)

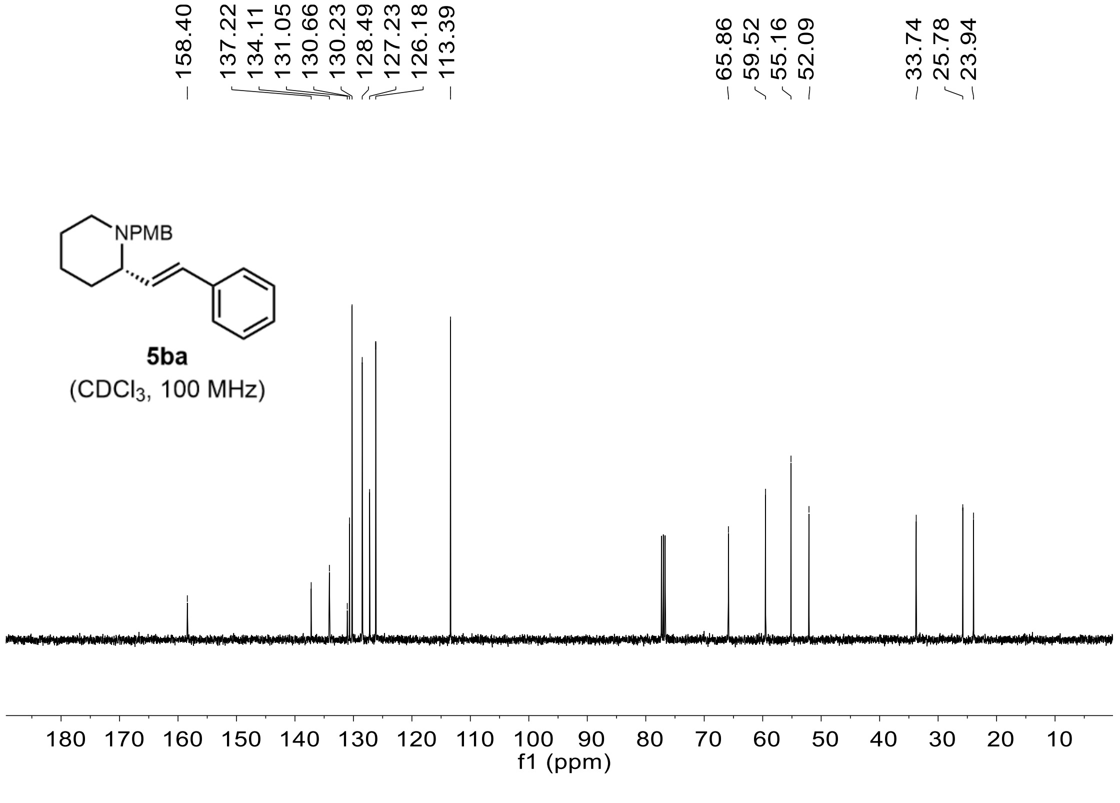
158.40 137.22 134.11 131.05 130.66 130.23 128.49 127.23 126.18 113.39 65.86 59.52 55.16 52.09 33.74 25.78 23.94
NPMB
5ba
(CDCl$_3$, 100 MHz)
180 170 160 150 140 130 120 110 100 90 80 70 60 50 40 30 20 10
f1 (ppm)

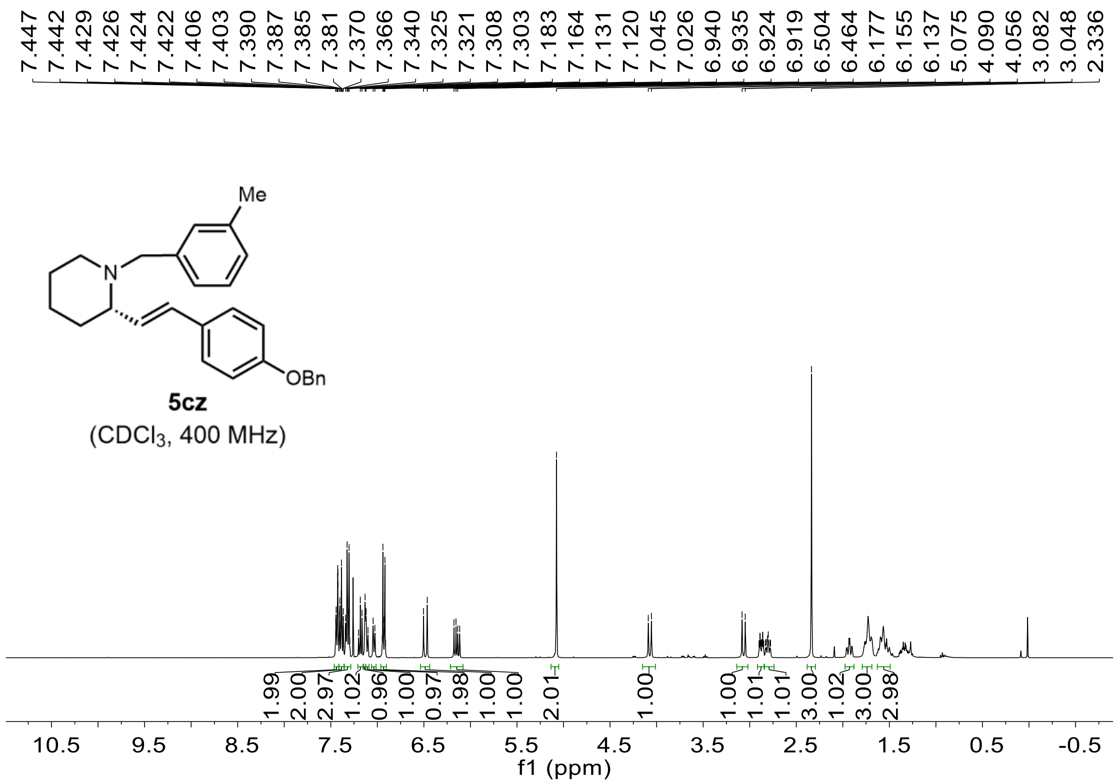
7.447 7.442 7.429 7.426 7.424 7.422 7.406 7.403 7.390 7.387 7.385 7.381 7.370 7.366 7.340 7.325 7.321 7.308 7.303 7.183 7.164 7.131 7.120 7.045 7.026 6.940 6.935 6.924 6.919 6.504 6.464 6.177 6.155 6.137 5.075 4.090 4.056 3.082 3.048 2.336
Me
N
OBn
5cz
(CDCl3, 400 MHz)
1.99 2.00 2.97 1.02 0.96 1.00 0.97 1.98 1.00 1.00 2.01 1.00 1.00 1.01 1.01 3.00 1.02 3.00 2.98
10.5 9.5 8.5 7.5 6.5 5.5 4.5 3.5 2.5 1.5 0.5 -0.5
f1 (ppm)

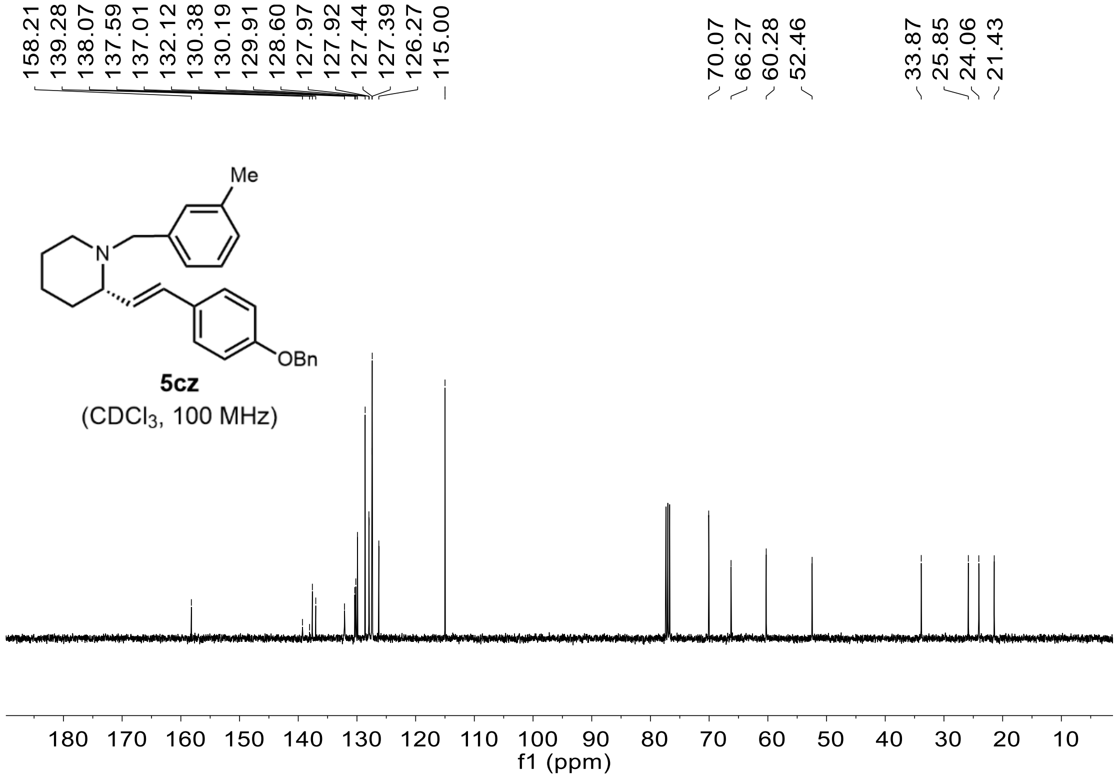
158.21 139.28 138.07 137.59 137.01 132.12 130.38 130.19 129.91 128.60 127.97 127.92 127.44 127.39 126.27 115.00
70.07 66.27 60.28 52.46
33.87 25.85 24.06 21.43
Me
N
OBn
5cz
(CDCl3, 100 MHz)
180 170 160 150 140 130 120 110 100 90 80 70 60 50 40 30 20 10
f1 (ppm)

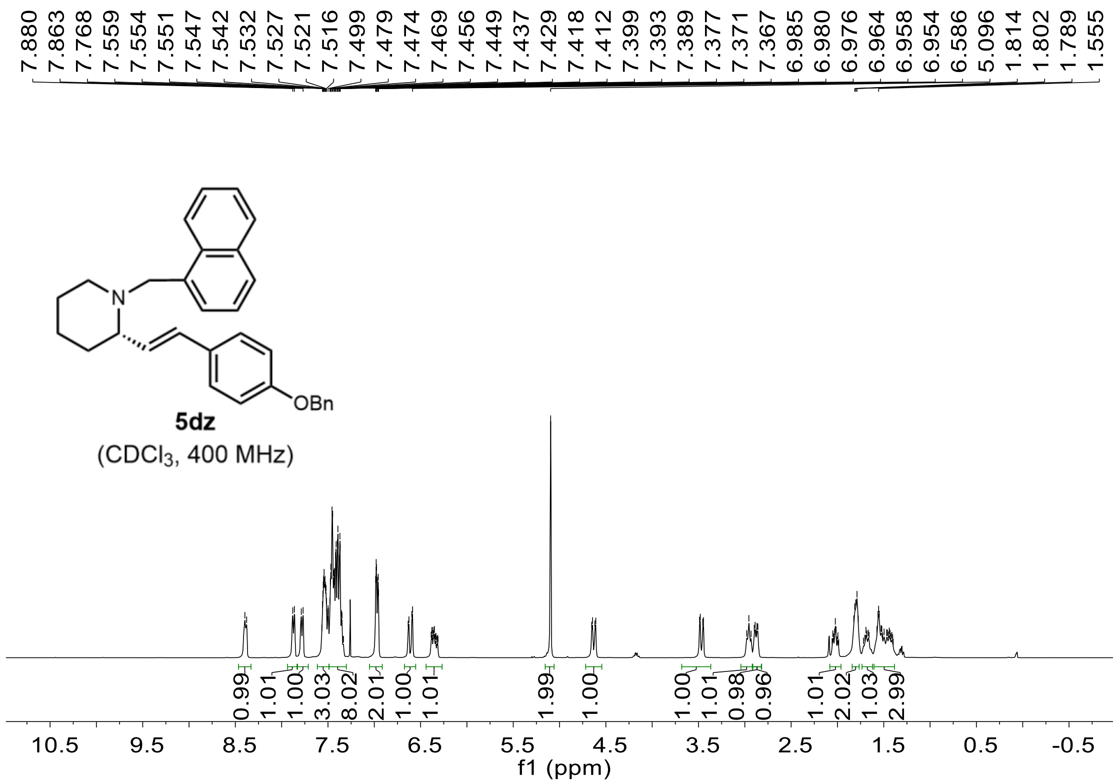
7.880 7.863 7.768 7.559 7.554 7.551 7.547 7.542 7.532 7.527 7.521 7.516 7.499 7.479 7.474 7.469 7.456 7.449 7.437 7.429 7.418 7.412 7.399 7.393 7.389 7.377 7.371 7.367 6.985 6.980 6.976 6.964 6.958 6.954 6.586 5.096 1.814 1.802 1.789 1.555
N
OBn
5dz
(CDCl$_3$, 400 MHz)
0.99 1.01 1.00 3.03 8.02 2.01 1.00 1.01 1.99 1.00 1.00 1.01 0.98 0.96 1.01 2.02 1.03 2.99
10.5 9.5 8.5 7.5 6.5 5.5 4.5 3.5 2.5 1.5 0.5 -0.5
f1 (ppm)

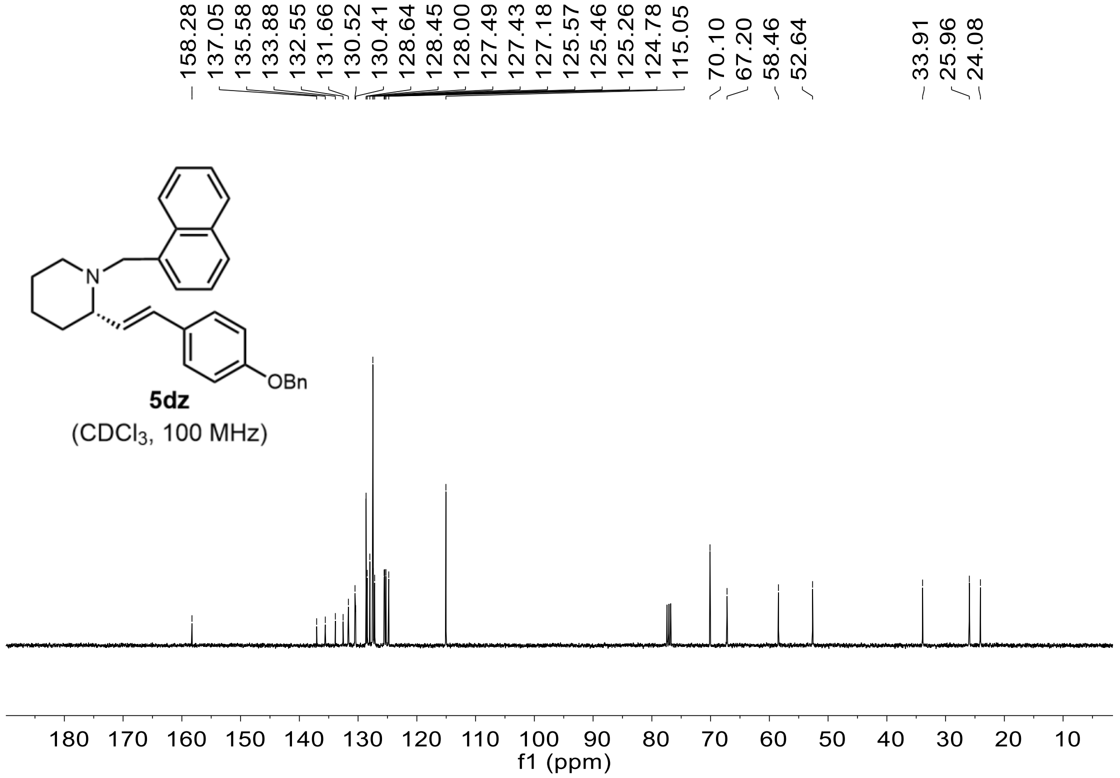
158.28 137.05 135.58 133.88 132.55 131.66 130.52 130.41 128.64 128.45 128.00 127.49 127.43 127.18 125.57 125.46 125.26 124.78 115.05 70.10 67.20 58.46 52.64 33.91 25.96 24.08
N
OBn
5dz
(CDCl$_3$, 100 MHz)
180 170 160 150 140 130 120 110 100 90 80 70 60 50 40 30 20 10
f1 (ppm)

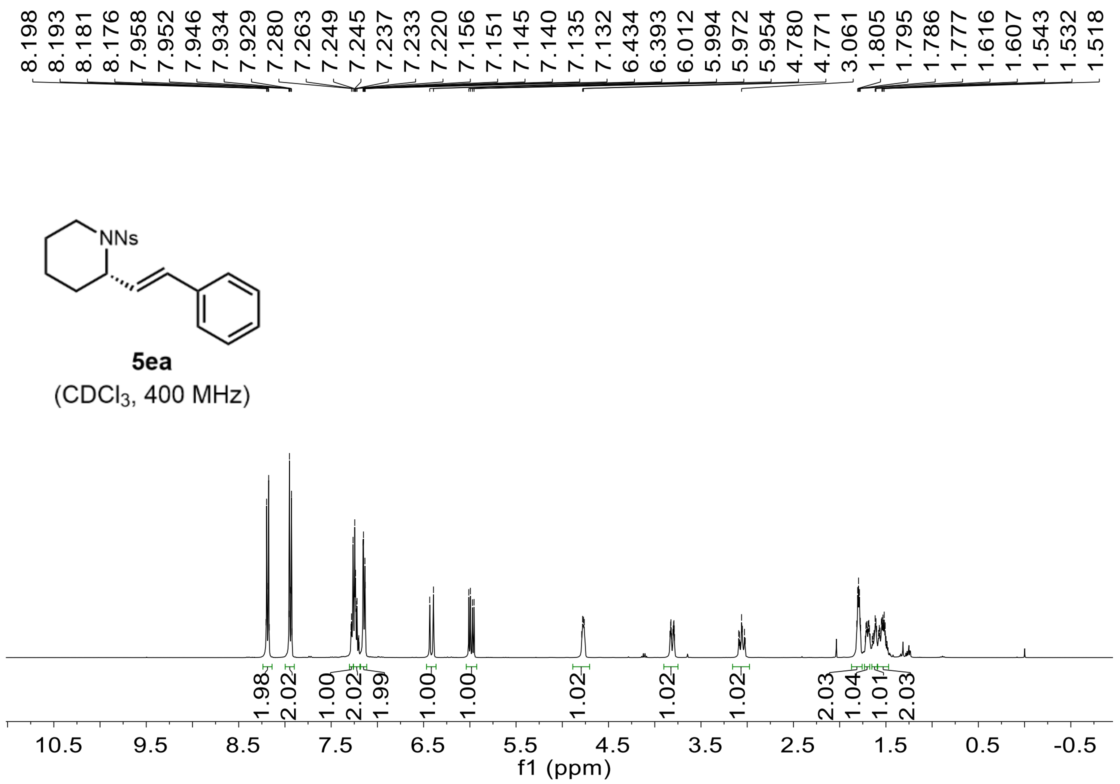
8.198 8.193 8.181 8.176 7.958 7.952 7.946 7.934 7.929 7.280 7.263 7.249 7.245 7.237 7.233 7.220 7.156 7.151 7.145 7.140 7.135 7.132 6.434 6.393 6.012 5.994 5.972 5.954 4.780 4.771 3.061 1.805 1.795 1.786 1.777 1.616 1.607 1.543 1.532 1.518
NNs
5ea
(CDCl3, 400 MHz)
1.98 2.02 1.00 2.02 1.99 1.00 1.00 1.02 1.02 1.02 2.03 1.04 1.01 2.03
10.5 9.5 8.5 7.5 6.5 5.5 4.5 3.5 2.5 1.5 0.5 -0.5
f1 (ppm)

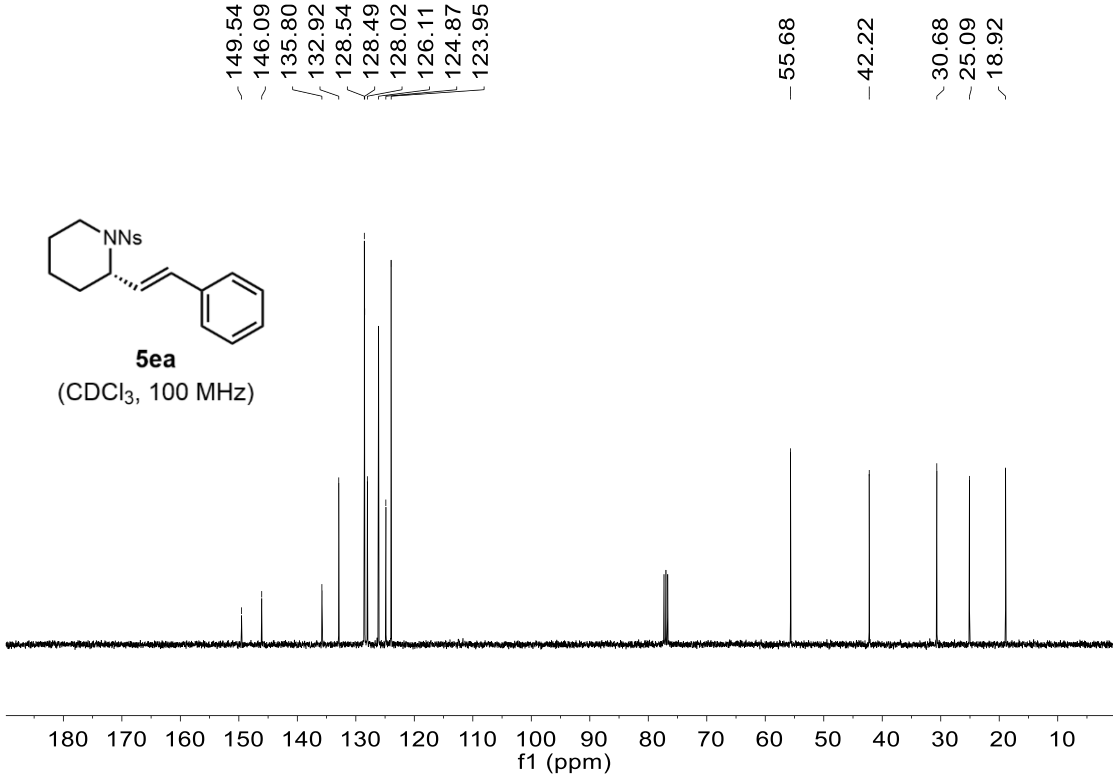
149.54 146.09 135.80 132.92 128.54 128.49 128.02 126.11 124.87 123.95
55.68
42.22
30.68 25.09 18.92
NNs
5ea
(CDCl3, 100 MHz)
180 170 160 150 140 130 120 110 100 90 80 70 60 50 40 30 20 10
f1 (ppm)

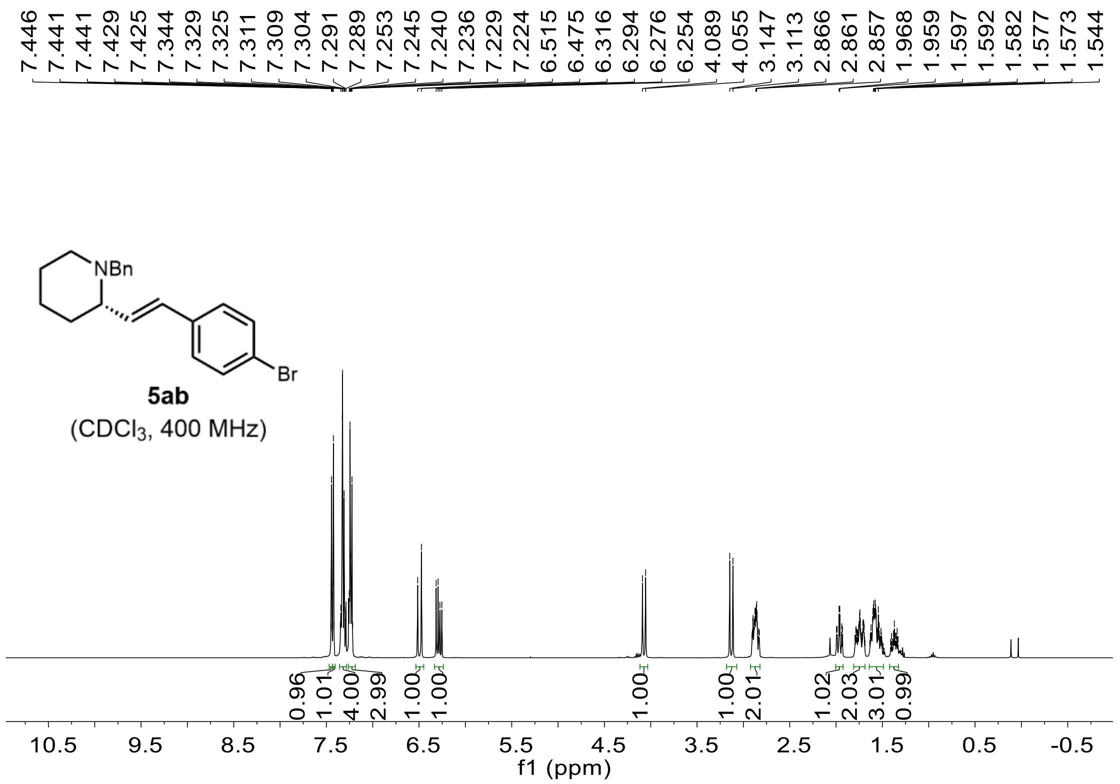
7.446
7.441
7.441
7.429
7.425
7.344
7.329
7.325
7.311
7.309
7.304
7.291
7.289
7.253
7.245
7.240
7.236
7.229
7.224
6.515
6.475
6.316
6.294
6.276
6.254
4.089
4.055
3.147
3.113
2.866
2.861
2.857
1.968
1.959
1.597
1.592
1.582
1.577
1.573
1.544
NBn
Br
5ab
(CDCl3, 400 MHz)
0.96
1.01
4.00
2.99
1.00
1.00
1.00
1.00
2.01
1.02
2.03
3.01
0.99
10.5
9.5
8.5
7.5
6.5
5.5
4.5
3.5
2.5
1.5
0.5
-0.5
f1 (ppm)

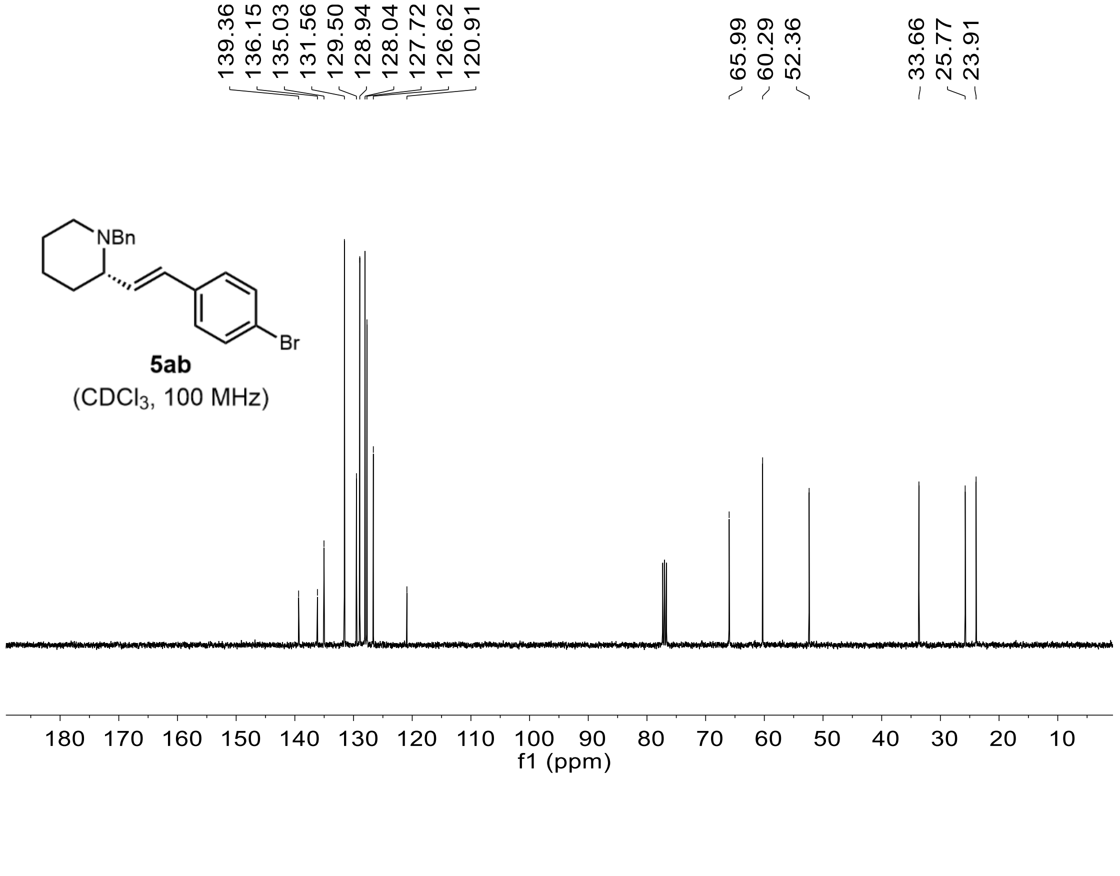
139.36
136.15
135.03
131.56
129.50
128.94
128.04
127.72
126.62
120.91
65.99
60.29
52.36
33.66
25.77
23.91
NBn
Br
5ab
(CDCl3, 100 MHz)
180
170
160
150
140
130
120
110
100
90
80
70
60
50
40
30
20
10
f1 (ppm)

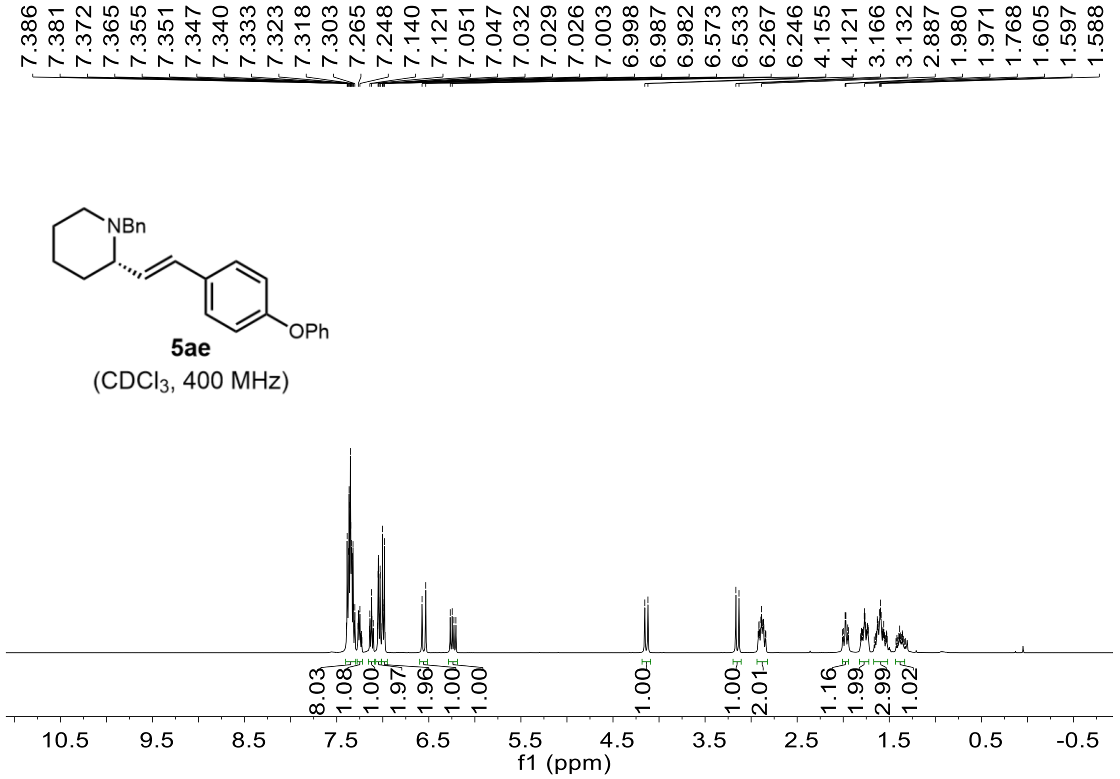
7.386
7.381
7.372
7.365
7.355
7.351
7.347
7.340
7.333
7.323
7.318
7.303
7.265
7.248
7.140
7.121
7.051
7.047
7.032
7.029
7.026
7.003
6.998
6.987
6.982
6.573
6.533
6.267
6.246
4.155
4.121
3.166
3.132
2.887
1.980
1.971
1.768
1.605
1.597
1.588
NBn
OPh
5ae
(CDCl3, 400 MHz)
8.03
1.08
1.00
1.97
1.96
1.00
1.00
1.00
1.00
2.01
1.16
1.99
2.99
1.02
10.5
9.5
8.5
7.5
6.5
5.5
4.5
3.5
2.5
1.5
0.5
-0.5
f1 (ppm)
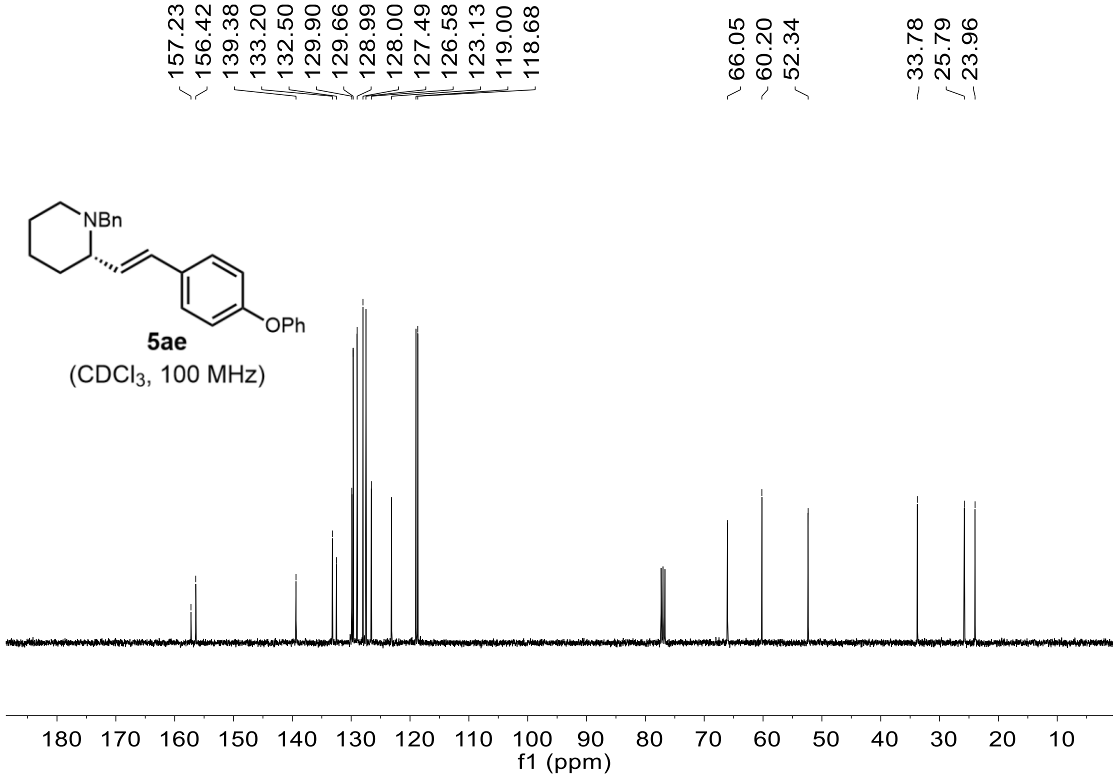
157.23
156.42
139.38
133.20
132.50
129.90
129.66
128.99
128.00
127.49
126.58
123.13
119.00
118.68
66.05
60.20
52.34
33.78
25.79
23.96
NBn
OPh
5ae
(CDCl3, 100 MHz)
180
170
160
150
140
130
120
110
100
90
80
70
60
50
40
30
20
10
f1 (ppm)

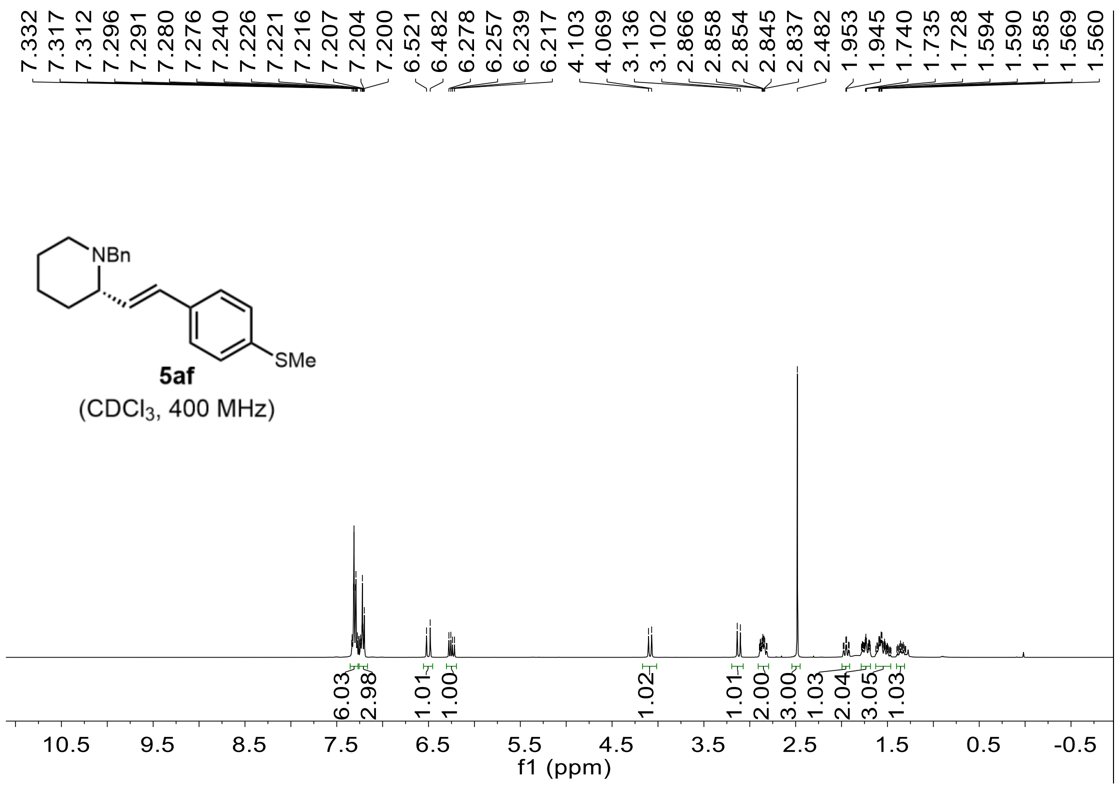
7.332 7.317 7.312 7.296 7.291 7.280 7.276 7.240 7.226 7.221 7.216 7.207 7.204 7.200 6.521 6.482 6.278 6.257 6.239 6.217 4.103 4.069 3.136 3.102 2.866 2.858 2.854 2.845 2.837 2.482 1.953 1.945 1.740 1.735 1.728 1.594 1.590 1.585 1.569 1.560
NBn
SMe
5af
(CDCl3, 400 MHz)
6.03 2.98 1.01 1.00 1.02 1.01 2.00 3.00 1.03 2.04 3.05 1.03
f1 (ppm)

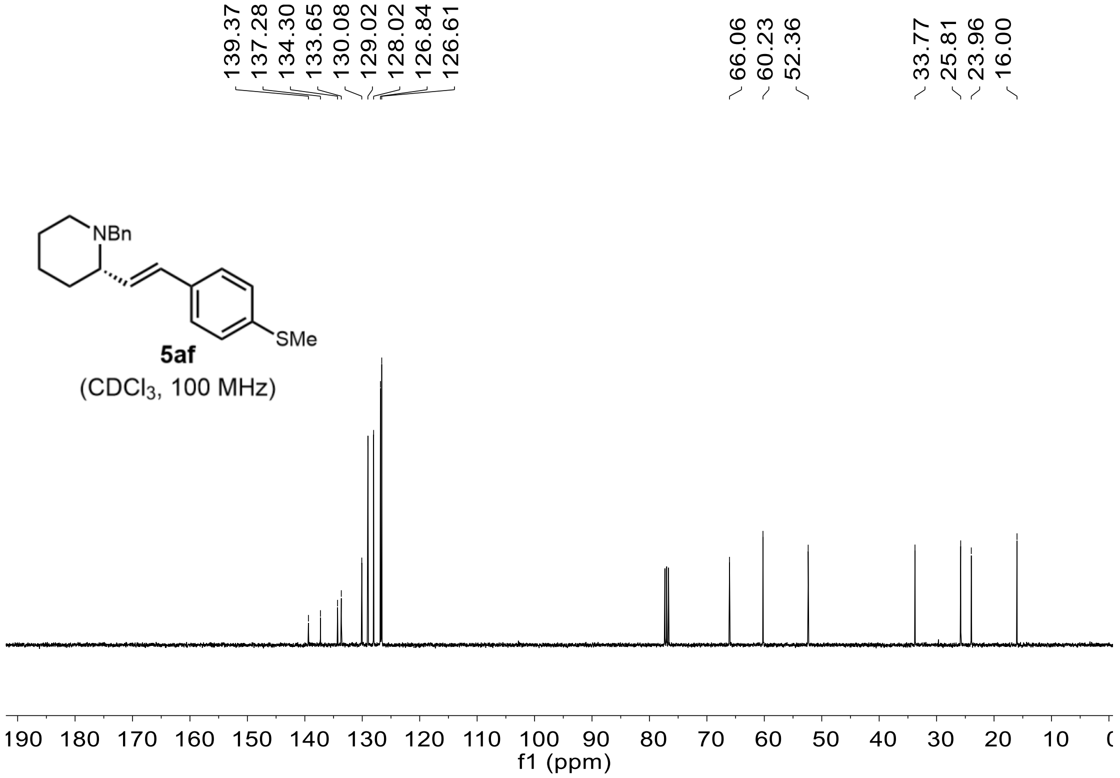
139.37 137.28 134.30 133.65 130.08 129.02 128.02 126.84 126.61
66.06 60.23 52.36
33.77 25.81 23.96 16.00
NBn
SMe
5af
(CDCl3, 100 MHz)
f1 (ppm)

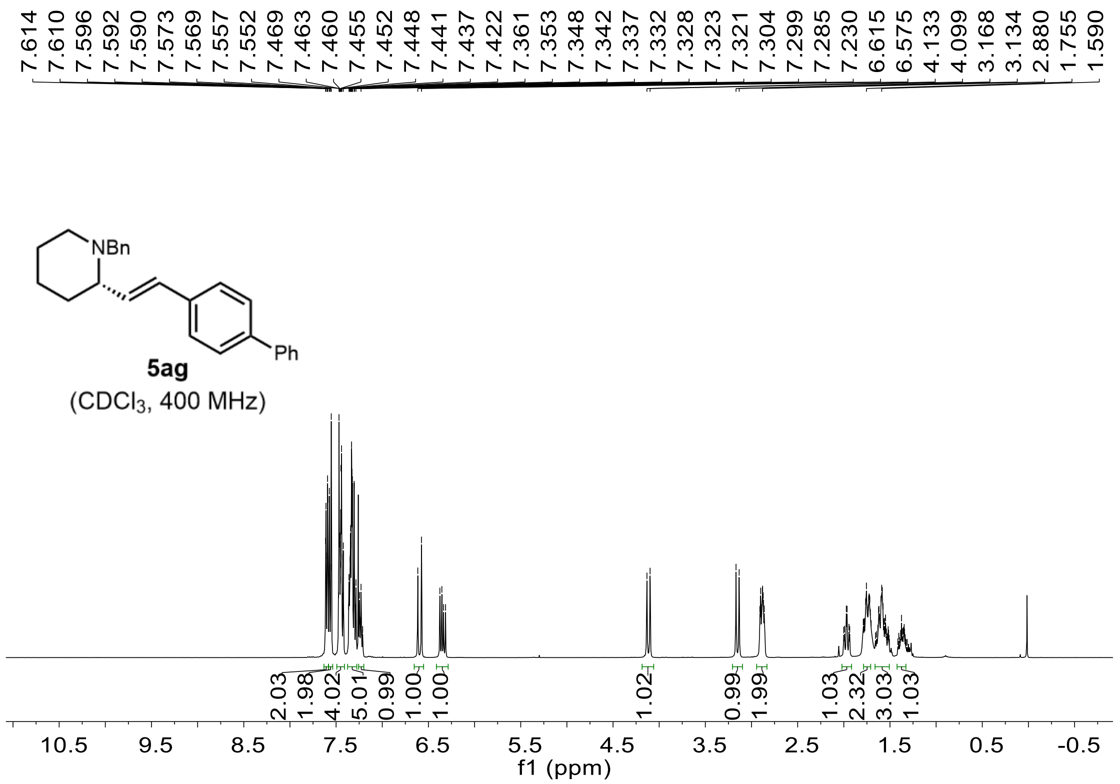
7.614 7.610 7.596 7.592 7.590 7.573 7.569 7.557 7.552 7.469 7.463 7.460 7.455 7.452 7.448 7.441 7.437 7.422 7.361 7.353 7.348 7.342 7.337 7.332 7.328 7.323 7.321 7.304 7.299 7.285 7.230 6.615 6.575 4.133 4.099 3.168 3.134 2.880 1.755 1.590
NBn
Ph
5ag
(CDCl3, 400 MHz)
2.03 1.98 4.02 5.01 0.99 1.00 1.00 1.02 0.99 1.99 1.03 2.32 3.03 1.03
10.5 9.5 8.5 7.5 6.5 5.5 4.5 3.5 2.5 1.5 0.5 -0.5
f1 (ppm)

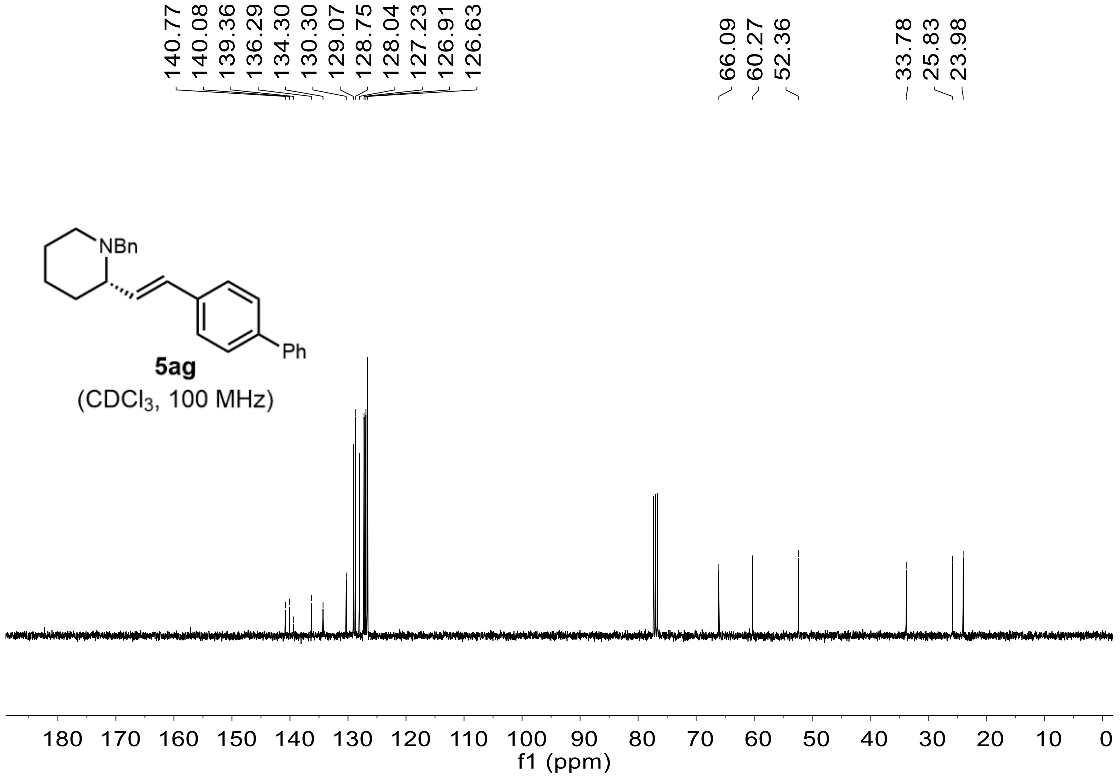
140.77 140.08 139.36 136.29 134.30 130.30 129.07 128.75 128.04 127.23 126.91 126.63
66.09 60.27 52.36
33.78 25.83 23.98
NBn
Ph
5ag
(CDCl3, 100 MHz)
180 170 160 150 140 130 120 110 100 90 80 70 60 50 40 30 20 10 0
f1 (ppm)

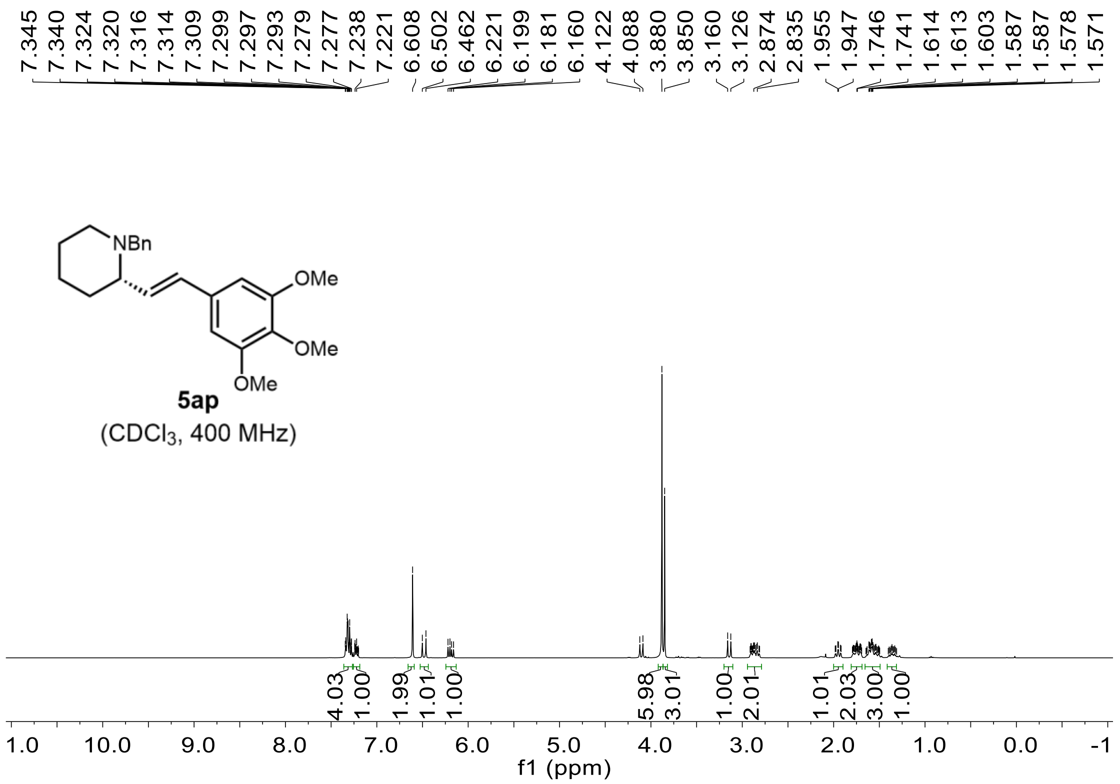
7.345 7.340 7.324 7.320 7.316 7.314 7.309 7.299 7.297 7.293 7.279 7.277 7.238 7.221 6.608 6.502 6.462 6.221 6.199 6.181 6.160 4.122 4.088 3.880 3.850 3.160 3.126 2.874 2.835 1.955 1.947 1.746 1.741 1.614 1.613 1.603 1.587 1.587 1.578 1.571
NBn
OMe
OMe
OMe
5ap
(CDCl3, 400 MHz)
4.03 1.00 1.99 1.01 1.00 5.98 3.01 1.00 2.01 1.01 2.03 3.00 1.00
f1 (ppm)

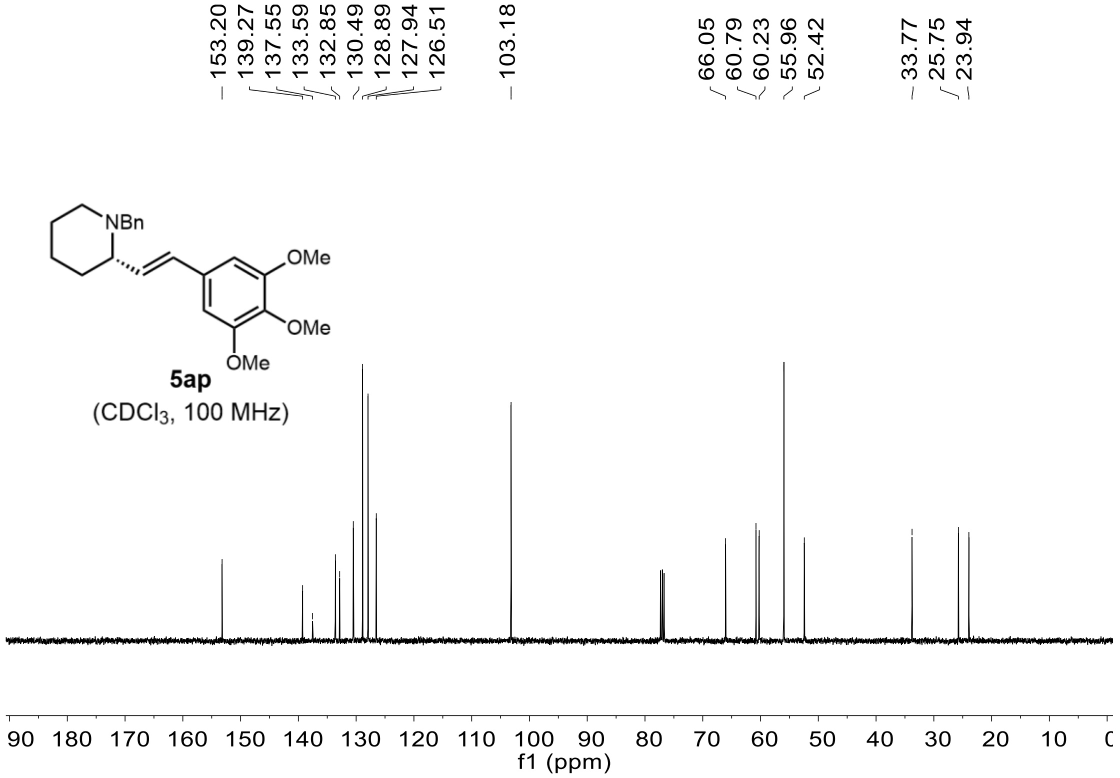
153.20 139.27 137.55 133.59 132.85 130.49 128.89 127.94 126.51 103.18 66.05 60.79 60.23 55.96 52.42 33.77 25.75 23.94
NBn
OMe
OMe
OMe
5ap
(CDCl3, 100 MHz)
f1 (ppm)

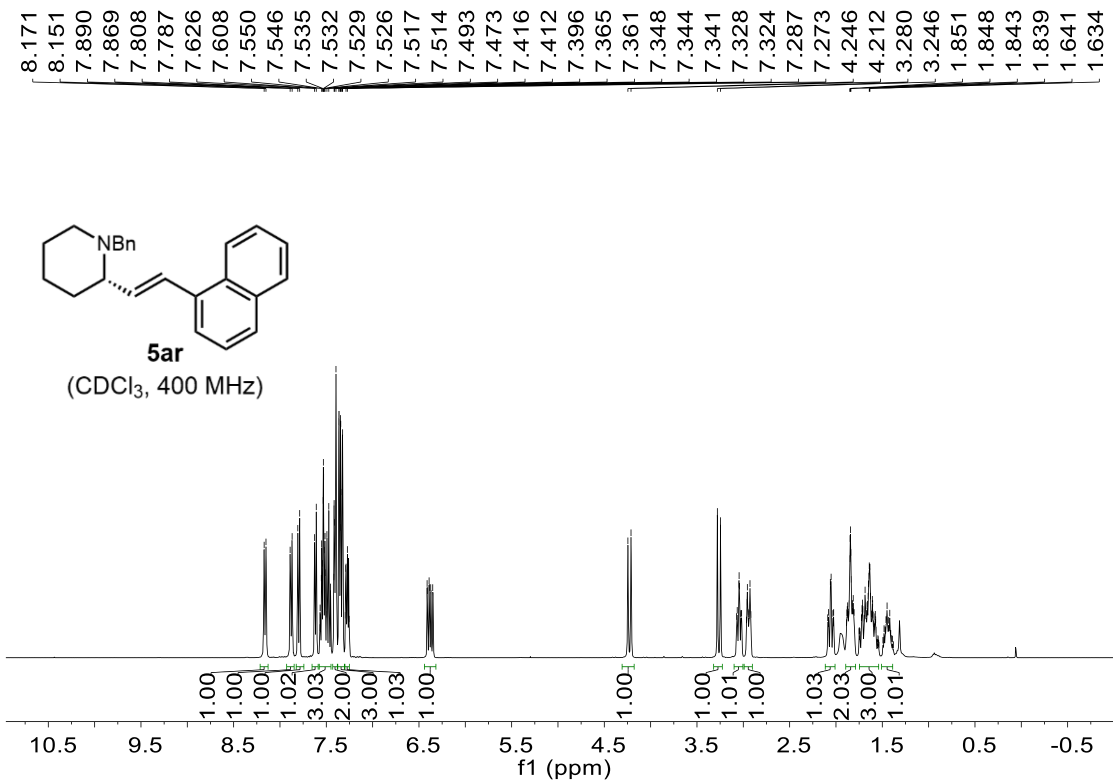
8.171 8.151 7.890 7.869 7.808 7.787 7.626 7.608 7.550 7.546 7.535 7.532 7.529 7.526 7.517 7.514 7.493 7.473 7.416 7.412 7.396 7.365 7.361 7.348 7.344 7.341 7.328 7.324 7.287 7.273 4.246 4.212 3.280 3.246 1.851 1.848 1.843 1.839 1.641 1.634
NBn
5ar
(CDCl$_3$, 400 MHz)
1.00 1.00 1.00 1.02 3.03 2.00 3.00 1.03 1.00
1.00
1.00 1.01 1.00
1.03 2.03 3.00 1.01
10.5 9.5 8.5 7.5 6.5 5.5 4.5 3.5 2.5 1.5 0.5 -0.5
f1 (ppm)

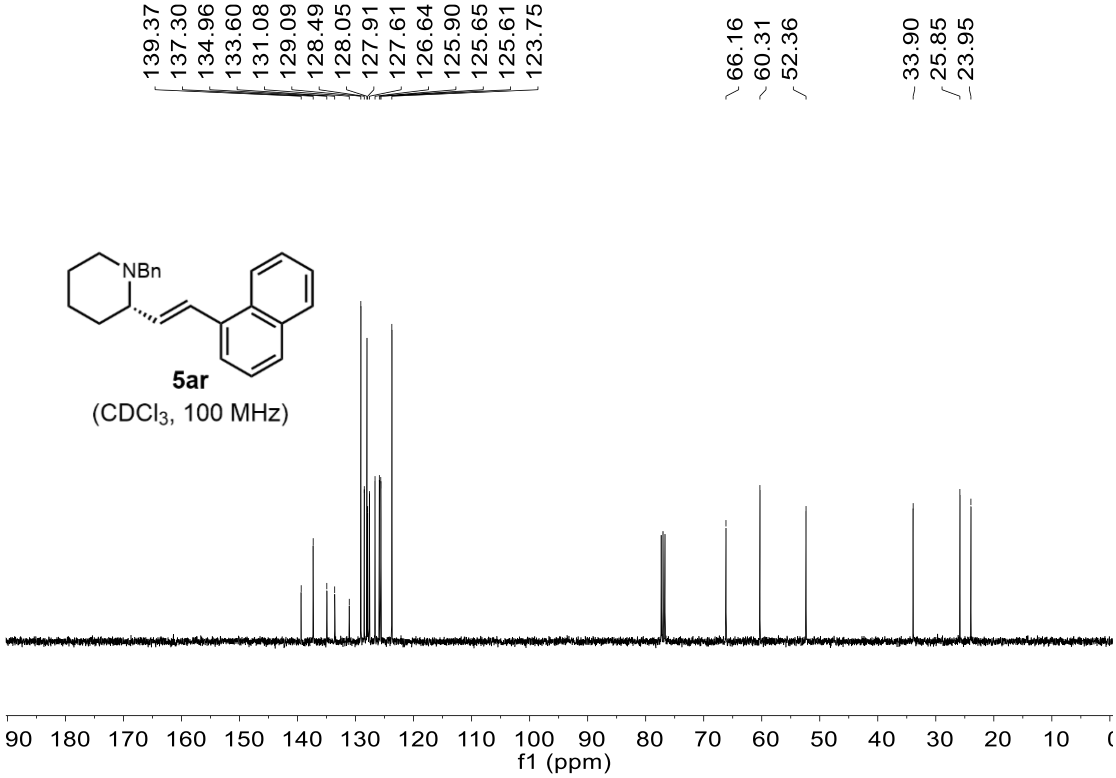
139.37 137.30 134.96 133.60 131.08 129.09 128.49 128.05 127.91 127.61 126.64 125.90 125.65 125.61 123.75
66.16 60.31 52.36
33.90 25.85 23.95
NBn
5ar
(CDCl$_3$, 100 MHz)
90 180 170 160 150 140 130 120 110 100 90 80 70 60 50 40 30 20 10
f1 (ppm)

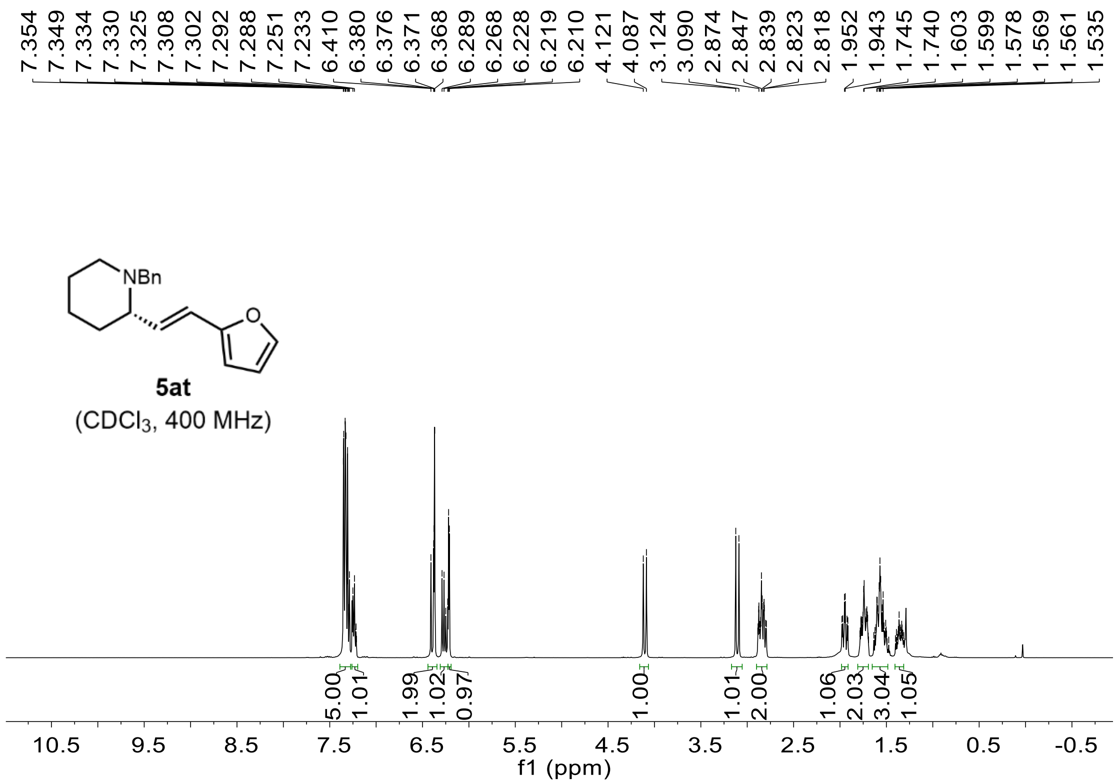
7.354 7.349 7.334 7.330 7.325 7.308 7.302 7.292 7.288 7.251 7.233 6.410 6.380 6.376 6.371 6.368 6.289 6.268 6.228 6.219 6.210 4.121 4.087 3.124 3.090 2.874 2.847 2.839 2.823 2.818 1.952 1.943 1.745 1.740 1.603 1.599 1.578 1.569 1.561 1.535
NBn
O
5at
(CDCl3, 400 MHz)
5.00 1.01 1.99 1.02 0.97 1.00 1.01 2.00 1.06 2.03 3.04 1.05
10.5 9.5 8.5 7.5 6.5 5.5 4.5 3.5 2.5 1.5 0.5 -0.5
f1 (ppm)

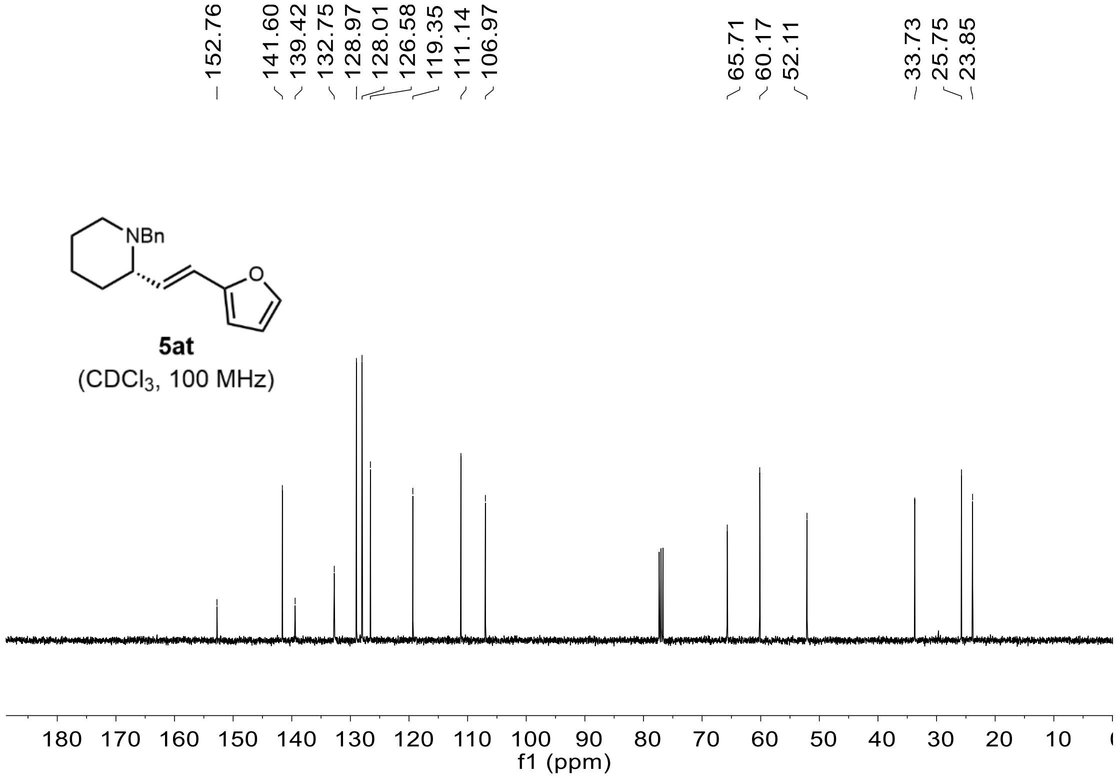
152.76 141.60 139.42 132.75 128.97 128.01 126.58 119.35 111.14 106.97 65.71 60.17 52.11 33.73 25.75 23.85
NBn
O
5at
(CDCl3, 100 MHz)
180 170 160 150 140 130 120 110 100 90 80 70 60 50 40 30 20 10
f1 (ppm)

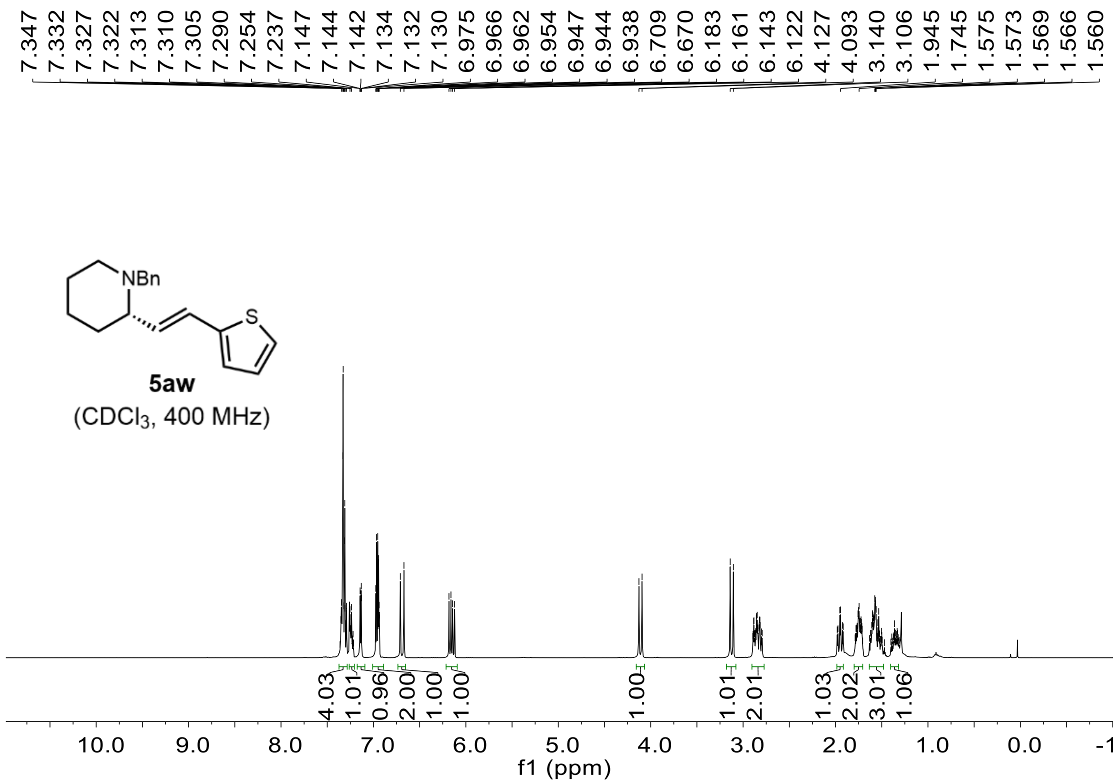
7.347
7.332
7.327
7.322
7.313
7.310
7.305
7.290
7.254
7.237
7.147
7.144
7.142
7.134
7.132
7.130
6.975
6.966
6.962
6.954
6.947
6.944
6.938
6.709
6.670
6.183
6.161
6.143
6.122
4.127
4.093
3.140
3.106
1.945
1.745
1.575
1.573
1.569
1.566
1.560
NBn
S
5aw
(CDCl3, 400 MHz)
4.03
1.01
0.96
2.00
1.00
1.00
1.00
1.01
2.01
1.03
2.02
3.01
1.06
10.0
9.0
8.0
7.0
6.0
5.0
4.0
3.0
2.0
1.0
0.0
-1
f1 (ppm)

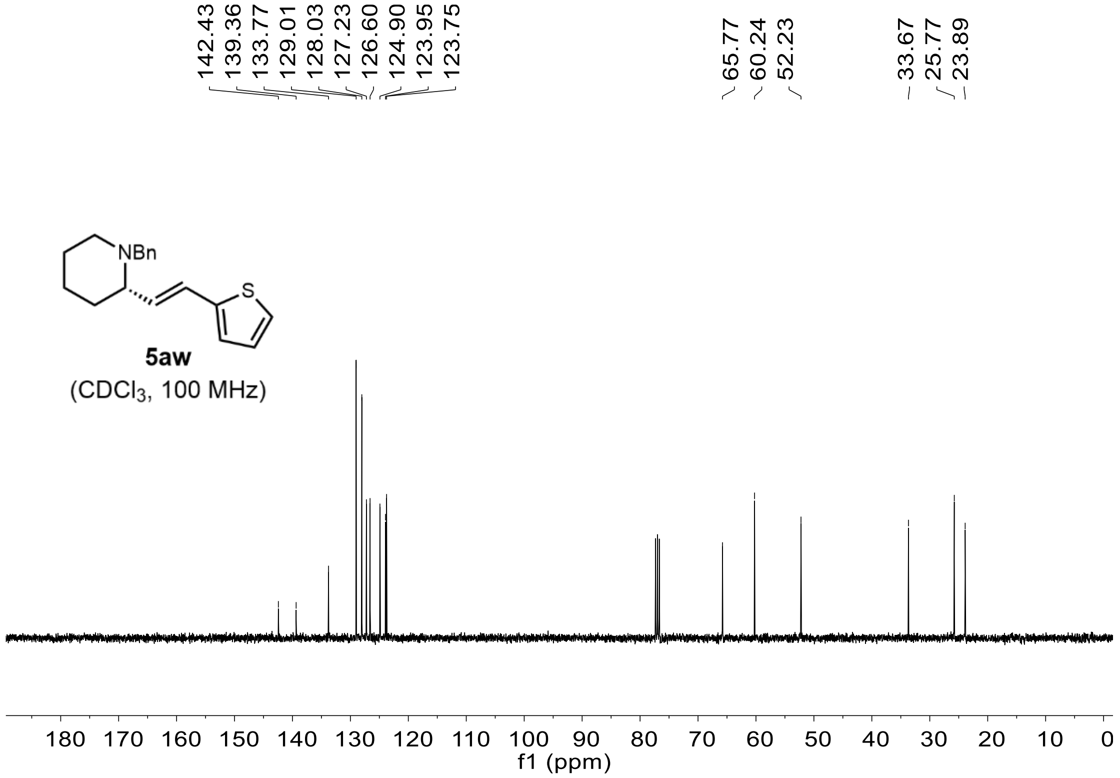
142.43
139.36
133.77
129.01
128.03
127.23
126.60
124.90
123.95
123.75
65.77
60.24
52.23
33.67
25.77
23.89
NBn
S
5aw
(CDCl3, 100 MHz)
180
170
160
150
140
130
120
110
100
90
80
70
60
50
40
30
20
10
0
f1 (ppm)

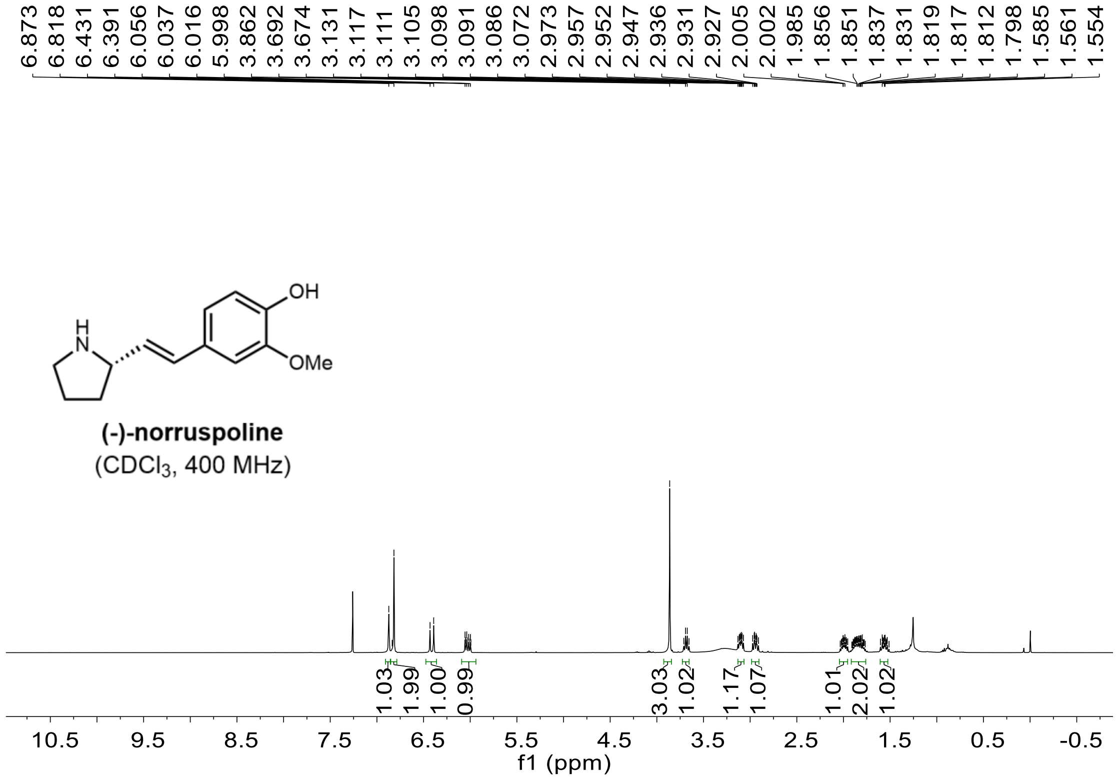
6.873 6.818 6.431 6.391 6.056 6.037 6.016 5.998 3.862 3.692 3.674 3.131 3.117 3.111 3.105 3.098 3.091 3.086 3.072 2.973 2.957 2.952 2.947 2.936 2.931 2.927 2.005 2.002 1.985 1.856 1.851 1.837 1.831 1.819 1.817 1.812 1.798 1.585 1.561 1.554
OH
OMe
(-)-norruspoline
(CDCl3, 400 MHz)
1.03 1.99 1.00 0.99 3.03 1.02 1.17 1.07 1.01 2.02 1.02
10.5 9.5 8.5 7.5 6.5 5.5 4.5 3.5 2.5 1.5 0.5 -0.5
f1 (ppm)

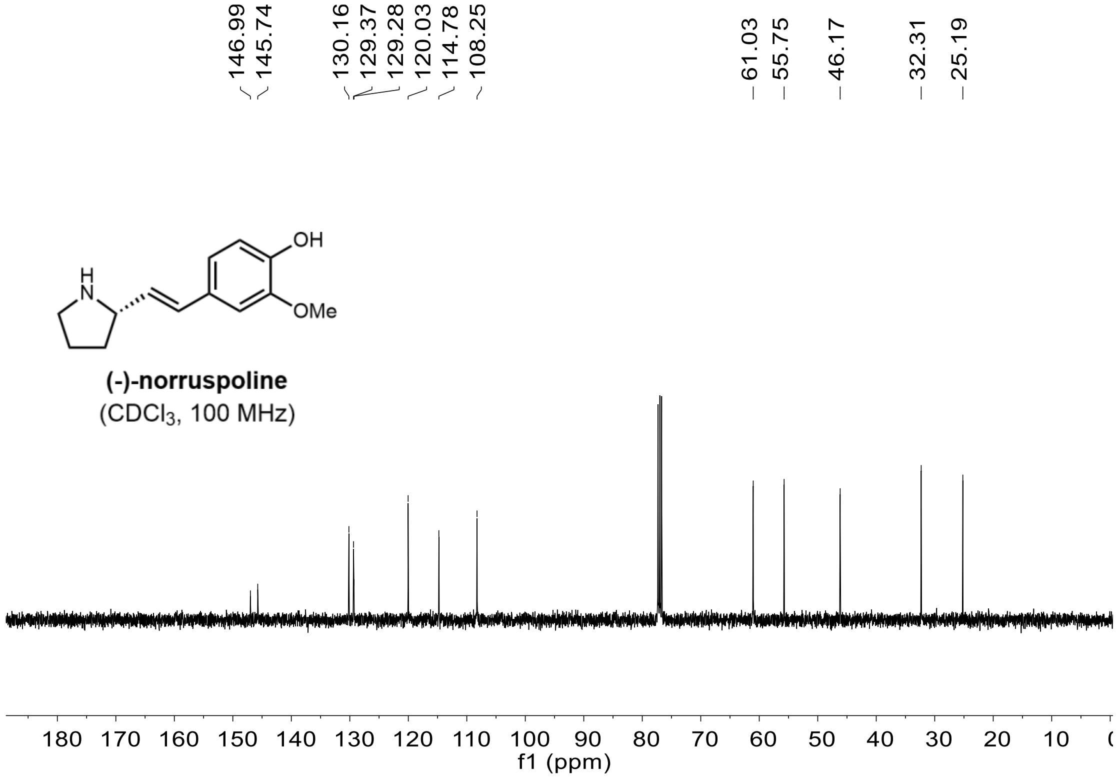
146.99 145.74 130.16 129.37 129.28 120.03 114.78 108.25 61.03 55.75 46.17 32.31 25.19
OH
OMe
(-)-norruspoline
(CDCl3, 100 MHz)
180 170 160 150 140 130 120 110 100 90 80 70 60 50 40 30 20 10
f1 (ppm)
